# Cross-coupling of organic fluorides with allenes: a silyl-radical-relay pathway for the construction of α-alkynyl-substituted all-carbon quaternary centres†

**DOI:** 10.1039/d3sc06617g

**Published:** 2024-03-01

**Authors:** Jun Zhou, Zhengyu Zhao, Soichiro Mori, Katsuhiro Yamamoto, Norio Shibata

**Affiliations:** a Department of Nanopharmaceutical Sciences, Nagoya Institute of Technology Gokiso, Showa-ku Nagoya 466-8555 Japan nozshiba@nitech.ac.jp; b Department of Life Science and Applied Chemistry, Nagoya Institute of Technology Gokiso, Showa-ku Nagoya 466-8555 Japan

## Abstract

Controlling the transformation of versatile and reactive allenes is a considerable challenge. Herein, we report an efficient silylboronate-mediated cross-coupling reaction of organic fluorides with allenes to construct a series of sterically demanding α-ethynyl-containing all-carbon quaternary centers (ACQCs), using catalyst-free silyl-radical-relay reactions to selectively functionalize highly inert C–F bonds in organic fluorides. The key to the success of this transformation lies in the radical rearrangement of an *in situ*-generated allenyl radical to form a bulky tertiary propargyl radical; however, the transformation does not show efficiency when using the propargyl isomer directly. This unique reaction enables the cross-coupling of a tertiary carbon radical center with a C(sp^2^)–F bond or a benzylic C(sp^3^)–F bond. α-Ethynyl-containing ACQCs with (hetero)aromatic substituents and benzyl were efficiently synthesized in a single step using electronically and sterically diverse organic fluorides and allenes. The practical utility of this protocol is showcased by the late-stage functionalization of bioactive molecules and the modification of a liquid crystalline material.

## Abstract 2

Cross-coupling of organic fluorides with allenes via radical rearrangement to afford all-carbon quaternary centres mediated by silylboronate/potassium *tert*-butoxide is disclosed.

## Graphical abstract

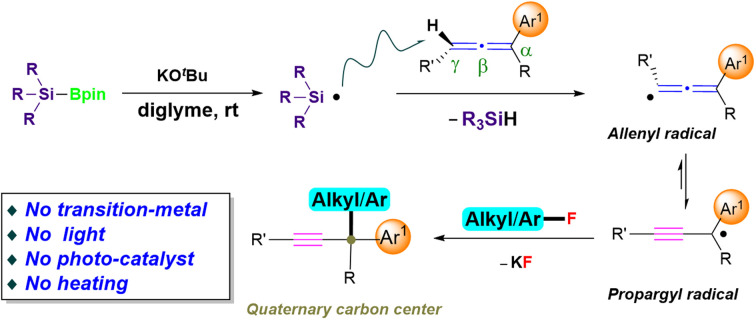

## Introduction

All-carbon quaternary centers (ACQCs) exhibit rigidity and structural diversity and are key structural units that occur frequently in many natural products, pharmaceuticals, and bioactive molecules.^1^ Moreover, at least 12% of the 200 top-selling prescription drugs in the US since 2011 contain a stereo-quaternary carbon center.^2^ Therefore, the construction of ACQCs presents a quite attractive challenge for organic synthetic chemists.^3^ In particular, ACQCs that contain an alkyne moiety serve as versatile intermediates and basic functionalized groups in organic transformations.^4^ Generally, the synthetic methods for alkyne-containing ACQCs involve either the direct introduction of alkynyl moieties into target molecules^5^ or the transformation from halogenated allenes in the presence of Knochel reagents.^6^ However, the aforementioned methodologies are associated with major drawbacks, including the use of transition-metal (TM) catalysts, dimerization of terminal alkynes, β-H elimination of branched tertiary alkyl units, and/or the reliance on special functionalized precursors. Therefore, the development of efficient strategies to overcome these limitations and the extremely strong steric effect to realize alkynyl-substituted ACQCs through C–C bond coupling reactions remains challenging.

C–C bond formation is a perpetual subject of interest in organic chemistry and represents one of the most important transformations for the manufacture of products used in daily life.^7^ The majority of C–C bond coupling reactions require TM catalysts and/or organic (pseudo)halides (Ar/alkyl–X; X = *e.g.*, I, Br, Cl, OTf, OMs).^8^ However, organic fluorides are rarely employed as coupling partners because the C–F bond is rather inert and has a higher bond dissociation energy (BDE) (*e.g.*, fluorobenzene: 126 kcal mol^−1^; 1-fluoropropane: 114 kcal mol^−1^) compared with the corresponding C–I/Br/Cl bonds.^9^ Additionally, with the rapid development of synthetic methodologies,^10^ the abundance and ready accessibility of organofluorine compounds^11^ make them attractive functional moieties as well as building blocks for further organic transformations.

**Fig. 1:**
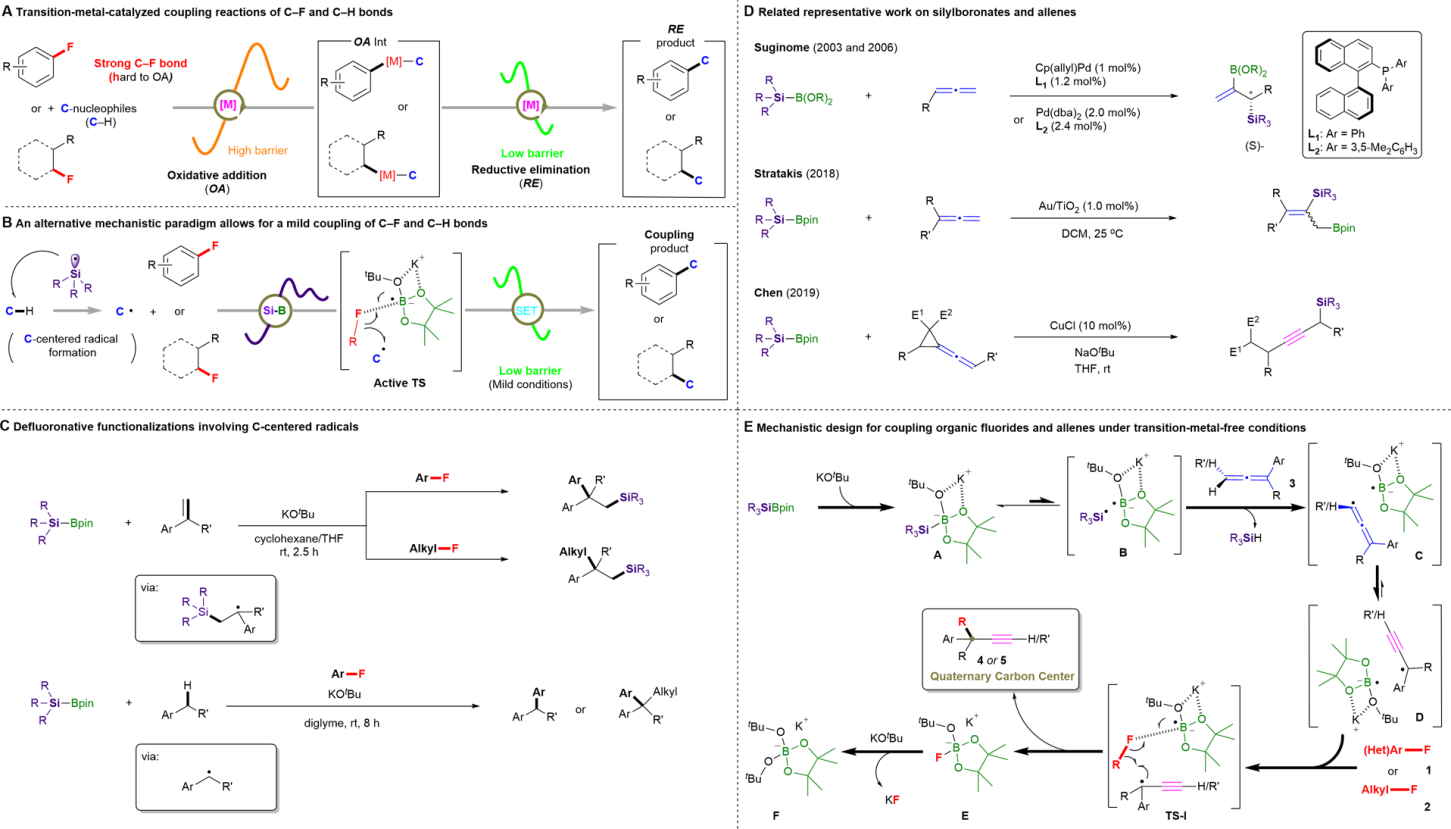
Cross-couplings of C–F and C–H bonds. (A) Transition-metal-catalyzed coupling reactions of C–F and C–H bonds. (B) A low-barrier cross-coupling of C–F and C–H bonds enabled by silylboronate and KO^*t*^Bu. (C) Our previous defluoronative functionalization work involving C-centered radicals using silylboronates and organic fluorides. (D) Related representative work on silylboronates and allenes. (E) Mechanistic design for the cross-coupling of organic fluorides and allenes under transition-metal-free conditions (this work).

Yet, the activation of robust C–F bonds remains a major challenge in contemporary chemistry. In this context, TM catalysis has proven a promising strategy for the direct functionalization of otherwise unreactive C–F bonds *via* the oxidative addition of C–F bonds to TMs followed by selective functionalization, providing access to the desired defluorinated molecules.^12^ However, the selective activation of C–F bonds usually either suffers from high oxidative-addition barriers, thus the employment of highly elaborate TM catalysts^13^ and/or forcing conditions^14^ is often indispensable, or confined to multi/poly-fluorinated arenes^15^ (Fig. 1A). Therefore, an alternative method that efficiently bypasses the high barriers required for the progress of the oxidative addition and that promotes the smooth coupling of C–F and C–H bonds under mild conditions would make a great contribution to this field.

Specifically, we envisioned that *in situ*-generated silyl radicals would quickly abstract a proton from targeted C–H reactants to form the corresponding C-centered radicals, thus enabling subsequent catalyst-free transformations with organic fluorides *via* the activation of an inert C–F bond to realize C–C coupling products (Fig. 1B). Such silyl-radical-relay reactions have already been demonstrated by our recently reported TM-free silylboronate (R_3_SiBpin)-mediated cross-couplings of organic fluorides with alkenes/arylmethanes (Fig. 1C).^16^

Recently, the unique structural features and versatile reactivity of allenes enabled them to be not only a versatile functional group that is incorporated in natural products, pharmaceuticals, and organic materials,^17^ but also serve as an ideal platform for the development of new methodologies in synthetic transformations, chiral ligands or even catalysts.^18^ In this context, reactions between silylboronates and allenes have been pioneered by the Suginome group^19^ and further explored by Stratakis;^20^ however, most hitherto reported protocols require TM catalysts and many provide silaboration products with one double bond retained. To date, only the Chen group^21^ has reported the successful transformation of vinylidene cyclopropanes into propargylic silanes in the presence of copper(i) chloride and NaO^*t*^Bu (Fig. 1D). Nevertheless, the silyl moiety is involved in the final products.

Based on these previous results, we designed a new silylboronate-mediated cross-coupling of organic fluorides with allenes to access ACQCs that feature an α-ethynyl group *via* C–F bond activation and radical rearrangement at room temperature (Fig. 1E).^16,22^ Initially, R_3_SiBpin and potassium *tert*-butoxide (KO^*t*^Bu) in an ether-based solvent smoothly generate intermediate A. Owing to the radical-initiation properties of KO^*t*^Bu^23^ and the steric demand of intermediate A, A splits into the bulky frustrated radical pair B,^24^ which consists of a trialkylsilyl radical (˙SiR_3_) and a boron-radical species (B˙), *via* homolytic cleavage of the Si–B bond. The silyl radical in B then directly abstracts a hydrogen atom from the allene (3) to form allenyl radical-containing frustrated radical pair C, which could easily isomerize to the sterically highly demanding propargylic radical-containing frustrated radical pair D.^25^ Subsequently, D would attract organic fluoride 1 or 2 by preferential interaction between the F atom and the B center to afford TS-I. Finally, the desired ethynyl-containing product with a quaternary carbon center (4 or 5) would be obtained *via* C–C bond coupling, accompanied by the release of E ([Bpin(O^*t*^Bu)F]K), which would promptly react with another equivalent of KO^*t*^Bu to provide a stable [Bpin(O^*t*^Bu)_2_] species and KF.

## Results and discussion

### Silylboronate-mediated cross-coupling reactions of aryl fluorides and aryl allenes

As depicted in our mechanistic hypothesis for the proposed C–C coupling (Fig. 1E), we expected that a silyl radical generated from the silylboronate and KO^*t*^Bu could abstract a hydrogen atom from the allene terminal. Therefore, we initiated the cross-coupling reactions by using 4-fluorobiphenyl (1a) and penta-1,2-dien-3-ylbenzene (3a) as model substrates. The desired product, 4-(3-phenylpent-1-yn-3-yl)biphenyl (4aa), which possesses a quaternary carbon center with an α-ethynyl moiety, was obtained in 34% yield under the standard reaction conditions [Et_3_SiBpin (2.0 equiv.), KO^*t*^Bu (4.0 equiv.), THF, room temperature; entry 1, Table 1]. Furthermore, among the silylboronates tested under the same conditions, the Suginome reagent (PhMe_2_SiBpin) improved the yield of 4aa to 41% (entry 2). Subsequent optimization focused on screening the number of reactant equivalents, solvent, and reaction time in the presence of PhMe_2_SiBpin and finally afforded 4aa in 94% yield under the optimized conditions (entry 3; for details, see the ESI; Tables S1–S4†). Control experiments showed that the reaction did not proceed in the absence of silylboronate or KO^*t*^Bu (entries 4). Moreover, the desired product was not obtained using other bases such as KOMe, NaO^*t*^Bu, LiO^*t*^Bu, or KHMDS (entries 5 and 6). This indicated that the countercations in MO^*t*^Bu exhibited superior ability for K^+^ over Na^+^ or Li^+^ in facilitating this transformation may lie in its established capacity to function as a single-electron reductant.^23^ Decreasing the amount of PhMe_2_SiBpin or KO^*t*^Bu resulted in lower yields of 4aa (86% and 51%, respectively; entries 7 and 8). Replacing PhMe_2_SiBpin with Et_3_SiBpin under otherwise identical optimal conditions gave 4aa in only 75% yield (71% isolated; entry 9). Additionally, the use of an inadequate amount of 3a (2.0 equiv.) had a negative effect on the reaction yield (entry 10).

**Table 1:** Optimization of the cross-coupling conditions^a^

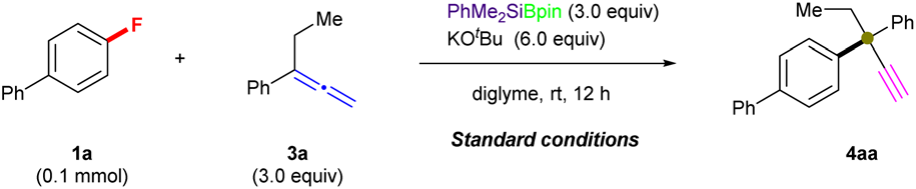		
Entry	Variation from the “standard conditions”	4aa^b^ (%)
1	Et_3_SiBpin (2.0 equiv.), KO^*t*^Bu (4.0 equiv.), THF	34
2	PhMe_2_SiBpin (2.0 equiv.), KO^*t*^Bu (4.0 equiv.), THF	41
3	None	94
4	Without PhMe_2_SiBpin or KO^*t*^Bu	0
5	KOMe instead of KO^*t*^Bu	0
6	NaOtBu, LiOtBu or KHMDS instead of KO^*t*^Bu	Trace
7	PhMe_2_SiBpin (2.5 equiv.) and KO^*t*^Bu (5.5 equiv.) instead	86
8	PhMe_2_SiBpin (2.0 equiv.) and KO^*t*^Bu (4.0 equiv.) instead	51
9^c^	Et_3_SiBpin instead of PhMe_2_SiBpin	75(71)
10	3a (2.0 equiv.) instead	78
11	PhMe_2_SiBpin (1.5 equiv.) and KO^*t*^Bu (3.0 equiv.) and 3a (1.5 equiv.) at 50 °C	79
12	Et_3_SiBpin (1.5 equiv.) and KO^*t*^Bu (3.0 equiv.) and 3a (1.5 equiv.) at 50 °C	38

a Unless otherwise noted, the standard reactions refer to: 1a (17.2 mg, 0.1 mmol), 3a (43.2 mg, 0.3 mmol), PhMe_2_SiBpin (78.7 mg, 0.3 mmol), KO^*t*^Bu (67.2 mg, 0.6 mmol), and diglyme (1.0 mL); room temperature; 12 h.

b Determined by ^19^F NMR and ^1^H NMR spectroscopy using 3-fluoropyridine as the internal standard.

c The isolated yield is shown in parentheses.

It should also be noted here that the Suginome reagent usually generates the undesired side product 1,2-di-*tert*-butoxy-1,1,2,2-tetramethyldisilane^26^ (same polarity as 4aa), which is formed by the dimerization of the *tert*-butoxydimethylsilyl radical (^*t*^BuOMe_2_Si˙), which renders the purification of 4aa difficult *via* column chromatography on silica gel. Instead, the use of Et_3_SiBpin afforded pure 4aa, albeit in a lower yield. Furthermore, the slightly elevated reaction temperature (50 °C) could significantly reduce the consumption of both the Suginome reagent, KO^*t*^Bu, and allene 3a to half the amount, with 79% yield (entry 11). However, only a 38% yield was observed when PhMe_2_SiBpin was replaced by Et_3_SiBpin under the same conditions as in entry 11 (entry 12).

#### Scope and limitations

**Fig. 2:**
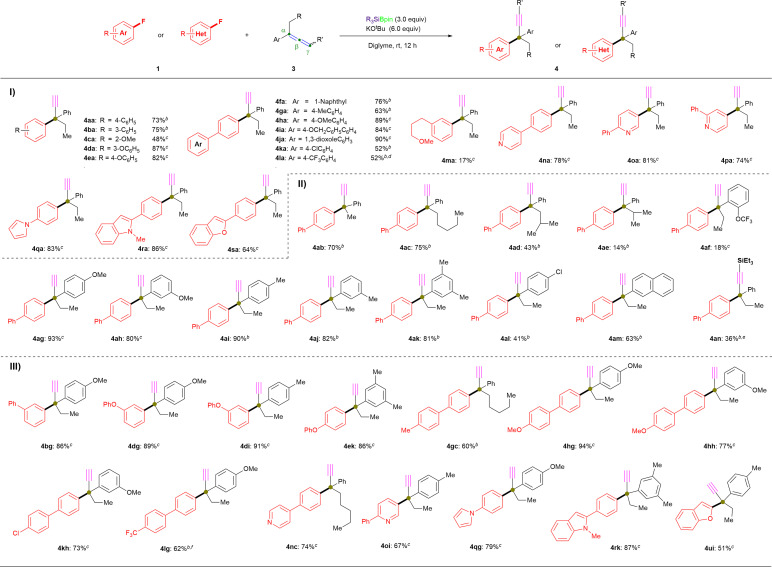
Substrate scope of 1 and 3. ^*a*^ Unless otherwise noted, all reactions were conducted using 1 (0.2 mmol), 3 (3.0 equiv.), R_3_SiBpin (3.0 equiv.), KO^*t*^Bu (135 mg, 6.0 equiv.), and diglyme (2.0 mL) at room temperature for 12 h, and isolated yields are shown. ^*b*^ Reactions were performed using Et_3_SiBpin. ^*c*^ Reactions were performed using PhMe_2_SiBpin. ^*d*^ Reaction was performed for 4 h. ^*e*^4aa was obtained in 61% yield. ^*f*^ Reaction was performed for 2 h.

Then, the substrate scope of this silylboronate-mediated defluoronative cross-coupling was further evaluated using the optimal reaction conditions (entries 3 and 9, Table 1) in the presence of PhMe_2_SiBpin or Et_3_SiBpin (Fig. 2). As shown in Fig. 2-I, (hetero)aryl fluorides with diverse electronic properties (1) were treated with 3a. First, five types of fluorobenzenes with different substituents (1a–1e), including the sterically hindered *ortho*-substituted substrate 1c, reacted efficiently with 3a under the optimal conditions to generate the corresponding cross-coupling amination products (4aa–4ea) in high yield (48–87%). For example, biphenyl (4aa: 73%; 4ba: 75%), *ortho*-OMe (4ca: 48%), and phenoxy (4da: 87%; 4ea: 82%) products were all obtained using this methodology. Furthermore, a wide range of fluorobenzenes that contain π-extended moieties with various electronic properties were efficiently converted into the corresponding ethynyl-containing defluorinative products with an ACQC (4fa–4la) in good-to-high yield (52–90%), which is virtually independent of the attached functional group. The use of *p*-substituted fluorobenzenes with electron-donating (1-naphthyl–: 1f; Me–: 1g; MeO–: 1h; BnO–: 1i; benzo[1,3]dioxol–: 1j) or electron-withdrawing groups (Cl–: 1k; CF_3_: 1l) successfully furnished the desired products (4fa: 76%; 4ga: 63%; 4ha: 89%; 4ia: 84%; 4ja: 90%; 4ka: 52%; 4la: 52%). The excellent chemoselectivity profile of this coupling reaction is nicely illustrated by the tolerance of the reaction conditions toward functional groups such as ethers, the Cl group, and even the CF_3_ group. However, simple fluorobenzene with a *meta*-substituted carbon chain (1m) only afforded a 17% yield of 4ma, which might be due to the active benzyl C–H bonds in 1m. Additionally, N-containing heteroaromatic fluorides (1n–1r) were successfully coupled with 3a under the same conditions in higher yield (≤86%). Pyridine derivatives (4na: 78%; 4oa: 81%; 4pa: 74%), a 1*H*-pyrrole derivative (4qa: 83%) and an indole derivative (4ra: 86%) were also obtained *via* these cross-coupling reactions. A benzofuran-containing aryl fluoride (1s) was also functionalized in good yield even though it contained several reactive aryl C(sp^2^)–H bonds, selectively yielding the corresponding product (4sa: 64%) *via* C–F bond cleavage. All results clearly demonstrate the remarkable functional-group tolerance of these silylboronate-mediated cross-coupling reactions of aryl fluorides and allene 3a.

Next, the scope of allenes (3) was examined *via* coupling with 1a under the standard conditions (Fig. 2-II). Initially, we evaluated α-position-substituted allenes (methyl: 3b; *n*-pentyl: 3c; iso-butyl: 3d; iso-propyl: 3e) with 1a using Et_3_SiBpin as the silylboronate reagent, which provided the coupling products (4ab: 70%; 4ac: 75%) in similar yield, while the yields of the sterically hindered products (4ad: 43%; 4ae: 14%) were significantly decreased. Similarly, *ortho*-OCF_3_-substituted allene 3f also successfully afforded defluoronative product 4af, albeit only in 18% yield. Moreover, allenes that bear electron-donating (4-OMe: 3g; 3-OMe: 3h; 4-Me: 3i; 3-Me: 3j; 3,5-di-Me: 3k), electron-withdrawing (4-Cl: 3l) or 2-naphthyl (3m) groups underwent defluoroamination to afford the desired products in good-to-high yield (4ag: 93%; 4ah: 80%; 4ai: 90%; 4aj: 82%; 4ak: 81%; 4al: 41%; 4am: 63%). Moreover, triethylsilyl-substituted allene at the γ-position (3n) was evaluated with 1a under the standard reaction conditions (entry 8, Table 1); however, 3n afforded a mixture of 4an (36% yield) and 4aa (61% yield). Other γ-position-substituted allenes (*n*-heptyl, 3p; phenyl, 3r; dimethyl, 3s) and purely aliphatic allene 3-ethylhepta-1,2-diene (3w) failed to afford any desired products, which should be attributed to the deprotonation of γ-C(sp^2^)–H in allenes 3 which is a crucial step in this transformation (for more details, see the ESI†). These above results strongly support our hypothesis of a radical-relay reaction, as shown in Fig. 1E, because the aryl moiety in allenes 3 increases the stability of the generated radical, which is indispensable for a successful transformation.

After evaluating the ranges of 1 and 3 in this coupling reaction, we then further demonstrated the scope of this feasible coupling reaction using various combinations of 1 and 3 (Fig. 2-III). Fluoroarenes with phenyl (1b), electron-donating (1d, 1e, 1g, and 1h), and electron-withdrawing (1k and 1l) substituents were coupled with various allenes to generate the desired α-ethynyl-substituted quaternary center products 4 in good yields (4bg: 86%; 4dg: 89%; 4di: 91%; 4ek: 86%; 4gc: 60%; 4hg: 94%; 4hh: 77%; 4kh: 73%; 4lg: 62%), and even the complete transformation of 1l into 4lg was achieved within 2 hours with the potentially cleavable C–F bonds of the trifluoromethyl group remaining intact. The reactions of *N*-heterocycle-containing aryl fluorides (1n, 1o, 1q, and 1r) with substituted-allenes (3c, 3g, 3i, and 3k) under standard conditions mediated by PhMe_2_SiBpin resulted in pyridine derivatives and 1*H*-pyrrole derivatives in good-to-high yields (4nc: 74%; 4oi: 67%; 4qg: 79%; 4rk: 87%). Additionally, 2-fluorobenzofuran (1u) is also an acceptable coupling partner, giving 4ui in a moderate yield of 51%.

**Fig. 3:**
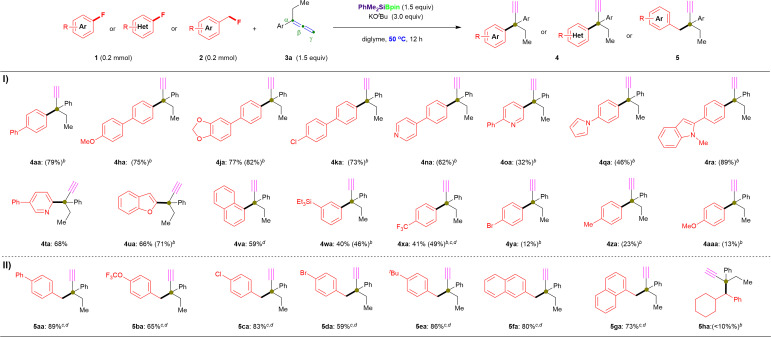
Further scopes and limitations. ^*a*^ Unless otherwise noted, all reactions were conducted using 1 or 2 (0.2 mmol), 3a (43.2 mg, 1.5 equiv.), PhMe_2_SiBpin (78.7 mg, 1.5 equiv.), KO^*t*^Bu (67.5 mg, 3.0 equiv.), and diglyme (1.5 mL) at 50 °C for 12 h. ^*b*^ The ^1^H NMR yields are shown in the parentheses. ^*c*^ Reactions were performed at room temperature. ^*d*^ Reactions were performed using Et_3_SiBpin.

Furthermore, we repeated the defluorinative cross-coupling reactions under the heating reaction conditions (entry 11, Table 1) by using representative aryl fluorides 1 with allene 3a (Fig. 3). First, as shown in Fig. 3-I, four types of π-extended aryl fluorides substituted with electron-donating (1a, 1h and 1j) and withdrawing (1k) groups were evaluated, and the desired α-ethynyl-containing ACQCs were observed in ^1^H NMR yields of ≤82% (4aa: 79%; 4ha: 75%; 4ja: 82%; 4ka: 73%) and with 77% isolated yield for 4ja. Notably, N-containing heteroaromatic fluorides (1n–1t) were successfully coupled with 3a under the same conditions (entry 11, Table 1) to afford the corresponding coupling products with an ACQC in moderate to high ^1^H NMR yields (4na: 62%; 4oa: 32%; 4qa: 46%; 4ra: 89%), whereas desired product 4ta could be obtained in 68% isolate yield by coupling active 2-fluoro-5-phenylpyridine (1t) and allene 3a. Similarly, 2-fluorobenzofuran (1u) and the simplest polycyclic aromatic fluoride (1v) were efficiently coupled with allene 3a to afford 4ua and 4va in 66% and 59% yield, respectively. Additionally, fluorobenzene substituted with triethylsilyl (1w), trifluoromethyl (1x), bromo (1y), methyl (1z), methoxyl (1aa), and *N*,*N*-dimethyl (1ab) were also coupled with allene 3a, albeit in low yields (4wa: 40% isolated yield; 4xa: 41% isolated yield; 4ya: 12%; 4za: 23%; 4aaa: 13%; 4aba: 0%). Thus, the lower yield regarding the substituted fluorobenzenes (especially for 1z, 1aa, and 1ab) could be explained by their C(sp^2^)–F bond possessing higher BDE than that of substituted biaryl fluorides. In this case, a complex mixture (containing 12 or 15) was obtained rather than the desired products with an ACQC. However, carbonyl containing fluorobenzenes, such as carboxylate (1ac), resulted in no desired product under the aforementioned conditions with or without heating (Fig. 3-I).

To our delight, the cross-coupling of benzyl fluorides 2 with allene 3a under similar reaction conditions while at room temperature were also successful in affording desired products with ACQCs (Fig. 3-II). Primary benzyl fluorides bearing electron-donating substituents (phenyl: 2a; *tert*-butyl: 2e) and electron-withdrawing substituents (trifluoromethoxy: 2b; chloro: 2c; bromo: 2d) reacted efficiently with allene to afford the corresponding products in up to 89% yield (5aa: 89%; 5ba: 65%; 5ca: 83%; 5da: 59%; 5ea: 86%). Additionally, the naphthyl-containing primary fluorides (2f and 2g) successfully yielded the desired products (5fa: 80%; 5ga: 73%) in good yields regardless of the α/β-substituted position on the naphthalene ring. However, secondary alkyl fluorides 2h furnished the desired coupling product 5ha in <10% ^1^H NMR yield, even when the reaction was performed at 50 °C. Additionally, the primary aliphatic alkyl fluorides (2i and 2j) did not afford the corresponding products even under heating.

#### Synthetic applications

**Fig. 4:**
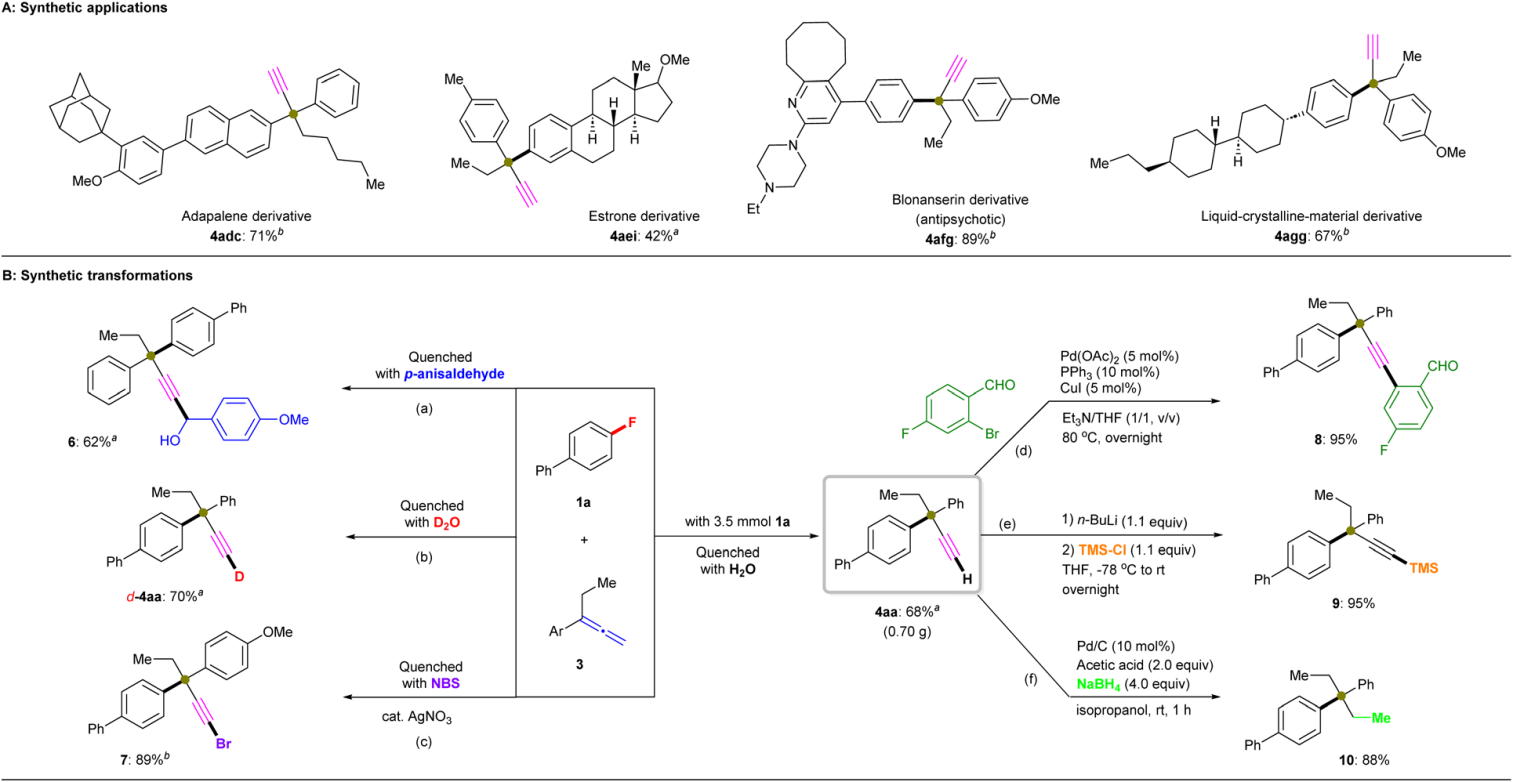
Synthetic utility. (A) Synthetic applications. Unless otherwise noted, all reactions were conducted using 1 (0.2 mmol), 3 (3.0 equiv.), R_3_SiBpin (3.0 equiv.), KO^*t*^Bu (135 mg, 6.0 equiv.), and diglyme (2.0 mL) at room temperature for 12 h (isolated yields are shown). (B) Synthetic transformations. ^*a*^ Reaction performed using Et_3_SiBpin. ^*b*^ Reaction performed using PhMe_2_SiBpin. (a) 1a (0.2 mmol), 3a (0.6 mmol), *p*-anisaldehyde (0.3 mmol); (b) 1a (0.2 mmol), 3a (0.6 mmol), D_2_O (2.0 mL); (c) 1a (0.2 mmol), 3g (0.6 mmol), NBS (0.4 mmol), AgNO_3_ (0.1 mmol); (d) 4aa (0.2 mmol), Pd(OAc)_2_ (0.01 mmol), PPh_3_ (0.02 mmol), CuI (0.01 mmol), aryl bromide (0.24 mmol), Et_3_N/THF (1.0 mL, 1/1, v/v), 80 °C, overnight; (e) 4aa (0.2 mmol), *n*-BuLi (0.24 mmol), TMS-Cl (0.22 mmol), THF (1.0 mL), −78 °C to rt, overnight; (f) 4aa (0.2 mmol), Pd/C (0.02 mmol), acetic acid (0.04 mmol), NaBH_4_ (0.8 mmol), rt, 1 h.

To highlight the synthetic applications of this silylboronate-mediated defluorinative coupling reaction, some easily accessible functional molecules with α-alkynylated quaternary centers were obtained after several drug derivatives and liquid-crystalline materials were evaluated under the standard conditions (Fig. 4A). Adapalene derivative 4adc with two substituents at the β-position was successfully obtained in 71% yield by coupling β-fluoronaphthyl-containing adapalene derivative 1ad with allene 3c. Steroid derivative 4aei was synthesized in 42% yield *via* the defluorinative coupling of fluoro-incorporated estrone derivative 1ae and 3i. Blonanserin-derived fluoroarene 1af underwent the coupling reaction with 3g to generate Blonanserin derivative 4afg in 89% yield. Moreover, the liquid-crystalline material 1ag was successfully functionalized using this transformation with 3g to give 4agg in 67% yield. The presence of the α-ethynyl group in these derivatives could potentially allow further late-stage functionalization.

#### Synthetic transformations

It is noteworthy that quenching with different reagents efficiently afforded a variety of products with functional moieties. Specifically, the cross-coupling of 1a with 3a under the standard conditions afforded 6 (62% yield) and *d*-4aa (70% yield) when quenched using *p*-anisaldehyde and deuterium oxide, respectively. Bromoethynyl derivative 7 (89% yield) was also successfully synthesized when the coupling reaction of 1a with 3g was quenched with *N*-bromosuccinimide (NBS) in the presence of a catalytic amount of silver(i) nitrate (Fig. 4B, left). Additionally, 4aa can serve as a versatile precursor for further synthetic transformations. First, a gram-scale reaction of 1a and 3a proceeded smoothly to afford 4aa in 68% yield under the standard conditions in the presence of Et_3_SiBpin. Thereafter, a dual catalyst (Pd(OAc)_2_/PPh_3_ and CuI) enabled the cross-coupling reaction of 4aa with a substituted arylbromide,^27^ which furnished the phenyl-coupling product 8 (95% yield) in the presence of Et_3_N in THF at 80 °C. Treatment of 4aa with trimethylsilyl chloride (TMS-Cl) in the presence of butyllithium furnished TMS-acetylene product 9 (95% yield).^28^ A Pd/C-catalyzed reduction of 4aa employing acetic acid/NaBH_4_ afforded hydrocarbon product 10 in 88% yield (Fig. 4B, right).^29^ These straightforward functionalization reactions significantly expand the scope and utility of these silyl-radical-relay cross-coupling reactions between aryl fluorides and allenes.

#### Mechanistic study

**Fig. 5:**
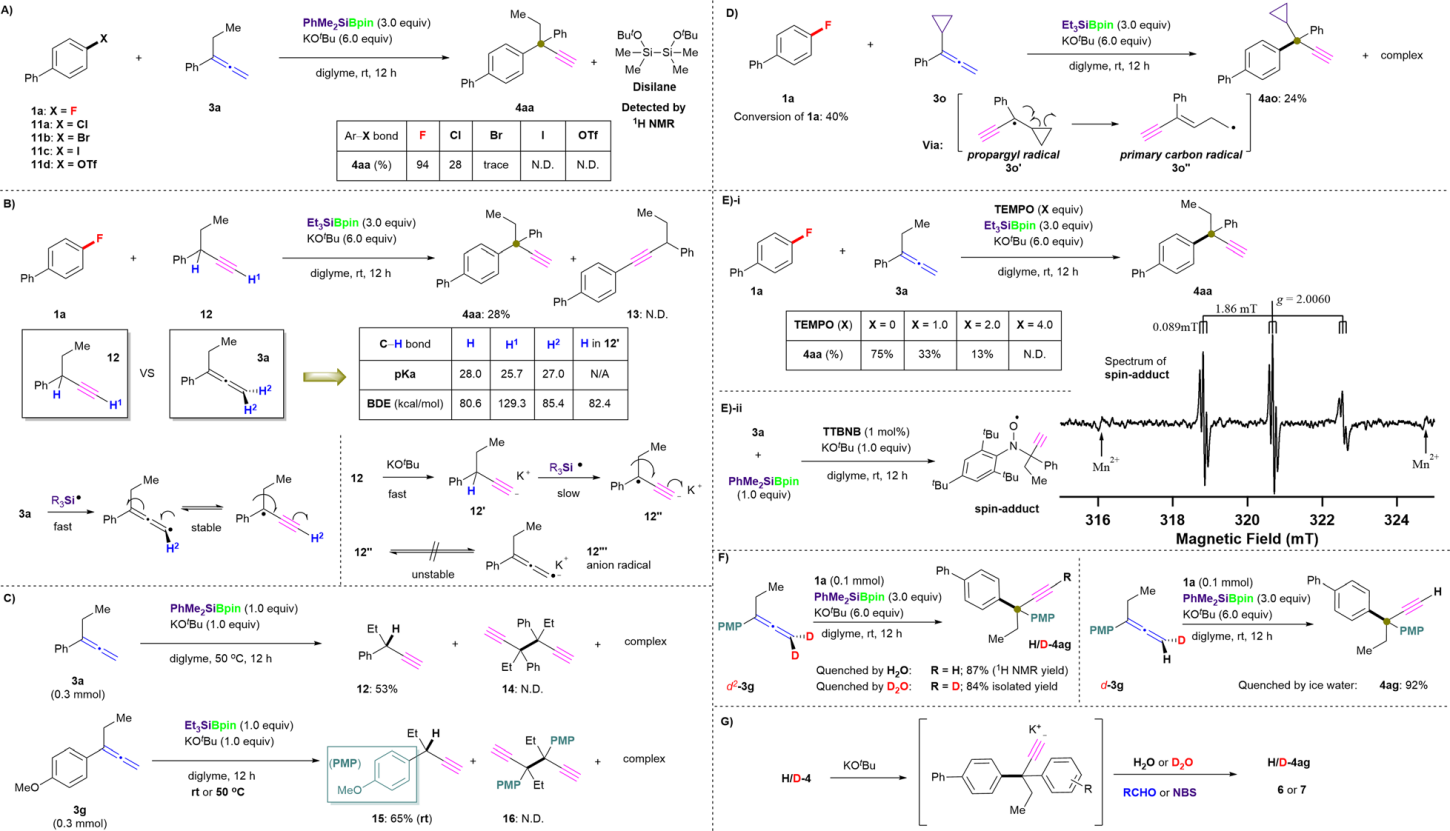
Mechanistic study. (A) Chemoselectivites of organic (pseudo)halides Ar–X. (B) An attempt to use an alternative process. (C) Homocoupling attempt of generated propargyl radicals. (D) Radical ring-opening reaction. (E) Effect of TEMPO on this silylboronate-mediated coupling reaction (i) and ESR experiment in the presence of spin trapping reagent TTBNB (ii). (F) Kinetic-isotope-effect experiments. (G) Possible mechanism for the quenching process.

To shed further light on the postulated coupling mechanism of aryl fluorides and allenes envisioned in Fig. 1E, several control experiments were conducted (Fig. 5). The uniqueness of this silylboronate-mediated cross-coupling reaction using aryl fluorides 1, rather than conventional aryl (pseudo)halides Ar–X 11a–d (X = Cl, Br, I, or OTf), was revealed *via* parallel experiments (Fig. 5A). 4-Chlorobiphenyl (11a) was converted to the desired coupling product 4aa in 28% yield under the same conditions (Table 1, entry 3). However, the use of bromo-, iodo-, or TfO-substituted biphenyl (Br: 11b; I: 11c; TfO: 11c) generated a complex mixture, in which the desired cross-coupling product 4aa was barely detected. Instead, the by-product 1,2-di-*tert*-butoxy-1,1,2,2-tetramethyldisilane largely accumulated (determined by ^1^H NMR; for details, see the ESI†) (Fig. 5A). We then attempted the reaction using the ethynyl isomer of 3a, *i.e.*, 3-phenyl-1-pentyne (12). While 3a and 12 are isomers of each other, their p*K*_a_ values differ significantly, and 12 should be more reactive under the basic conditions (p*K*_a_ of 12: H = 28.0; H^1^ = 25.7; p*K*_a_ of 3a: H^2^ = 27.0).^30^ Interestingly, the use of 1a and 12 under the standard conditions (for details, see the ESI†) only afforded 4aa in 28% ^1^H NMR yield, and alkynyl adduct 13 was not detected; instead, the corresponding defluorosilylation product, *i.e.*, 4-biphenyltriethylsilane (4-Ph-C_6_H_4_-SiEt_3_) was detected (Fig. 5B). This result excludes both the anionic *pre*-allenyl/propargyl-isomerization pathway from 3a to 12 and the anionic S_N_Ar pathway. In contrast, the predicted bond dissociation energy (BDE)^9^ of C–H^2^ in 3a (85.4 kcal mol^−1^) was higher than that of C–H (80.6 kcal mol^−1^) but much smaller than that of C–H^1^ in 12 (129.3 kcal mol^−1^). Thus, radical cleavage of C–H^2^ in 3a is preferable to that of C–H^1^ in 12, and the low yield of 4aa can be explained by the tertiary propargyl C–H bond in 12 which could be radically removed owing to its low BDE value; however, owing to the high acidity of C–H^1^ in 12, C–H^1^ is promptly deprotonated by KO^*t*^Bu to provide potassium acetylide 12′ (BDE of C–H in 12′: 82.4 kcal mol^−1^). Thus, the generation of tertiary propargylic radicals is slow because of the instability of the generated radical anion isomers. When allene 3a was treated with the Suginome reagent and KO^*t*^Bu at 50 °C in diglyme, only 12 was obtained (53% isolated yield). However, self-coupling dimer product 14 was not observed, which might be due to high steric repulsion. Similarly, allene 3g gave the same result using Et_3_SiBpin regardless of whether it reacted at room temperature or 50 °C, that is, 15 (65% isolated yield) without the observation of 16 (Fig. 5C). In addition, a radical-clock experiment employing 1a and α-cyclopropyl substituted allene (3o) was conducted. The conversion of 1a was only 40% (determined by ^19^F NMR; for details, see the ESI†), whereas 3o was fully consumed to give a complex mixture (Fig. 5D). Fortunately, the desired product (4ao) was isolated in 24% yield, which agrees with our ^1^H NMR analysis of the crude reaction mixture (for details, see the ESI†). The low yield of 4ao can be explained by the low reactivity of the primary carbon radical 3o′′ generated *via* the ring-opening process. We then evaluated the effect of (2,2,6,6-tetramethylpiperidin-1-yl)oxyl (TEMPO) on the coupling reaction between 1a and 3a under the optimal conditions in the presence of Et_3_SiBpin. Although 4aa was obtained in a yield of 75% (^1^H NMR yield) under standard conditions, the yield decreased significantly when the amount of TEMPO was increased (1.0 equiv. of TEMPO: 33%; 2.0 equiv. of TEMPO: 13%; 4.0 equiv. of TEMPO: 0%; Fig. 5E-i). It should be noted that the premixed silylboronate and KO^*t*^Bu were used to prevent TEMPO from being consumed by the reducing reagent silylboronate, followed by the addition of TEMPO and other materials. No desired product 4aa was detected, which indicated that the *in situ* generated silyl radical should be fully trapped by TMEPO (for details, see the ESI†). ESR experiments were also performed. Since we have already demonstrated the generation of silyl radicals from Et_3_SiBpin and KO^*t*^Bu under the same reaction conditions,^16*b*^ we tried to find the radical species derived from allene 3. As expected, the ESR spectrum (triple–triplet) was detected for the reaction of PhMe_2_SiBpin, KO^*t*^Bu, and allene 3a in diglyme at room temperature, which was assigned to the spin-adduct of the *tert*-propargyl radical trapped by 2,4,6-tri-*tert*-butylnitrosobenzene (TTBNB) (Fig. 5E-ii).^31^ Namely, the hyperfine splitting (hfs) constant due to nitrogen (*A*_N_; spin quantum number *I* = 1) was 1.86 mT, and the small splitting constant due to the two hydrogens at the *meta*-position of the TTBNB benzene ring (*A*_Hm_; *I* = 1/2) was 0.089 mT. The *g*-value of 2.006 was assigned to a nitroxide-type radical. Although further studies are required to show clear evidence, this observation strongly supports the formation of the propargyl radical (see the ESI† for details).

The kinetic-isotope effect of the C–H/C–D cleavage under ionic conditions is more substantially observed than that by the radical reactions.^32^ Thus, we evaluated 1a in combination with deuterated allenes (*d*^2^-3g and *d*-3g) in several parallel reactions under the standard conditions in the presence of PhMe_2_SiBpin (Fig. 5F). However, independent of the existence of deuterium in allene 3g, the formation of D- or H-4ag depends on the quenching method, *i.e.*, on using H_2_O or D_2_O, even when quenching with ice water. The formed products 4/5 should exist as potassium acetylides in the reaction mixture, as the excess of ^*t*^BuOK could easily result in a further deprotonation process. Therefore, the acetylide can be captured by D^+^, H^+^, Br^+^, RCHO, *etc.* (Fig. 5G). Besides, due to the high acidity of the C(sp)–H/D moiety in the terminal alkynyl position of 4ag, the H/D exchange occurs easily during the workup steps.^32,33^ Interestingly, the reaction time and yield were almost identical independent of the use of 3g, *d*^2^-3g, or *d*-3g. Therefore, we concluded that the C(sp^2^)–H/D bond cleavage should occur prior to the C–F bond cleavage, and thus the C(sp^2^)–H/D bond cleavage should not be the rate-limiting step. All observations in the mechanistic study led us to conclude that this defluorinative cross-coupling reaction proceeds *via* a single-electron transfer (SET)/radical process, in accordance with our mechanistic hypothesis shown in Fig. 1E.

## Conclusions

In summary, we have realized the first cross-coupling reaction of organic fluorides with allenes to construct a library of α-ethynyl-containing all-carbon quaternary centers *via* C–F bond and C(sp^2^)–H bond radical cross-couplings using a silyl-radical-relay strategy. The C–F bond cleavage occurs concomitant with the formation of an isomerized propargylic radical, which takes place through cleavage of a C(sp^2^)–H bond, to cooperatively form a new C–C(sp^3^) bond. A notable feature of this cross-coupling reaction is that the *in situ*-generated silyl radical is able to directly abstract a proton from a C(sp^2^)–H bond of the allene to form an allenyl radical, which then easily isomerizes to form a propargylic radical that exerts a more profound steric influence. Significantly, in this transformation it is not possible to use the corresponding propargyl isomers directly instead of the allenes. This method proceeds under very mild conditions and efficiently obviates the use of TM catalysis or light irradiation to allow a range of *para*-, *meta*-, and even *ortho*-substituted (hetero)aryl fluoride, benzyl fluoride and allene substrates to undergo the normally challenging defluorinative coupling process to afford all-carbon quaternary centers in moderate-to-excellent yield, with good functional-group compatibility and C–F bond selectivity. This radical-coupling system was further extended to the late-stage functionalization of several biologically active molecules.

## Data availability

The data that support the fndings of this study are available within the article and the ESI.† Details about materials and methods, experimental procedures, characterization data,mechanistic studies, ESR study and NMR spectral are included. All relevant data are also available from the authors.

## Author contributions

JZ optimized the reaction conditions. JZ and ZZ surveyed the substrate scope, analyzed the data, and then discussed the results with NS. JZ, ZZ, and SM prepared the starting materials. ZZ and KY performed the ESR experiment and analyzed the data. JZ and NS wrote the manuscript. NS supervised the project. All authors contributed to the manuscript and have approved the final version of the manuscript.

## Conflicts of interest

There are no conflicts to declare.

## Supplementary Material

SC-015-D3SC06617G-s001
