# Supplementary material for: Cross-coupling of organic fluorides with allenes: a silyl-radical-relay pathway for the construction of α-alkynyl-substituted all-carbon quaternary centres
Source: Chem Sci. 2024 Mar 1;15(14):5113–22. doi: 10.1039/d3sc06617g (PMC10988592; doi:10.1039/d3sc06617g)
Supplement: SC-015-D3SC06617G-s001 [file SC-015-D3SC06617G-s001.pdf]

Electronic Supplementary Material (ESI) for Chemical Science.
This journal is © The Royal Society of Chemistry 2024

Electronic Supplementary Information

Cross-coupling of organic fluorides with allenes: a silyl-radical-relay pathway for the construction of α-alkynyl-substituted all-carbon quaternary centres

Jun Zhou,[1] Zhengyu Zhao,[1] Soichiro Mori,[2] Katsuhiro Yamamoto,[2] and Norio Shibata[1,2,*]

[1]*Department of Nanopharmaceutical Sciences, Nagoya Institute of Technology, Gokiso, Showa-ku, Nagoya 466-8555, Japan*

[2]*Department of Life Science and Applied Chemistry, Nagoya Institute of Technology, Gokiso, Showa-ku, Nagoya, 466-8555, Japan.*

[*]*Correspondence and requests for materials should be addressed to N.S. (email: nozshiba@nitech.ac.jp)*

List of Contents

1. General Information and Materials 3
2. Experimental Details 4
    2.1. Procedures for the synthesis of starting materials 4
    2.2. Optimization of the reaction conditions 29
    2.3. General procedures for the cross-coupling of aryl fluorides and allenes 32
3. Characterization Data of Products 33
4. Synthetic Utility 65
    4.1 Quench with different nucleophiles 65
    4.2 Scale-up reaction 66
    4.3 Synthetic transformations 67
5. Preliminary Mechanistic Investigations 69
    5.1 Chemoselectivity of organic (pseudo)halides 69
    5.2 The attempt of an alternative process 70
    5.3 The NMR spectroscopic studies 71
    5.4 Reaction with radical scavenger TEMPO 73
    5.5 Radical ring-opening experiment 75
    5.6 Control experiments by using deuterated allenes 77
    5.7 ESR experiment 80
6. Unsuccessful Substrates 81
7. Copies of NMR Data (^{1}H NMR, ^{13}C NMR and ^{19}F NMR) 82
8. References 181

1. General Information and Materials

All reactions were performed in oven-dried glassware under a positive pressure of nitrogen or argon. Solvents were transferred via syringe and were introduced into the reaction vessels through a rubber septum. All solvents were dried by standard method. All the reactions were monitored by thin-layer chromatography (TLC) carried out on 0.25 mm Merck silica gel (60-F254). The TLC plates were visualized with UV light. All the reaction products were purified by column chromatography and was carried out on a column packed with silica gel 60N spherical neutral size 50-63 mm. The ^{1}H NMR (300 MHz), ^{13}C NMR (75 MHz), and ^{19}F NMR (282 MHz) spectra as for solution in $CDCl_3$ or D_2O were recorded on a Varian Mercury 300. ^{1}H NMR (500 MHz) and ^{13}C NMR (126 MHz) spectra as for solution in $CDCl_3$ was recorded on a BRUKER 500 Ultra Shield TR. ^{1}H NMR (700 MHz) and ^{13}C NMR (176 MHz) spectra as for solution in $CDCl_3$ was recorded on a JEOL RESONANCE ECZ700R. The chemical shifts (δ) are expressed in ppm downfield from internal TMS ($\delta = 0.00$) or $CDCl_3$ ($\delta = 7.26$) and coupling constants (J) are reported in hertz (Hz). The hexafluorobenzene (C_6F_6) [$\delta = -162.2$ ($CDCl_3$)] was used as internal standard for ^{19}F NMR. The following abbreviations were used to explain the multiplicities: s = singlet, d = doublet, t = triplet, q = quartet, m = multiplet, br = broad. Mass spectra were recorded on a JEOL JMS-Q1050GC (EI-MS) and SHIMADZU LCMS-2020 (ESI-MS). High resolution mass spectrometry (HRMS) was carried out on an electron impact ionization mass spectrometer with a micro-TOF analyzer and recorded on a Waters, GCT Premier (EI-MS) with a TOF analyzer. Infrared spectra were recorded on a JASCO FT/IR-4100 spectrometer. Melting points were recorded on a BUCHI M-565.

Commercially available chemicals were obtained from Aldrich Chemical Co., Alfa Aesar, TCI and used as received unless otherwise noted. Solvents 1,2-dimethoxyethane (DME), diethylene glycol dimethyl ether (diglyme), triethylene glycol dimethyl ether (triglyme), and tetrahydrofuran (THF) were dried and distilled before use.

2. Experimental Details

2.1. Procedures for the synthesis of starting materials

Synthesis of aryl fluorides 1

Fluoroarenes **1a**, **1c**, **1w**, **1x**, **1y**, **1z**, **1aa** and **1ag** were purchased from TCI. Fluoroarenes **1b**, **1f**, **1g**, **1h**, **1i**, **1j**, **1k**, **1l**, **1m**, **1n**, **1o**, **1p**, **1q**, **1r**, **1s**, **1t**, **1ab**, and **1ae** were prepared according to repeat previous methods.[1] A typical experimental procedure for the preparation of **1d**, **1e**, **1u**, **1ad**, and **1af** were described below.

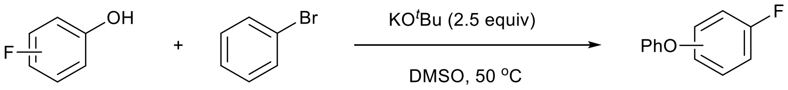

Fluoroarenes **1d** and **1e** were synthesized according to known procedures (**General procedure A**). A flame-dried flask was charged with fluorophenol, bromobenzene, and dry DMSO. Then KO^{t}Bu was added to the reaction flask portion-wise at room temperature, followed by stirring at 50 °C overnight. The reaction mixture was poured into water, and extracted with EtOAc, the combined organic phases were washed with brine, and dried over Na_2SO_4. After filtration, the filtrate was concentrated under reduced pressure to give the residue, which was purified by column chromatography on silica gel (*n*-hexane) to give corresponding products.

1-Fluoro-3-phenoxybenzene (1d)

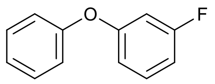

According to **General procedure A**, 3-fluorophenol (560 mg, 5.0 mmol), bromobenzene (1.0 mL, 10.0 mmol), dry DMSO (5.0 mL), and KO^{t}Bu (1.4 g, 12.5 mmol) were used as starting materials. And give title compound **1d** as colorless oil (527 mg, yield: 56 %).

^{1}H NMR (300 MHz, $CDCl_3$) δ 7.42 – 7.31 (m, 2H), 7.30 – 7.20 (m, 1H), 7.19 – 7.10 (m, 1H), 7.07 – 7.00 (m, 2H), 6.83 – 6.73 (m, 2H), 6.69 (dt, *J* = 10.3, 2.4 Hz, 1H).

^{19}F NMR (282 MHz, $CDCl_3$) δ -111.50 (q, *J* = 8.4 Hz, 1F).

MS(EI): *m/z* 188 $[M]^+$. The chemical shifts were consistent with those reported in the literature.[2]

1-Fluoro-4-phenoxybenzene (1e)

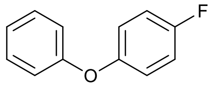

According to **General procedure A**, 4-fluorophenol (560 mg, 5.0 mmol), bromobenzene (1.0 mL, 10.0 mmol), dry DMSO (5.0 mL), and KO^{t}Bu (1.4 g, 12.5 mmol) were used as starting materials. And give title compound **1e** as colorless oil (720 mg, yield: 77 %).

^{1}H NMR (300 MHz, $CDCl_3$) δ 7.39 – 7.29 (m, 2H), 7.14 – 6.91 (m, 7H).

^{19}F NMR (282 MHz, $CDCl_3$) δ -120.63 (td, *J* = 7.7, 3.9 Hz, 1F).

MS(EI): *m/z* 188 $[M]^+$. The chemical shifts were consistent with those reported in the literature.[2]

2-Fluorobenzofuran (1u)

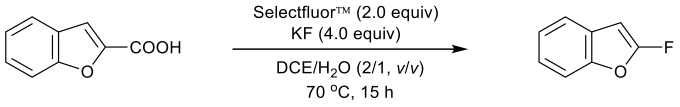

1u: 63%

To a round-bottle reaction flask were added benzofuran-2-carboxylic acid (972 mg, 6.0 mmol), Selectfluor™ (4.25 g, 12.0 mmol), KF (1.40 g, 24.0 mmol). Then dichloroethane (20 mL) and water (10 mL) were added. The reaction mixture was stirred and heat to 70 ºC in an oil bath for 15 hours. The reaction mixture was diluted with water (100 mL), followed by extracting with DCM. The combined organic phases were washed with brine and dried over Na_2SO_4. After filtration, the filtrate was concentrated under reduced pressure to give the residue, which was purified by column chromatography on silica gel (*n*-hexane/EtOAc: 15/1) to give title compound **1u** as colorless oil (513 mg, yield: 63 %).

^{1}H NMR (300 MHz, $CDCl_3$) δ 7.52 – 7.43 (m, 1H), 7.41 – 7.35 (m, 1H), 7.25 – 7.17 (m, 2H), 5.85 (dd, *J* = 6.5, 0.9 Hz, 1H).

^{19}F NMR (282 MHz, $CDCl_3$) δ -112.30 (d, *J* = 6.4 Hz, 1F).

MS(EI): *m/z* 136 $[M]^+$. The chemical shifts were consistent with those reported in the literature.[3]

1-(5-(6-Fluoronaphthalen-2-yl)-2-methoxyphenyl)adamantane (Adapalene derivative, 1ad)

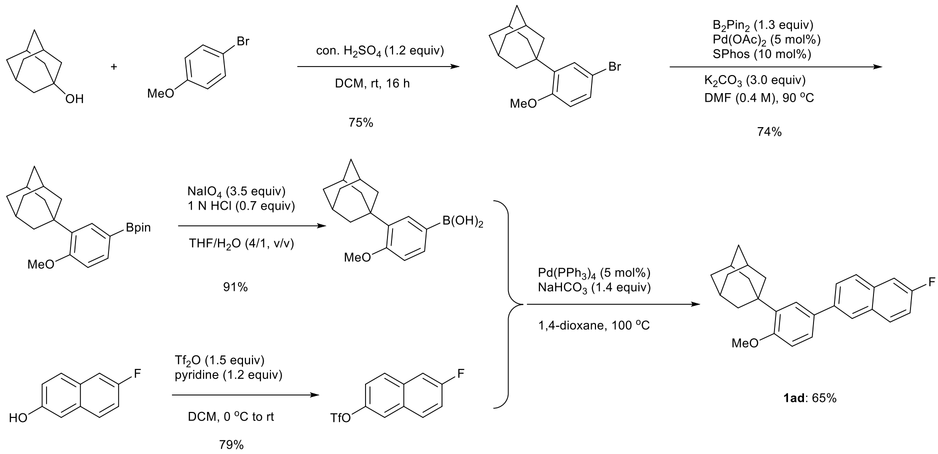

Adapalene derivative (**1ad**) was synthesized according to known procedures with modification.[4]

To a round-bottle reaction flask were added 1-adamantol (3.0 g, 20 mmol), 4-bromoanisole (3.0 mL, 24 mmol), and DCM (15 mL) at room temperature. Then concentrated H_2SO_4 (1.27 mL, 24 mmol) was added dropwise to the previously resulted solution. After the addition finished, keep stirring for 4 h at room temperature, then H_2O (12 mL) was added to the mixture slowly. After stirring for 10 min, the mixture was extracted with EtOAc, washed with water and brine, dried over Na_2SO_4, and filtered through a short pad of silica. The solvent was removed and the solid was recrystallized from *n*-hexane under 0 °C to give aryl bromide as a white solid (4.82 g, yield: 75%).

A flame-dried flask was charged with arylbromide (3.2 g, 10.0 mmol), $Pd(OAc)_2$ (113 mg, 0.5 mmol), S-Phos (410 mg, 1.0 mmol), K_2CO_3 (4.4 g, 30.0 mmol) and B_2Pin_2 (3.3 g, 13.0 mmol), then sealed. After the flask was evacuated and backfilled with N_2 for 3 times, dry DMF was added to the flask. The mixture was allowed to stir for 30 min at room temperature, then elevated to 90 °C for 10 h. After the reaction finished, the resulted mixture was extracted with EtOAc, washed with water and brine, dried over Na_2SO_4, and filtered through a short pad of silica. The solvent was removed and the solid was recrystallized under 0 °C to give aryl boronate as a white solid (2.72 g, yield: 74%).

To a round-bottle reaction flask, previously obtained aryl boronate (2.72 g, 7.4 mmol) and aqueous 1N HCl (5.2 mL, 5.2 mmol) were suspended in a mixed solvent of THF/H_2O (50 mL, 4/1, *v*/*v*), then $NaIO_4$ (4.7 g, 22.2 mmol) was added while stirring. The reaction mixture was kept stirring for 16 h at room temperature, then extracted with EtOAc, washed with brine, dried over Na_2SO_4. After filtered, the filtrate was concentrated under reduced pressure to give the residue, which was purified by recrystallization (EtOAc) to give aryl boronic acid as a white solid (1.92 g, yield: 91 %).

To a round-bottle reaction flask was added 6-fluoronaphthalen-2-ol (0.81 g, 5.0 mmol), pyridine (0.48 mL, 6.0 mmol), and dry DCM (20 mL), then sealed and cooled to 0 °C. followed by the dropwise addition of Tf_2O (1.23 mL,

7.5 mmol), then slowly warm up to room temperature, and keep stirring overnight. The reaction was quenched with saturated $NaHCO_3$, extracted with DCM, washed with brine, dried over Na_2SO_4. After filtered, the filtrate was concentrated under reduced pressure to give the residue, which was purified by column chromatography on silica gel (*n*-hexane/EtOAc: 20/1) to give aryl triflate as a colorless oil (1.16 g, yield: 79 %).

A 30 mL flame-dried flask was charged with aryl boronic acid (686 mg, 2.4 mmol), aryl triflate (380 μL, 2.0 mmol), $Pd(PPh_3)_4$ (120 mg, 0.1 mmol), $NaHCO_3$ (235 mg, 2.8 mmol), then sealed. The flask was evacuated and backfilled with N_2 for 3 times, then degassed 1,4-dioxane (6 mL) was added to the flask. The mixture was allowed to stir at 100 °C overnight. After the reaction finished, the resulted mixture was filtered through a short pad of celite, washed with hot toluene, then the filtrate was extracted with hot toluene and hot water, after dried over Na_2SO_4, filtered, and concentrated under reduced pressure. The obtained residue was recrystallized from hot toluene to give adapalene derivative as a white solid (500 mg, yield: 65%).

^{1}H NMR (300 MHz, $CDCl_3$) δ 7.98 (s, 1H), 7.86 (dd, *J* = 17.2, 7.7 Hz, 2H), 7.75 (d, *J* = 8.2 Hz, 1H), 7.64 – 7.41 (m, 3H), 7.35 – 7.25 (m, 1H), 6.99 (d, *J* = 8.4 Hz, 1H), 3.90 (s, 3H), 2.19 (s, 6H), 2.11 (s, 3H), 1.81 (s, 6H).

^{19}F NMR (282 MHz, $CDCl_3$) δ -115.27 (q, *J* = 8.6 Hz, 1F).

IR (KBr): 2994, 2955, 2903, 2846, 1607, 1497, 1461, 1283, 1239, 1180, 1145, 1061, 1029, 870, 805, 738 cm^{-1}

MS(EI): *m/z* 386 $[M]^+$. The chemical shifts were consistent with those reported in the literature.[4]

2-(4-Ethylpiperazin-1-yl)-4-(4-fluorophenyl)-5,6,7,8,9,10-hexahydrocycloocta[*b*]pyridine (Blonanserin, 1af)

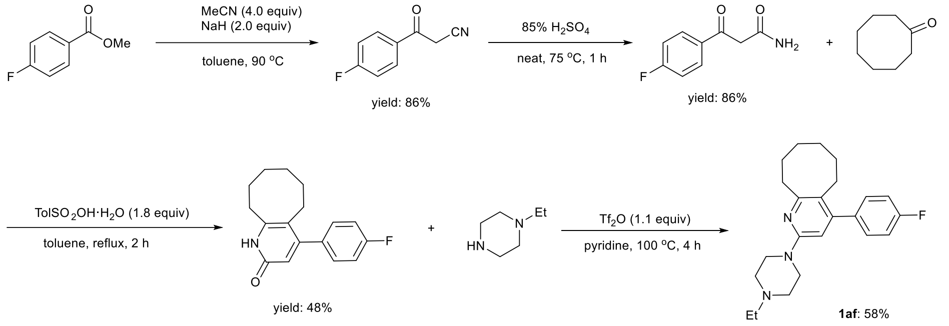

Blonanserin (**1af**) was synthesized according to known procedures with modification.[5]

To a solution of acetonitrile (1.1 mL, 20.0 mmol) in anhydrous toluene (10 mL) was added sodium hydride (0.8 g, 20.0 mmol) at room temperature while stirring, then a solution of methyl 4-fluorobenzoate (1.54 g, 10.0 mmol) in anhydrous toluene (5 mL) was added dropwise. The reaction mixture was then warmed up to 90 °C for 2 h, followed by adding another portion of acetonitrile (1.1 mL, 20.0 mmol). Keep stirring for another 5 h at the same temperature until the reaction fully completed. The mixture was cooled to room temperature and evaporated under vacuum to remove solvent toluene, then water (5 mL) and HCl (3 N) were added to quench the reaction (pH = 5～6). The

reaction mixture was extracted with DCM, and the combined organic phase was washed with brine and dried over Na_2SO_4. After filtration, the filtrate was concentrated under reduced pressure. The residue was purified by recrystallization (*n*-hexane/DCM) two times to give 3-(4-fluorophenyl)-3-oxopropanenitrile as a pale-yellow solid (1.40 g, yield: 86%).

A dried flask was charged with 85% sulfuric acid (15.7 mL, 6 mmol), and 3-(4-fluorophenyl)-3-oxopropanenitrile (1.4 g, 8.59 mmol) was added portion-wise while stirring in an oil bath at 60 °C, then the mixture was stirred at 75 °C for 1 h. The reaction mixture was poured to ice-water and stirred for 10 min until the yellow solid was fully participated. The resulting solid was filtered off and washed with water to afford pale-yellow solid, which was dried *in vacuo* to give product amide (1.34 g, yield: 86%).

A dried flask was charged with 4-methylbenzenesulfonic acid monohydrate (*p*-$TolSO_2OH$ • H_2O, 2.55 g, 13.4 mmol), which dryness by keep stirring under 110 °C for 1 h. Then cooled to 65 °C before a solution of 3-(4-fluorophenyl)-3-oxopropaneamide (1.34 g, 7.4 mmol) and cyclooctanone (0.94 g, 7.4 mmol) in toluene (20 mL) were added, followed by equipped with a Deanstock device. The mixture was stirred at reflux for 2 hours and monitored by TLC until the reaction completed. The reaction was cooled to room temperature evaporated under reduced pressure to remove toluene, followed by DCM and water were added. The reaction mixture was neutered (pH = 7～8) by adding saturated $NaHSO_4$ solution. Stirred for 2 h while maintaining at 10 °C. The resulting solid was filtered and washed with cold toluene and water. The resulting solid was dried *in vacuo* to give pyridin-2(1*H*)-one (0.96 g, yield: 48%) as a white solid.

An oven-dried tube was charged with previously obtained pyridin-2(1*H*)-one (271 mg, 1.0 mmol) and pyridine (725 μL, 9.0 mmol). Then trifluoromethanesulfonic anhydride (185 μL, 1.1 mmol) was added dropwise and keep stirring for 1.5 h at room temperature. Followed by addition of 1-ethylpiperazine (525 μL, 4.0 mmol), and warm up to 100 °C while stirring for 4 h until the reaction completed. To the reaction mixture was added ice water (2 mL), and stirring for 10 min. Then extracted by DCM, washed with brine, dried over anhydrous Na_2SO_4, filtered and concentrated *in vacuo* to give the residue, which was purificated by silica gel column chromatography (DCM/MeOH: 10/1) to afford the title compound, which further recrystallized from *n*-hexane in a refrigerator to give pure Blonanserin (213 mg, yield: 58%) as a white solid.

^{1}H NMR (300 MHz, $CDCl_3$) δ 7.27 – 7.14 (m, 2H), 7.14 – 7.00 (m, 2H), 6.29 (s, 1H), 3.52 (t, *J* = 5.1 Hz, 4H), 2.95 – 2.82 (m, 2H), 2.56 (t, *J* = 5.0 Hz, 6H), 2.46 (q, *J* = 7.2 Hz, 2H), 1.85 – 1.68 (m, 2H), 1.51 – 1.26 (m, 6H), 1.12 (t, *J* = 7.2 Hz, 3H).

^{19}F NMR (282 MHz, $CDCl_3$) δ -115.10 – -115.81 (m, 1F).

MS(EI): *m/z* 386 $[M]^+$. The chemical shifts were consistent with those reported in the literature.[5]

Synthesis of benzyl fluorides 2

Benzyl fluorides **2a**, **2b**, **2c**, **2d**, **2e**, **2f**, **2g**, **2h**, **2i** and **2j** were prepared according to previous methods.[1]

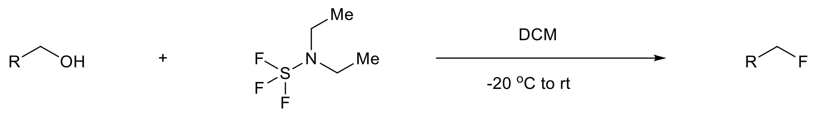

2

General procedure B: To the solution of alcohol (1.0 equiv) in the DCM (0.4 M) was added *N*,*N*-diethylaminosulfur trifluoride (DAST, 1.1 equiv) at –20 °C dropwise. Then the mixture was moved to room temperature and stirred for 6 h. After that, the reaction mixture was poured into cooled water, quenched with saturated $NaHCO_3$ solution, and extracted with DCM. The combined organic phase was dried over Na_2SO_4, filtered, and concentrated under reduced pressure. The crude was purified by column chromatography on silica gel (*n*-hexane) to give alkyl fluorides **2**.

4-(Fluoromethyl)biphenyl (2a)

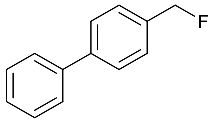

Compound **2a** was synthesized from DAST (1.45 mL, 11.0 mmol) and biphenyl-4-methanol (1.84 g, 10.0 mmol) by using **General procedure B**. And title compound was obtained as a white solid (1.63 g, 88% yield), which packed into a few sample bottles and stored in refrigerator (-20 °C).

^{1}H NMR (300 MHz, $CDCl_3$) δ 7.69 – 7.55 (m, 4H), 7.52 – 7.40 (m, 4H), 7.41 – 7.33 (m, 1H), 5.43 (d, *J* = 47.9 Hz, 2H).

^{19}F NMR (282 MHz, $CDCl_3$) δ -206.63 (t, *J* = 47.8 Hz, 1F).

MS(EI): *m/z* 186 $[M]^+$. The chemical shifts were consistent with those reported in the literature.[1]

1-(Fluoromethyl)-4-(trifluoromethoxy)benzene (2b)

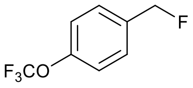

Compound **2b** was synthesized from DAST (0.73 mL, 5.5 mmol) and (4-(trifluoromethyl)phenyl)methanol (0.88 g,

5.0 mmol) by using **General procedure B**. And title compound was obtained as a colorless oil (0.65 g, 67% yield), which stored in refrigerator (-20 °C).

^{1}H NMR (300 MHz, $CDCl_3$) δ 7.46 – 7.34 (m, 2H), 7.29 – 7.15 (m, 2H), 5.38 (d, J = 47.5 Hz, 2H).

^{19}F NMR (282 MHz, $CDCl_3$) δ -58.33 (s, 3F), -208.18 (t, J = 47.6 Hz, 1F).

MS(EI): m/z 194 $[M]^+$. The chemical shifts were consistent with those reported in the literature.[1]

1-Chloro-4-(fluoromethyl)benzene (2c)

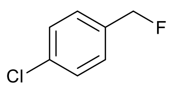

Compound **2c** was synthesized from DAST (0.85 mL, 5.9 mmol) and (4-chlorophenyl)methanol (0.64 g, 4.5 mmol) by using **General procedure B**. And title compound was obtained as a colorless oil (373 mg, 57% yield), which stored in refrigerator (-20 °C).

^{1}H NMR (300 MHz, $CDCl_3$) δ 7.42 – 7.28 (m, 4H), 5.35 (d, J = 47.6 Hz, 2H).

^{19}F NMR (282 MHz, $CDCl_3$) δ -207.84 (t, J = 47.6 Hz, 1F). **MS(EI)**: m/z 144 $[M]^+$.

1-Bromo-4-(fluoromethyl)benzene (2d)

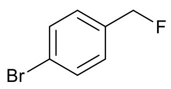

Compound **2d** was synthesized from DAST (0.85 mL, 5.9 mmol) and (4-bromophenyl)methanol (0.84 g, 4.5 mmol) by using **General procedure B**. And title compound was obtained as a white solid (431 mg, 51% yield), which stored in refrigerator (-20 °C).

^{1}H NMR (300 MHz, $CDCl_3$) δ 7.53 (d, J = 8.1 Hz, 2H), 7.25 (d, J = 6.5 Hz, 2H), 5.33 (d, J = 47.6 Hz, 2H).

^{19}F NMR (282 MHz, $CDCl_3$) δ -208.45 (t, J = 47.5 Hz, 1F). **MS(EI)**: m/z 189 $[M]^+$.

1-(*tert*-Butyl)-4-(fluoromethyl)benzene (2e)

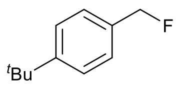

Compound **2e** was synthesized from DAST (0.85 mL, 5.9 mmol) and (4-(*tert*-butyl)phenyl)methanol (0.73 mL, 4.5 mmol) by using **General procedure B**. And title compound was obtained as a colorless oil (386 mg, 52% yield), which stored in refrigerator (-20 °C).

^{1}H NMR (300 MHz, $CDCl_3$) δ 7.44 (d, *J* = 7.5 Hz, 2H), 7.39 – 7.30 (m, 2H), 5.36 (d, *J* = 48.1 Hz, 2H), 1.34 (s, 9H).

^{19}F NMR (282 MHz, $CDCl_3$) δ -204.67 (t, *J* = 48.1 Hz, 1F).

MS(EI): *m/z* 166 $[M]^+$. The chemical shifts were consistent with those reported in the literature.[1]

1-(Fluoromethyl)naphthalene (2g)

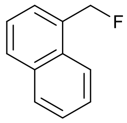

Compound **2g** was synthesized from DAST (0.73 mL, 5.5 mmol) and 1-naphthalenemethanol (0.79 g, 5.0 mmol) by using **General procedure B**. And title compound was obtained as a colorless oil (0.60 g, 75% yield), which stored in refrigerator (-20 °C).

^{1}H NMR (300 MHz, $CDCl_3$) δ 8.17 – 8.01 (m, 1H), 8.02 – 7.80 (m, 2H), 7.68 – 7.37 (m, 4H), 5.87 (d, *J* = 47.9 Hz, 2H).

^{19}F NMR (282 MHz, $CDCl_3$) δ -206.61 (t, *J* = 47.9 Hz, 1F).

MS(EI): *m/z* 160 $[M]^+$. The chemical shifts were consistent with those reported in the literature.[1]

(Cyclohexylfluoromethyl)benzene (2h)

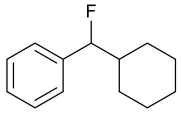

Compound **2h** was synthesized from DAST (0.77 mL, 5.8 mmol) and cyclohexyl(phenyl)methanol (1.0 g, 5.2 mmol) by using **General procedure B**. And title compound was obtained as a colorless oil (0.91 g, 92% yield) , which stored in refrigerator (-20 °C).

^{1}H NMR (300 MHz, $CDCl_3$) δ 7.65 – 7.21 (m, 5H), 5.13 (dd, *J* = 47.2, 7.1 Hz, 1H), 1.98 (d, *J* = 12.4 Hz, 1H), 1.85 – 1.62 (m, 4H), 1.43 (d, *J* = 12.6 Hz, 1H), 1.26 – 0.97 (m, 5H).

^{19}F NMR (282 MHz, $CDCl_3$) δ -180.62 (dd, *J* = 47.2, 16.1 Hz, 1F).

MS(EI): *m/z* 192 $[M]^+$. The chemical shifts were consistent with those reported in the literature.[1]

2-(Fluoromethyl)naphthalene (2f)

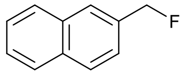

Compound **2f** was synthesized according to known procedure: To a stirred solution of the 2-(bromomethyl)naphthalene (0.89 g, 4.0 mmol) in anhydrous CH_3CN (8 mL) was added tetrabutylammonium fluoride trihydrate (2.52 g, 8.0 mmol). The reaction mixture was refluxed for 24 h. After completion, the reaction

was quenched with water and extracted with EA three times. The combined organic extracts were washed with brine, dried with Na_2SO_4. After filtration and concentration under reduced pressure, the crude product was purified by column chromatography (*n*-hexane) on silica gel to afford pure product **2f** as a white solid (0.56g, 87%), which stored in refrigerator (-20 °C).

^{1}H NMR (700 MHz, $CDCl_3$) δ 7.95 – 7.75 (m, 4H), 7.61 – 7.42 (m, 3H), 5.55 (d, *J* = 47.8 Hz, 2H).

^{19}F NMR (659 MHz, $CDCl_3$) δ -207.27 (t, *J* = 48.0 Hz, 1F).

MS(EI): *m/z* 160 $[M]^+$. The chemical shifts were consistent with those reported in the literature.[1]

General procedure for the synthesis of allenes 3

Allenes **3a-3m**, **3o**, **3t**, **3u**, **3w** were prepared according to known methods. A typical experimental procedure for the preparation of *N*-Methyl anilines were described below (**General procedure C**).

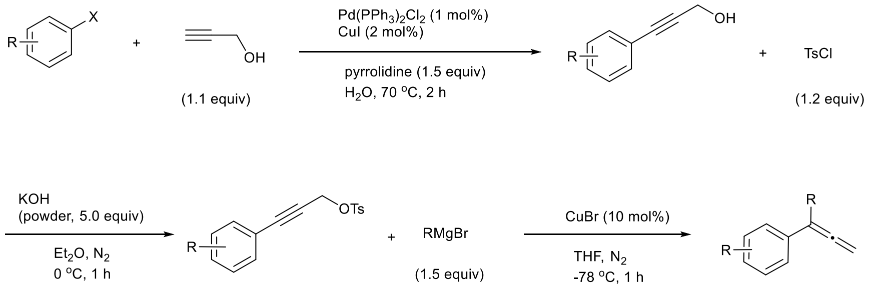

Propargyl alcohols were prepared according to the known procedure with modification.[6] $Pd(PPh_3)_2Cl_2$ (1 mol%), CuI (2 mol%) and pyrrolidine (1.5 equiv) were suspended in H_2O (0.2 M). Then aryl halides (1.0 equiv) and propargyl alcohol (1.1 equiv) was added, the reaction was stirred at 70 °C for 2 h. The mixture was allowed cooled to room temperature after aryl halides fully consumed, then filtered through a short silica pad and extracted with EtOAc, washed with brine, dried over anhydrous Na_2SO_4, filtered and concentrated *in vacuo*. The residue was purified by silica gel column chromatography to afford the corresponding propargyl alcohol.

In a glovebox, powder KOH (5.0 equiv) and propargyl alcohol (1.0 equiv) were added into a flask, sealed, and moved out from glovebox, then dry Et_2O (0.3 M) was injected under 0 °C while stirring. A solution of TsCl (1.2 equiv) in dry Et_2O was added in one portion under 0 °C, and then keep stirring for 1h. After the alcohol fully consumed (by checking TLC), filtered through a short pad of celite, and the filtrate was concentrated under vacuum to give the crude, which purificated by recrystallization yielded the corresponding *O*-tosylates.[7]

In a glovebox, to a dried flask were added *O*-tosylates (1.0 equiv), CuBr (0.1 equiv), and THF (0.4 M), and under argon. Then the resulting mixture was cooled to –78 °C followed by dropwise addition of

Aryl/alkylmagnesium bromide (RMgBr, 1.5 equiv) over 10 min. The resulting mixture was stirred for 1 h at –78 °C and quenched with a saturated aqueous solution of NH_4Cl. After extraction with Et_2O, the organic layer was combined and dried over anhydrous Na_2SO_4. After filtration and concentration under reduced pressure, the crude product was purified by column chromatography on silica gel to afford corresponding allenes.[8]

Penta-1,2-dien-3-ylbenzene (3a)

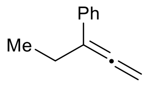

According to **General procedure C**, allene **3a** was finally synthesized from 3-phenylprop-2-yn-1-yl 4-methylbenzenesulfonate (1.91 g, 6.66 mmol), ethylmagnesium bromide (EtMgBr, 10.0 mL, 1.0 M in THF, 10 mmol) and CuBr (95 mg, 0.67 mmol). The obtained residue was purified by column chromatography on silica gel (*n*-hexane) as a light-yellow oil (0.78 g, yield: 81%).

^{1}H NMR (300 MHz, $CDCl_3$) δ 7.45 (d, *J* = 7.3 Hz, 2H), 7.35 (t, *J* = 7.7 Hz, 2H), 7.24 (d, *J* = 8.0 Hz, 1H), 5.14 (t, *J* = 3.6 Hz, 2H), 2.72 – 2.26 (m, 2H), 1.19 (t, *J* = 7.3 Hz, 3H).

MS(EI): *m/z* 144 $[M]^+$. The chemical shifts were consistent with those reported in the literature.[8]

Buta-2,3-dien-2-ylbenzene (3b)

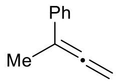

According to **General procedure C**, allene **3b** was finally synthesized from 3-phenylprop-2-yn-1-yl 4-methylbenzenesulfonate (1.15 g, 4.0 mmol), methylmagnesium bromide (MeMgBr, 2.0 mL, 3.0 M in Et_2O, 6 mmol) and CuBr (60 mg, 0.4 mmol). The obtained residue was purified by column chromatography on silica gel (*n*-hexane) as a light-yellow oil (0.39 g, yield: 76%).

^{1}H NMR (300 MHz, $CDCl_3$) δ 7.41 (d, *J* = 6.9 Hz, 2H), 7.32 (t, *J* = 7.9 Hz, 2H), 7.19 (t, *J* = 7.2 Hz, 1H), 5.02 (q, *J* = 3.2 Hz, 2H), 2.10 (t, *J* = 3.1 Hz, 3H).

MS(EI): *m/z* 130 $[M]^+$. The chemical shifts were consistent with those reported in the literature.[9]

Octa-1,2-dien-3-ylbenzene (3c)

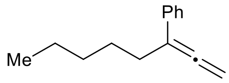

According to **General procedure C**, allene **3c** was finally synthesized from 3-phenylprop-2-yn-1-yl 4-methylbenzenesulfonate (1.44 g, 5.0 mmol), pentylmagnesium bromide (PentylMgBr, freshly prepared from pentyl

bromide, 7.5 mmol) and CuBr (72 mg, 0.5 mmol). The obtained residue was purified by column chromatography on silica gel (*n*-hexane) as a light-yellow oil (0.84 g, yield: 90%).

^{1}H NMR (300 MHz, $CDCl_3$) δ 7.44 (d, J = 7.3 Hz, 2H), 7.35 (t, J = 7.6 Hz, 2H), 7.22 (t, J = 7.3 Hz, 1H), 5.09 (t, J = 3.3 Hz, 2H), 2.44 (tt, J = 7.1, 3.3 Hz, 2H), 1.58 (q, J = 7.7, 7.1 Hz, 2H), 1.39 (q, J = 3.7 Hz, 4H), 0.95 (t, J = 6.8 Hz, 3H).

MS(EI): *m/z* 186 $[M]^+$. The chemical shifts were consistent with those reported in the literature.[10]

(5-Methylhexa-1,2-dien-3-yl)benzene (3d)

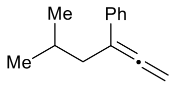

According to **General procedure C**, allene **3d** was finally synthesized from 3-phenylprop-2-yn-1-yl 4-methylbenzenesulfonate (1.44 g, 5.0 mmol), isobutylmagnesium bromide (freshly prepared from pentyl bromide, 10.0 mmol) and CuBr (72 mg, 0.5 mmol). The obtained residue was purified by column chromatography on silica gel (*n*-hexane) as a light-yellow oil (0.82 g, yield: 95%).

^{1}H NMR (300 MHz, $CDCl_3$) δ 7.48 – 7.39 (m, 2H), 7.38 – 7.30 (m, 2H), 7.22 (t, J = 7.2 Hz, 1H), 5.07 (t, J = 2.8 Hz, 2H), 2.34 (dt, J = 6.9, 2.8 Hz, 2H), 1.90 (dp, J = 13.4, 6.7 Hz, 1H), 1.01 (s, 3H), 0.99 (s, 3H).

MS(EI): *m/z* 172 $[M]^+$. The chemical shifts were consistent with those reported in the literature.[11]

(4-Methylpenta-1,2-dien-3-yl)benzene (3e)

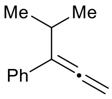

According to **General procedure C**, allene **3e** was finally synthesized from 3-phenylprop-2-yn-1-yl 4-methylbenzenesulfonate (1.44 g, 5.0 mmol), isopropylmagnesium bromide (^{i}PrMgBr, 15.0 mL, 0.5 M, 7.5 mmol) and CuBr (72 mg, 0.5 mmol). The obtained residue was purified by column chromatography on silica gel (*n*-hexane) as a light-yellow oil (0.62 g, yield: 81%).

^{1}H NMR (300 MHz, $CDCl_3$) δ 7.44 – 7.37 (m, 2H), 7.35 – 7.27 (m, 2H), 7.23 – 7.15 (m, 1H), 5.08 (d, J = 2.8 Hz, 2H), 2.90 – 2.72 (m, 1H), 1.15 (s, 3H), 1.13 (s, 3H).

MS(EI): *m/z* 158 $[M]^+$. The chemical shifts were consistent with those reported in the literature.[10]

1-(Penta-1,2-dien-3-yl)-2-(trifluoromethoxy)benzene (3f)

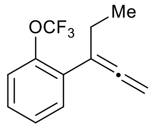

According to **General procedure C**, allene **3f** was finally synthesized from 3-(2-(trifluoromethoxy)phenyl)prop-2-yn-1-yl 4-methylbenzenesulfonate (1.85 g, 5.0 mmol), EtMgBr (10.0 mL, 1.0 M, 10.0 mmol) and CuBr (72 mg, 0.5 mmol). The obtained residue was purified by column chromatography on silica gel (*n*-hexane) as a colorless oil (0.76 g, yield: 67%).

^{1}H NMR (500 MHz, $CDCl_3$) δ 7.38 – 7.28 (m, 1H), 7.29 – 7.14 (m, 3H), 4.92 (t, *J* = 3.6 Hz, 2H), 2.38 (qt, *J* = 7.3, 3.6 Hz, 2H), 1.07 (t, *J* = 7.4 Hz, 3H).

^{13}C NMR (126 MHz, $CDCl_3$) δ 208.4, 146.7, 131.8, 130.1, 128.2, 126.9, 121.4, 120.7 (q, *J* = 257.6 Hz), 102.4, 76.6, 25.2, 12.5.

^{19}F NMR (282 MHz, $CDCl_3$) δ -57.48 (s, 3F).

IR (KBr): 2972, 2939, 2875, 1951, 1491, 1450, 1259, 1222, 1167, 1072, 933, 849, 763, 631 cm^{-1}

HRMS (EI) [$C_{12}H_{11}F_3O$] $[M]^+$ calculated: 228.0762, found: 228.0765.

1-Methoxy-4-(penta-1,2-dien-3-yl)benzene (3g)

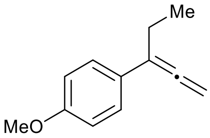

According to **General procedure C**, allene **3g** was finally synthesized from 3-(4-methoxyphenyl)prop-2-yn-1-yl 4-methylbenzenesulfonate (2.50 g, 7.86 mmol), EtMgBr (15.7 mL, 1 M in THF, 15.7 mmol,) and CuBr (112 mg, 0.79 mmol). The obtained residue was purified by column chromatography on silica gel (*n*-hexane/DCM: 10:1) as a light-yellow oil (1.08 g, yield: 78%).

^{1}H NMR (300 MHz, $CDCl_3$) δ 7.34 (d, *J* = 8.9 Hz, 2H), 6.88 (d, *J* = 8.9 Hz, 2H), 5.09 (t, *J* = 3.5 Hz, 2H), 3.81 (s, 3H), 2.41 (tt, *J* = 7.2, 3.7 Hz, 2H), 1.15 (t, *J* = 7.3 Hz, 3H).

MS(EI): *m/z* 174 $[M]^+$. The chemical shifts were consistent with those reported in the literature.[9]

1-Methoxy-3-(penta-1,2-dien-3-yl)benzene (3h)

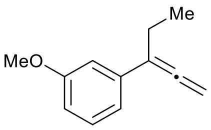

According to **General procedure C**, allene **3h** was finally synthesized from 3-(3-methoxyphenyl)prop-2-yn-1-yl 4-methylbenzenesulfonate (2.1 g, 6.63 mmol), EtMgBr (8.0 mL, 1 M in THF, 8.0 mmol,) and CuBr (95 mg, 0.663 mmol). The obtained residue was purified by column chromatography on silica gel (*n*-hexane/DCM: 10/1) as a light-yellow oil (866 mg, yield: 75%).

^{1}H NMR (300 MHz, $CDCl_3$) δ 7.25 (t, *J* = 7.9 Hz, 1H), 7.07 – 6.88 (m, 2H), 6.77 (dd, *J* = 8.2, 2.6 Hz, 1H), 5.12 (t, *J* = 3.7 Hz, 2H), 3.82 (s, 3H), 2.43 (tt, *J* = 7.3, 3.6 Hz, 2H), 1.17 (t, *J* = 7.3 Hz, 3H).

^{13}C NMR (75 MHz, $CDCl_3$) δ 208.6, 159.8, 138.3, 129.4, 118.6, 112.1, 111.9, 106.8, 78.9, 55.3, 22.6, 12.6.

IR (KBr): 3050, 2998, 2968, 2934, 2834, 1940, 1599, 1579, 1487, 1455, 1284, 1200, 1168, 1051, 852, 778, 697, 633 cm^{-1}

HRMS (EI) [$C_{12}H_{14}O$] $[M]^+$ calculated: 174.1045, found: 174.1047.

1-Methyl-4-(penta-1,2-dien-3-yl)benzene (3i)

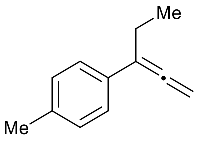

According to **General procedure C**, allene **3i** was finally synthesized from 3-(*p*-tolyl)prop-2-yn-1-yl 4-methylbenzenesulfonate (3.02 g, 10.0 mmol), EtMgBr (20.0 mL, 1.0 M in THF, 20 mmol) and CuBr (144 mg, 1.0 mmol). The obtained residue was purified by column chromatography on silica gel (*n*-pentane) as a light-yellow oil (1.73 g, yield: 80%).

^{1}H NMR (300 MHz, $CDCl_3$) δ 7.32 (d, *J* = 8.2 Hz, 2H), 7.15 (d, *J* = 8.4 Hz, 2H), 5.10 (t, *J* = 3.7 Hz, 2H), 2.43 (qt, *J* = 7.3, 3.6 Hz, 2H), 2.35 (s, 3H), 1.23 – 1.08 (m, 3H).

MS(EI): *m/z* 158 $[M]^+$. The chemical shifts were consistent with those reported in the literature.[9]

1-Methyl-3-(penta-1,2-dien-3-yl)benzene (3j)

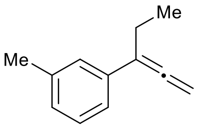

According to **General procedure C**, allene **3j** was finally synthesized from 3-(*m*-tolyl)prop-2-yn-1-yl 4-methylbenzenesulfonate (1.944 g, 6.47 mmol), EtMgBr (9.7 mL, 1 M in THF, 9.7 mmol) and CuBr (93 mg, 0.5

mmol). The obtained residue was purified by column chromatography on silica gel (*n*-hexane) as a light-yellow oil (0.63 g, yield: 61%).

^{1}H NMR (300 MHz, $CDCl_3$) δ 7.30 – 7.14 (m, 3H), 7.07 – 6.94 (m, 1H), 5.08 (t, J = 3.7 Hz, 2H), 2.41 (qt, J = 7.3, 3.7 Hz, 2H), 2.34 (s, 3H), 1.14 (t, J = 7.3 Hz, 3H).

^{13}C NMR (75 MHz, $CDCl_3$) δ 208.5, 138.0, 136.6, 128.4, 127.5, 126.8, 123.1, 106.8, 78.7, 22.6, 21.6, 12.6.

IR (KBr): 3038, 2969, 2933, 2914, 2875, 1940, 1604, 1488, 1457, 1378, 1267, 1077, 849, 780, 700, 636 cm^{-1}

HRMS (EI) [$C_{12}H_{14}$] [M]$^+$ calculated: 158.1096, found: 158.1099.

1,3-Dimethyl-5-(penta-1,2-dien-3-yl)benzene (3k)

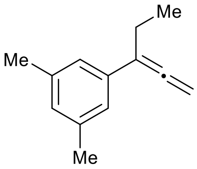

According to **General procedure C**, allene **3k** was finally synthesized from 3-(3,5-dimethylphenyl)prop-2-yn-1-yl 4-methylbenzenesulfonate (2.73 g, 9.08 mmol), EtMgBr (13.6 mL, 1 M in THF, 13.6 mmol) and CuBr (130 mg, 0.91 mmol). The obtained residue was purified by column chromatography on silica gel (*n*-hexane) as a light-yellow oil (1.04 g, yield: 66%).

^{1}H NMR (300 MHz, $CDCl_3$) δ 7.03 (s, 2H), 6.85 (s, 1H), 5.07 (d, J = 3.8 Hz, 2H), 2.41 (dt, J = 7.3, 3.7 Hz, 2H), 2.31 (s, 6H), 1.14 (t, J = 7.4 Hz, 3H).

MS(EI): *m/z* 172 [M]$^+$. The chemical shifts were consistent with those reported in the literature.[12]

1-Chloro-4-(penta-1,2-dien-3-yl)benzene (3l)

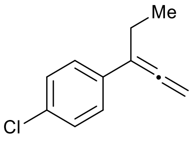

According to **General procedure C**, allene **3l** was finally synthesized from 3-(4-chlorophenyl)prop-2-yn-1-yl 4-methylbenzenesulfonate (2.44 g, 7.61 mmol), EtMgBr (11.4 mL, 1 M in THF, 11.4 mmol) and CuBr (109 mg, 0.76 mmol). The obtained residue was purified by column chromatography on silica gel (*n*-hexane) as a light-yellow oil (0.86 g, yield: 63%).

^{1}H NMR (300 MHz, $CDCl_3$) δ 7.46 – 7.13 (m, 4H), 5.11 (t, J = 3.6 Hz, 2H), 2.47 – 2.30 (m, 2H), 1.14 (t, J = 7.3 Hz, 3H).

MS(EI): *m/z* 178 [M]$^+$. The chemical shifts were consistent with those reported in the literature.[9]

2-(Penta-1,2-dien-3-yl)naphthalene (3m)

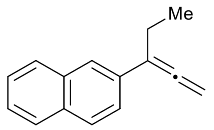

According to **General procedure C**, allene **3m** was finally synthesized from 3-(naphthalen-2-yl)prop-2-yn-1-yl 4-methylbenzenesulfonate (1.83 g, 5.4 mmol), EtMgBr (8.1 mL, 1.0 M in THF, 8.1 mmol) and CuBr (72 mg, 0.5 mmol). The obtained residue was purified by column chromatography on silica gel (*n*-hexane) as a white solid (0.90 g, yield: 86%).

^{1}H NMR (300 MHz, $CDCl_3$) δ 7.88 – 7.72 (m, 4H), 7.71 – 7.58 (m, 1H), 7.54 – 7.35 (m, 2H), 5.21 (t, *J* = 3.6 Hz, 2H), 2.58 (qt, *J* = 7.3, 3.6 Hz, 2H), 1.24 (t, *J* = 7.3 Hz, 3H).

MS(EI): *m/z* 194 $[M]^+$. The chemical shifts were consistent with those reported in the literature.[9]

3-Ethylhepta-1,2-diene (3w)

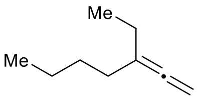

According to **General procedure C**, allene **3w** was finally synthesized from hept-2-yn-1-yl 4-methylbenzenesulfonate (1.33 g, 5.0 mmol), EtMgBr (7.5 mL, 1.0 M in THF, 7.5 mmol) and CuBr (72 mg, 0.5 mmol). The obtained residue was purified by column chromatography on silica gel (*n*-hexane) as a light-yellow oil (0.46 g, yield: 74%).

^{1}H NMR (300 MHz, $CDCl_3$) δ 4.72 – 4.60 (m, 2H), 2.01 – 1.86 (m, 4H), 1.50 – 1.20 (m, 4H), 1.01 (td, *J* = 7.4, 0.9 Hz, 3H), 0.90 (t, *J* = 6.9 Hz, 3H).

MS(EI): *m/z* 124 $[M]^+$. The chemical shifts were consistent with those reported in the literature.[13]

(1-Cyclopropylpropa-1,2-dien-1-yl)benzene (3o)

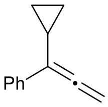

According to **General procedure C**, allene **3o** was finally synthesized from 3-phenylprop-2-yn-1-yl 4-methylbenzenesulfonate (0.86 g, 3.0 mmol), cyclopropylmagnesium bromide (freshly prepared, 4.5 mmol) and CuBr (43 mg, 0.3 mmol). The obtained residue was purified by column chromatography on silica gel (*n*-pentane) as a colorless oil (115 mg, yield: 24%).

^{1}H NMR (300 MHz, $CDCl_3$) δ 7.67 – 7.57 (m, 2H), 7.41 – 7.32 (m, 2H), 7.29 – 7.20 (m, 1H), 5.12 (d, *J* = 3.0 Hz, 2H), 1.65 – 1.54 (m, 1H), 0.97 – 0.86 (m, 2H), 0.65 – 0.53 (m, 2H).

MS(EI): *m/z* 156 $[M]^+$. The chemical shifts were consistent with those reported in the literature.[14]

Propa-1,2-diene-1,1-diyldibenzene (3t)

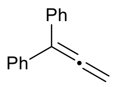

According to **General procedure C**, allene **3t** was finally synthesized from 3-phenylprop-2-yn-1-yl 4-methylbenzenesulfonate (1.15 g, 4.0 mmol), phenylmagnesium bromide (PhMgBr, 6.0 mL, 1.0 M, 6.0 mmol) and CuBr (60 mg, 0.4 mmol). The obtained residue was purified by column chromatography on silica gel (*n*-hexane) as a light-yellow oil (0.62 g, yield: 81%).

^{1}H NMR (300 MHz, $CDCl_3$) δ 7.64 – 7.53 (m, 4H), 7.49 – 7.38 (m, 4H), 7.35 – 7.28 (m, 2H), 5.26 (s, 2H).

MS(EI): *m/z* 192 $[M]^+$. The chemical shifts were consistent with those reported in the literature.[10]

Penta-1,2,4-trien-3-ylbenzene (3u)

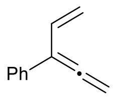

According to **General procedure C**, allene **3u** was finally synthesized from 3-phenylprop-2-yn-1-yl 4-methylbenzenesulfonate (1.44 g, 5.0 mmol), vinylmagnesium bromide (CH_2=CHMgBr, 7.5 mL, 1.0 M, 7.5 mmol) and CuBr (72 mg, 0.5 mmol). The obtained residue was purified by column chromatography on silica gel (*n*-hexane) as a light-yellow oil (0.52 g, yield: 73%).

^{1}H NMR (300 MHz, $CDCl_3$) δ 7.50 – 7.36 (m, 2H), 7.36 – 7.17 (m, 3H), 6.10 – 5.74 (m, 1H), 5.49 – 5.34 (m, 1H), 5.25 – 5.09 (m, 1H), 3.33 – 2.98 (m, 2H).

MS(EI): *m/z* 142 $[M]^+$.

Synthesis of substituted allenes

γ-Position substituted allenes **3p-3s** were prepared according to known methods.[15] A typical experimental procedure for the preparation of γ-Position substituted allenes were described below (**General procedure D**).

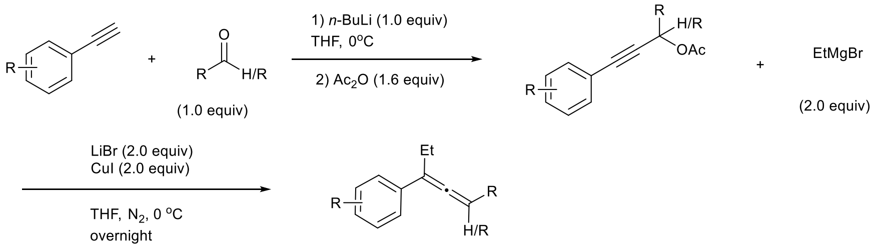

To a solution of aryl acetylene (1.0 equiv) in THF (1.0 M) was added *n*-BuLi (1.6 M in *n*-hexane, 1.0 equiv) at

0 °C under N_2 atmosphere, and the mixture was stirred at 0 °C for 30 min before it was treated with aldehyde or ketone (1.0 equiv). The mixture was stirred for 30 min at room temperature and then acetic anhydride (1.6 equiv) was added to the flask at 0 °C, and the mixture was stirred for another 2 h at room temperature before adding water. The aqueous solution was extracted with EtOAc. The combined organic layers were dried over anhydrous Na_2SO_4, filtered, and concentrated *in vacuo*. The residue was purified by silica gel column chromatography to afford the corresponding propargyl acetates.

To a 50 mL oven-dried flask were added CuI (2.0 equiv), LiBr (2.0 equiv), THF (0.25 M), and then EtMgBr (1.0 M in THF, 2.0 equiv) under N_2 atmosphere at 0 °C. The mixture was stirred for 30 min at room temperature and then propargyl acetate was added at 0 °C. The resulting mixture was stirred overnight at room temperature, and then quenched by saturated NH_4Cl solution. The mixture was filtered to remove the solid. The filtrate was extracted with Et_2O. The combined organic layers were dried over anhydrous Na_2SO_4, filtered, and concentrated *in vacuo*. The residue was purified by silica gel column chromatography to afford the final product.

Dodeca-3,4-dien-3-ylbenzene (3p)

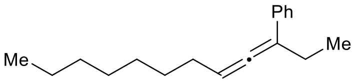

According to **General procedure D**, allene **3p** was finally synthesized from 1-phenyldec-1-yn-3-yl acetate (1.5 g, 5.5 mmol), EtMgBr (11 mL, 11.0 mmol), CuBr•DMS (2.27 g, 11.0 mmol) and LiBr (0.96 g, 11 mmol). The obtained residue was purified by column chromatography on silica gel (*n*-hexane) as a light-yellow oil (1.05 g, yield: 79%).

^{1}H NMR (300 MHz, $CDCl_3$) δ 7.46 – 7.38 (m, 2H), 7.36 – 7.27 (m, 2H), 7.23 – 7.13 (m, 1H), 5.55 (tt, *J* = 6.7, 3.3 Hz, 1H), 2.44 (qdd, *J* = 7.3, 3.4, 1.4 Hz, 2H), 2.13 (q, *J* = 7.0 Hz, 2H), 1.48 (q, *J* = 7.4 Hz, 2H), 1.41 – 1.22 (m, 8H), 1.15 (t, *J* = 7.3 Hz, 3H), 0.97 – 0.77 (m, 3H).

^{13}C NMR (75 MHz, $CDCl_3$) δ 203.6, 137.8, 128.4, 126.4, 125.9, 107.4, 95.4, 32.0, 29.5, 29.4, 29.3, 23.0, 22.8, 14.3, 12.8.

HRMS (EI) [$C_{18}H_{26}$] $[M]^+$ calculated: 242.2035, found: 242.2037.

Octa-3,4-dien-3-ylbenzene (3q)

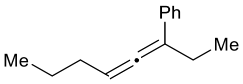

According to **General procedure D**, allene **3q** was finally synthesized from 1-phenylhex-1-yn-3-yl acetate (1.08 g, 5.0 mmol), EtMgBr (10 mL, 10.0 mmol), CuI (1.90 g, 10.0 mmol) and LiBr (0.87 g, 10 mmol). The obtained residue was purified by column chromatography on silica gel (*n*-hexane) as a light-yellow oil (0.68 g, yield: 73%).

^{1}H NMR (300 MHz, $CDCl_3$) δ 7.41 (d, *J* = 7.8 Hz, 2H), 7.30 (t, *J* = 7.5 Hz, 2H), 7.17 (t, *J* = 7.3 Hz, 1H), 5.54 (dd, *J* = 6.5, 3.3 Hz, 1H), 2.53 – 2.26 (m, 2H), 2.09 (q, *J* = 6.6 Hz, 2H), 1.48 (p, *J* = 7.5 Hz, 2H), 1.13 (t, *J* = 7.3 Hz, 3H), 0.96 (t, *J* = 7.2 Hz, 3H).

MS(EI): *m/z* 186 $[M]^+$. The chemical shifts were consistent with those reported in the literature.[16]

Penta-1,2-diene-1,3-diyldibenzene (3r)

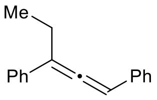

According to **General procedure D**, allene **3r** was finally synthesized from 1,3-diphenylprop-2-yn-1-yl acetate (2.18 g, 8.7 mmol), EtMgBr (17.4 mL, 17.4 mmol), CuI (1.51 g, 17.4 mmol) and LiBr (3.31 g, 17.4 mmol). The obtained residue was purified by column chromatography on silica gel (*n*-hexane) as a light-yellow oil (1.23 g, yield: 64%).

^{1}H NMR (300 MHz, $CDCl_3$) δ 7.56 – 7.46 (m, 2H), 7.46 – 7.30 (m, 6H), 7.30 – 7.20 (m, 2H), 6.62 (t, *J* = 3.5 Hz, 1H), 2.84 – 2.45 (m, 2H), 1.26 (t, *J* = 7.3 Hz, 3H).

MS(EI): *m/z* 220 $[M]^+$. The chemical shifts were consistent with those reported in the literature.[15]

(5-Methylhexa-3,4-dien-3-yl)benzene (3s)

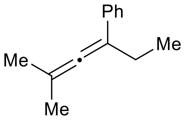

According to **General procedure D**, allene **3s** was finally synthesized from 2-methyl-4-phenylbut-3-yn-2-yl acetate (1.01 g, 5.0 mmol), EtMgBr (10 mL, 10.0 mmol), CuI (1.90 g, 10.0 mmol) and LiBr (0.87 g, 10 mmol). The obtained residue was purified by column chromatography on silica gel (*n*-hexane) as a light-yellow oil (0.73 g, yield: 85%).

^{1}H NMR (300 MHz, $CDCl_3$) δ 7.37 (d, *J* = 8.4 Hz, 2H), 7.31 – 7.22 (m, 2H), 7.20 – 7.08 (m, 1H), 2.39 (q, *J* = 7.2 Hz, 2H), 1.80 (d, *J* = 1.0 Hz, 6H), 1.10 (t, *J* = 7.3 Hz, 3H).

MS(EI): *m/z* 172 $[M]^+$. The chemical shifts were consistent with those reported in the literature.[16]

Synthesis of triethyl(3-phenylpenta-1,2-dien-1-yl)silane (3n)

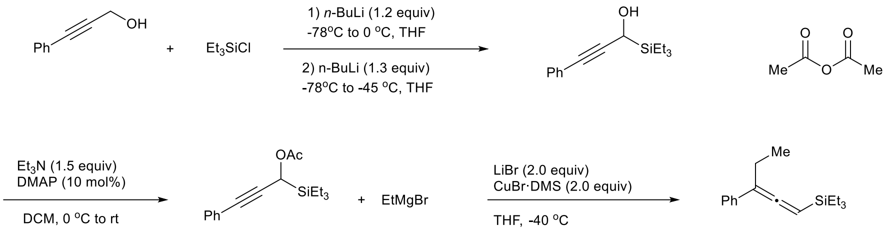

Propargyl alcohol were prepared according to the known procedure.[17]

To a solution of 3-phenylprop-2-yn-1-ol (1.25 mL, 10 mmol) in THF (10.0 mL) was added *n*-BuLi (7.5 mL, 12 mmol, 1.6 M solution in *n*-hexane) at –78 °C and the mixture was stirred at 0 °C for 30 minutes. To the solution was added a solution of Et_3SiCl (1.7 mL, 11 mmol) in THF (11.0 mL) at –78 °C. After stirring at rt for 4 h, *n*-BuLi (8.2 mL, 13 mmol, 1.6 M solution in hexanes) was added dropwise to the solution at –78 °C and the mixture stirred at –45 °C for 2 h. The reaction was quenched by 10% AcOH in THF at –78 °C. The mixture was extracted with Et_2O, and the organic layer was washed with saturated $NaHCO_3$ and brine, dried over Na_2SO_4, and concentrated *in vacuo*. The residue was purified by flash column chromatography on silica gel (*n*-hexane/EtOAc: 5/1) to give the propargyl alcohol as a colorless oil (1.75 g, yield: 71%).

According to a known procedure.[18] To a solution of propargyl alcohol (0.5 g, 2.0 mmol) in CH_2Cl_2 (20 mL) was added Et_3N (0.42 mL, 3.0 mmol), acetic anhydride (284 μL, 3.0 mmol), and DMAP (25 mg, 0.2 mmol) at 0 °C under an argon atmosphere. After the reaction mixture was stirred at room temperature until TLC indicated fully consumption of the starting material, the mixture was quenched by saturated NH_4Cl. The mixture was extracted with EtOAc, and organic layer was washed with brine, dried over Na_2SO_4, and concentrated *in vacuo*. The residue was purified by flash column chromatography on silica gel (*n*-hexane/EtOAc: 20/1) to give the propargyl acetate as a colorless oil (0. 57 g, yield: 99%).

To a 50 mL oven-dried flask were added LiBr (0.35 g, 4.0 mmol), CuBr•DMS (0.82 g, 4.0 mmol), THF (8 mL) and stirred at –40 °C under N_2 atmosphere, then EtMgBr (4.0 mL, 4.0 mmol) was added dropwise. The mixture was stirred for 40 min and then propargyl acetate was added dropwise before the mixture slowly warm-up to 0 °C. The resulting mixture was kept stirred for 1 h, and then quenched by saturated NH_4Cl solution. The mixture was extracted with Et_2O, dried over anhydrous Na_2SO_4, filtered, and concentrated *in vacuo*. The residue was purified by silica gel column chromatography (*n*-hexane) to afford the title product as a light-yellow oil (0.49 g, yield: 94%).

^{1}H NMR (300 MHz, $CDCl_3$) δ 7.47 – 7.21 (m, 4H), 7.15 (t, *J* = 7.1 Hz, 1H), 5.38 (t, *J* = 4.2 Hz, 1H), 2.41 (tq, *J* = 7.6, 3.9 Hz, 2H), 1.17 (t, *J* = 7.2 Hz, 3H), 1.07 – 0.88 (t, *J* = 7.6 Hz, 9H), 0.65 (q, *J* = 7.6 Hz, 6H).

^{13}**C NMR** (176 MHz, $CDCl_3$) δ 210.7, 137.5, 128.4, 125.7, 125.4, 100.1, 83.8, 22.0, 12.9, 7.5, 4.2.

IR (KBr): 3060, 2954, 2910, 2874, 1922, 1597, 1494, 1456, 1353, 1260, 1237, 1074, 1015, 773, 741, 692, 635 cm^{-1}

HRMS (EI) [$C_{15}H_{21}Si$] $[M-Et]^+$ calculated: 229.1413, found: 229.1416.

1-Methyl-4-(propa-1,2-dien-1-yl)benzene (3v)

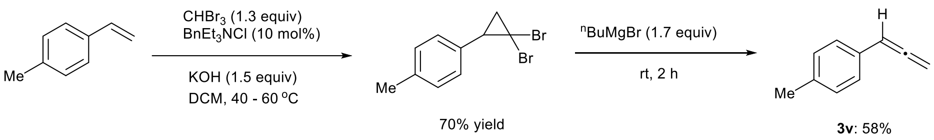

Compound **3v** was prepared according to a known procedure.[19]

In an N_2-filled glovebox, to a 50 mL oven-dried flask were added potassium hydroxide (0.84 g, 15.0 mmol), DCM (5 mL), 4-methylstyrene (1.18 g, 10.0 mmol) and benzyltriethylammonium bromide (228 mg, 1.0 mmol). Then the flask was sealed and move out from glovebox, keep stirring at 40 °C in an oil-bath. A solution of $CHBr_3$ (3.3 g in 2.0 mL of DCM) was added dropwise to the mixture (The reaction mixture was kept below 50 °C). Then the reaction mixture was stirred for 20 hours at 60 °C. The resulting mixture was filtered through a short pad of silica, and concentrated *in vacuo*, the crude product was purified by a short flash silica gel column chromatography (*n*-hexane) to give 1-(2,2-dibromocyclopropyl)-4-methylbenzene as a yellow oil (2.03 g, 70% yield).

To a dried flask were added 1-(2,2-dibromocyclopropyl)-4-methylbenzene (1.73 g, 6.0 mmol), and anhydrous THF (12 mL) under argon atmosphere. Then the resulting mixture was followed by dropwise addition of butylmagnesium bromide (*n*-BuMgBr, 0.5 M, freshly prepared from 10.0 mmol butyl bromide) over 10 min. The resulting mixture was stirred for 2 h at room temperature and quenched with Et_2O (6 mL), H_2O (2 mL) and a solution of HCl (5 mL, 3N). After extraction with Et_2O, washed with brine, dried over anhydrous Na_2SO_4. After filtration and concentrated *in vacuo*, the crude product was purified by column chromatography on silica gel (*n*-hexane) to afford desired allene **3w** as a colorless oil (450 mg, 58% yield).

^{1}**H NMR** (300 MHz, $CDCl_3$) δ 7.22 (d, J = 7.9 Hz, 2H), 7.14 (d, J = 7.8 Hz, 2H), 6.17 (t, J = 6.8 Hz, 1H), 5.16 (d, J = 6.8 Hz, 2H), 2.36 (s, 3H).

MS(EI): m/z 130 $[M]^+$. The chemical shifts were consistent with those reported in the literature.[19]

Synthesis of deuterated allenes

1-Methoxy-4-(penta-1,2-dien-3-yl-1,1-d^2)benzene (d^2-3g)

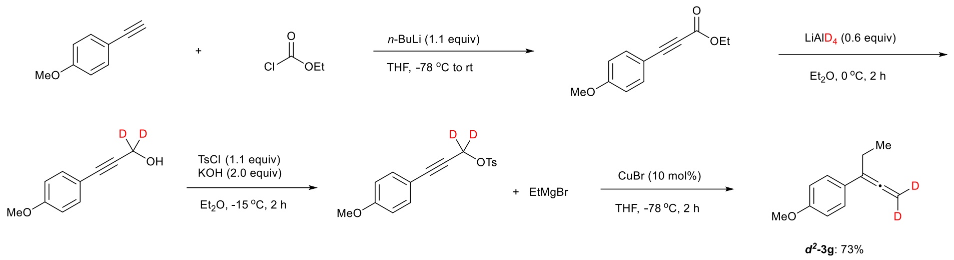

To a 100 mL oven-dried flask were added 4-ethynylanisole (1.0 g, 7.5 mmol) and anhydrous THF (10 mL), then stirred under –78 °C. *n*-BuLi (5.2 mL, 1.59 M in *n*-hexane, 8.3 mmol) was then added dropwise. The solution was stirred at –78 °C for 1 h, followed by adding a solution of ethyl chloroformate (0.8 mL, 8.3 mmol) in anhydrous THF (5 mL) dropwise. The mixture was allowed stirred further 1 h and then slowly warm-up to room temperature. After stirring overnight at room temperature, the reaction mixture was quenched by saturated NH_4Cl solution, and extracted with EtOAc, washed with brine, dried over anhydrous Na_2SO_4, filtered and concentrated *in vacuo*. The residue was purified by silica gel column chromatography (*n*-hexane/EtOAc: 10/1) to afford ethyl 3-(4-methoxyphenyl)propiolate as a light-yellow solid (1.19 g, yield: 78%).[20]

A solution of ethyl 3-(4-methoxyphenyl)propiolate (1.1 g, 5.4 mmol) in dry Et_2O (5 mL) was added slowly to a stirred suspension of lithium aluminum deuteride ($LiAlD_4$, 147 mg, 3.5 mmol) in Et_2O (5 mL) at 0 °C under argon. The resulting mixture was stirred for further 2h, followed by addition of EtOAc (5 mL) and hydrochloric acid (2 M, 15 mL). The mixture was extracted with EtOAc, washed with brine, dried over anhydrous Na_2SO_4, filtered and concentrated *in vacuo*. The residue was purified by silica gel column chromatography (*n*-hexane/EtOAc: 8/1) to afford di-deuterated propargyl alcohol as a light-yellow solid (0.7 g, yield: 73%).[21]

A solution of di-deuterated propargyl alcohol (0.7 g, 4.2 mmol) and TsCl (0.9 g, 4.7 mmol) in dry Et_2O (10 mL) was stirred under –15 °C. Powder KOH (0.96 g, 4.0 equiv) was added portion-wise over 30 min, and then keep stirring for 2 h. After the alcohol fully consumed (by checking TLC), filtered through a short pad of celite, and the filtrate was concentrated under vacuum to give the crude, which purificated by recrystallization (Et_2O/*n*-hexane) under –18 °C to afford di-deuterated *O*-tosylate as a white solid (1.0 g, yield: 75%).

In a glovebox, to a dried flask were added previously obtained di-deuterated *O*-tosylates (1.02 g, 3.2 mmol), CuBr (46 mg, 0.32 mmol), and THF (10 mL), and under argon. Then the resulting mixture was cooled to –78 °C followed by dropwise addition of EtMgBr (6.0 mL, 1 M in THF, 6.0 mmol) over 10 min. The resulting mixture was stirred for 1 h at –78 °C and quenched with a saturated aqueous solution of NH_4Cl (5 mL). The mixture was then

extracted with Et_2O, washed with brine, dried over anhydrous Na_2SO_4, filtered, and concentrated *in vacuo*. The residue was purified by silica gel column chromatography (*n*-hexane/DCM: 10/1) to afford titled di-deuterated allene (0.41 g, yield: 73%).

^{1}H NMR (300 MHz, $CDCl_3$) δ 7.39 – 7.27 (m, 2H), 6.96 – 6.77 (m, 2H), 3.78 (s, 3H), 2.39 (q, J = 7.3 Hz, 2H), 1.14 (t, J = 7.3 Hz, 3H).

MS(EI): *m/z* 176 $[M]^+$.

(Penta-1,2-dien-3-yl-1-d)benzene (*d*^{2}-3a)

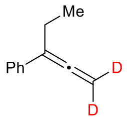

The di-deuterated allene ***d*^{2}-3a** was prepared according to the procedures same to the previous for the preparing of di-deuterated allene ***d*^{2}-3g** by starting with ethynylbenzene (1.1 mL, 10.0 mmol). Desired allene was finally obtained as a colorless oil (0.91 g, yield: 82%).

^{1}H NMR (300 MHz, $CDCl_3$) δ 7.44 – 7.38 (m, 2H), 7.35 – 7.26 (m, 2H), 7.22 – 7.13 (m, 1H), 2.42 (q, J = 7.3 Hz, 2H), 1.15 (t, J = 7.3 Hz, 3H).

MS(EI): *m/z* 146 $[M]^+$.

1-Methoxy-4-(penta-1,2-dien-3-yl-1-d)benzene (*d*-3g)

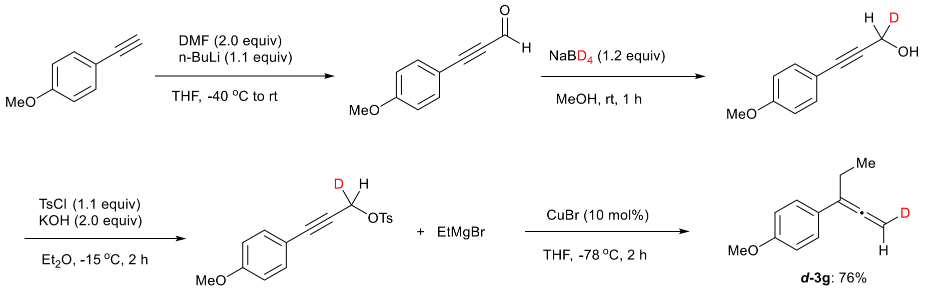

To a 100 mL oven-dried flask were added 4-ethynylanisole (1.32 g, 10 mmol) and anhydrous THF (20 mL), then stirred under –40 °C. *n*-BuLi (7.0 mL, 1.59 M in *n*-hexane) was then added dropwise. The solution was stirred at –40 °C for 30 min, followed by adding anhydrous DMF (1.6 mL, 20 mmol) in one portion. The mixture was allowed to slowly warm-up to room temperature. After stirring for 1 h at room temperature, the reaction mixture was poured into a vigorously stirred mixture of NaH_2PO_4 (3.2 g in 20 mL water, 20 mmol) and *tert*-butyl methyl ether (MTBE, 20 mL). The mixture was extracted with MTBE, washed with brine, dried over anhydrous Na_2SO_4, filtered and concentrated *in vacuo*. The residue was purified by silica gel column chromatography (*n*-hexane/EtOAc: 10/1)

to afford 3-(4-methoxyphenyl)propiolaldehyde as a light-yellow solid (1.48 g, yield: 93%).[22]

Propargyl alcohol were prepared according to the known procedure with modification.[23] In a glovebox, to a stirred solution of 3-(4-methoxyphenyl)propiolaldehyde (1.44 g, 9.0 mmol) in methanol (10 mL), $NaBD_4$ (0.45 g, 10.8 mmol) was added portion-wise to the mixture and keep stirring for 1 h. After neutralization with hydrochloric acid (2 M, 5 mL), the mixture was extracted with EtOAc, washed with brine, dried over anhydrous Na_2SO_4, filtered and concentrated *in vacuo*. The residue was purified by silica gel column chromatography (*n*-hexane/EtOAc: 4/1) to afford mono-deuterated propargyl alcohol as a white solid (1.39 g, yield: 95%).

A solution of previously obtained mono-deuterated propargyl alcohol (1.39 g, 8.5 mmol) and TsCl (1.8 g, 9.4 mmol) in dry Et_2O (30 mL) was stirred under –15 °C. Powder KOH (1.92 g, 4.0 equiv) was added portion-wise over 30 min, and then keep stirring for 2 h. After the alcohol fully consumed (by checking TLC), filtered through a short pad of celite, and the filtrate was concentrated under vacuum to give the crude, which purificated by recrystallization (Et_2O/*n*-hexane) under –18 °C to afford mono-deuterated *O*-tosylates as a white solid (2.50 g, yield: 92%).

In a glovebox, to a dried flask were added previously obtained mono-deuterated *O*-tosylates (2.5 g, 7.88 mmol), CuBr (112 mg, 0.79 mmol), and THF (30 mL), and under argon. Then the resulting mixture was cooled to –78 ^{o}C followed by dropwise addition of EtMgBr (15.7 mL, 1 M in THF, 15.7 mmol) over 20 min. The resulting mixture was stirred for 1 h at –78 ^{o}C and quenched with a saturated aqueous solution of NH_4Cl (15 mL). The mixture was then extracted with Et_2O, washed with brine, dried over anhydrous Na_2SO_4, filtered and concentrated *in vacuo*. The residue was purified by silica gel column chromatography (*n*-hexane/DCM: 10/1) to afford titled allene ***d*-3g** (1.06 g, yield: 76%).

^{1}H NMR (300 MHz, $CDCl_3$) δ 7.36 (d, *J* = 8.9 Hz, 2H), 6.89 (d, *J* = 8.9 Hz, 2H), 5.10 (dt, *J* = 4.1, 2.3 Hz, 1H), 3.82 (s, 3H), 2.43 (qd, *J* = 7.3, 3.6 Hz, 2H), 1.17 (t, *J* = 7.4 Hz, 3H).

MS(EI): *m/z* 175 $[M]^+$.

Synthesis of pent-1-yn-3-ylbenzene (12)

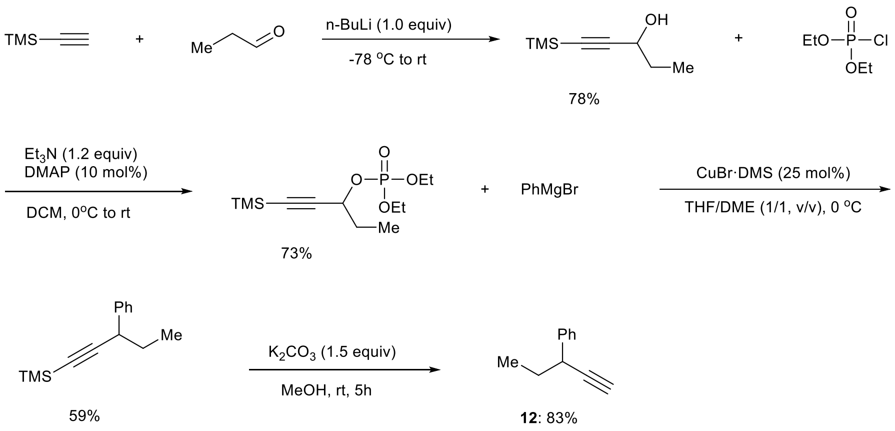

In an oven-dried flask was charged with trimethylsilylacetylene (2.85 mL, 20 mmol) and THF (20 mL) stirred at –78 °C under N_2. Then *n*-BuLi (1.6 M in hexane, 12.5 mL, 20 mmol, 1.0 equiv) was added dropwise over 10 min. And stirred for 30 min and propionaldehyde (1.16 mL, 20 mmol, 1.0 equiv) was added dropwise. The mixture was allowed to warm to room temperature and was stirred for 1 h. The mixture was poured into a saturated aqueous NH_4Cl solution (20 mL) and the aqueous layer was extracted with Et_2O. The combined organic layers were washed with brine, dried over Na_2SO_4, filtered and concentrated *in vacuo* to afford the crude, which was purified by silica gel column chromatography (*n*-hexane/EtOAc: 8/1) to afford propargyl alcohol as a colorless oil (2.45 g, yield: 78%).

Propargyl phosphate were prepared according to the known procedure.[24] In an oven-dried flask was charged with propargyl alcohol (1.56 g, 10 mmol), DMAP (12.2 mg, 0.1 mmol), and dry DCM (15 mL) stirred at 0 °C under N_2. Then diethyl chlorophosphate (1.87 mL, 13 mmol) and Et_3N (1.67 mL, 12 mmol) were added to the reaction flask. Then the reaction mixture was allowed to warm to room temperature until the full consumption of the propargyl alcohol, the mixture was diluted with Et_2O and washed with saturated NH_4Cl solution. The organic phase was then washed with brine, dried with Na_2SO_4, and concentrated *in vacuo*. The crude was purified by silica gel chromatography (*n*-hexane/EtOAc: 20/1) to afford propargyl phosphate as a colorless oil (2.15 g, yield: 73%).

Propargyl silane were prepared according to the known procedure.[25] To an ice-cold suspension of CuBr·Me_2S (136 mg, 0.66 mmol, 0.25 equiv) in THF (5 mL) and DME (5 mL) was added PhMgBr (fresh prepared, 6.4 mmol, 2.5 equiv) dropwise. The resulting mixture was stirred at 0 °C for 30 min and propargyl phosphate (0.75 g, 2.56 mmol) in THF (20 mL) was added dropwise. The mixture was stirred at 0 °C for 1 h and diluted with saturated NH_4Cl. The organic phase was then washed with brine, dried with Na_2SO_4, and concentrated *in vacuo*. The crude was purified by silica gel chromatography (*n*-hexane) to afford propargyl silane as a colorless oil (0.33 g, yield: 59%).

To a solution of propargyl silane (216 mg, 1 mmol) in MeOH (8 mL) was added K_2CO_3 (208 mg, 1.5 mmol). The mixture was stirred at room temperature for 5 h, diluted with Et_2O, and filtered through a pad of Celite. The filtrate was concentrated to afford an oil, which was purified by chromatography on silica gel chromatography (*n*-hexane/EtOAc: 50/1) to afford title product pent-1-yn-3-ylbenzene as a light-yellow oil (120 mg, yield: 83%).

^{1}H NMR (300 MHz, $CDCl_3$) δ 7.47 – 7.15 (m, 5H), 3.59 (td, J = 7.2, 2.4 Hz, 1H), 2.29 (d, J = 2.5 Hz, 1H), 1.92 – 1.73 (m, 2H), 1.02 (t, J = 7.4 Hz, 3H).

^{13}C NMR (75 MHz, $CDCl_3$) δ 141.5, 128.6, 127.6, 126.9, 86.0, 71.1, 39.2, 31.5, 11.8.

MS(EI): *m/z* 144 $[M]^+$. The chemical shifts were consistent with those reported in the literature.[26]

Synthesis of silylboronates

Silylboranates, such as $Et_3SiBPin$, $^nPr_3SiBpin$, $^tBuMe_2SiBpin$, and $TMS_3SiBpin$ were prepared according to the procedures previously reported.[1] $PhMe_2SiBpin$ was purchased from TCI. A typical experimental procedure for the preparation of $Et_3SiBPin$ was described below.

An oven-dried vial was charged with $[Ir(COD)OMe]_2$ (66.3 mg, 0.1 mmol), dtbpy (54.0 mg, 0.2 mmol), B_2pin_2 (5.1 g, 20 mmol), cyclohexane (10.0 mL), and triethylsilane (12.8 mL, 80 mmol) inside a nitrogen filled glovebox. The resulting dark brown solution was heated at 80 °C overnight outside the glovebox. After being cooled to room temperature, the crude reaction mixture was concentrated in vacuo, and the residue was purified by flash column chromatography to afford the $Et_3SiBpin$ as colorless oil (3.5 g, yield: 72%).

^{1}H NMR (300 MHz, $CDCl_3$) δ 1.22 (s, 12H), 0.95 (t, J = 8.0 Hz, 9H), 0.59 (dd, J = 15.8, 7.9 Hz, 6H).

MS(EI): *m/z* 242 $[M]^+$. The chemical shifts were consistent with those reported in the literature.[1]

2.2. Optimization of the reaction conditions

Table S1. Screening suitable silylboronate for the coupling reaction[a]

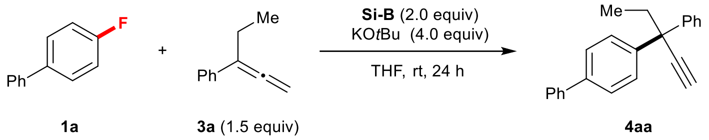

Entry	Si-B	Conversion (%)	4aa (%)[b]
1	$Et_3SiBpin$	53	34
**2**	**$PhMe_2SiBpin$**	**58**	**41**
3	$TMS_3SiBpin$	66	36
4	*n*-$Pr_3SiBpin$	16	12
5	$^tBuMe_2SiBpin$	25	11

[a] Unless otherwise noted, reactions were conducted with **1a** (17.2 mg, 0.1 mmol) and THF (1.0 mL), indicated amount of **3a**, **Si-B**, KO*t*Bu and react at room temperature for 24 h.

[b] Determined by ^{19}F NMR and ^1H NMR spectroscopy using 3-fluoropyridine as an internal standard.

Table S2. Screening for the proper equivalent combination of allene and silylboronate[a]

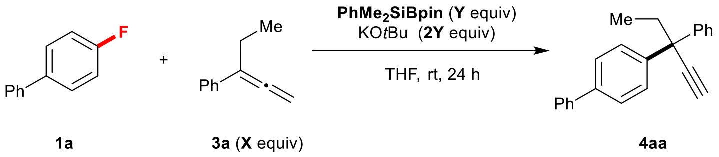

Entry	X	Y	Conversion (%)	4aa (%)[b]
1	1.5	2.0	58	41
2[c]	1.5	2.0	100	73
3	3.0	4.0	100	91
**4**	**3.0**	**3.0**	**95**	**76**
5	3.0	2.0	56	34

[a] Unless otherwise noted, reactions were conducted with **1a** (17.2 mg, 0.1 mmol) and THF (1.0 mL), indicated amount of **3a**, $PhMe_2SiBpin$, KO*t*Bu and react at room temperature for 24 h.

[b] Determined by ^{19}F NMR and ^1H NMR spectroscopy using 3-fluoropyridine as an internal standard.

[c] Using 0.05 mmol of **1a**.

Table S3. Screening solvent for the coupling reaction[a]

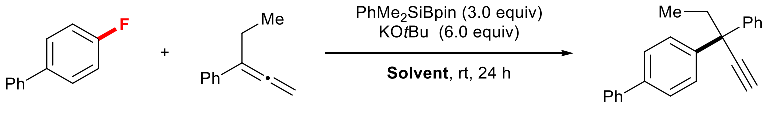

Entry	Solvent	Conversion (%)	**4aa** (%)[b]
1	THF	95	76
2	DME	94	89
**3**	**diglyme**	**98**	**95**
4	triglyme	94	91
5	tetraglyme	96	84
6	dioxane	71	58
7	DMF	15	trace
8	toluene	23	N.D.

[a] Unless otherwise noted, reactions were conducted with **1a** (17.2 mg, 0.1 mmol), **3a** (43.2 mg, 0.3 mmol), $PhMe_2SiBpin$ (78.6 mg, 0.3 mmol), and KO*t*Bu (67.2 mg, 0.6 mmol) in indicated solvent (1.0 mL) at room temperature for 24 h.

[b] Determined by ^{19}F NMR and ^{1}H NMR spectroscopy using 3-fluoropyridine as an internal standard.

Table S4. Reaction time optimization for the coupling reaction[a]

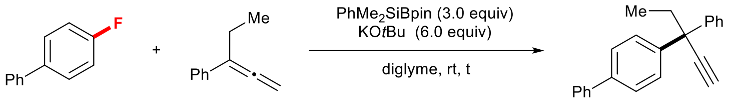

Entry	t (h)	Conversion (%)	**4aa** (%)
1	4	80	71
2	8	89	82
**3**	**12**	**98**	**94**
4	24	97	95

[a] Unless otherwise noted, reactions were conducted with **1a** (17.2 mg, 0.1 mmol), **3a** (43.2 mg, 0.3 mmol), $PhMe_2SiBpin$ (78.6 mg, 0.3 mmol), and KO*t*Bu (67.2 mg, 0.6 mmol) in diglyme (1.0 mL) at room temperature for indicated hours.

[b] Determined by ^{19}F NMR and ^{1}H NMR spectroscopy using 3-fluoropyridine as an internal standard.

Table S5. Variations from the optimal reaction conditions[a]

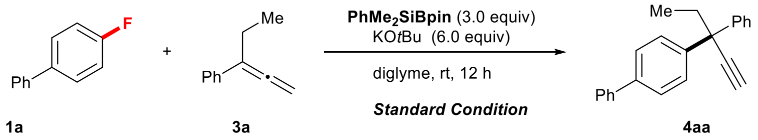

Entry	Variations from "***Standard Condition***"	**4aa**(%)[b]
1	none	94
2	Without $PhMe_2SiBpin$	0
3	Without KO*t*Bu	0
4	KOMe instead of KO*t*Bu	n.d.
5	NaO*t*Bu, LiO*t*Bu or KHMDS instead of KO*t*Bu	trace
6[c]	$Et_3SiBpin$ instead of $PhMe_2SiBpin$	75(71)
7	2.0 equiv of **3a**, 2.0 equiv of $Et_3SiBpin$, and 4.0 equiv of KO*t*Bu instead	62
8	2.5 equiv of $PhMe_2SiBpin$, and 5.5 equiv of KO*t*Bu instead	86
9	2.0 equiv of **3a** instead	78
10	$PhMe_2SiBpin$ (1.5 equiv), and KO^tBu (3.0 equiv), and **3a** (1.5 equiv) under **50 ºC**	79
11	$Et_3SiBpin$ (1.5 equiv), KO^tBu (3.0 equiv), and **3a** (1.5 equiv) under **50 ºC**	38

[a] Unless otherwise noted, reactions were conducted with **1a** (17.2 mg, 0.1 mmol) and diglyme (1.0 mL), indicated amount of **3a**, silylboronate, KO*t*Bu were used and react at room temperature for 12 h.

[b] Determined by ^{19}F NMR and ^{1}H NMR spectroscopy using 3-fluoropyridine as an internal standard.

[c] The isolated yield was shown in the parenthesis.

2.3. General procedures for the cross-coupling of aryl fluorides and allenes

General procedure for the optimization of the cross-coupling reaction (General procedure E)

In a N_2 filled glovebox, to a flame-dried screw-capped test tube was added 4-fluorobiphenyl **1a** (17.2 mg, 0.10 mmol), silyl boronates, solvent (1.0 mL), allene **3a**, and then base, sequentially. The tube was then sealed and moved out from the glovebox. The solution was stirred at room temperature for indicated hours. The reaction tube was diluted with Et_2O (5 mL) and water (2 mL), then extracted with Et_2O, concentrated under vacuum, followed by 3-fluoropyridine (8.6 μL, 0.1 mmol) as an internal standard. Corresponding yields were copied from the ^{1}H NMR and ^{19}F NMR analysis.

General procedure for the cross-coupling reaction under room temperature (General procedure F)

In a N_2 filled glovebox, to a flame-dried screw-capped test tube was added aryl fluorides **1** (0.2 mmol), silyl boronates (0.6 mmol), dry diglyme (2.0 mL), and allenes **3** (0.6 mmol) KO*t*Bu (135 mg, 1.2 mmol), sequentially. Then the tube was sealed and moved out from the glovebox. The solution was stirred at room temperature for 12 h. The reaction tube was diluted with Et_2O (5 mL) and water, then extracted with Et_2O, washed with brine, dried over Na_2SO_4, then concentrated under vacuum, followed by adding 3-fluoropyridine (8.6 μL, 0.1 mmol) as an internal standard. After NMR analysis was conducted. The mixture was then concentrated again to give the crude, which was purified by column chromatography on silica gel to give the corresponding products **4**.

General procedure for the cross-coupling reaction under heating (General procedure G)

In a N_2 filled glovebox, to a flame-dried screw-capped test tube was added KO*t*Bu (33.6 mg, 0.3 mmol), dry diglyme (0.5 mL), silyl boronates (0.3 mmol), and then stirring for 5 min under room temperature, then allenes **3a** (43.2 mg, 0.3 mmol) was added into the mixture, which was continued to stir for 5 min followed by the addition of organic fluorides **1** or **2** (0.2 mmol) and KO*t*Bu (33.6 mg, 0.3 mmol), and dry diglyme (1.0 mL). Then the tube was sealed and moved out from the glovebox. The solution was stirred at 50 ^{o}C using a liquid phase organic synthesizer SIBATA CP-1000 for 12 h. The reaction tube was diluted with Et_2O (5 mL) and water, then extracted with Et_2O, washed with brine, dried over Na_2SO_4, then concentrated under vacuum, followed by adding 3-fluoropyridine (8.6 μL, 0.1 mmol) as an internal standard. After NMR analysis was conducted. The mixture was then concentrated again to give the crude, which was purified by column chromatography on silica gel to give the corresponding products **4** or **5**.

3. Characterization Data of Products

4-(3-Phenylpent-1-yn-3-yl)biphenyl (4aa)

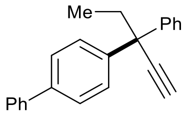

Compound **4aa** was prepared according to the **General procedure F** from **1a** (34.4 mg, 0.2 mmol) and **3a** (86.4 mg, 0.6 mmol) using $Et_3SiBpin$, and purified by silica gel column chromatography (*n*-hexane/DCM: 10/1) as a white solid (43.2 mg, yield: 73%), m.p.: 50.2 – 51.3 °C.

^{1}H NMR (500 MHz, $CDCl_3$) δ 7.59 – 7.53 (m, 2H), 7.55 – 7.45 (m, 6H), 7.45 – 7.37 (m, 2H), 7.35 – 7.27 (m, 3H), 7.25 – 7.18 (m, 1H), 2.60 (s, 1H), 2.35 (q, *J* = 7.3 Hz, 2H), 0.99 (t, *J* = 7.3 Hz, 3H).

^{13}C NMR (126 MHz, $CDCl_3$) δ 144.7, 144.1, 140.8, 139.5, 128.8, 128.4, 127.9, 127.5, 127.3, 127.2, 127.0, 126.7, 87.9, 74.0, 49.9, 34.3, 10.1.

IR (KBr): 3280, 3056, 3025, 2972, 2933, 2875, 1484, 1445, 1073, 1007, 832, 759, 731, 694, 639 cm^{-1}

HRMS (EI) [$C_{23}H_{20}$] $[M]^+$ calculated: 296.1565, found: 296.1564.

3-(3-Phenylpent-1-yn-3-yl)biphenyl (4ba)

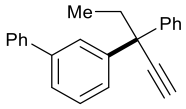

Compound **4ba** was prepared according to the **General procedure F** from **1b** (34.4 mg, 0.2 mmol) and **3a** (86.4 mg, 0.6 mmol) using $Et_3SiBpin$, and purified by silica gel column chromatography (*n*-hexane/DCM: 10/1) as a colorless oil (44.5 mg, yield: 75%).

^{1}H NMR (300 MHz, $CDCl_3$) δ 7.71 (t, *J* = 2.0 Hz, 1H), 7.57 – 7.52 (m, 2H), 7.50 – 7.46 (m, 2H), 7.45 – 7.37 (m, 4H), 7.36 – 7.26 (m, 4H), 7.22 – 7.17 (m, 1H), 2.59 (s, 1H), 2.36 (q, *J* = 7.2 Hz, 2H), 0.99 (t, *J* = 7.2 Hz, 3H).

^{13}C NMR (126 MHz, $CDCl_3$) δ 145.4, 144.8, 141.4, 141.2, 128.8, 128.7, 128.3, 127.5, 127.4, 126.7, 126.5, 126.4, 125.6, 87.9, 74.1, 50.2, 34.3, 10.1.

IR (KBr): 3295, 3061, 3031, 2968, 2936, 2877, 1599, 1490, 1477, 1447, 1413, 1269, 1193, 914, 758, 700, 634 cm^{-1}

HRMS (EI) [$C_{23}H_{20}$] $[M]^+$ calculated: 296.1565, found: 296.1568.

1-Methoxy-2-(3-phenylpent-1-yn-3-yl)benzene (4ca)

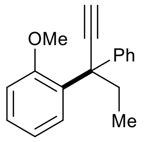

Compound **4ca** was prepared according to the **General procedure F** from **1c** (25.2 mg, 0.2 mmol) and **3a** (86.4 mg, 0.6 mmol) using $PhMe_2SiBpin$, and purified by silica gel column chromatography (*n*-hexane/DCM: 9/1) as a white solid (24.1 mg, yield: 48%), m.p.: 79.0 – 80.5 °C.

^{1}H NMR (500 MHz, $CDCl_3$) δ 7.78 (dd, J = 7.7, 1.7 Hz, 1H), 7.36 – 7.32 (m, 2H), 7.29 – 7.20 (m, 3H), 7.18 – 7.12 (m, 1H), 7.00 (td, J = 7.6, 1.2 Hz, 1H), 6.81 (dd, J = 8.1, 1.2 Hz, 1H), 3.39 (s, 3H), 2.61 (dq, J = 12.7, 7.3 Hz, 1H), 2.52 (s, 1H), 2.25 (dq, J = 12.7, 7.3 Hz, 1H), 0.89 (t, J = 7.3 Hz, 3H).

^{13}C NMR (126 MHz, $CDCl_3$) δ 157.6, 145.5, 132.7, 128.6, 128.5, 127.7, 126.8, 125.9, 120.5, 113.3, 88.2, 72.9, 55.7, 48.2, 32.1, 10.0.

IR (KBr): 3282, 3053, 2980, 2957, 2935, 2875, 2839, 1580, 1489, 1457, 1432, 1285, 1243, 1174, 1084, 1024, 751, 697, 642 cm^{-1}

HRMS (EI) [$C_{18}H_{18}O$] $[M]^+$ calculated: 250.1358, found: 250.1354.

1-Phenoxy-3-(3-phenylpent-1-yn-3-yl)benzene (4da)

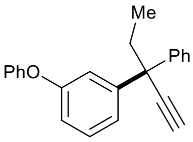

Compound **4da** was prepared according to the **General procedure F** from **1d** (37.6 mg, 0.2 mmol) and **3a** (86.4 mg, 0.6 mmol) using $PhMe_2SiBpin$, and purified by silica gel column chromatography (*n*-hexane/DCM: 9/1) as a light-yellow oil (54.2 mg, yield: 87%).

^{1}H NMR (500 MHz, $CDCl_3$) δ 7.46 – 7.41 (m, 2H), 7.33 – 7.26 (m, 4H), 7.25 – 7.19 (m, 3H), 7.19 – 7.14 (m, 1H), 7.07 (tt, J = 7.3, 1.1 Hz, 1H), 6.99 – 6.94 (m, 2H), 6.81 (ddd, J = 8.0, 2.4, 1.1 Hz, 1H), 2.56 (s, 1H), 2.29 (qd, J = 7.2, 1.7 Hz, 2H), 0.97 (t, J = 7.2 Hz, 3H).

^{13}C NMR (126 MHz, $CDCl_3$) δ 157.3, 156.9, 147.1, 144.5, 129.8, 129.5, 128.3, 127.3, 126.8, 123.2, 122.6, 118.7, 118.6, 116.9, 87.6, 74.1, 50.1, 34.2, 10.0.

IR (KBr): 3296, 3060, 3032, 2972, 2937, 2877, 1581, 1487, 1434, 1275, 1242, 1210, 1165, 1073, 857, 751, 699, 642 cm^{-1}

HRMS (ESI) [$C_{23}H_{21}O$] $[M+H]^+$ calculated: 313.1592, found: 313.1595.

1-Phenoxy-4-(3-phenylpent-1-yn-3-yl)benzene (4ea)

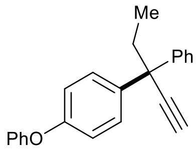

Compound **4ea** was prepared according to the **General procedure F** from **1e** (37.6 mg, 0.2 mmol) and **3a** (86.4 mg, 0.6 mmol) using $PhMe_2SiBpin$, and purified by silica gel column chromatography (*n*-hexane/DCM: 5/1) as a light-yellow oil (51.4 mg, yield: 82%).

^{1}H NMR (500 MHz, $CDCl_3$) δ 7.48 – 7.41 (m, 2H), 7.40 – 7.36 (m, 2H), 7.35 – 7.26 (m, 4H), 7.23 – 7.18 (m, 1H), 7.12 – 7.05 (m, 1H), 7.02 – 6.97 (m, 2H), 6.94 – 6.89 (m, 2H), 2.58 (s, 1H), 2.30 (q, *J* = 7.3 Hz, 2H), 0.97 (t, *J* = 7.2 Hz, 3H).

^{13}C NMR (126 MHz, $CDCl_3$) δ 157.2, 155.9, 144.8, 139.7, 129.8, 128.8, 128.3, 127.4, 126.7, 123.4, 119.1, 118.4, 88.0, 73.9, 49.6, 34.5, 10.1.

IR (KBr): 3298, 3060, 3037, 2972, 2935, 2877, 1588, 1487, 1247, 1171, 1015, 869, 839, 751, 698, 638 cm^{-1}

HRMS (ESI) [$C_{23}H_{21}O$] $[M+H]^+$ calculated: 313.1592, found: 313.1597.

1-(4-(3-Phenylpent-1-yn-3-yl)phenyl)naphthalene (4fa)

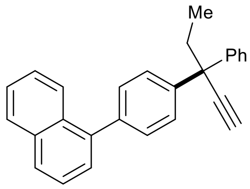

Compound **4fa** was prepared according to the **General procedure F** from **1f** (44.4 mg, 0.2 mmol) and **3a** (86.4 mg, 0.6 mmol) using $Et_3SiBpin$, and purified by silica gel column chromatography (*n*-hexane/DCM: 10/1) as a white solid (53.0 mg, yield: 82%), m.p.: 109.4 – 110.6 °C.

^{1}H NMR (500 MHz, $CDCl_3$) δ 7.90 (dd, *J* = 16.2, 8.3 Hz, 2H), 7.83 (d, *J* = 8.2 Hz, 1H), 7.57 – 7.52 (m, 4H), 7.52 – 7.45 (m, 2H), 7.45 – 7.37 (m, 4H), 7.38 – 7.31 (m, 2H), 7.27 – 7.21 (m, 1H), 2.64 (s, 1H), 2.40 (q, *J* = 7.4 Hz, 2H), 1.04 (t, *J* = 7.2 Hz, 3H).

^{13}C NMR (126 MHz, $CDCl_3$) δ 144.7, 144.0, 140.0, 139.0, 133.9, 131.6, 130.0, 128.4, 127.7, 127.5, 127.3, 127.1, 126.8, 126.2, 126.1, 125.9, 125.5 , 88.0, 74.1, 50.0, 34.4, 10.2.

IR (KBr): 3276, 3057, 3031, 2974, 2931, 2872, 1491, 1446, 1396, 1019, 841, 803, 781, 699, 633 cm^{-1}

HRMS (ESI) [$C_{27}H_{23}$] $[M+H]^+$ calculated: 347.1800, found: 347.1803.

4-Methyl-4'-(3-phenylpent-1-yn-3-yl)biphenyl (4ga)

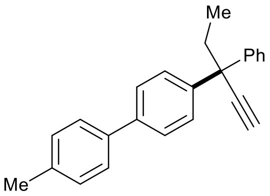

Compound **4ga** was prepared according to the **General procedure F** from **1g** (37.2 mg, 0.2 mmol) and **3a** (86.4 mg, 0.6 mmol) using $Et_3SiBpin$, and purified by silica gel column chromatography (*n*-hexane) as a white solid (38.9 mg, yield: 63%), m.p.: 52.7 – 53.9 °C.

^{1}H NMR (300 MHz, $CDCl_3$) δ 7.53 – 7.44 (m, 8H), 7.35 – 7.27 (m, 2H), 7.25 – 7.19 (m, 3H), 2.60 (s, 1H), 2.38 (s, 3H), 2.37 – 2.32 (m, 2H), 0.99 (t, J = 7.3 Hz, 3H).

^{13}C NMR (126 MHz, $CDCl_3$) δ 144.8, 143.7, 139.4, 137.9, 137.1, 129.6, 128.3, 127.8, 127.5, 127.0, 126.8, 126.7, 88.0, 74.0, 49.9, 34.3, 21.3, 10.1.

IR (KBr): 3297, 3054, 3025, 2972, 2934, 2875, 1600, 1495, 1445, 1376, 1263, 1006, 842, 809, 757, 699, 637 cm^{-1}

HRMS (ESI) [$C_{24}H_{23}$] [M+H]$^+$ calculated: 311.1800, found: 311.1797.

4-Methoxy-4'-(3-phenylpent-1-yn-3-yl)biphenyl (4ha)

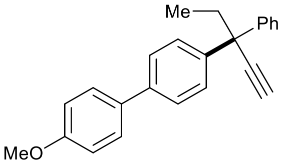

Compound **4ha** was prepared according to the **General procedure F** from **1h** (40.4 mg, 0.2 mmol) and **3a** (86.4 mg, 0.6 mmol) using $PhMe_2SiBpin$, and purified by silica gel column chromatography (*n*-hexane/DCM: 5/1) as a white solid (57.9 mg, yield: 89%), m.p.: 73.4 – 74.2 °C.

^{1}H NMR (500 MHz, $CDCl_3$) δ 7.52 – 7.44 (m, 8H), 7.34 – 7.26 (m, 2H), 7.24 – 7.17 (m, 1H), 7.01 – 6.79 (m, 2H), 3.81 (s, 3H), 2.59 (s, 1H), 2.34 (q, J = 6.8 Hz, 2H), 0.99 (t, J = 7.3 Hz, 3H).

^{13}C NMR (126 MHz, $CDCl_3$) δ 159.1, 144.8, 143.4, 139.0, 133.3, 128.3, 128.1, 127.8, 127.5, 126.7, 126.6, 114.2, 88.0, 73.9, 55.4, 49.9, 34.3, 10.1.

IR (KBr): 3297, 3032, 2973, 2933, 2875, 1604, 1496, 1448, 1376, 1287, 1253, 1180, 1036, 822, 760, 720, 700, 638, 506 cm^{-1}

HRMS (ESI) [$C_{24}H_{23}O$] [M+H]$^+$ calculated: 327.1749, found: 327.1743.

4-(Benzyloxy)-4'-(3-phenylpent-1-yn-3-yl)biphenyl (4ia)

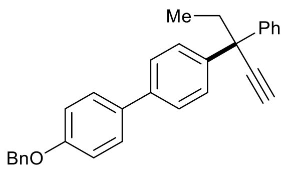

Compound **4ia** was prepared according to the **General procedure F** from **1i** (55.6 mg, 0.2 mmol) and **3a** (86.4 mg, 0.6 mmol) using $PhMe_2SiBpin$, and purified by silica gel column chromatography (*n*-hexane/DCM: 5/1) as a light-yellow solid (67.4 mg, yield: 84%), m.p.: 97.5 – 98.9 °C.

^{1}H NMR (500 MHz, $CDCl_3$) δ 7.52 – 7.43 (m, 10H), 7.41 – 7.37 (m, 2H), 7.35 – 7.28 (m, 3H), 7.23 – 7.19 (m, 1H), 7.06 – 6.97 (m, 2H), 5.08 (s, 2H), 2.60 (s, 1H), 2.34 (q, J = 7.1 Hz, 2H), 0.99 (t, J = 7.2 Hz, 3H).

^{13}C NMR (126 MHz, $CDCl_3$) δ 158.4, 144.8, 143.4, 139.0, 137.0, 133.6, 128.7, 128.3, 128.2, 127.8, 127.6, 127.5, 126.7, 126.6, 115.2, 88.0, 73.9, 70.1, 49.9, 34.3, 10.1.

IR (KBr): 3285, 3029, 2974, 2938, 2876, 1606, 1496, 1383, 1249, 1176, 1037, 810, 730, 699, 640 cm^{-1}

HRMS (ESI) [$C_{30}H_{27}O$] $[M+H]^+$ calculated: 403.2062, found: 403.2043.

5-(4-(3-Phenylpent-1-yn-3-yl)phenyl)benzo[*d*][1,3]dioxole (4ja)

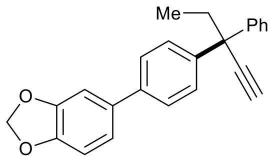

Compound **4ja** was prepared according to the **General procedure F** from **1j** (43.2 mg, 0.2 mmol) and **3a** (86.4 mg, 0.6 mmol) using $PhMe_2SiBpin$, and purified by silica gel column chromatography (*n*-hexane/DCM: 5/1) as a light-yellow oil (61.3 mg, yield: 90%).

^{1}H NMR (500 MHz, $CDCl_3$) δ 7.49 – 7.44 (m, 4H), 7.43 (d, J = 8.7 Hz, 2H), 7.34 – 7.26 (m, 2H), 7.23 – 7.19 (m, 1H), 7.05 – 7.01 (m, 2H), 6.85 (d, J = 7.9 Hz, 1H), 5.96 (s, 2H), 2.60 (s, 1H), 2.34 (q, J = 7.2 Hz, 2H), 0.99 (t, J = 7.2 Hz, 3H).

^{13}C NMR (126 MHz, $CDCl_3$) δ 148.2, 147.1, 144.7, 143.7, 139.1, 135.2, 128.3, 127.8, 127.4, 126.7, 120.6, 108.7, 107.7, 101.2, 87.9, 74.0, 49.9, 34.3, 10.1.

IR (KBr): 3294, 3058, 3029, 2972, 2934, 2876, 1607, 1483, 1441, 1408, 1338, 1224, 1106, 1041, 936, 807, 760, 700, 643 cm^{-1}

HRMS (ESI) [$C_{24}H_{21}O_2$] $[M+H]^+$ calculated: 341.1542, found: 341.1534.

4-Chloro-4'-(3-phenylpent-1-yn-3-yl)biphenyl (4ka)

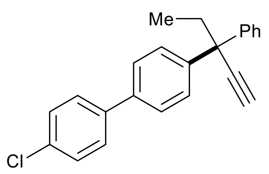

Compound **4ka** was prepared according to the **General procedure F** from **1k** (41.2 mg, 0.2 mmol) and **3a** (86.4 mg, 0.6 mmol) using $Et_3SiBpin$, and purified by silica gel column chromatography (*n*-hexane/DCM: 10/1) as a light-yellow oil (34.6 mg, yield: 52%).

^{1}H NMR (500 MHz, $CDCl_3$) δ 7.55 – 7.44 (m, 8H), 7.39 – 7.35 (m, 2H), 7.33 – 7.28 (m, 2H), 7.25 – 7.18 (m, 1H), 2.60 (s, 1H), 2.34 (q, J = 7.2 Hz, 2H), 0.99 (t, J = 7.2 Hz, 3H).

^{13}C NMR (126 MHz, $CDCl_3$) δ 144.6, 144.5, 139.2, 138.2, 134.8, 133.4, 129.0, 128.4, 128.0, 127.4, 126.9, 126.8, 87.8, 74.1, 49.9, 34.3, 10.1.

IR (KBr): 3301, 3060, 3031, 2970, 2934, 2877, 1597, 1486, 1445, 1389, 1265, 1092, 1005, 846, 813, 766, 700, 637 cm^{-1}

HRMS (ESI) [$C_{23}H_{20}Cl$] $[M+H]^+$ calculated: 331.1254, found: 331.1257.

4-(3-Phenylpent-1-yn-3-yl)-4'-(trifluoromethyl)biphenyl (4la)

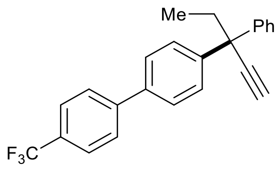

Compound **4la** was prepared according to the **General procedure F** from **1l** (48.0 mg, 0.2 mmol) and **3a** (86.4 mg, 0.6 mmol) using $Et_3SiBpin$, and purified by silica gel column chromatography (*n*-hexane/DCM: 10/1) as a light-yellow oil (37.8 mg, yield: 52%).

^{1}H NMR (300 MHz, $CDCl_3$) δ 7.67 (s, 4H), 7.54 (s, 4H), 7.51 – 7.46 (m, 2H), 7.38 – 7.28 (m, 2H), 7.27 – 7.20 (m, 1H), 2.63 (s, 1H), 2.37 (q, J = 7.3 Hz, 2H), 1.01 (t, J = 7.2 Hz, 3H).

^{13}C NMR (126 MHz, $CDCl_3$) δ 145.2, 144.5, 144.3, 138.0, 129.3 (d, J = 32.4 Hz), 128.4, 128.1, 127.5, 127.4, 127.2, 126.9, 125.8, 124.4 (d, J = 272.4 Hz), 87.7, 74.2, 50.0, 34.3, 10.0.

^{19}F NMR (282 MHz, $CDCl_3$) δ -62.89 (s, 3F).

IR (KBr): 3297, 3060, 3029, 2968, 2934, 2876, 1617, 1492, 1446, 1328, 1165, 1129, 1070, 1006, 822, 765, 700 cm^{-1}

HRMS (ESI) [$C_{24}H_{20}F_3$] $[M+H]^+$ calculated: 365.1517, found: 365.1517.

1-(3-Methoxypropyl)-3-(3-phenylpent-1-yn-3-yl)benzene (4ma)

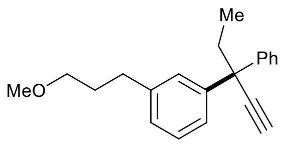

Compound **4ma** was prepared according to the **General procedure F** from **1m** (33.6 mg, 0.2 mmol) and **3a** (86.4 mg, 0.6 mmol) using $PhMe_2SiBpin$, and purified by silica gel column chromatography (*n*-hexane/EtOAc: 20/1) as a colorless oil (9.9 mg, yield: 17%).

^{1}H NMR (500 MHz, $CDCl_3$) δ 7.54 – 7.35 (m, 2H), 7.32 – 7.26 (m, 3H), 7.25 – 7.17 (m, 3H), 7.04 (dt, *J* = 7.2, 1.5 Hz, 1H), 3.34 (t, *J* = 6.4 Hz, 2H), 3.31 (s, 3H), 2.77 – 2.59 (m, 2H), 2.58 (s, 1H), 2.31 (q, *J* = 7.2 Hz, 2H), 1.97 – 1.73 (m, 2H), 0.95 (t, *J* = 7.3 Hz, 3H).

^{13}C NMR (126 MHz, $CDCl_3$) δ 145.0, 144.8, 141.9, 128.3, 127.8, 127.4, 126.8, 126.6, 125.0, 88.1, 73.8, 72.0, 58.7, 50.0, 34.3, 32.5, 31.4, 10.0.

IR (KBr): 3301, 3060, 2973, 2935, 2875, 2827, 1602, 1486, 1446, 1379, 1280, 1187, 1119, 907, 788, 753, 701, 635 cm^{-1}

HRMS (EI) [$C_{21}H_{24}O$] $[M]^+$ calculated: 292.1827, found: 292.1831.

4-(4-(3-Phenylpent-1-yn-3-yl)phenyl)pyridine (4na)

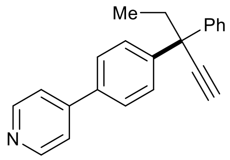

Compound **4na** was prepared according to the **General procedure F** from **1n** (34.6 mg, 0.2 mmol) and **3a** (86.4 mg, 0.6 mmol) using $PhMe_2SiBpin$, and purified by silica gel column chromatography (*n*-hexane/EtOAc: 8/1) as a light-yellow oil (46.3 mg, yield: 78%).

^{1}H NMR (500 MHz, $CDCl_3$) δ 8.92 – 8.44 (m, 2H), 7.56 (s, 4H), 7.50 – 7.44 (m, 4H), 7.36 – 7.28 (m, 2H), 7.27 – 7.19 (m, 1H), 2.63 (s, 1H), 2.36 (q, *J* = 7.2 Hz, 2H), 0.99 (t, *J* = 7.2 Hz, 3H).

^{13}C NMR (126 MHz, $CDCl_3$) δ 150.3, 147.9, 146.2, 144.4, 136.3, 128.4, 128.2, 127.4, 126.9, 121.6, 87.6, 74.3, 49.9, 34.2, 10.0.

IR (KBr): 3297, 3199, 3057, 3031, 2972, 2934, 2877, 1596, 1542, 1488, 1448, 1401, 1266, 1034, 993, 811, 749, 700, 638 cm^{-1}

HRMS (ESI) [$C_{22}H_{20}N$] $[M+H]^+$ calculated: 298.1596, found: 298.1597.

2-Phenyl-5-(3-phenylpent-1-yn-3-yl)pyridine (4oa)

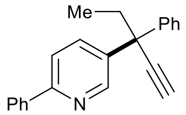

Compound **4oa** was prepared according to the **General procedure F** from **1o** (34.6 mg, 0.2 mmol) and **3a** (86.4 mg, 0.6 mmol) using $PhMe_2SiBpin$, and purified by silica gel column chromatography (*n*-hexane/EtOAc: 15/1) as a light-yellow solid (48.2 mg, yield: 81%), m.p.: 82.1 – 83.4 ℃.

^{1}H NMR (500 MHz, $CDCl_3$) δ 8.80 (dd, *J* = 2.5, 0.9 Hz, 1H), 8.01 – 7.94 (m, 2H), 7.76 (dd, *J* = 8.3, 2.5 Hz, 1H), 7.65 (dd, *J* = 8.3, 0.9 Hz, 1H), 7.49 – 7.41 (m, 4H), 7.41 – 7.36 (m, 1H), 7.34 – 7.28 (m, 2H), 7.26 – 7.20 (m, 1H), 2.64 (s, 1H), 2.36 (q, *J* = 7.1 Hz, 2H), 1.01 (t, *J* = 7.2 Hz, 3H).

^{13}C NMR (126 MHz, $CDCl_3$) δ 155.6, 148.7, 143.6, 138.9, 136.0, 133.4, 129.0, 128.8, 128.5, 127.4, 127.1, 126.9, 119.9, 86.7, 74.7, 48.4, 34.0, 9.9.

IR (KBr): 3199, 3060, 3024, 2971, 2935, 2874, 1595, 1556, 1493, 1473, 1443, 1376, 1019, 741, 695cm^{-1}

HRMS (ESI) [$C_{22}H_{20}N$] $[M+H]^+$ calculated: 298.1596, found: 298.1609.

2-Phenyl-4-(3-phenylpent-1-yn-3-yl)pyridine (4pa)

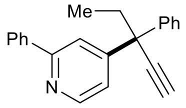

Compound **4pa** was prepared according to the **General procedure F** from **1p** (34.6 mg, 0.2 mmol) and **3a** (86.4 mg, 0.6 mmol) using $PhMe_2SiBpin$, and purified by silica gel column chromatography (*n*-hexane/EtOAc: 15/1) as a light-yellow oil (44.0 mg, yield: 74%).

^{1}H NMR (500 MHz, $CDCl_3$) δ 8.60 (dd, *J* = 5.2, 0.8 Hz, 1H), 7.98 – 7.92 (m, 2H), 7.84 (dd, *J* = 1.8, 0.8 Hz, 1H), 7.49 – 7.43 (m, 4H), 7.42 – 7.37 (m, 1H), 7.36 – 7.29 (m, 2H), 7.29 – 7.22 (m, 2H), 2.66 (s, 1H), 2.48 – 2.24 (m, 2H), 1.00 (t, *J* = 7.2 Hz, 3H).

^{13}C NMR (126 MHz, $CDCl_3$) δ 157.7, 154.6, 149.7, 143.1, 139.6, 129.1, 128.8, 128.6, 127.3, 127.2, 127.2, 121.0, 119.6, 86.4, 74.9, 50.1, 33.8, 9.9.

IR (KBr): 3297, 3057, 3033, 2973, 2937, 2876, 1593, 1550, 1492, 1472, 1445, 1393, 1276, 1193, 839, 775, 699, 635cm^{-1}

HRMS (ESI) [$C_{22}H_{20}N$] $[M+H]^+$ calculated: 298.1596, found: 298.1603.

1-(4-(3-Phenylpent-1-yn-3-yl)phenyl)-1*H*-pyrrole (4qa)

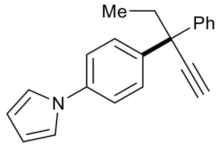

Compound **4qa** was prepared according to the **General procedure F** from **1q** (32.2 mg, 0.2 mmol) and **3a** (86.4 mg, 0.6 mmol) using $PhMe_2SiBpin$, and purified by silica gel column chromatography (*n*-hexane/DCM: 5/1) as a light-yellow oil (47.4 mg, yield: 83%).

^{1}H NMR (300 MHz, $CDCl_3$) δ 7.52 – 7.40 (m, 4H), 7.35 – 7.26 (m, 4H), 7.26 – 7.16 (m, 1H), 7.05 (t, *J* = 2.2 Hz, 2H), 6.32 (t, *J* = 2.2 Hz, 2H), 2.60 (s, 1H), 2.32 (q, *J* = 7.3 Hz, 2H), 0.98 (t, *J* = 7.3 Hz, 3H).

^{13}C NMR (126 MHz, $CDCl_3$) δ 144.6, 142.4, 139.3, 128.6, 128.4, 127.4, 126.8, 120.3, 119.4, 110.4, 87.7, 74.1, 49.7, 34.3, 10.0.

IR (KBr): 3295, 3059, 2972, 2934, 2877, 1611, 1515, 1484, 1446, 1332, 1191, 1119, 1070, 1021, 833, 757, 721, 700, 642 cm^{-1}

HRMS (ESI) [$C_{21}H_{20}N$] $[M+H]^+$ calculated: 286.1596, found: 286.1587.

1-Methyl-2-(4-(3-phenylpent-1-yn-3-yl)phenyl)-1*H*-indole (4ra)

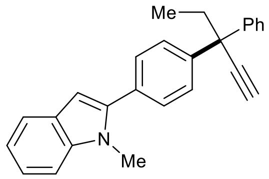

Compound **4ra** was prepared according to the **General procedure F** from **1r** (45.0 mg, 0.2 mmol) and **3a** (86.4 mg, 0.6 mmol) using $PhMe_2SiBpin$, and purified by silica gel column chromatography (*n*-hexane/DCM: 7/1) as a white solid (60.2 mg, yield: 86%), m.p.: 109.4 – 111.1 °C.

^{1}H NMR (500 MHz, $CDCl_3$) δ 7.62 (dt, *J* = 7.9, 1.0 Hz, 1H), 7.55 – 7.47 (m, 4H), 7.45 – 7.39 (m, 2H), 7.36 – 7.28 (m, 3H), 7.26 – 7.19 (m, 2H), 7.16 – 7.09 (m, 1H), 6.53 (s, 1H), 3.70 (s, 3H), 2.61 (s, 1H), 2.36 (q, *J* = 7.2 Hz, 2H), 1.01 (t, *J* = 7.2 Hz, 3H).

^{13}C NMR (126 MHz, $CDCl_3$) δ 144.7, 144.4, 141.3, 138.4, 131.1, 129.2, 128.4, 128.0, 127.6, 127.5, 126.8, 121.7, 120.5, 119.9, 109.7, 101.7, 87.8, 74.2, 50.0, 34.3, 31.4, 10.1.

IR (KBr): 3286, 3056, 2973, 2933, 2873, 1493, 1465, 1447, 1339, 1005, 834, 774, 740, 698, 641 cm^{-1}

HRMS (ESI) [$C_{26}H_{24}N$] $[M+H]^+$ calculated: 350.1909, found: 350.1907.

2-(4-(3-Phenylpent-1-yn-3-yl)phenyl)benzofuran (4sa)

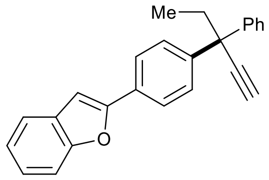

Compound **4sa** was prepared according to the **General procedure F** from **1s** (42.4 mg, 0.2 mmol) and **3a** (86.4 mg, 0.6 mmol) using $PhMe_2SiBpin$, and purified by silica gel column chromatography (*n*-hexane/DCM: 15/1) as a white solid (43.1 mg, yield: 64%), m.p.: 128.3 – 129.2 °C.

^{1}H NMR (500 MHz, $CDCl_3$) δ 7.80 – 7.75 (m, 2H), 7.56 – 7.53 (m, 1H), 7.52 – 7.48 (m, 3H), 7.47 – 7.43 (m, 2H), 7.33 – 7.26 (m, 2H), 7.27 – 7.17 (m, 3H), 6.96 (d, J = 0.9 Hz, 1H), 2.60 (s, 1H), 2.33 (q, J = 7.3 Hz, 2H), 0.98 (t, J = 7.2 Hz, 3H).

^{13}C NMR (126 MHz, $CDCl_3$) δ 155.8, 154.9, 145.4, 144.6, 129.3, 128.8, 128.4, 127.9, 127.4, 126.8, 124.9, 124.3, 123.0, 121.0, 111.3, 101.4, 87.7, 74.2, 50.0, 34.2, 10.0.

IR (KBr): 3278, 3031, 2969, 2936, 2874, 1502, 1489, 1448, 1409, 1256, 1168, 1013, 830, 806, 741, 701, 671 cm^{-1}

HRMS (ESI) [$C_{25}H_{21}O$] $[M+H]^+$ calculated: 337.1592, found: 337.1605.

4-(2-Phenylbut-3-yn-2-yl)biphenyl (4ab)

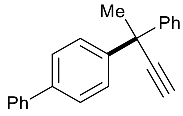

Compound **4ab** was prepared according to the **General procedure F** from **1a** (34.4 mg, 0.2 mmol) and **3b** (78.0 mg, 0.6 mmol) using $Et_3SiBpin$, and purified by silica gel column chromatography (*n*-hexane/DCM: 20/1) as a white solid (39.5 mg, yield: 70%), m.p.: 73.2 – 74.7 °C.

^{1}H NMR (500 MHz, $CDCl_3$) δ 7.60 – 7.54 (m, 2H), 7.54 – 7.46 (m, 6H), 7.45 – 7.38 (m, 2H), 7.35 – 7.29 (m, 3H), 7.26 – 7.21 (m, 1H), 2.58 (s, 1H), 2.01 (s, 3H).

^{13}C NMR (126 MHz, $CDCl_3$) δ 146.0, 145.2, 140.8, 139.6, 128.9, 128.4, 127.5, 127.4, 127.2, 127.1, 127.0, 126.8, 89.7, 72.5, 44.4, 30.6.

IR (KBr): 3281, 3060, 3026, 2983, 2937, 1598, 1487, 1444, 1399, 1214, 1067, 1006, 832, 766, 731, 694, 591 cm^{-1}

HRMS (ESI) [$C_{22}H_{19}$] $[M+H]^+$ calculated: 283.1487, found: 283.1488.

4-(3-Phenyloct-1-yn-3-yl)biphenyl (4ac)

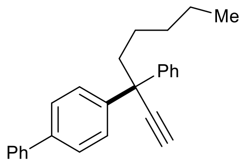

Compound **4ac** was prepared according to the **General procedure F** from **1a** (34.4 mg, 0.2 mmol) and **3c** (112.0 mg, 0.6 mmol) using $Et_3SiBpin$, and purified by silica gel column chromatography (*n*-hexane/DCM: 20/1) as a light-yellow oil (50.6 mg, yield: 75%).

^{1}H NMR (500 MHz, $CDCl_3$) δ 7.59 – 7.54 (m, 2H), 7.53 – 7.45 (m, 6H), 7.40 (dd, *J* = 8.4, 6.9 Hz, 2H), 7.34 – 7.27 (m, 3H), 7.23 – 7.18 (m, 1H), 2.60 (s, 1H), 2.34 – 2.24 (m, 2H), 1.46 – 1.36 (m, 2H), 1.35 – 1.23 (m, 4H), 0.96 – 0.76 (m, 3H).

^{13}C NMR (126 MHz, $CDCl_3$) δ 145.0, 144.3, 140.8, 139.5, 128.9, 128.4, 127.8, 127.4, 127.3, 127.2, 127.0, 126.7, 88.3, 73.9, 49.3, 41.5, 32.2, 25.3, 22.7, 14.2.

IR (KBr): 3304, 3057, 3029, 2953, 2928, 2864, 1699, 1487, 1446, 1279, 1074, 1008, 835, 764, 731, 698, 645 cm^{-1}

HRMS (ESI) [$C_{26}H_{27}N$] $[M+H]^+$ calculated: 339.2113, found: 339.2123.

4-(5-Methyl-3-phenylhex-1-yn-3-yl)biphenyl (4ad)

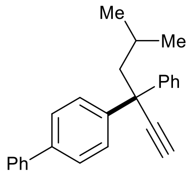

Compound **4ad** was prepared according to the **General procedure F** from **1a** (34.4 mg, 0.2 mmol) and **3d** (103.2 mg, 0.6 mmol) using $Et_3SiBpin$, and purified by silica gel column chromatography (*n*-hexane/DCM: 10/1) as a light-yellow oil (28.0 mg, yield: 43%).

^{1}H NMR (500 MHz, $CDCl_3$) δ 7.60 – 7.54 (m, 2H), 7.55 – 7.48 (m, 5H), 7.50 – 7.47 (m, 1H), 7.41 (t, *J* = 7.4 Hz, 2H), 7.35 – 7.27 (m, 3H), 7.25 – 7.19 (m, 1H), 2.63 (s, 1H), 2.29 (d, *J* = 5.5 Hz, 2H), 1.93 – 1.71 (m, 1H), 0.87 (d, *J* = 6.7 Hz, 3H), 0.83 (d, *J* = 6.6 Hz, 3H).

^{13}C NMR (126 MHz, $CDCl_3$) δ 145.4, 144.8, 140.8, 139.4, 128.9, 128.3, 127.8, 127.5, 127.3, 127.1, 127.0, 126.7, 88.5, 74.6, 49.5, 48.7, 25.9, 24.6.

IR (KBr): 3301, 3059, 3030, 2953, 2867, 1600, 1487, 1446, 1365, 1268, 1007, 836, 764, 698, 648 cm^{-1}

HRMS (EI) [$C_{25}H_{24}$] $[M]^+$ calculated: 324.1878, found: 324.1873.

4-(4-Methyl-3-phenylpent-1-yn-3-yl)biphenyl (4ae)

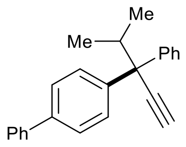

Compound **4ae** was prepared according to the **General procedure F** from **1a** (34.4 mg, 0.2 mmol) and **3e** (95.0 mg, 0.6 mmol) using $Et_3SiBpin$, and purified by silica gel column chromatography (*n*-hexane/DCM: 10/1) as a light-yellow oil (9.1 mg, yield: 14%).

^{1}H NMR (500 MHz, $CDCl_3$) δ 7.66 – 7.59 (m, 4H), 7.57 – 7.52 (m, 2H), 7.52 – 7.48 (m, 2H), 7.40 (dd, *J* = 8.3, 7.0 Hz, 2H), 7.33 – 7.26 (m, 3H), 7.20 – 7.14 (m, 1H), 2.89 (p, *J* = 6.5 Hz, 1H), 2.69 (s, 1H), 1.03 (d, *J* = 6.5 Hz, 3H), 1.00 (d, *J* = 6.5 Hz, 3H).

^{13}C NMR (126 MHz, $CDCl_3$) δ 144.2, 143.5, 140.8, 139.2, 128.8, 128.3, 127.7, 127.3, 127.1, 127.0, 126.5, 85.4, 75.7, 55.5, 35.1, 19.1.

IR (KBr): 3302, 3057, 3030, 2966, 2932, 2874, 1599, 1487, 1446, 1385, 1264, 1007, 829, 761, 699, 636cm^{-1}

HRMS (EI) [$C_{24}H_{22}$] $[M]^+$ calculated: 310.1722, found: 310.1725.

4-(3-(2-(Trifluoromethoxy)phenyl)pent-1-yn-3-yl)biphenyl (4af)

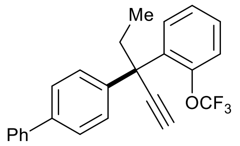

Compound **4af** was prepared according to the **General procedure F** from **1a** (34.4 mg, 0.2 mmol) and **3f** (136.9 mg, 0.6 mmol) using $PhMe_2SiBpin$, and purified by silica gel column chromatography (*n*-hexane/DCM: 10/1) as a light-yellow oil (13.6 mg, yield: 18%).

^{1}H NMR (700 MHz, $CDCl_3$) δ 7.91 (dd, *J* = 7.4, 2.2 Hz, 1H), 7.59 – 7.56 (m, 2H), 7.52 – 7.48 (m, 2H), 7.43 – 7.37 (m, 4H), 7.34 – 7.27 (m, 3H), 7.21 – 7.17 (m, 1H), 2.59 (s, 1H), 2.58 – 2.48 (m, 1H), 2.38 – 2.28 (m, 1H), 0.95 (t, *J* = 7.2 Hz, 3H).

^{13}C NMR (176 MHz, $CDCl_3$) δ 148.0, 143.0, 141.0, 139.3, 135.2, 129.3, 128.9, 128.8, 127.4, 127.3, 127.2, 126.8, 125.7, 120.3 (q, *J* = 258.7 Hz), 119.0, 86.5, 74.0, 47.8, 32.7, 9.8.

^{19}F NMR (282 MHz, $CDCl_3$) δ -55.88 (s, 3F).

IR (KBr): 3304, 3060, 3032, 2972, 2936, 2878, 1905, 1602, 1486, 1448, 1266, 1204, 1146, 1058, 1008, 922, 836, 761, 634 cm^{-1}

HRMS (ESI) [$C_{24}H_{20}F_3O$] $[M+H]^+$ calculated: 381.1466, found: 381.1469.

4-(3-(4-Methoxyphenyl)pent-1-yn-3-yl)biphenyl (4ag)

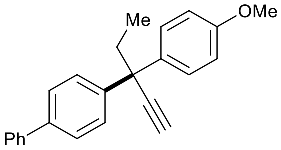

Compound **4ag** was prepared according to the **General procedure F** from **1a** (34.4 mg, 0.2 mmol) and **3g** (104.4 mg, 0.6 mmol) using $PhMe_2SiBpin$, and purified by silica gel column chromatography (*n*-hexane/DCM: 5/1) as a colorless oil (60.7 mg, yield: 93%).

^{1}H NMR (300 MHz, $CDCl_3$) δ 7.64 – 7.57 (m, 2H), 7.60 – 7.47 (m, 4H), 7.50 – 7.37 (m, 4H), 7.40 – 7.28 (m, 1H), 6.88 (d, *J* = 8.9 Hz, 2H), 3.81 (s, 3H), 2.62 (s, 1H), 2.35 (q, *J* = 7.3 Hz, 2H), 1.02 (t, *J* = 7.2 Hz, 3H).

^{13}C NMR (126 MHz, $CDCl_3$) δ 158.2, 144.3, 140.8, 139.4, 136.9, 128.8, 128.5, 127.8, 127.3, 127.1, 127.0, 113.6, 88.2, 73.8, 55.3, 49.2, 34.5, 10.1.

IR (KBr): 3295, 3031, 2971, 2934, 2877, 2835, 1607, 1583, 1506, 1486, 1286, 1181, 1036, 1008, 836, 764, 698, 638 cm^{-1}

HRMS (ESI) [$C_{24}H_{23}O$] $[M+H]^+$ calculated: 327.1749, found: 327.1745.

4-(3-(3-Methoxyphenyl)pent-1-yn-3-yl)biphenyl (4ah)

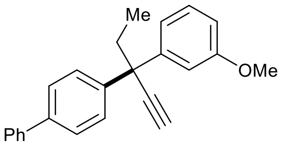

Compound **4ah** was prepared according to the **General procedure F** from **1a** (34.4 mg, 0.2 mmol) and **3h** (104.4 mg, 0.6 mmol) using $PhMe_2SiBpin$, and purified by silica gel column chromatography (*n*-hexane/DCM: 5/1) as a colorless oil (52.2 mg, yield: 80%).

^{1}H NMR (500 MHz, $CDCl_3$) δ 7.59 – 7.53 (m, 2H), 7.51 (br-s, 4H), 7.44 – 7.37 (m, 2H), 7.35 – 7.28 (m, 1H), 7.25 – 7.19 (m, 1H), 7.11 – 7.06 (m, 1H), 7.08 – 7.02 (m, 1H), 6.83 – 6.69 (m, 1H), 3.77 (s, 3H), 2.60 (s, 1H), 2.34 (q, *J* = 7.2 Hz, 2H), 1.00 (t, *J* = 7.2 Hz, 3H).

^{13}C NMR (126 MHz, $CDCl_3$) δ 159.5, 146.4, 143.9, 140.8, 139.5, 129.3, 128.8, 127.8, 127.3, 127.1, 127.0, 119.9, 114.1, 111.4, 87.8, 74.0, 55.3, 49.9, 34.3, 10.1.

IR (KBr): 3290, 3030, 2971, 2035, 2878, 2834, 1601, 1485, 1432, 1290, 1254, 1166, 1048, 841, 764, 698, 640 cm^{-1}

HRMS (ESI) [$C_{24}H_{23}O$] $[M+H]^+$ calculated: 327.1749, found: 327.1752.

4-(3-(*p*-Tolyl)pent-1-yn-3-yl)biphenyl (4ai)

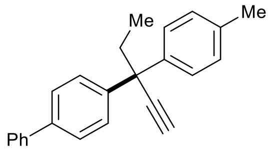

Compound **4ai** was prepared according to the **General procedure F** from **1a** (34.4 mg, 0.2 mmol) and **3i** (94.8 mg, 0.6 mmol) using $Et_3SiBpin$, and purified by silica gel column chromatography (*n*-hexane/DCM: 9/1) as a white solid (55.7 mg, yield: 90%), m.p.: 84.2 – 85.9 °C.

^{1}**H NMR** (500 MHz, $CDCl_3$) δ 7.58 – 7.54 (m, 2H), 7.53 – 7.47 (m, 4H), 7.40 (dd, *J* = 8.4, 7.0 Hz, 2H), 7.38 – 7.34 (m, 2H), 7.33 – 7.28 (m, 1H), 7.11 (d, *J* = 8.0 Hz, 2H), 2.58 (s, 1H), 2.33 (q, *J* = 7.3 Hz, 2H), 2.31 (s, 3H), 0.99 (t, *J* = 7.2 Hz, 3H).

^{13}**C NMR** (126 MHz, $CDCl_3$) δ 144.2, 141.8, 140.8, 139.4, 136.3, 129.1, 128.8, 127.8, 127.3, 127.1, 127.0, 88.1, 73.8, 49.6, 34.3, 21.1, 10.1.

IR (KBr): 3277, 3052, 3029, 2971, 2933, 2872, 1597, 1508, 1486, 1458, 1279, 1005, 834, 811, 763, 726, 688, 639, 503 cm^{-1}

HRMS (ESI) [$C_{24}H_{23}$] $[M+H]^+$ calculated: 311.1800, found: 311.1803.

4-(3-(*m*-Tolyl)pent-1-yn-3-yl)biphenyl (4aj)

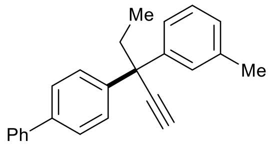

Compound **4aj** was prepared according to the **General procedure F** from **1a** (34.4 mg, 0.2 mmol) and **3j** (94.8 mg, 0.6 mmol) using $Et_3SiBpin$, and purified by silica gel column chromatography (*n*-hexane/DCM: 9/1) as a white solid (51.2 mg, yield: 82%), m.p.: 76.8 – 78.3 °C.

^{1}**H NMR** (500 MHz, $CDCl_3$) δ 7.59 – 7.54 (m, 2H), 7.53 – 7.47 (m, 4H), 7.44 – 7.37 (m, 2H), 7.34 – 7.29 (m, 2H), 7.28 – 7.24 (m, 1H), 7.22 – 7.17 (m, 1H), 7.05 – 7.01 (m, 1H), 2.60 (s, 1H), 2.34 (q, *J* = 7.3 Hz, 2H), 2.33 (s, 3H), 0.99 (t, *J* = 7.2 Hz, 3H).

^{13}**C NMR** (126 MHz, $CDCl_3$) δ 144.6, 144.2, 140.8, 139.4, 137.9, 128.9, 128.2, 127.8, 127.5, 127.3, 127.2, 127.0, 124.5, 88.1, 73.9, 49.8, 34.3, 21.8, 10.1.

IR (KBr): 3274, 3023, 2970, 2935, 2877, 1602, 1483, 1445, 1376, 1293, 1007, 822, 761, 697, 652 cm^{-1}

HRMS (ESI) [$C_{24}H_{23}$] $[M+H]^+$ calculated: 311.1800, found: 311.1801.

4-(3-(3,5-Dimethylphenyl)pent-1-yn-3-yl) biphenyl (4ak)

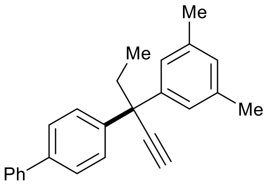

Compound **4ak** was prepared according to the **General procedure F** from **1a** (34.4 mg, 0.2 mmol) and **3k** (103.2 mg, 0.6 mmol) using $Et_3SiBpin$, and purified by silica gel column chromatography (*n*-hexane/DCM: 10/1) as a white solid (52.5 mg, yield: 81%), m.p.: 94.8 – 96.2 °C.

^{1}H NMR (500 MHz, $CDCl_3$) δ 7.59 – 7.55 (m, 2H), 7.53 – 7.48 (m, 4H), 7.41 (t, *J* = 7.7 Hz, 2H), 7.35 – 7.27 (m, 1H), 7.08 (s, 2H), 6.86 (s, 1H), 2.59 (s, 1H), 2.33 (q, *J* = 7.3 Hz, 2H), 2.28 (s, 6H), 0.99 (t, *J* = 7.2 Hz, 3H).

^{13}C NMR (126 MHz, $CDCl_3$) δ 144.6, 144.2, 140.8, 139.4, 137.9, 128.9, 128.2, 127.8, 127.5, 127.2, 127.0, 124.5, 88.1, 73.9, 49.8, 34.3, 21.8, 10.1.

IR (KBr): 3279, 3025, 2976, 2940, 2926, 1599, 1483, 1443, 1307, 1007, 850, 811, 760, 742, 698, 659 cm^{-1}

HRMS (ESI) [$C_{25}H_{25}$] $[M+H]^+$ calculated: 325.1956, found: 325.1953.

4-(3-(4-Chlorophenyl)pent-1-yn-3-yl)biphenyl (4al)

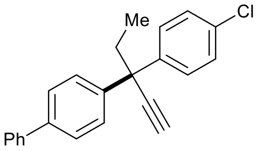

Compound **4al** was prepared according to the **General procedure F** from **1a** (34.4 mg, 0.2 mmol) and **3l** (107 mg, 0.6 mmol) using $Et_3SiBpin$, and purified by silica gel column chromatography (*n*-hexane/DCM: 15/1) as a light-yellow oil (27.5 mg, yield: 41%).

^{1}H NMR (500 MHz, $CDCl_3$) δ 7.58 – 7.55 (m, 2H), 7.54 – 7.51 (m, 2H), 7.49 – 7.46 (m, 2H), 7.45 – 7.39 (m, 4H), 7.36 – 7.31 (m, 1H), 7.30 – 7.26 (m, 2H), 2.62 (s, 1H), 2.42 – 2.21 (m, 2H), 0.99 (t, *J* = 7.2 Hz, 3H).

^{13}C NMR (126 MHz, $CDCl_3$) δ 143.6 , 143.4, 140.7, 139.7, 132.6, 128.9, 128.5, 127.8, 127.4, 127.2, 87.4, 74.3, 49.5, 34.3, 10.0.

IR (KBr): 3300, 3057, 3029, 2972, 2935, 2876, 1601, 1487, 1401, 1267, 1094, 1013, 835, 766, 699, 638 cm^{-1}

HRMS (ESI) [$C_{23}H_{20}Cl$] $[M+H]^+$ calculated: 331.1254, found: 331.1257.

2-(3-(Biphenyl-4-yl)pent-1-yn-3-yl)naphthalene (4am)

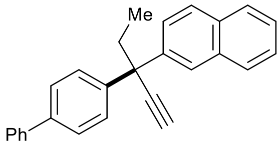

Compound **4am** was prepared according to the **General procedure F** from **1a** (34.4 mg, 0.2 mmol) and **3m** (116.4 mg, 0.6 mmol) using $Et_3SiBpin$, and purified by silica gel column chromatography (*n*-hexane/DCM: 15/1) as a white solid (43.7 mg, yield: 63%), m.p.: 103.5 – 104.4 °C.

^{1}H NMR (500 MHz, $CDCl_3$) δ 8.07 (d, J = 1.9 Hz, 1H), 7.86 (d, J = 7.6 Hz, 1H), 7.80 – 7.76 (m, 1H), 7.74 (d, J = 8.7 Hz, 1H), 7.58 – 7.54 (m, 2H), 7.52 (s, 4H), 7.50 – 7.42 (m, 3H), 7.40 (t, J = 7.2 Hz, 2H), 7.35 – 7.29 (m, 1H), 2.66 (s, 1H), 2.46 (qd, J = 7.3, 4.9 Hz, 2H), 1.02 (t, J = 7.2 Hz, 3H).

^{13}C NMR (126 MHz, $CDCl_3$) δ 143.9, 142.0, 140.8, 139.6, 133.2, 132.3, 128.9, 128.3, 128.2, 128.0, 127.6, 127.3, 127.2, 127.1, 126.3, 126.2, 126.0, 125.5, 87.8, 74.3, 50.0, 34.0, 10.1.

IR (KBr): 3279, 3053, 3024, 2966, 2929, 2874, 1599, 1483, 1273, 1006, 834, 819, 756, 699, 640 cm^{-1}

HRMS (ESI) [$C_{27}H_{23}$] $[M+H]^+$ calculated: 347.1800, found: 347.1805.

(3-(Biphenyl-4-yl)-3-phenylpent-1-yn-1-yl)triethylsilane (4an)

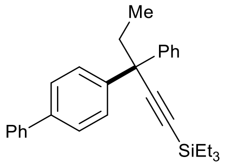

Compound **4an** was prepared according to the **General procedure F** from **1a** (34.4 mg, 0.2 mmol) and **3n** (154.8 mg, 0.6 mmol) using $Et_3SiBpin$, and purified by silica gel column chromatography (*n*-hexane/DCM: 10/1) as a light-yellow oil (30.1 mg, yield: 36%).

^{1}H NMR (500 MHz, $CDCl_3$) δ 7.59 – 7.56 (m, 2H), 7.55 – 7.49 (m, 6H), 7.41 (t, J = 7.7 Hz, 2H), 7.35 – 7.27 (m, 3H), 7.23 – 7.18 (m, 1H), 2.32 (q, J = 7.2 Hz, 2H), 1.05 (t, J = 7.9 Hz, 9H), 1.01 (t, J = 7.3 Hz, 3H), 0.67 (q, J = 7.9 Hz, 6H).

^{13}C NMR (126 MHz, $CDCl_3$) δ 145.1, 144.4, 140.9, 139.2, 128.8, 128.2, 127.9, 127.5, 127.3, 127.1, 126.9, 126.5, 111.2, 87.5, 51.1, 34.6, 10.2, 7.8, 4.8.

IR (KBr): 3059, 3030, 2955, 2875, 2162, 1600, 1487, 1448, 1414, 1235, 1196, 1007, 834, 767, 697 cm^{-1}

HRMS (ESI) [$C_{29}H_{35}Si$] $[M+H]^+$ calculated: 411.2508, found: 411.2511.

3-(3-(4-Methoxyphenyl)pent-1-yn-3-yl)biphenyl (4bg)

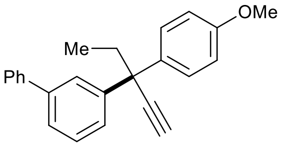

Compound **4bg** was prepared according to the **General procedure F** from **1b** (34.4 mg, 0.2 mmol) and **3g** (104.4 mg, 0.6 mmol) using $PhMe_2SiBpin$, and purified by silica gel column chromatography (*n*-hexane/DCM: 5/1) as a colorless oil (56.0 mg, yield: 86%).

^{1}H NMR (300 MHz, $CDCl_3$) δ 7.76 – 7.65 (m, 1H), 7.61 – 7.54 (m, 2H), 7.49 – 7.31 (m, 8H), 6.95 – 6.78 (m, 2H), 3.80 (s, 3H), 2.62 (s, 1H), 2.37 (q, J = 7.2 Hz, 2H), 1.02 (t, J = 7.2 Hz, 3H).

^{13}C NMR (126 MHz, $CDCl_3$) δ 158.2, 145.7, 141.4, 141.1, 137.0, 128.8, 128.7, 128.5, 127.4, 126.4, 126.3, 125.5, 113.6, 88.2, 73.7, 55.3, 49.5, 34.5, 10.1.

IR (KBr): 3297, 3033, 2971, 2935, 2876, 2836, 1608, 1578, 1507, 1477, 1411, 1253, 1182, 1035, 830, 759, 703, 632 cm^{-1}

HRMS (ESI) [$C_{24}H_{23}O$] $[M+H]^+$ calculated: 327.1749, found: 327.1746.

1-(3-(4-Methoxyphenyl)pent-1-yn-3-yl)-3-phenoxybenzene (4dg)

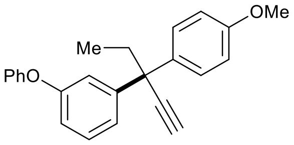

Compound **4dg** was prepared according to the **General procedure F** from **1d** (38.0 mg, 0.2 mmol) and **3g** (104.4 mg, 0.6 mmol) using $PhMe_2SiBpin$, and purified by silica gel column chromatography (*n*-hexane/DCM: 4/1) as a colorless oil (60.9 mg, yield: 89%).

^{1}H NMR (500 MHz, $CDCl_3$) δ 7.40 – 7.35 (m, 2H), 7.35 – 7.30 (m, 2H), 7.25 (t, J = 7.9 Hz, 1H), 7.22 (t, J = 2.1 Hz, 1H), 7.19 (ddd, J = 7.8, 1.8, 1.1 Hz, 1H), 7.10 (tt, J = 7.4, 1.2 Hz, 1H), 7.02 – 6.98 (m, 2H), 6.88 – 6.81 (m, 3H), 3.80 (s, 3H), 2.58 (s, 1H), 2.29 (qd, J = 7.3, 2.6 Hz, 2H), 0.99 (t, J = 7.3 Hz, 3H).

^{13}C NMR (126 MHz, $CDCl_3$) δ 158.3, 157.4, 156.9, 147.4, 136.7, 129.8, 129.4, 128.4, 123.2, 122.5, 118.7, 118.6, 116.8, 113.6, 87.9, 73.9, 55.3, 49.4, 34.4, 10.0.

IR (KBr): 3290, 3063, 2967, 2934, 2835, 1607, 1579, 1508, 1436, 1249, 1180, 1035, 861, 826, 694, 640 cm^{-1}

HRMS (ESI) [$C_{24}H_{23}O_2$] $[M+H]^+$ calculated: 343.1698, found: 343.1695.

1-Phenoxy-3-(3-(p-tolyl)pent-1-yn-3-yl)benzene (4di)

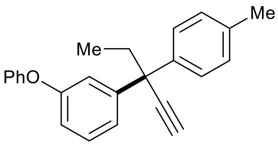

Compound **4di** was prepared according to the **General procedure F** from **1d** (38.0 mg, 0.2 mmol) and **3i** (94.8 mg, 0.6 mmol) using $PhMe_2SiBpin$, and purified by silica gel column chromatography (*n*-hexane/DCM: 5/1) as a colorless oil (59.1 mg, yield: 91%).

^{1}H NMR (500 MHz, $CDCl_3$) δ 7.35 – 7.26 (m, 4H), 7.24 – 7.18 (m, 2H), 7.16 (dt, *J* = 7.9, 1.4 Hz, 1H), 7.09 (d, *J* = 7.9 Hz, 2H), 7.06 (tt, *J* = 7.3, 1.1 Hz, 1H), 6.99 – 6.94 (m, 2H), 6.79 (ddd, *J* = 7.8, 2.5, 1.1 Hz, 1H), 2.53 (s, 1H), 2.30 (s, 3H), 2.29 – 2.21 (m, 2H), 0.96 (t, *J* = 7.3 Hz, 3H).

^{13}C NMR (126 MHz, $CDCl_3$) δ 157.3, 156.9, 147.3, 141.6, 136.3, 129.8, 129.5, 129.1, 127.2, 123.1, 122.6, 118.6, 116.8, 87.8, 73.9, 49.7, 34.2, 21.1, 10.0.

IR (KBr): 3298, 3028, 2972, 2936, 2877, 1582, 1486, 1433, 1246, 1163, 1022, 861, 813, 753, 694, 639 cm^{-1}

HRMS (ESI) [$C_{24}H_{23}O$] $[M+H]^+$ calculated: 327.1749, found: 327.1751.

1,3-Dimethyl-5-(3-(4-phenoxyphenyl)pent-1-yn-3-yl)benzene (4ek)

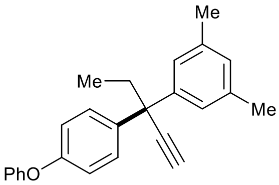

Compound **4ek** was prepared according to the **General procedure F** from **1e** (38.0 mg, 0.2 mmol) and **3k** (103.2 mg, 0.6 mmol) using $PhMe_2SiBpin$, and purified by silica gel column chromatography (*n*-hexane/DCM: 5/1) as a colorless oil (58.5 mg, yield: 86%).

^{1}H NMR (500 MHz, $CDCl_3$) δ 7.41 – 7.36 (m, 2H), 7.34 – 7.28 (m, 2H), 7.08 (tt, *J* = 7.3, 1.1 Hz, 1H), 7.05 (br-s, 2H), 7.03 – 6.98 (m, 2H), 6.95 – 6.89 (m, 2H), 6.85 (br-s, 1H), 2.57 (s, 1H), 2.28 (s, 6H), 2.28 – 2.23 (m, 2H), 0.96 (t, *J* = 7.2 Hz, 3H).

^{13}C NMR (126 MHz, $CDCl_3$) δ 157.2, 155.7, 144.6, 140.0, 137.7, 129.8, 128.7, 128.4, 125.2, 123.3, 119.1, 118.4, 88.3, 73.8, 49.4, 34.5, 21.7, 10.1.

IR (KBr): 3297, 3036, 2972, 2935, 2877, 1588, 1488, 1245, 1171, 1016, 870, 846, 693, 637 cm^{-1}

HRMS (ESI) [$C_{25}H_{25}O$] $[M+H]^+$ calculated: 341.1905, found: 341.1907.

4-Methyl-4'-(3-phenyloct-1-yn-3-yl)biphenyl (4gc)

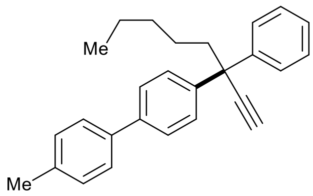

Compound **4gc** was prepared according to the **General procedure F** from **1g** (37.2 mg, 0.2 mmol) and **3c** (112.0 mg, 0.6 mmol) using $Et_3SiBpin$, and purified by silica gel column chromatography (*n*-hexane/DCM: 10/1) as a white solid (41.7 mg, yield: 60%), m.p.: 70.8 – 72.2 °C.

^{1}H NMR (500 MHz, $CDCl_3$) δ 7.53 – 7.43 (m, 8H), 7.31 (t, *J* = 7.8 Hz, 2H), 7.25 – 7.19 (m, 3H), 2.60 (s, 1H), 2.37 (s, 3H), 2.31 – 2.24 (m, 2H), 1.46 – 1.33 (m, 2H), 1.35 – 1.22 (m, 4H), 0.85 (t, *J* = 6.9 Hz, 3H).

^{13}C NMR (126 MHz, $CDCl_3$) δ 145.0, 144.0, 139.4, 137.9, 137.1, 129.6, 128.3, 127.7, 127.4, 127.0, 126.83 , 126.7, 88.3, 73.9, 49.3, 41.5, 32.2, 25.3, 22.7, 21.3, 14.2.

IR (KBr): 3297, 3025, 2938, 2925, 2858, 1598, 1496, 1466, 1445, 1373, 806, 768, 704, 642 cm^{-1}

HRMS (ESI) [$C_{27}H_{29}$] $[M+H]^+$ calculated: 353.2269, found: 353.2263.

4-Methoxy-4'-(3-(4-methoxyphenyl)pent-1-yn-3-yl)biphenyl (4hg)

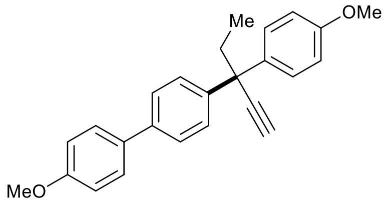

Compound **4hg** was prepared according to the **General procedure F** from **1h** (40.4 mg, 0.2 mmol) and **3g** (104.4 mg, 0.6 mmol) using $PhMe_2SiBpin$, and purified by silica gel column chromatography (*n*-hexane/DCM: 3/1) as a white solid (67.0 mg, yield: 94%), m.p.: 75.7 – 76.4 °C.

^{1}H NMR (500 MHz, $CDCl_3$) δ 7.54 – 7.48 (m, 2H), 7.46 (s, 4H), 7.42 – 7.30 (m, 2H), 7.01 – 6.89 (m, 2H), 6.89 – 6.68 (m, 2H), 3.82 (s, 3H), 3.77 (s, 3H), 2.58 (s, 1H), 2.31 (q, *J* = 7.2 Hz, 2H), 0.98 (t, *J* = 7.2 Hz, 3H).

^{13}C NMR (126 MHz, $CDCl_3$) δ 159.1, 158.2, 143.7, 139.0, 137.0 , 133.3, 128.5, 128.1, 127.7, 126.5, 114.2, 113.6, 88.2, 73.7, 55.4, 55.3, 49.2, 34.4, 10.1.

IR (KBr): 3294, 2967, 2932, 2833, 1606, 1579, 1496, 1449, 1281, 1249, 1182, 1036, 823, 637 cm^{-1}

HRMS (ESI) [$C_{25}H_{25}O_2$] $[M+H]^+$ calculated: 357.1855, found: 357.1840.

4-Methoxy-4'-(3-(3-methoxyphenyl)pent-1-yn-3-yl)biphenyl (4hh)

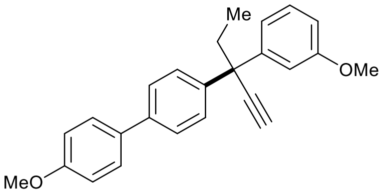

Compound **4hh** was prepared according to the **General procedure F** from **1h** (40.4 mg, 0.2 mmol) and **3h** (104.4 mg, 0.6 mmol) using $PhMe_2SiBpin$, and purified by silica gel column chromatography (*n*-hexane/DCM: 3/1) as a light-yellow oil (54.7 mg, yield: 77%).

^{1}H NMR (500 MHz, $CDCl_3$) δ 7.52 – 7.48 (m, 2H), 7.47 (s, 4H), 7.22 (t, J = 8.0 Hz, 1H), 7.10 – 7.07 (m, 1H), 7.07 – 7.01 (m, 1H), 6.98 – 6.91 (m, 2H), 6.80 – 6.72 (m, 1H), 3.82 (s, 3H), 3.77 (s, 3H), 2.60 (s, 1H), 2.33 (q, J = 7.2 Hz, 2H), 0.99 (t, J = 7.2 Hz, 3H).

^{13}C NMR (126 MHz, $CDCl_3$) δ 159.5 , 159.1, 146.4, 143.3, 139.1, 133.3, 129.2, 128.1, 127.7, 126.6, 119.9, 114.2, 114.1, 111.3, 87.9, 74.0, 55.4, 55.3, 49.9, 34.2, 10.1.

IR (KBr): 3271, 3005, 2975, 2935, 2836, 1607, 1495, 1435, 1291, 1247, 1177, 1048, 822, 782, 701, 641, 504 cm^{-1}

HRMS (ESI) [$C_{25}H_{25}O_2$] $[M+H]^+$ calculated: 357.1855, found: 357.1856.

4-Chloro-4'-(3-(3-methoxyphenyl)pent-1-yn-3-yl)biphenyl (4kh)

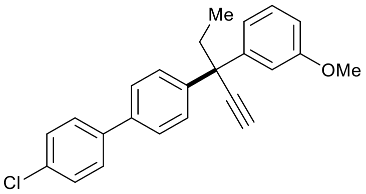

Compound **4kh** was prepared according to the **General procedure F** from **1k** (41.2 mg, 0.2 mmol) and **3h** (104.4 mg, 0.6 mmol) using $PhMe_2SiBpin$, and purified by silica gel column chromatography (*n*-hexane/DCM: 5/1) as a light-yellow oil (52.6 mg, yield: 73%).

^{1}H NMR (500 MHz, $CDCl_3$) δ 7.54 – 7.43 (m, 6H), 7.39 – 7.35 (m, 2H), 7.23 (t, J = 7.2 Hz, 1H), 7.07 (t, J = 2.2 Hz, 1H), 7.05 – 7.02 (m, 1H), 6.76 (dd, J = 8.2, 2.5 Hz, 1H), 3.77 (s, 3H), 2.61 (s, 1H), 2.33 (q, J = 7.2 Hz, 2H), 0.99 (t, J = 7.2 Hz, 3H).

^{13}C NMR (126 MHz, $CDCl_3$) δ 159.6, 146.3, 144.4, 139.3, 138.3, 133.4, 129.3, 129.0, 128.4, 127.9, 126.9, 119.9, 114.2, 111.4, 87.7, 74.1, 55.3, 49.9, 34.2, 10.1.

IR (KBr): 3296, 3028, 2972, 2834, 1603, 1485, 1433, 1290, 1250, 1166, 1093, 1047, 1005, 851, 813, 738, 700, 651 cm^{-1}

HRMS (ESI) [$C_{24}H_{21}ONaCl$] $[M+Na]^+$ calculated: 383.1179, found: 383.1186.

4-(3-(4-Methoxyphenyl)pent-1-yn-3-yl)-4'-(trifluoromethyl)-biphenyl (4lg)

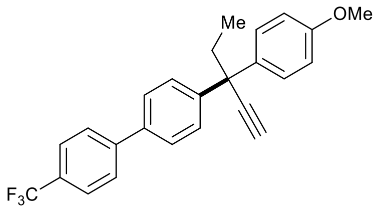

Compound **4lg** was prepared according to the **General procedure F** from **1l** (48.0 mg, 0.2 mmol) and **3g** (104.4 mg, 0.6 mmol) using $Et_3SiBpin$, and purified by silica gel column chromatography (*n*-hexane/DCM: 5/1) as a light-yellow oil (49.0 mg, yield: 62%).

^{1}H NMR (700 MHz, $CDCl_3$) δ 7.75 – 7.62 (m, 4H), 7.58 – 7.50 (m, 4H), 7.48 – 7.35 (m, 2H), 6.95 – 6.81 (m, 2H), 3.80 (s, 3H), 2.63 (s, 1H), 2.35 (q, *J* = 7.2 Hz, 2H), 1.02 (t, *J* = 7.2 Hz, 3H).

^{13}C NMR (176 MHz, $CDCl_3$) δ 158.4, 145.5, 144.4, 137.9, 136.7, 129.4 (q, *J* = 32.0 Hz), 128.5, 128.0, 127.4, 127.2, 125.8 (q, *J* = 4.4 Hz), 124.4 (q, *J* = 271.1 Hz), 113.7, 88.0, 73.9, 55.4, 49.3, 34.4, 10.1.

^{19}F NMR (282 MHz, $CDCl_3$) δ -62.86 (s, 3F).

IR (KBr): 3304, 3034, 2971, 2935, 2878, 1615, 1506, 1464, 1328, 1252, 1179, 1128, 1070, 1034, 822, 639 cm^{-1}

HRMS (ESI) [$C_{25}H_{22}F_3O$] $[M+H]^+$ calculated: 395.1623, found: 395.1627.

4-(4-(3-Phenyloct-1-yn-3-yl)phenyl)pyridine (4nc)

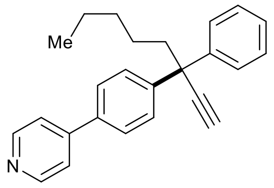

Compound **4nc** was prepared according to the **General procedure F** from **1n** (34.6 mg, 0.2 mmol) and **3c** (112.0 mg, 0.6 mmol) using $PhMe_2SiBpin$, and purified by silica gel column chromatography (DCM) as a light-yellow solid (50.3 mg, yield: 74%), m.p.: 88.6 – 90.0 °C.

^{1}H NMR (500 MHz, $CDCl_3$) δ 8.74 – 8.54 (m, 2H), 7.56 (s, 4H), 7.50 – 7.44 (m, 4H), 7.35 – 7.28 (m, 2H), 7.24 – 7.20 (m, 1H), 2.63 (s, 1H), 2.48 – 2.15 (m, 2H), 1.44 – 1.37 (m, 2H), 1.35 – 1.27 (m, 4H), 0.93 – 0.69 (m, 3H).

^{13}C NMR (126 MHz, $CDCl_3$) δ 150.3, 147.9, 146.4, 144.6, 136.3, 128.4, 128.1, 127.3, 126.9, 126.8, 121.6, 87.9, 74.2, 49.3, 41.3, 32.1, 25.2, 22.6, 14.2.

IR (KBr): 3299, 3184, 3060, 3032, 2952, 2869, 1595, 1542, 1490, 1445, 1403, 1221, 993, 809, 700, 645 cm^{-1}

HRMS (ESI) [$C_{25}H_{26}N$] $[M+H]^+$ calculated: 340.2065, found: 340.2055.

2-Phenyl-5-(3-(*p*-tolyl)pent-1-yn-3-yl)pyridine (4oi)

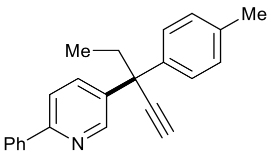

Compound **4oi** was prepared according to the **General procedure F** from **1o** (34.6 mg, 0.2 mmol) and **3i** (94.8 mg, 0.6 mmol) using $PhMe_2SiBpin$, and purified by silica gel column chromatography (*n*-hexane/DCM: 1/1) as a light-yellow oil (42.0 mg, yield: 67%).

^{1}H NMR (300 MHz, $CDCl_3$) δ 8.80 (dd, *J* = 2.5, 0.8 Hz, 1H), 8.02 – 7.93 (m, 2H), 7.77 (dd, *J* = 8.4, 2.5 Hz, 1H), 7.65 (dd, *J* = 8.4, 0.9 Hz, 1H), 7.52 – 7.38 (m, 3H), 7.41 – 7.31 (m, 2H), 7.19 – 7.08 (m, 2H), 2.64 (s, 1H), 2.36 (q, *J* = 7.6 Hz, 2H), 2.33 (s, 3H), 1.01 (t, *J* = 7.2 Hz, 3H).

^{13}C NMR (75 MHz, $CDCl_3$) δ 155.6, 148.7, 140.8, 139.1, 139.0, 136.7, 136.0, 129.3, 129.0, 128.8, 127.3, 126.9, 120.0, 86.9, 74.5, 48.1, 34.1, 21.1, 10.0.

IR (KBr): 3301, 3028, 2970, 2932, 2878, 1592, 1558, 1508, 1473, 1443, 1376, 1272, 1190, 1017, 920, 840, 812, 740, 640 cm^{-1}

HRMS (ESI) [$C_{23}H_{22}N$] $[M+H]^+$ calculated: 312.1752, found: 312.1755.

1-(4-(3-(4-Methoxyphenyl)pent-1-yn-3-yl)phenyl)-1*H*-pyrrole (4qg)

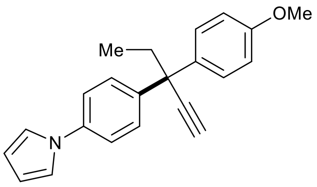

Compound **4qg** was prepared according to the **General procedure F** from **1q** (32.2 mg, 0.2 mmol) and **3g** (104.4 mg, 0.6 mmol) using $PhMe_2SiBpin$, and purified by silica gel column chromatography (*n*-hexane/DCM: 4/1) as a light-yellow oil (50.0 mg, yield: 79%).

^{1}H NMR (500 MHz, $CDCl_3$) δ 7.48 – 7.44 (m, 2H), 7.38 – 7.34 (m, 2H), 7.33 – 7.28 (m, 2H), 7.05 (t, *J* = 2.2 Hz, 2H), 6.90 – 6.77 (m, 2H), 6.32 (t, *J* = 2.2 Hz, 2H), 3.78 (s, 3H), 2.59 (s, 1H), 2.29 (q, *J* = 7.3, 6.6 Hz, 2H), 0.97 (t, *J* = 7.2 Hz, 3H).

^{13}C NMR (126 MHz, $CDCl_3$) δ 158.3, 142.7, 139.2, 136.7, 128.5, 128.4, 120.3, 119.4, 113.7, 110.4, 88.0, 73.9, 55.3, 49.0, 34.5, 10.0.

IR (KBr): 3295, 3043, 2969, 2935, 2877, 2837, 1606, 1505, 1464, 1331, 1251, 1181, 1119, 1070, 924, 834, 724 cm^{-1}

HRMS (ESI) [$C_{22}H_{22}NO$] $[M+H]^+$ calculated: 316.1701, found: 316.1701.

2-(4-(3-(3,5-Dimethylphenyl)pent-1-yn-3-yl)phenyl)-1-methyl-1*H*-indole (4rk)

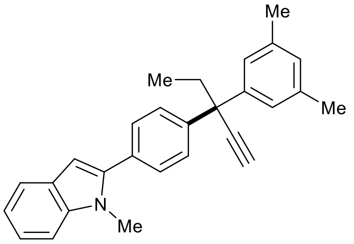

Compound **4rk** was prepared according to the **General procedure F** from **1r** (45.0 mg, 0.2 mmol) and **3k** (103.2 mg, 0.6 mmol) using $PhMe_2SiBpin$, and purified by silica gel column chromatography (*n*-hexane/DCM: 5/1) as a light-yellow solid (66.0 mg, yield: 87%).

^{1}H NMR (500 MHz, $CDCl_3$) δ 7.62 (d, *J* = 7.7 Hz, 1H), 7.55 – 7.50 (m, 2H), 7.45 – 7.40 (m, 2H), 7.33 (d, *J* = 8.2 Hz, 1H), 7.23 (ddd, *J* = 8.2, 7.0, 1.2 Hz, 1H), 7.15 – 7.07 (m, 3H), 6.87 (s, 1H), 6.53 (s, 1H), 3.72 (s, 3H), 2.61 (s, 1H), 2.34 (qd, *J* = 7.3, 1.8 Hz, 2H), 2.30 (s, 6H), 1.00 (t, *J* = 7.2 Hz, 3H).

^{13}C NMR (126 MHz, $CDCl_3$) δ 144.9, 144.2, 141.4, 138.4, 137.8, 131.0, 129.2, 128.6, 128.0, 127.5, 125.3, 121.7, 120.5, 119.9, 109.7, 101.7, 88.0, 74.0, 49.9, 34.3, 31.4, 21.7, 10.1.

IR (KBr): 3285, 3033, 2967, 2935, 2873, 2781, 1597, 1465, 1409, 1338, 1319, 1287, 1129, 1007, 851, 741, 705, 649cm^{-1}

HRMS (ESI) [$C_{28}H_{28}N$] $[M+H]^+$ calculated: 378.2222, found: 378.2227.

2-(3-(*p*-Tolyl)pent-1-yn-3-yl)benzofuran (4ui)

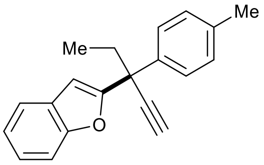

Compound **4ui** was prepared according to the **General procedure F** from **1u** (27.2 mg, 0.2 mmol) and **3i** (94.8 mg, 0.6 mmol) using $Et_3SiBpin$, and purified by silica gel column chromatography (*n*-hexane/DCM: 15/1) as a light-yellow oil (28.1 mg, yield: 51%).

^{1}H NMR (700 MHz, $CDCl_3$) δ 7.52 (d, *J* = 7.6 Hz, 1H), 7.45 (d, *J* = 8.2 Hz, 2H), 7.42 (d, *J* = 8.1 Hz, 1H), 7.25 – 7.18 (m, 2H), 7.14 (d, *J* = 8.2 Hz, 2H), 6.74 (s, 1H), 2.61 (s, 1H), 2.53 – 2.45 (m, 1H), 2.33 (s, 3H), 2.29 – 2.22 (m, 1H), 1.00 (t, *J* = 7.3 Hz, 3H).

^{13}C NMR (175 MHz, $CDCl_3$) δ 159.8, 155.2, 138.5, 137.0, 129.2, 128.4, 126.9, 123.9, 122.8, 120.9, 111.4, 103.9, 84.9, 73.8, 46.9, 33.5, 21.1, 9.8.

IR (KBr): 3298, 3029, 2973, 2935, 2877, 1576, 1510, 1454, 1250, 1182, 976, 869, 814, 750, 640 cm^{-1}

HRMS (ESI) [$C_{20}H_{19}O$] $[M+H]^+$ calculated: 275.1436, found: 275.1439.

5-Phenyl-2-(3-phenylpent-1-yn-3-yl)pyridine (4ta)

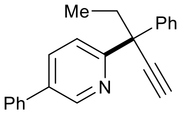

Compound **4ta** was prepared according to the **General procedure G** from **1t** (34.6 mg, 0.2 mmol) and **3a** (43.2 mg, 0.3 mmol) using $PhMe_2SiBpin$ under 50 ^oC, and purified by silica gel column chromatography (*n*-hexane/DCM: 2/1) as a colorless oil (40.6 mg, yield: 68%).

^{1}H NMR (500 MHz, $CDCl_3$) δ 8.83 (dd, *J* = 2.4, 0.9 Hz, 1H), 7.80 (dd, *J* = 8.2, 2.4 Hz, 1H), 7.69 (dd, *J* = 8.2, 0.8 Hz, 1H), 7.63 – 7.60 (m, 2H), 7.59 – 7.55 (m, 2H), 7.50 – 7.44 (m, 2H), 7.42 – 7.37 (m, 1H), 7.35 – 7.30 (m, 2H), 7.25 – 7.20 (m, 1H), 2.70 (s, 1H), 2.75 – 2.63 (m, 1H), 2.53 – 2.33 (m, 1H), 1.01 (t, *J* = 7.3 Hz, 3H).

^{13}C NMR (126 MHz, $CDCl_3$) δ 161.8, 147.2, 143.7, 137.8, 135.0, 134.6, 129.2, 128.3, 128.1, 127.24, 127.18, 126.9, 122.9, 87.2, 74.8, 52.4, 33.8, 10.0.

IR (KBr): 3297, 3060, 3028, 2972, 2933, 2876, 1593, 1471, 1447, 1369, 1031, 1005, 841, 747, 696, 649 cm^{-1}

HRMS (ESI) [$C_{22}H_{20}N$] $[M+H]^+$ calculated: 298.1596, found: 298.1599.

2-(3-Phenylpent-1-yn-3-yl)benzofuran (4ua)

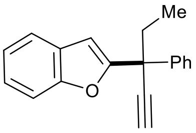

Compound **4ua** was prepared according to the **General procedure G** from **1u** (27.2 mg, 0.2 mmol) and **3a** (43.2 mg, 0.3 mmol) using $PhMe_2SiBpin$ under 50 ^oC, and purified by silica gel column chromatography (*n*-hexane/DCM: 15/1) as a colorless oil (34.4 mg, yield: 66%).

^{1}H NMR (700 MHz, $CDCl_3$) δ 7.63 – 7.60 (m, 2H), 7.56 (ddd, *J* = 7.5, 1.4, 0.6 Hz, 1H), 7.47 – 7.45 (m, 1H), 7.38 – 7.34 (m, 2H), 7.31 – 7.28 (m, 1H), 7.28 – 7.25 (m, 1H), 7.23 (td, *J* = 7.5, 1.1 Hz, 1H), 6.79 (d, *J* = 0.9 Hz, 1H), 2.66 (s, 1H), 2.59 – 2.47 (m, 1H), 2.36 – 2.20 (m, 1H), 1.04 (t, *J* = 7.3 Hz, 3H).

^{13}C NMR (176 MHz, $CDCl_3$) δ 159.5, 155.2, 141.4, 128.5, 128.3, 127.3, 127.0, 124.0, 122.8, 121.0, 111.4, 104.1, 84.7, 74.0, 47.2, 33.6, 9.8.

IR (KBr): 3294, 3061, 2973, 2935, 2877, 1598, 1577, 1492, 1454, 1379, 1306, 1249, 1181, 1032, 975, 867, 809, 741, 658 cm^{-1}

HRMS (ESI) [$C_{19}H_{16}ONa$] $[M+Na]^+$ calculated: 283.1099, found: 283.1088.

1-(3-Phenylpent-1-yn-3-yl)naphthalene (4va)

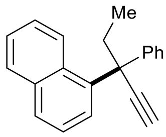

Compound **4va** was prepared according to the **General procedure G** from **1v** (29.2 mg, 0.2 mmol) and **3a** (43.2 mg, 0.3 mmol) using $Et_3SiBpin$ under 50 ºC, and purified by silica gel column chromatography (*n*-hexane) as a colorless oil (32.2 mg, yield: 59%).

^{1}H NMR (500 MHz, $CDCl_3$) δ 8.01 (dd, *J* = 8.7, 1.0 Hz, 1H), 7.86 (dd, *J* = 7.4, 1.1 Hz, 1H), 7.82 – 7.75 (m, 2H), 7.50 (dd, *J* = 8.2, 7.4 Hz, 1H), 7.39 – 7.35 (m, 2H), 7.34 – 7.29 (m, 1H), 7.25 – 7.12 (m, 4H), 2.59 (dq, *J* = 12.6, 7.3 Hz, 1H), 2.58 (s, 1H), 2.30 (dq, *J* = 12.6, 7.3 Hz, 1H), 0.99 (t, *J* = 7.2 Hz, 3H).

^{13}C NMR (126 MHz, $CDCl_3$) δ 145.1, 139.6, 135.0, 131.1, 128.8, 128.6, 128.3, 127.3, 127.0, 126.5, 125.2, 125.0, 124.8, 124.4, 87.7, 74.6, 48.9, 35.9, 9.8.

IR (KBr): 3297, 3052, 2975, 2876, 1599, 1508, 1488, 1448, 1397, 1264, 1076, 1032, 907, 796, 776, 702, 641 cm^{-1}

HRMS (ESI) [$C_{21}H_{19}$] $[M+H]^+$ calculated: 271.1487, found: 271.1490.

Triethyl(3-(3-phenylpent-1-yn-3-yl)phenyl)silane (4wa)

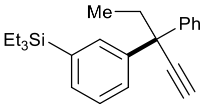

Compound **4wa** was prepared according to the **General procedure G** from **1w** (42.0 mg, 0.2 mmol) and **3a** (43.2 mg, 0.3 mmol) using $PhMe_2SiBpin$ under 50 ºC, and purified by silica gel column chromatography (*n*-hexane) as a colorless oil (27.1 mg, yield: 40%).

^{1}H NMR (500 MHz, $CDCl_3$) δ 7.60 (s, 1H), 7.45 (dd, *J* = 8.4, 1.3 Hz, 2H), 7.39 (ddd, *J* = 7.8, 2.1, 1.3 Hz, 1H), 7.37 – 7.27 (m, 4H), 7.24 – 7.18 (m, 1H), 2.59 (s, 1H), 2.33 (qd, *J* = 7.3, 1.6 Hz, 2H), 0.99 (t, *J* = 7.2 Hz, 3H), 0.95 (t, *J* = 7.8 Hz, 9H), 0.77 (q, *J* = 7.5 Hz, 6H).

^{13}C NMR (126 MHz, $CDCl_3$) δ 145.0, 143.7, 137.2, 133.2, 132.5, 128.3, 128.1, 127.6, 127.5, 126.6, 88.2, 73.8, 50.2, 34.4, 10.1, 7.5, 3.5.

IR (KBr): 3306, 3055, 2953, 2877, 1596, 1492, 1447, 1416, 1394, 1237, 1120, 1082, 1013, 791, 647 cm^{-1}

HRMS (ESI) [$C_{23}H_{31}Si$] $[M+H]^+$ calculated: 335.2195, found: 335.2185.

1-(3-Phenylpent-1-yn-3-yl)-4-(trifluoromethyl)benzene (4xa)

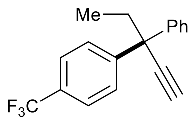

Compound **4xa** was prepared according to the **General procedure G** from **1x** (32.8 mg, 0.2 mmol) and **3a** (43.2 mg, 0.3 mmol) using $Et_3SiBpin$ under room temperature, and purified by silica gel column chromatography (*n*-hexane) as a colorless oil (23.7 mg, yield: 41%).

^{1}H NMR (500 MHz, $CDCl_3$) δ 7.55 (d, *J* = 1.7 Hz, 4H), 7.42 (dd, *J* = 8.3, 1.2 Hz, 2H), 7.36 – 7.26 (m, 2H), 7.27 – 7.17 (m, 1H), 2.62 (s, 1H), 2.33 (p, *J* = 7.2 Hz, 2H), 0.97 (t, *J* = 7.3 Hz, 3H).

^{13}C NMR (126 MHz, $CDCl_3$) δ 149.1, 144.0, 128.9 (q, *J* = 32.8 Hz), 128.5, 127.9, 127.4, 127.1, 125.3, 124.3 (q, *J* = 271.9 Hz), 87.2, 74.6, 50.2, 34.2, 9.9.

^{19}F NMR (282 MHz, $CDCl_3$) δ -62.92 (s, 3F).

IR (KBr): 3306, 3061, 2976, 2938, 2880, 1618, 1493, 1447, 1411, 1327, 1128, 1069, 1018, 834, 756, 700, 651 cm^{-1}

HRMS (ESI) [$C_{18}H_{16}F_3$] $[M+H]^+$ calculated: 289.1204, found: 289.1017.

4-(2-Ethyl-2-phenylbut-3-yn-1-yl)-1,1'-biphenyl (5aa)

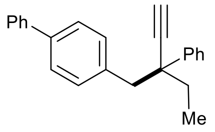

Compound **5aa** was prepared according to the **General procedure G** from **2a** (37.2 mg, 0.2 mmol) and **3a** (43.2 mg, 0.3 mmol) using $Et_3SiBpin$ under room temperature, and purified by silica gel column chromatography (*n*-hexane/DCM: 15/1) as a colorless oil (55.2 mg, yield: 89%).

^{1}H NMR (500 MHz, $CDCl_3$) δ 7.60 – 7.49 (m, 2H), 7.48 – 7.43 (m, 2H), 7.42 – 7.35 (m, 4H), 7.32 – 7.27 (m, 3H), 7.26 – 7.20 (m, 1H), 7.02 (d, *J* = 8.3 Hz, 2H), 3.10 (s, 2H), 2.48 (s, 1H), 2.14 – 1.82 (m, 2H), 0.88 (t, *J* = 7.3 Hz, 3H).

^{13}C NMR (126 MHz, $CDCl_3$) δ 142.1, 141.1, 139.2 136.5, 131.2, 128.8, 128.2, 127.3, 127.1, 126.7, 126.2, 87.2, 74.7, 49.4, 47.5, 34.3, 9.7.

IR (KBr): 3294, 3059, 3028, 2969, 2932, 2874, 1599, 1519, 1488, 1408, 1378, 1267, 1077, 1007, 911, 834, 736, 700, 650 cm^{-1}

HRMS (ESI) [$C_{24}H_{23}$] $[M+H]^+$ calculated: 311.1800, found: 311.1807.

1-(2-Ethyl-2-phenylbut-3-yn-1-yl)-4-(trifluoromethoxy)benzene (5ba)

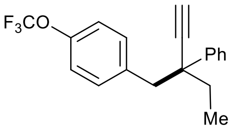

Compound **5ba** was prepared according to the **General procedure G** from **2b** (38.8 mg, 0.2 mmol) and **3a** (43.2 mg, 0.3 mmol) using $Et_3SiBpin$ under room temperature, and purified by silica gel column chromatography (*n*-hexane/DCM: 10/1) as a colorless oil (41.5 mg, yield: 65%).

^{1}**H NMR** (500 MHz, $CDCl_3$) δ 7.43 – 7.38 (m, 2H), 7.33 – 7.27 (m, 2H), 7.25 – 7.20 (m, 1H), 6.95 (q, *J* = 8.7 Hz, 4H), 3.29 – 2.88 (m, 2H), 2.48 (s, 1H), 2.17 – 1.82 (m, 2H), 0.88 (t, *J* = 7.3 Hz, 3H).

^{13}**C NMR** (126 MHz, $CDCl_3$) δ 148.0, 141.6, 136.1, 132.0, 128.3, 127.2, 126.8, 120.6 (q, *J* = 256.6 Hz), 119.9, 86.8, 74.9, 48.9, 47.5, 34.5, 9.6.

^{19}**F NMR** (282 MHz, $CDCl_3$) δ -58.33 (s, 3F).

IR (KBr): 3308, 3030, 2972, 2936, 2879, 1599, 1509, 1448, 1268, 1165, 1021, 840, 759, 700, 633 cm^{-1}

HRMS (ESI) [$C_{19}H_{18}F_3O$] $[M+H]^+$ calculated: 319.1310, found: 319.1312.

1-Chloro-4-(2-ethyl-2-phenylbut-3-yn-1-yl)benzene (5ca)

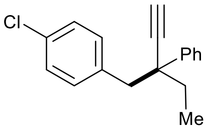

Compound **5ca** was prepared according to the **General procedure G** from **2c** (28.8 mg, 0.2 mmol) and **3a** (43.2 mg, 0.3 mmol) using $Et_3SiBpin$ under room temperature, and purified by silica gel column chromatography (*n*-hexane/DCM: 10/1) as a colorless oil (44.5 mg, yield: 83%).

^{1}**H NMR** (500 MHz, $CDCl_3$) δ 7.43 – 7.37 (m, 2H), 7.33 – 7.28 (m, 2H), 7.25 – 7.20 (m, 1H), 6.90 – 6.80 (m, 2H), 3.17 – 2.90 (m, 2H), 2.48 (s, 1H), 2.14 – 1.82 (m, 2H), 0.88 (t, *J* = 7.3 Hz, 3H).

^{13}**C NMR** (126 MHz, $CDCl_3$) δ 141.6, 135.8, 132.3, 132.0, 128.2, 127.6, 127.2, 126.8, 86.8, 74.9, 49.0, 47.6, 34.5, 9.6.

IR (KBr): 3301, 3029, 2970, 2934, 2877, 1599, 1491, 1447, 1095, 1016, 833, 760, 724, 700, 639 cm^{-1}

HRMS (ESI) [$C_{18}H_{18}Cl$] $[M+H]^+$ calculated: 269.1097, found: 269.1099.

1-Bromo-4-(2-ethyl-2-phenylbut-3-yn-1-yl)benzene (5da)

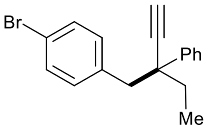

Compound **5da** was prepared according to the **General procedure G** from **2d** (37.8 mg, 0.2 mmol) and **3a** (43.2 mg, 0.3 mmol) using $Et_3SiBpin$ under room temperature, and purified by silica gel column chromatography (*n*-hexane/DCM: 20/1) as a colorless oil (37.4 mg, yield: 59%).

^{1}H NMR (500 MHz, $CDCl_3$) δ 7.42 – 7.34 (m, 2H), 7.32 – 7.26 (m, 2H), 7.25 – 7.18 (m, 3H), 6.82 – 6.74 (m, 2H), 3.07 – 2.94 (m, 2H), 2.45 (s, 1H), 2.12 – 1.83 (m, 2H), 0.86 (t, *J* = 7.3 Hz, 3H).

^{13}C NMR (126 MHz, $CDCl_3$) δ 141.6, 136.3, 132.4, 130.5, 128.2, 127.2, 126.8, 120.5, 86.8, 74.9, 49.0, 47.5, 34.6, 9.6.

IR (KBr): 3299, 3060, 3027, 2969, 2933, 2876, 1599, 1509, 1488, 1447, 1405, 1379, 1012, 831, 803, 760, 720, 700, 641 cm^{-1}

HRMS (ESI) [$C_{18}H_{18}Br$] $[M+H]^+$ calculated: 313.0592, found: 313.0596.

1-(*tert*-Butyl)-4-(2-ethyl-2-phenylbut-3-yn-1-yl)benzene (5ea)

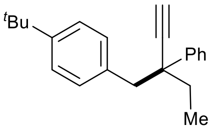

Compound **5ea** was prepared according to the **General procedure G** from **2e** (33.2 mg, 0.2 mmol) and **3a** (43.2 mg, 0.3 mmol) using $Et_3SiBpin$ under room temperature, and purified by silica gel column chromatography (*n*-hexane/DCM: 20/1) as a white solid (50.4 mg, yield: 86%), m.p.: 73.0 – 74.2 °C.

^{1}H NMR (500 MHz, $CDCl_3$) δ 7.50 – 7.44 (m, 2H), 7.35 – 7.29 (m, 2H), 7.26 – 7.21 (m, 1H), 7.20 – 7.17 (m, 2H), 6.95 (d, *J* = 8.3 Hz, 2H), 3.06 (s, 2H), 2.48 (s, 1H), 2.09 – 1.79 (m, 2H), 1.29 (s, 9H), 0.87 (t, *J* = 7.3 Hz, 3H).

^{13}C NMR (126 MHz, $CDCl_3$) δ 149.1, 142.4, 134.3, 130.5, 128.1, 127.2, 126.6, 124.4, 87.5, 74.4, 49.3, 47.4, 34.5, 34.0, 31.5, 9.6.

IR (KBr): 3273, 3255, 3058, 2963, 2869, 1600, 1517, 1496, 1452, 1363, 1267, 1111, 1024, 838, 763, 700, 652, 637 cm^{-1}

HRMS (ESI) [$C_{22}H_{27}$] $[M+H]^+$ calculated: 291.2113, found: 291.2113.

2-(2-Ethyl-2-phenylbut-3-yn-1-yl)naphthalene (5fa)

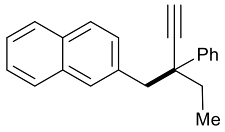

Compound **5fa** was prepared according to the **General procedure G** from **2f** (32.0 mg, 0.2 mmol) and **3a** (43.2 mg, 0.3 mmol) using $Et_3SiBpin$ under room temperature, and purified by silica gel column chromatography (*n*-hexane/DCM: 10/1) as a colorless oil (46.0 mg, yield: 80%).

^{1}H NMR (700 MHz, $CDCl_3$) δ 7.76 – 7.71 (m, 1H), 7.68 – 7.64 (m, 1H), 7.58 (d, *J* = 8.4 Hz, 1H), 7.44 – 7.41 (m, 2H), 7.41 (s, 1H), 7.40 – 7.35 (m, 2H), 7.30 – 7.25 (m, 2H), 7.24 – 7.18 (m, 1H), 7.05 (dd, *J* = 8.4, 1.7 Hz, 1H), 3.33 – 3.03 (m, 2H), 2.46 (s, 1H), 2.17 – 1.89 (m, 2H), 0.88 (t, *J* = 7.3 Hz, 3H).

^{13}C NMR (176 MHz, $CDCl_3$) δ 142.1, 135.0, 133.1, 132.3, 129.5, 129.4, 128.2, 127.8, 127.6, 127.3, 126.7, 125.7, 125.4, 87.3, 74.8, 50.0, 47.7, 34.3, 9.7.

IR (KBr): 3298, 3056, 3024, 2968, 2933, 2876, 1600, 1508, 1494, 1446, 1378, 1271, 1126, 1032, 959, 895, 819, 748, 701, 639 cm^{-1}

HRMS (ESI) [$C_{22}H_{21}$] $[M+H]^+$ calculated: 285.1643, found: 285.1645.

1-(2-Ethyl-2-phenylbut-3-yn-1-yl)naphthalene (5ga)

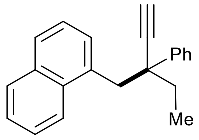

Compound **5ga** was prepared according to the **General procedure G** from **2g** (32.0 mg, 0.2 mmol) and **3a** (43.2 mg, 0.3 mmol) using $Et_3SiBpin$ under room temperature, and purified by silica gel column chromatography (*n*-hexane/DCM: 10/1) as a colorless oil (41.4 mg, yield: 73%).

^{1}H NMR (500 MHz, $CDCl_3$) δ 8.04 – 7.94 (m, 1H), 7.84 – 7.74 (m, 1H), 7.70 (d, *J* = 8.2 Hz, 1H), 7.50 – 7.46 (m, 2H), 7.43 – 7.33 (m, 2H), 7.31 – 7.26 (m, 3H), 7.24 – 7.19 (m, 1H), 7.15 (dd, *J* = 7.2, 1.2 Hz, 1H), 3.71 (d, *J* = 14.0 Hz, 1H), 3.49 (d, *J* = 14.0 Hz, 1H), 2.35 (s, 1H), 2.22 – 2.08 (m, 1H), 2.08 – 1.91 (m, 1H), 0.89 (t, *J* = 7.3 Hz, 3H).

^{13}C NMR (126 MHz, $CDCl_3$) δ 142.5, 133.7, 133.4, 129.1, 128.5, 128.2, 127.2, 126.7, 125.1, 125.0, 124.9, 87.5, 75.0, 47.6, 45.2, 34.2, 9.8.

IR (KBr): 3301, 3060, 2969, 2933, 2876, 1598, 1510, 1493, 1446, 1397, 1262, 1019, 780, 701, 635 cm^{-1}

HRMS (ESI) [$C_{22}H_{21}$] $[M+H]^+$ calculated: 285.1643, found: 285.1639.

Adapalene derivative (4adc)

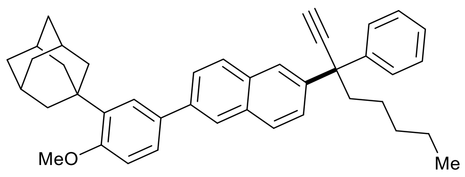

Compound **4adc** was prepared according to the **General procedure F** from **1ad** (77.2 mg, 0.2 mmol) and **3c** (112.0 mg, 0.6 mmol) using $PhMe_2SiBpin$, and purified by silica gel column chromatography (*n*-hexane/DCM: 7/1) as a colorless oil (79.2 mg, yield: 71%).

^{1}H NMR (500 MHz, $CDCl_3$) δ 8.03 (s, 1H), 7.92 (s, 1H), 7.88 (d, *J* = 8.5 Hz, 1H), 7.76 (d, *J* = 8.7 Hz, 1H), 7.73 (dd, *J* = 8.5, 1.8 Hz, 1H), 7.57 (d, *J* = 2.3 Hz, 1H), 7.51 (dd, *J* = 8.4, 2.3 Hz, 1H), 7.49 – 7.45 (m, 2H), 7.39 (dd, *J* = 8.7, 1.9 Hz, 1H), 7.29 (t, *J* = 7.7 Hz, 2H), 7.22 – 7.17 (m, 1H), 6.97 (d, *J* = 8.5 Hz, 1H), 3.87 (s, 3H), 2.64 (s, 1H), 2.37 (tq, *J* = 18.3, 6.6, 5.6 Hz, 2H), 2.17 (br-s, 6H), 2.09 (br-s, 3H), 1.79 (br-s, 6H), 1.44 – 1.36 (m, 2H), 1.36 – 1.23 (m, 4H), 0.85 (t, *J* = 6.9 Hz, 3H).

^{13}C NMR (126 MHz, $CDCl_3$) δ 158.6, 145.1, 142.1, 139.1, 138.9, 133.3, 132.6, 131.9, 128.6, 128.34, 128.26, 127.5, 126.7, 126.6, 126.04, 126.0, 125.7, 125.1, 124.7, 112.1, 88.3, 74.1, 55.3, 49.5, 41.1, 40.7, 37.3, 32.3, 29.2, 25.3, 22.7, 14.3.

IR (KBr): 3302, 3055, 2953, 2903, 2850, 1603, 1493, 1454, 1236, 1138, 1030, 908, 881, 737, 701, 639 cm^{-1}

HRMS (ESI) [$C_{41}H_{45}O$] $[M+H]^+$ calculated: 553.3470, found: 553.3476.

Estrone derivative (4aei)

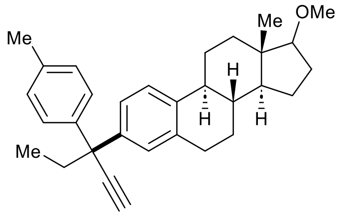

Compound **4aei** was prepared according to the **General procedure F** from **1ae** (57.6 mg, 0.2 mmol) and **3i** (94.8 mg, 0.6 mmol) using $Et_3SiBpin$, and purified by silica gel column chromatography (*n*-hexane/DCM: 5/1) as a colorless oil (36.2 mg, yield: 42%).

^{1}H NMR (300 MHz, $CDCl_3$) δ 7.38 – 7.30 (m, 2H), 7.23 – 7.14 (m, 3H), 7.13 – 7.06 (m, 2H), 3.37 (s, 3H), 3.30 (t, *J* = 8.2 Hz, 1H), 2.84(dd, *J* = 9.8, 4.4 Hz, 2H), 2.54 (s, 1H), 2.30 (s, 3H), 2.29 – 2.22 (m, 3H), 2.12 – 1.96 (m, 2H), 1.93 – 1.78 (m, 1H), 1.76 – 1.59 (m, 1H), 1.55 – 1.44 (m, 2H), 1.43 – 1.40 (m, 1H), 1.38 (d, *J* = 3.8 Hz, 1H), 1.33 (t, *J* = 4.4 Hz, 1H), 1.27 (t, *J* = 2.4 Hz, 1H), 1.24 – 1.14 (m, 1H), 0.95 (t, *J* = 7.2 Hz, 3H), 0.91 – 0.84 (m, 1H), 0.77

(s, 3H).

^{13}C NMR (75 MHz, $CDCl_3$) δ 142.3, 141.9, 138.6, 136.4, 136.0, 129.0, 127.8, 127.3, 125.2, 124.6, 90.9, 88.4, 73.5, 58.0, 50.5, 49.4, 44.3, 43.3, 38.4, 38.2, 34.3, 29.9, 27.9, 27.4, 26.2, 23.2, 21.1, 11.7, 10.2.

IR (KBr): 3305, 3026, 2971, 2932, 2871, 2848, 1510, 1496, 1265, 1134, 1105, 810, 735, 633 cm^{-1}

HRMS (ESI) [$C_{31}H_{39}O$] $[M+H]^+$ calculated: 427.3001, found: 427.3004.

Blonanserin derivative (Antipsychotic) (4afg)

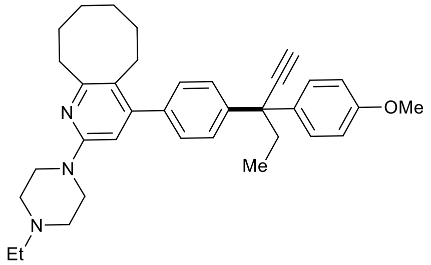

Compound **4afg** was prepared according to the **General procedure F** from **1af** (74.0 mg, 0.2 mmol) and **3g** (104.4 mg, 0.6 mmol) using $PhMe_2SiBpin$, and purified by silica gel column chromatography (DCM) as a colorless oil (93.5 mg, yield: 89%).

^{1}H NMR (300 MHz, $CDCl_3$) δ 7.49 – 7.34 (m, 4H), 7.18 (d, J = 8.0 Hz, 2H), 6.92 – 6.78 (m, 2H), 6.31 (s, 1H), 3.79 (s, 3H), 3.52 (t, J = 5.2 Hz, 4H), 2.88 (t, J = 6.1 Hz, 2H), 2.64 – 2.51 (m, 7H), 2.47 (q, J = 7.2 Hz, 2H), 2.31 (q, J = 7.2 Hz, 2H), 1.78 (dq, J = 11.9, 6.4, 5.3 Hz, 2H), 1.49 – 1.29 (m, 6H), 1.13 (t, J = 7.1 Hz, 3H), 0.99 (t, J = 7.2 Hz, 3H).

^{13}C NMR (126 MHz, $CDCl_3$) δ 159.8, 158.2, 157.3, 151.2, 144.1, 139.6, 136.7, 128.5, 128.4, 126.9, 123.1, 113.6, 106.1, 88.1, 73.8, 55.3, 52.8, 52.5, 49.2, 45.6, 35.6, 34.5, 31.7, 30.8, 26.6, 26.6, 25.9, 12.0, 10.1.

IR (KBr): 3303, 3034, 2932, 2831, 2770, 2680, 1608, 1585, 1506, 1471, 1411, 1251, 1182, 1126, 1037, 998, 830, 733, 638 cm^{-1}

HRMS (ESI) [$C_{35}H_{44}N_3O$] $[M+H]^+$ calculated: 522.3484, found: 522.3484.

Liquid crystal material derivative (4agg)

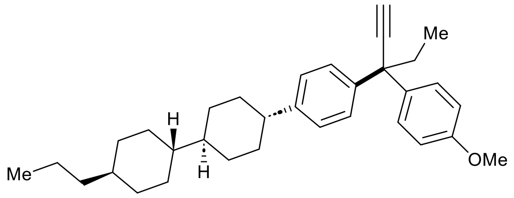

Compound **4agg** was prepared according to the **General procedure F** from **1ag** (61.0 mg, 0.2 mmol) and **3g** (104.4 mg, 0.6 mmol) using $PhMe_2SiBpin$, and purified by silica gel column chromatography (*n*-hexane/DCM: 5/1) as a

white solid (61.0 mg, yield: 67%), m.p.: 83.6 – 85.3 °C.

^{1}H NMR (300 MHz, $CDCl_3$) δ 7.40 – 7.26 (m, 4H), 7.17 – 7.06 (m, 2H), 6.89 – 6.77 (m, 2H), 3.78 (s, 3H), 2.56 (s, 1H), 2.41 (tt, J = 12.1, 3.3 Hz, 1H), 2.27 (q, J = 7.2 Hz, 2H), 1.96 – 1.67 (m, 8H), 1.46 – 1.22 (m, 4H), 1.21 – 1.09 (m, 5H), 1.02 (d, J = 7.0 Hz, 2H), 0.95 (t, J = 7.2 Hz, 3H), 0.88 (t, J = 7.2 Hz, 4H).

^{13}C NMR (75 MHz, $CDCl_3$) δ 158.1, 146.1, 142.5, 137.1, 128.5, 127.1, 126.7, 113.5, 88.5, 73.4, 55.3, 49.1, 44.2, 43.5, 43.0, 40.0, 37.7, 34.7, 34.5, 33.7, 30.5, 30.2, 20.2, 14.6, 10.1.

IR (KBr): 3276, 3055, 2949,2911, 2846, 1608, 1510, 1451, 1298, 1251, 1179, 1033, 830, 652 cm^{-1}

HRMS (ESI) [$C_{33}H_{45}$] [M+H]$^+$ calculated: 457.3470, found: 457.3464.

4-(1-Cyclopropyl-1-phenylprop-2-yn-1-yl)biphenyl (4ao)

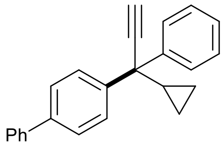

Compound **4ao** was prepared according to the **General procedure F** from **1a** (17.2 mg, 0.1 mmol) and **3o** (46.8 mg, 0.3 mmol) using $Et_3SiBpin$, and purified by silica gel column chromatography (*n*-hexane/DCM: 20/1) as a light-yellow oil (7.3 mg, yield: 24% yield).

^{1}H NMR (300 MHz, $CDCl_3$) δ 7.62 – 7.49 (m, 7H), 7.46 – 7.38 (m, 2H), 7.36 – 7.29 (m, 3H), 7.28 – 7.20 (m, 2H), 2.48 (s, 1H), 1.66 – 1.52 (m, 1H), 1.00 – 0.87 (m, 2H), 0.70 – 0.63 (m, 2H).

^{13}C NMR (176 MHz, $CDCl_3$) δ 145.9, 145.2, 140.9, 139.6, 128.8, 128.3, 127.9, 127.3, 127.2, 127.0, 126.9, 84.9, 74.2, 50.9, 20.7, 3.5, 3.4.

HRMS (ESI) [$C_{24}H_{21}$] [M+H]$^+$ calculated: 309.1643, found: 309.1648.

1-Methoxy-4-(pent-1-yn-3-yl)benzene (15)

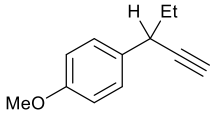

Compound **15** was prepared according to the **General procedure F** from **3g** (46.8 mg, 0.3 mmol) using $Et_3SiBpin$ under room temperature or 50 °C and purified by silica gel column chromatography (*n*-hexane/DCM: 5/1) as a colorless oil (33.9 mg, yield: 65% (room temperature)).

^{1}H NMR (300 MHz, $CDCl_3$) δ 7.27 (d, J = 8.7 Hz, 2H), 6.86 (d, J = 8.5 Hz, 2H), 3.79 (s, 3H), 3.52 (td, J = 7.1, 2.5 Hz, 1H), 2.26 (d, J = 2.6 Hz, 1H), 1.76 (p, J = 7.3 Hz, 2H), 0.98 (t, J = 7.3 Hz, 3H).

^{13}C NMR (75 MHz, $CDCl_3$) δ 158.5, 133.6, 128.5, 113.9, 86.4, 70.9, 55.4, 38.4, 31.5, 11.8.

MS(EI): *m/z* 174 $[M]^+$. The chemical shifts were consistent with those reported in the literature.[27]

4. Synthetic Utility

4.1 Quench with different nucleophiles

Quench with aldehyde (6)

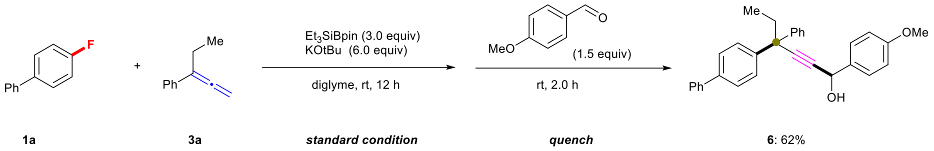

Following the **General procedure F** from **1a** (34.4 mg, 0.2 mmol) and **3a** (86.4 mg, 0.6 mmol) using $Et_3SiBpin$. After the mixture stirred at room temperature for 12 h, then quenched by adding *p*-anisaldehyde (36 µL, 0.3 mmol) and keep stirring for 2.0 h at room temperature. Then the mixture was diluted with Et_2O and saturated NH_4Cl, and extracted with Et_2O, washed with water, dried over Na_2SO_4, and concentrated under vacuum to give the crude. The crude was purified by silica gel column chromatography (*n*-hexane/EtOAc: 5/1) to afford alcohol **6** as a colorless oil (53.7 mg, yield: 62%).

^{1}H NMR (500 MHz, $CDCl_3$) δ 7.59 – 7.54 (m, 2H), 7.53 – 7.45 (m, 8H), 7.43 – 7.38 (m, 2H), 7.34 – 7.27 (m, 3H), 7.24 – 7.18 (m, 1H), 6.97 – 6.81 (m, 2H), 5.57 (s, 1H), 3.78 (s, 3H), 2.36 (q, *J* = 7.2 Hz, 2H), 2.31 (s, 1H), 1.00 (t, *J* = 7.2 Hz, 3H).

^{13}C NMR (126 MHz, $CDCl_3$) δ 159.7, 145.0, 144.3, 140.7, 139.4, 133.5, 128.8, 128.3, 127.9, 127.5, 127.3, 127.1, 127.0, 126.7, 114.0, 90.8, 85.8, 64.7, 55.4, 50.1, 34.4, 10.3.

IR (KBr): 3552, 3443, 3390, 3057, 3030, 2970, 2876, 1611, 1512, 1487, 1250, 1172, 1073, 1034, 990, 834, 699, 568 cm^{-1}

HRMS (ESI) $[C_{31}H_{28}O_2Na]$ $[M+Na]^+$ calculated: 455.1987, found: 455.1985.

Quench with D_2O (*d*-4aa)

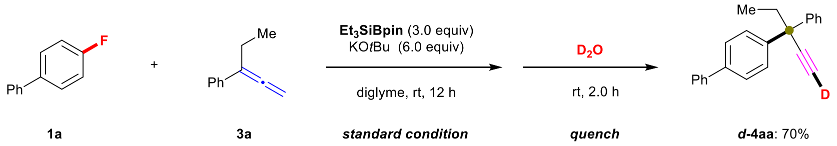

Following the **General procedure F** from **1a** (34.4 mg, 0.2 mmol) and **3a** (86.4 mg, 0.6 mmol) using $Et_3SiBpin$. After the mixture stirred at room temperature for 12 h, then quenched by adding D_2O (2.0 mL) and keep stirring for 2.0 h at room temperature. Then the mixture was diluted with Et_2O and saturated NH_4Cl, and extracted with Et_2O,

washed with water, dried over Na_2SO_4, and concentrated under vacuum to give the crude. The crude was purified by silica gel column chromatography (*n*-hexane/DCM: 10/1) to afford ***d*-4aa** as a white solid (41.6 mg, yield: 70%).

^{1}H NMR (300 MHz, $CDCl_3$) δ 7.62 – 7.54 (m, 2H), 7.50 (d, *J* = 4.2 Hz, 4H), 7.49 – 7.46 (m, 1H), 7.45 – 7.38 (m, 2H), 7.36 – 7.28 (m, 3H), 7.26 – 7.17 (m, 1H), 2.61 (s, 0.12H), 2.35 (q, *J* = 7.3 Hz, 2H), 1.00 (t, *J* = 7.3 Hz, 3H).

IR (KBr): 3295, 3057, 3028, 2972, 2934, 2876, 2588, 1599, 1487, 1446, 1403, 1194, 1077, 1008, 908, 833, 761, 730, 698, 635 cm^{-1}

Quench with *N*-bromosuccinimide (7)

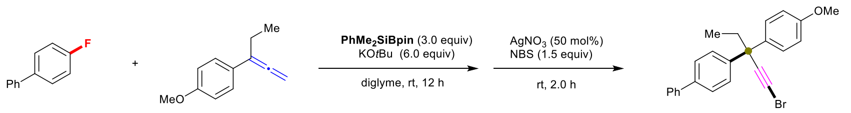

Following the **General procedure F** from **1a** (34.4 mg, 0.2 mmol) and **3g** (104.4 mg, 0.6 mmol) using $PhMe_2SiBpin$. After the mixture stirred at room temperature for 12 h, then quenched by adding NBS (71.2 mg, 0.4 mmol) and catalytic amount of silver(I)nitrate (17 mg, 0.1 mmol), then continue stirring for 2.0 h in glovebox at room temperature. Then the mixture was diluted with Et_2O and saturated NH_4Cl, and extracted with Et_2O, washed with water, dried over Na_2SO_4, and concentrated under vacuum to give the crude. The crude was purified by silica gel column chromatography (*n*-hexane/DCM: 10/1) to afford **7** as a colorless oil (71.7 mg, yield: 89%).

^{1}H NMR (500 MHz, $CDCl_3$) δ 7.58 – 7.54 (m, 2H), 7.52 – 7.49 (m, 2H), 7.45 – 7.38 (m, 4H), 7.35 – 7.29 (m, 3H), 6.88 – 6.74 (m, 2H), 3.76 (s, 3H), 2.30 (q, *J* = 7.2 Hz, 2H), 0.97 (t, *J* = 7.2 Hz, 3H).

^{13}C NMR (75 MHz, $CDCl_3$) δ 158.3, 144.2, 140.8, 139.4, 136.7, 128.9, 128.5, 127.8, 127.3, 127.1, 127.0, 113.6, 84.1, 55.3, 50.6, 43.9, 34.5, 10.2.

IR (KBr): 3057, 3029, 2970, 2934, 2876, 2834, 1608, 1506, 1486, 1403, 1301, 1252, 1180, 1037, 837, 766, 698 cm^{-1}

HRMS (ESI) [$C_{24}H_{22}BrO$] $[M+H]^+$ calculated: 405.0854, found: 405.0855.

4.2 Scale-up reaction

Following the **General procedure F** with modification, charging **1a** (602 mg, 3.5 mmol), anhydrous diglyme (25 mL), KO*t*Bu (2.35 g, 21.0 mmol) sequentially into a flame-dried flask. Then move the flask out of glovebox after sealed, and then injected a solution of $Et_3SiBpin$ (2.54 g, 10.5 mmol) and **3a** (1.51 g, 10.5 mmol) in anhydrous diglyme (15 mL) slowly, thereafter stirred at room temperature for 12 h. The reaction mixture was diluted with Et_2O

(50 mL) and quenched by adding saturated NH_4Cl while stirring for 5 min, then extracted with Et_2O, washed by water, dried over Na_2SO_4, and concentrated under vacuum to give the crude. The crude was purified by silica gel column chromatography (*n*-hexane/DCM: 10/1) to afford alcohol **4aa** as a light-yellow oil (700 mg, yield: 68%).

4.3 Synthetic transformations

Sonogashira coupling (8)

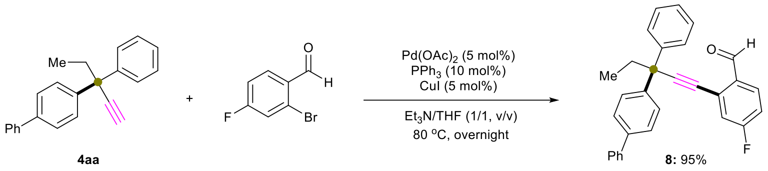

In a N_2 filled glovebox, to a solution of 2-bromo-4-fluoro-benzaldehyde (48.7 mg, 0.24 mmol) and **4aa** (59.2 mg, 0.2 mmol) in dry Et_3N (0.5 mL) was added $Pd(OAc)_2$ (4.5 mg, 0.01 mmol), PPh_3 (10.5 mg, 0.02 mmol) and CuI (3.81 mg, 0.01 mmol). Then dry THF (0.5 mL) was added to result a mixture which was sealed and keep stirring overnight at 80 ^{o}C. After completion of the reaction, the reaction mixture was cooled to room temperature and filtered through a short pad of celite, washed with Et_2O. The filtrate was evaporated under reduced pressure to give the reaction crude, which was purified by silica gel column chromatography (*n*-hexane/EtOAc: 100/1) to afford benzaldehyde **8** as a colorless oil (80 mg, yield: 95%).

^{1}H NMR (300 MHz, $CDCl_3$) δ 10.52 (s, 1H), 7.98 (dd, *J* = 8.7, 5.9 Hz, 1H), 7.63 – 7.56 (m, 4H), 7.55 – 7.48 (m, 4H), 7.48 – 7.40 (m, 2H), 7.40 – 7.24 (m, 5H), 7.14 (td, *J* = 8.1, 2.2 Hz, 1H), 2.50 (q, *J* = 7.3 Hz, 2H), 1.09 (t, *J* = 7.2 Hz, 3H).

^{13}C NMR (75 MHz, $CDCl_3$) δ 190.1, 167.5, 164.1, 144.4, 143.6, 140.6, 139.8, 133.1, 130.2, 130.1, 128.9, 128.6, 127.8, 127.4, 127.2, 127.1, 127.0, 120.2, 116.4, 102.6, 80.7, 51.0, 34.4, 10.4.

^{19}F NMR (282 MHz, $CDCl_3$) δ -103.35 (s, 1F).

IR (KBr): 3058, 3031, 2972, 2934, 2879, 2839, 2749, 2223, 1696, 1601, 1574, 1486, 1389, 1268, 1250, 1198, 833, 765, 699 cm^{-1}

HRMS (ESI) [$C_{30}H_{24}FO$] $[M+H]^+$ calculated: 419.1811, found: 419.1814.

Silylation (9)

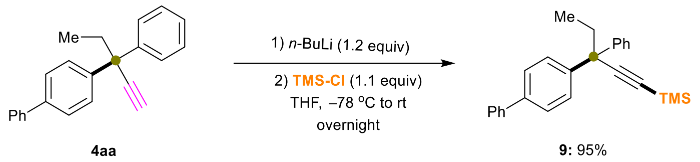

To a solution of **4aa** (59.2 mg, 0.2 mmol) in dry THF (1.0 mL) under −78ºC was injected *n*-BuLi (150 μL, 0.24 mmol) in a N_2 atmosphere. After keep stirring for 1 h at the same temperature, then trimethylsilyl chloride (30 μL, 0.22 mmol) was added. The reaction was allowed to continue stirring for another 30 min before warm-up to room temperature slowly. After completion of the reaction, the reaction mixture was quenched with saturated NH_4Cl, extracted with Et_2O, washed by water, brine, dried over Na_2SO_4, and concentrated under vacuum to afford the reaction crude. Which was purified by silica gel column chromatography (*n*-hexane/DCM: 10/1) to afford desired silane **9** as a white solid (70.2 mg, yield: 95%), m.p.: 82.7 – 83.8 °C.

^{1}H NMR (300 MHz, $CDCl_3$) δ 7.65 – 7.58 (m, 2H), 7.55 (s, 4H), 7.54 – 7.50 (m, 2H), 7.48 – 7.41 (m, 2H), 7.39 – 7.30 (m, 3H), 7.28 – 7.21 (m, 1H), 2.35 (q, *J* = 7.2 Hz, 2H), 1.04 (t, *J* = 7.2 Hz, 3H), 0.29 (s, 9H).

^{13}C NMR (75 MHz, $CDCl_3$) δ 145.0, 144.3, 140.9, 139.3, 128.8, 128.3, 127.9, 127.5, 127.3, 127.2, 127.0, 126.6, 110.1, 90.2, 50.9, 34.5, 10.2, 0.3.

IR (KBr): 3052, 3027, 2969, 2933, 2875, 2166, 1595, 1486, 1446, 1249, 991, 841, 761, 699 cm^{-1}

HRMS (ESI) [$C_{26}H_{29}Si$] $[M+H]^+$ calculated: 369.2039, found: 369.2042.

Reduction (10)

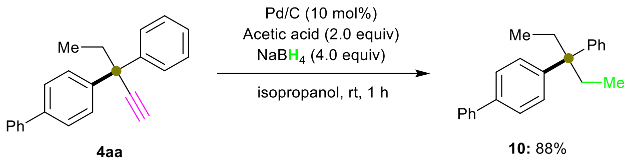

In an oven-dried reaction tube were added **4aa** (59.2 mg, 0.2 mmol), isopropanol (1.0 mL), Pd/C (3.2 mg, 0.02 mmol), $NaBH_4$ (31.0 mg, 0.8 mmol), then the tube was sealed and protected by N_2. Then acetic acid (24 μL) was injected into the mixture and stirred under the room temperature for 1 h. After completion of the reaction, the reaction mixture was filtered through a short pad of silica washed with Et_2O, then extracted with Et_2O, washed by water, brine, dried over Na_2SO_4, and concentrated under vacuum to afford the reaction crude. Which was purified by silica gel column chromatography (*n*-hexane/DCM: 20/1) to afford desired hydrocarbon **10** as a white solid (52.7 mg, yield: 88%), m.p.: 87.3 – 88.1 °C.

^{1}H NMR (500 MHz, $CDCl_3$) δ 7.61 – 7.57 (m, 2H), 7.49 (dd, *J* = 8.3, 1.4 Hz, 2H), 7.45 – 7.38 (m, 2H), 7.34 – 7.29 (m, 1H), 7.29 – 7.20 (m, 6H), 7.20 – 7.13 (m, 1H), 2.15 (q, *J* = 7.3 Hz, 4H), 0.65 (t, *J* = 7.3 Hz, 6H).

^{13}**C NMR** (126 MHz, $CDCl_3$) δ 148.7, 148.0, 141.0, 138.1, 128.8, 128.7, 128.3, 127.8, 127.1, 127.0, 126.4, 125.6, 49.8, 29.3, 8.6.

IR (KBr): 3056, 3022, 2965, 2934, 2871, 1599, 1484, 1444, 1375, 1073, 1008, 824, 760, 735, 695, 597, 505 cm^{-1}

HRMS (ESI) [$C_{23}H_{25}$] $[M+H]^+$ calculated: 301.1956, found: 301.1957.

5. Preliminary Mechanistic Investigations

5.1 Chemoselectivity of organic (pseudo)halides

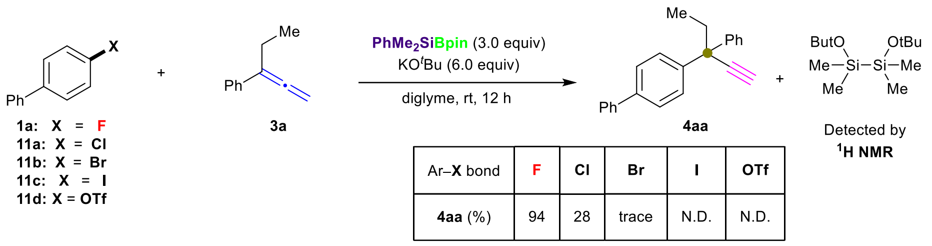

Ar–**X** bond	F	Cl	Br	I	OTf
**4aa** (%)	94	28	trace	N.D.	N.D.

Figure S1. Chemoselectivity of organic (pseudo)halides **1a** or **11** with **3a**.

Following the **General procedure F**, charging **1a** or **11** (0.1 mmol), **3a** (43.2 mg, 0.3 mmol), silylboronate (0.3 mmol), KO*t*Bu (67.2 mg, 0.6 mmol), and then anhydrous diglyme (1.0 mL) sequentially into a flame-dried screw-capped test tube. And the dark blue mixture was then stirred in glovebox at room temperature for 12 h. After that the reaction mixture was quenched by adding water (2.0 mL) while stirring for 5 min, then the organic system was extracted with Et_2O, washed by water, dried over Na_2SO_4, and concentrated under vacuum, followed by 3-fluoropyridine (8.6 µL, 0.1 mmol) as an internal standard. Then ^{1}H NMR analysis of the crude mixture were conducted to show the details of the model reaction.

Note: For the reaction using **11b-d**, after the reaction finished and work-up, the corresponding yield of **4aa** was observed by thin layer chromatography (TLC, eluent: *n*-hexane/DCM: 10/1).

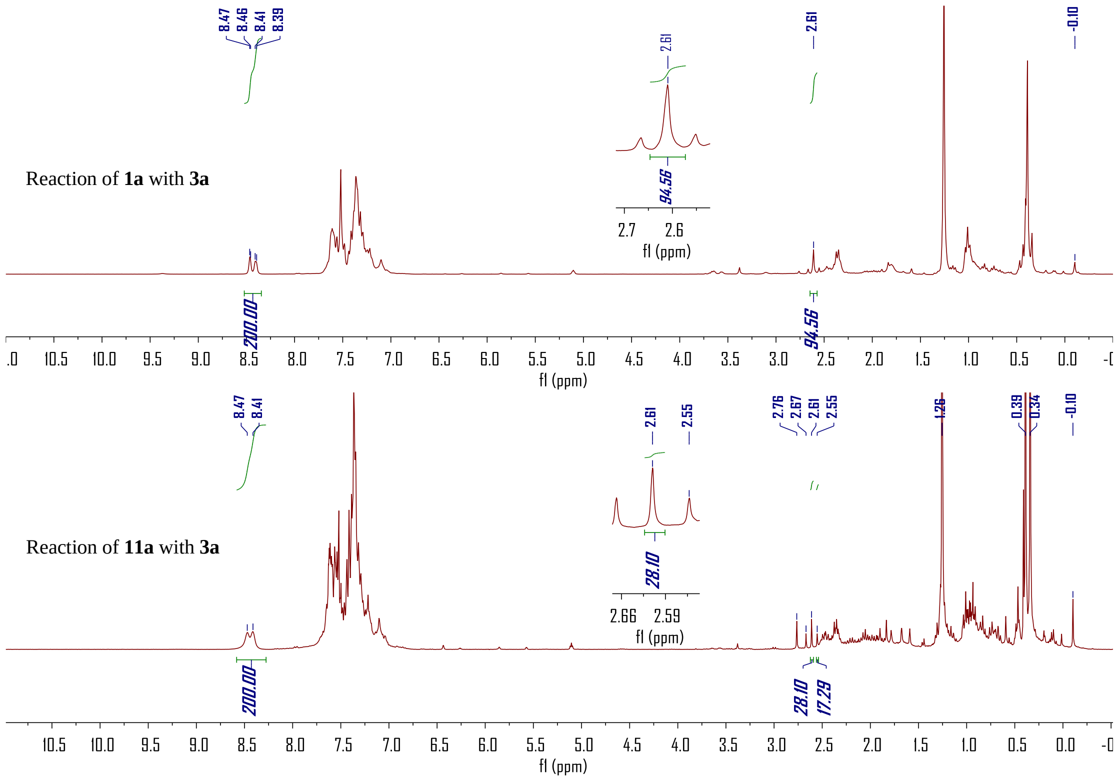

Figure S2. ^{1}H NMR observation of the control experiment of **1a** or **11a** with **3a**.

5.2 The attempt of an alternative process

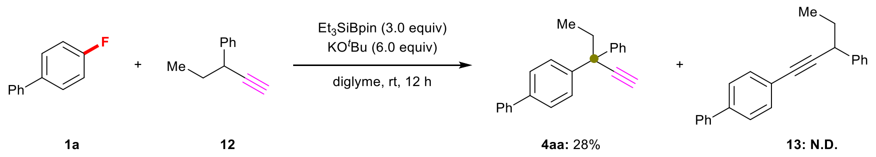

Following the **General procedure F** while with modification, charging KO*t*Bu (33.6 mg, 0.3 mmol), anhydrous diglyme (0.5 mL), $Et_3SiBpin$ (72.6 mg, 0.3 mmol) into a flame-dried screw-capped test tube resulted a yellow-brown mixture, then keep the mixture stirring for 5 min. After that **12** (43.2 mg, 0.3 mmol), **1a** (17.2 mg, 0.1 mmol), KO*t*Bu (33.6 mg, 0.3 mmol) and anhydrous diglyme (0.5 mL) were added sequentially to the mixture. And the dark blue mixture was then sealed and stirred in glovebox at room temperature for 12 h. The reaction tube was diluted with Et_2O (5 mL), then extracted with Et_2O and water, washed with brine, dried over Na_2SO_4, then concentrated under vacuum, followed by adding 3-fluoropyridine (8.6 μL, 0.1 mmol) as an internal standard, then ^{1}H NMR and ^{19}F NMR analysis was taken to show the reaction details (28% yield and 39% conversion of **1a**).

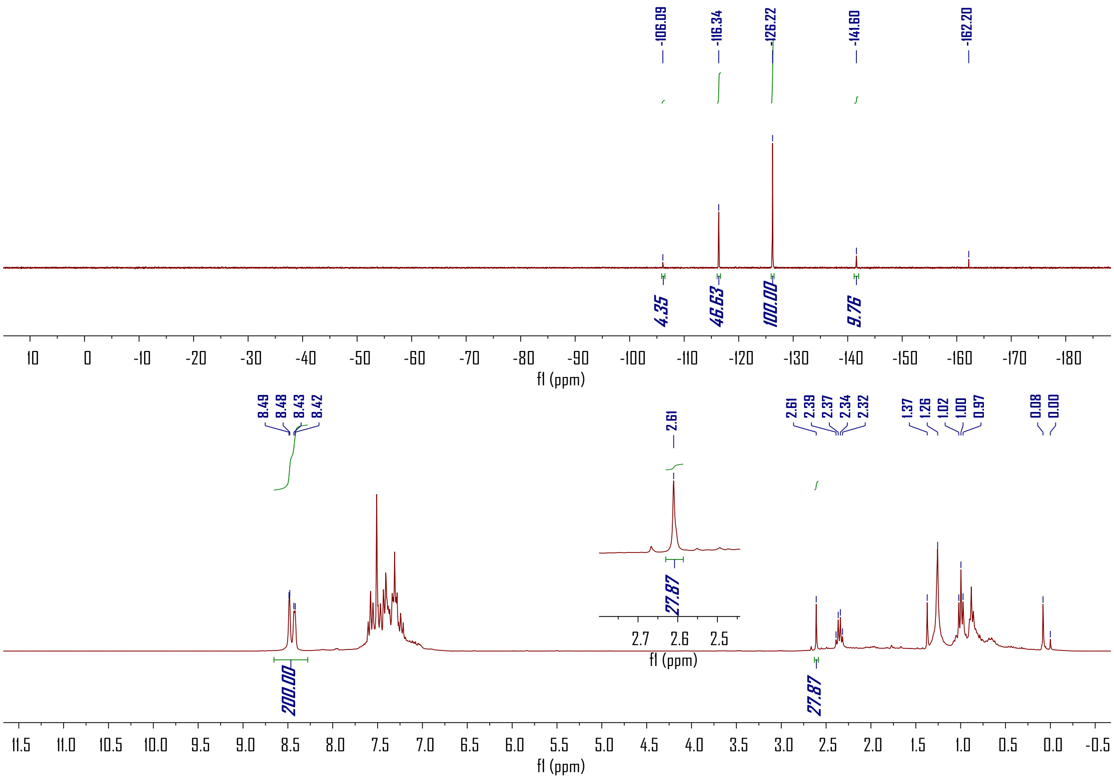

Figure S3. ^{19}F NMR and ^{1}H NMR observation of the control experiment of **1a** with **12**.

5.3 The NMR spectroscopic studies

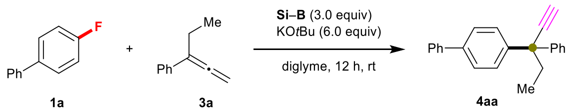

silylboronate (Si-B)	^{1}H NMR Yield of 4aa
$PhMe_2SiBpin$	94%
$Et_3SiBpin$	75%

Figure S4. Evaluation of silylboronate to the model reaction.

Following the **General procedure F**, charging **1a** (17.2 mg, 0.1 mmol), **3a** (43.2 mg, 0.3 mmol), silylboronate (0.3 mmol), KO*t*Bu (67.2 mg, 0.6 mmol), and then anhydrous diglyme (1.0 mL) sequentially into a flame-dried screw-capped test tube. And the dark blue mixture was then stirred in glovebox at room temperature for 12 h. After that the reaction mixture was quenched by adding D_2O (2.0 mL) while stirring for 5 min, then the ^{19}F NMR analysis of the water system were conducted to show the details of the reaction. The organic system was extracted with Et_2O, washed by water, dried over Na_2SO_4, and concentrated under vacuum, followed by 3-fluoropyridine (8.6 μL, 0.1 mmol) as an internal standard. Then the ^{19}F NMR analysis and ^{1}H NMR analysis of the crude mixture were conducted to show the details of the model reaction.

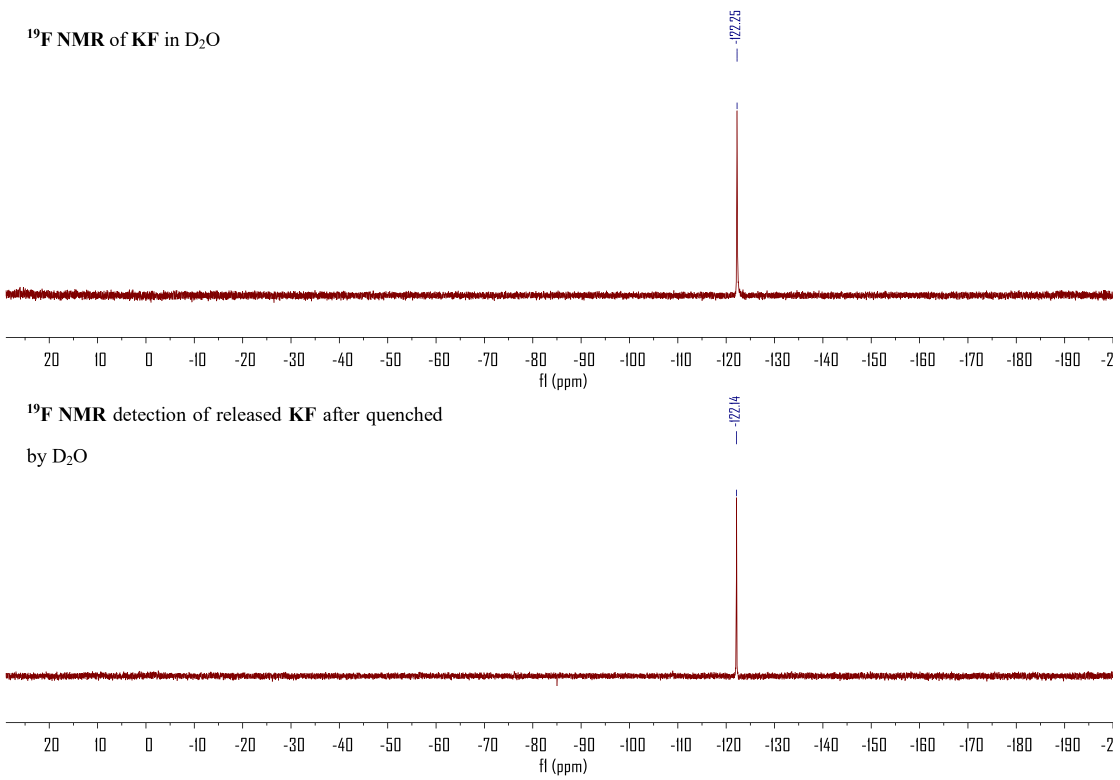

Figure S5. ^{19}F NMR observation of KF in D_2O and KF released in the model reaction.

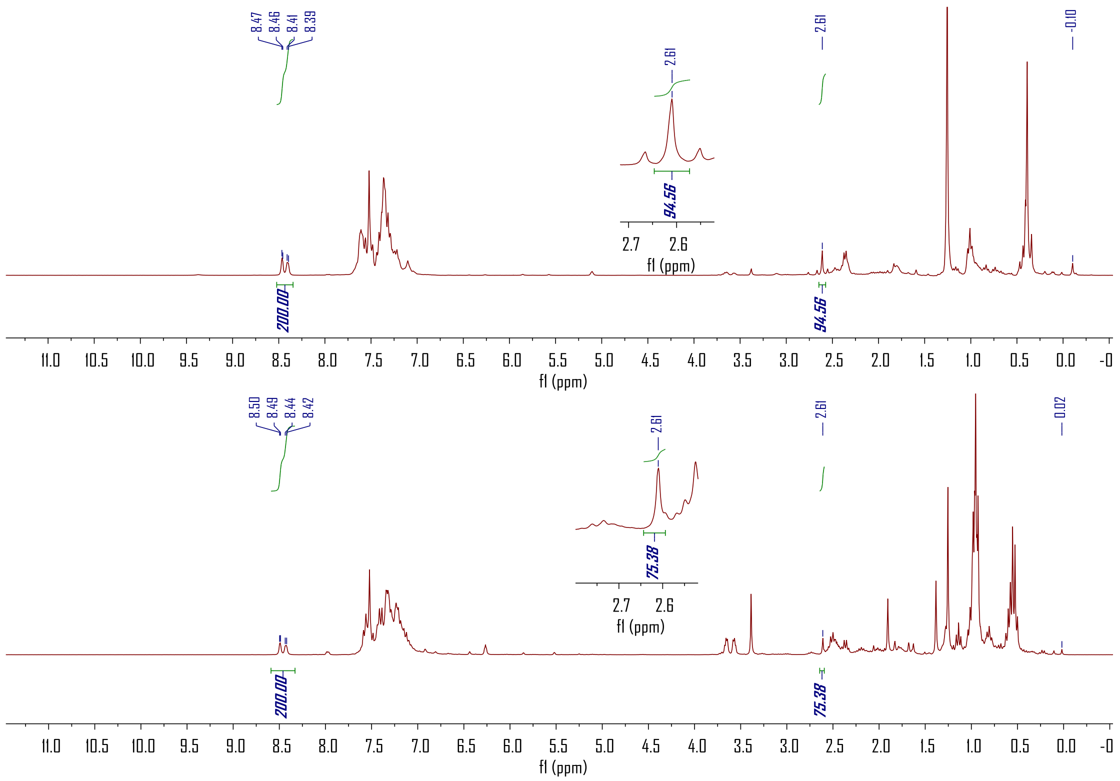

Figure S6. ^{1}H NMR analysis of the model reactions.

5.4 Reaction with radical scavenger TEMPO

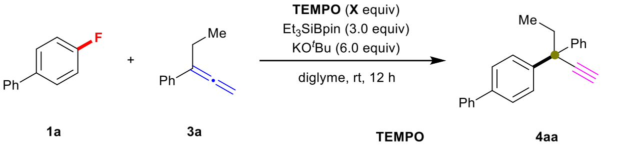

TEMPO	4aa
X = 0	75%
X= 1.0	33%
X = 2.0	13%
X = 4.0	N.D.

Figure S7. Effect of TEMPO to the silylboronate-mediated coupling reaction.

Following the **General procedure F**, charging **1a** (17.2 mg, 0.1 mmol), **3a** (50.0 µL, 0.3 mmol), silyl boronate $Et_3SiBpin$ (72.6 mg, 0.3 mmol), KO*t*Bu (67.2 mg, 0.6 mmol), TEMPO, and then anhydrous diglyme (1.0 mL) sequentially into a flame-dried screw-capped test tube. And then move out from glovebox and stirred at room temperature for 12 h. The reaction tube was diluted with Et_2O (5 mL), then extracted with Et_2O and water, washed with brine, dried over Na_2SO_4, then concentrated under vacuum, followed by adding 3-fluoropyridine (8.6 µL, 0.1 mmol) as an internal standard, then ^1H NMR analysis was taken to show the reaction details.

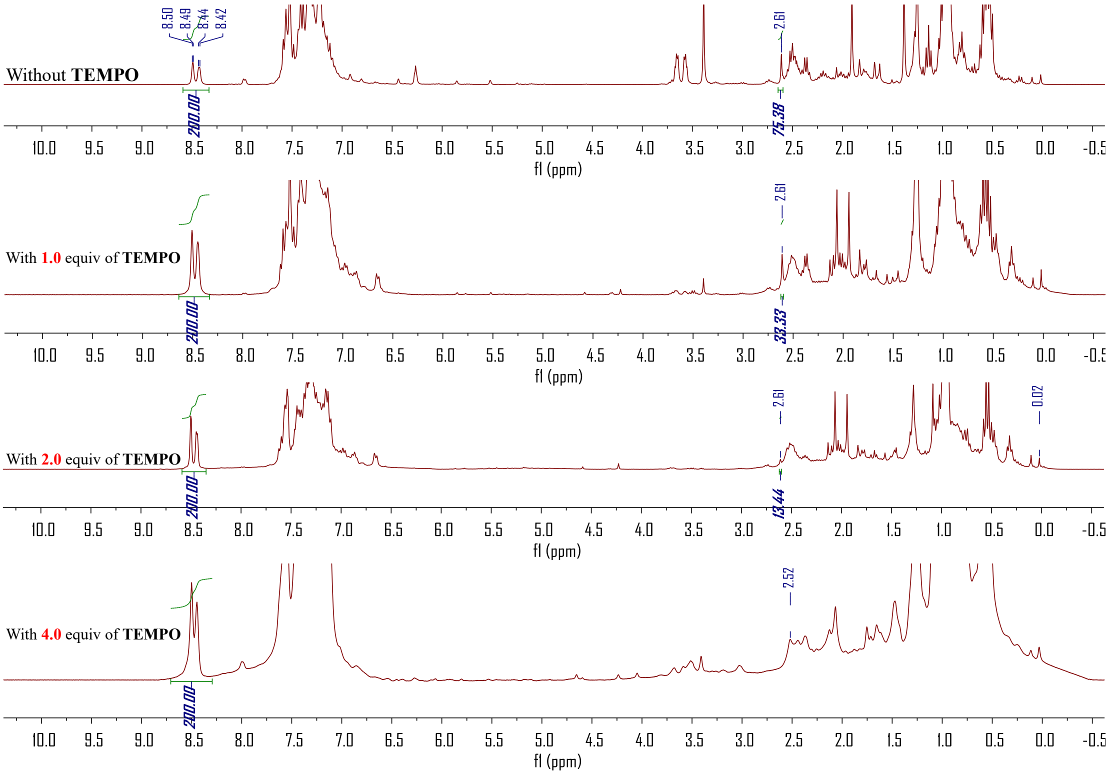

Figure S8. ^1H NMR observation of the effect of TEMPO to the coupling reaction.

Comparably, we attempted two reactions according to the normal procedure but with some changes. Pre-mixed silylboronate and KO^{t}Bu in dry diglyme to generate silyl radical. After stirring for 10 min, the resulting yellow-brown mixture was then added **TEMPO**, **3a**, **1a**, and KO^{t}Bu sequentially. The resulting mixture was allowed to stir at room temperature for 12 h. However, no desired product **4aa** were observed in both reactions, which indicated that the in-situ generated silyl radical should be fully trapped by TMEPO.

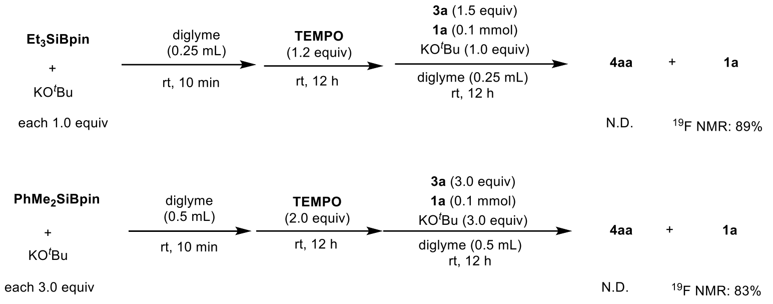

Figure S9. Effect of TEMPO on the different charging sequences by using $Et_3SiBpin$ and $PhMe_2SiBpin$.

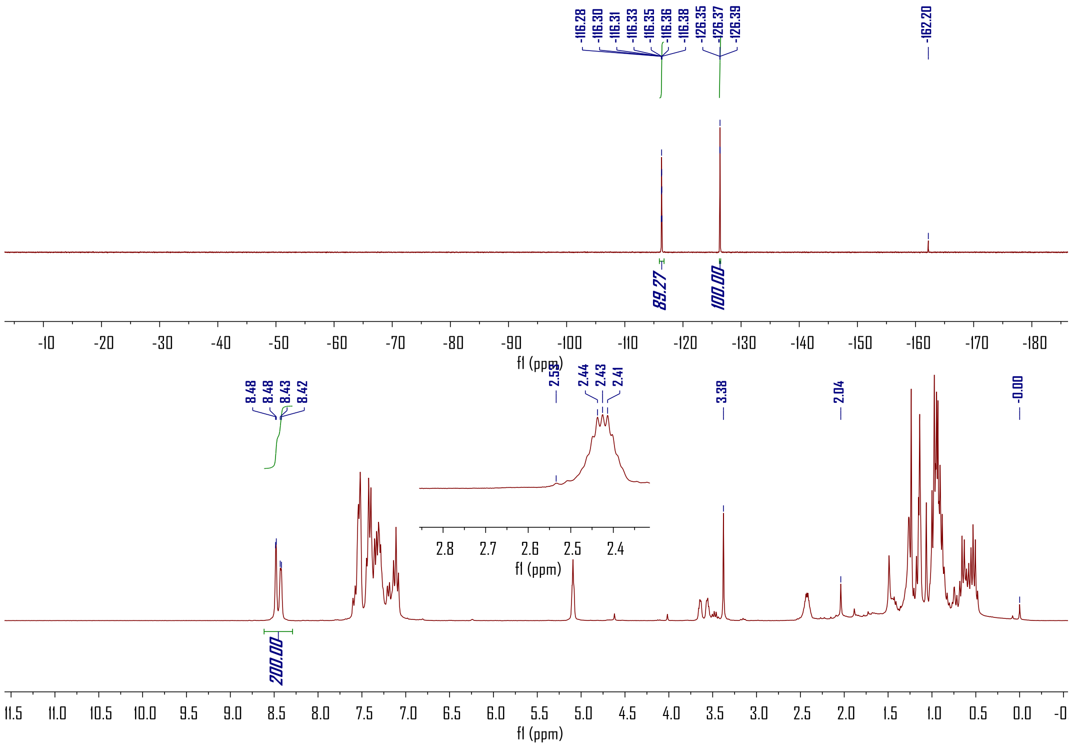

Figure S10. ^{19}F NMR and ^{1}H NMR observation of the effect of TEMPO on the coupling reaction of **1a** with **3a** in the presence of $Et_3SiBpin$.

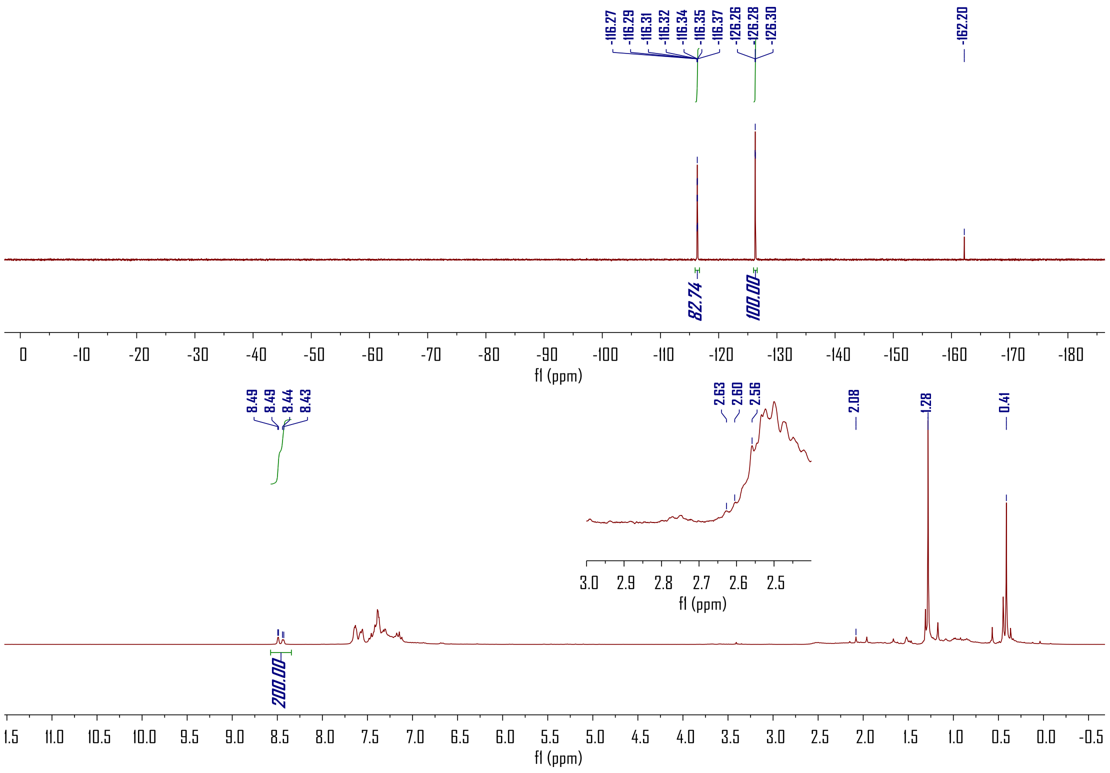

Figure S11. ^{19}F NMR and ^{1}H NMR observation of the effect of TEMPO on the coupling reaction of **1a** with **3a** in the presence of PhMe$_2$SiBpin.

5.5 Radical ring-opening experiment

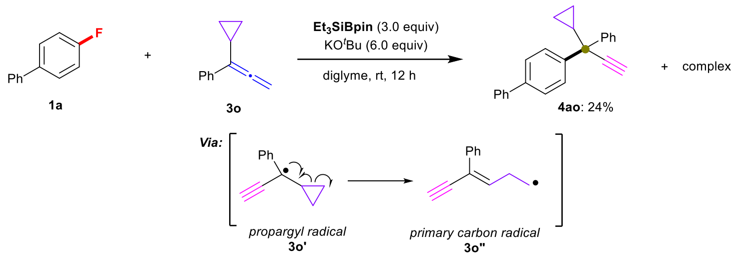

Figure S12. Radical ring-opening experiment of **1a** with **3o**.

Following the **General procedure F**, charging **1a** (17.2 mg, 0.1 mmol), **3o** (46.8 mg, 0.3 mmol), silyl boronate Et$_3$SiBpin (72.6 mg, 0.3 mmol), KO*t*Bu (67.2 mg, 0.6 mmol), and then anhydrous diglyme (1.0 mL) sequentially into a flame-dried screw-capped test tube. And the dark blue mixture was then move out the tube from glovebox and stirred at room temperature for 12 h. The reaction was diluted with Et$_2$O (5 mL), then extracted with Et$_2$O and water, washed with brine, dried over Na$_2$SO$_4$, then concentrated under vacuum, followed by adding 3-fluoropyridine (8.6 μL, 0.1 mmol) as an internal standard, then ^{1}H NMR analysis was taken to show the reaction details.

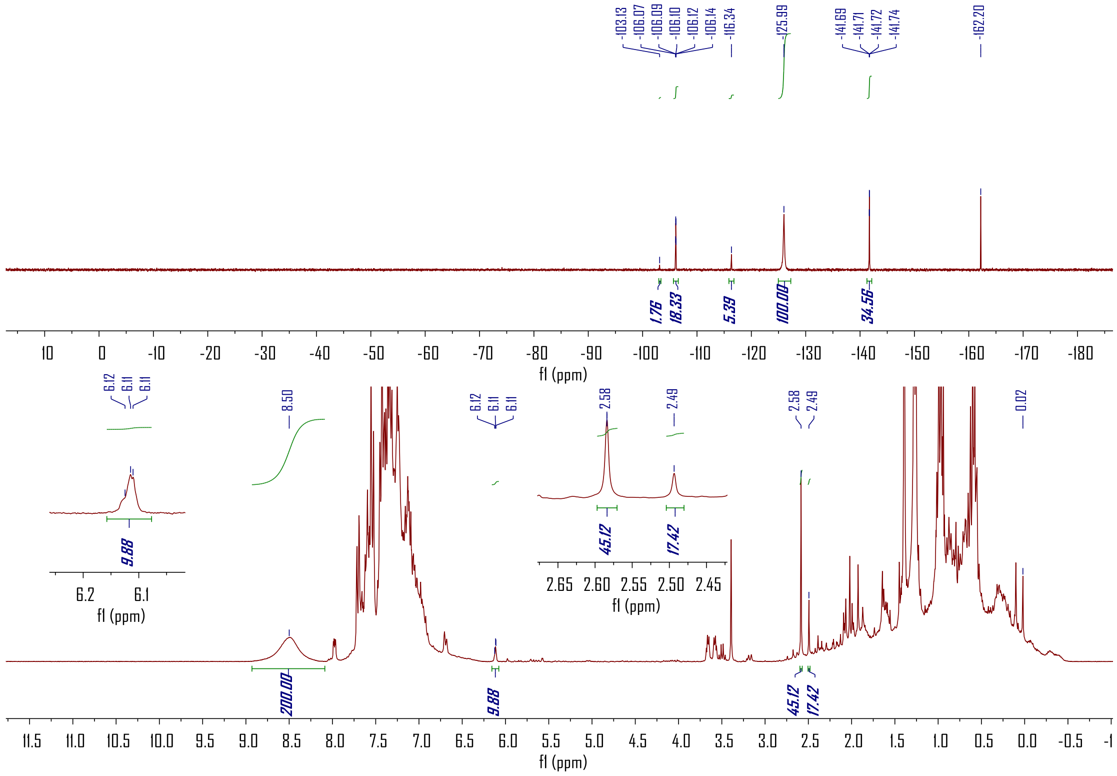

Figure S13. ^{19}F NMR and ^{1}H NMR observation of the radical ring-opening reaction.

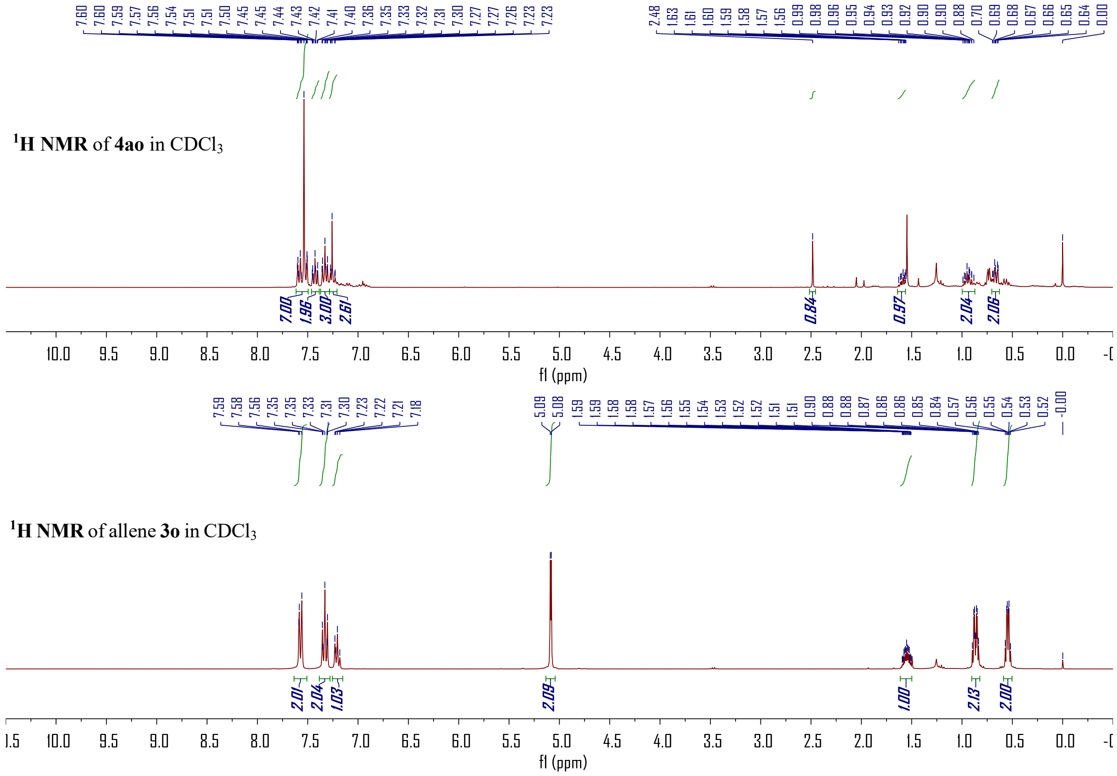

Figure S14. ^{1}H NMR spectrum comparison of product **4ar** with allene **3o**.

5.6 Control experiments by using deuterated allenes

The reaction of 1a and d^2-3a

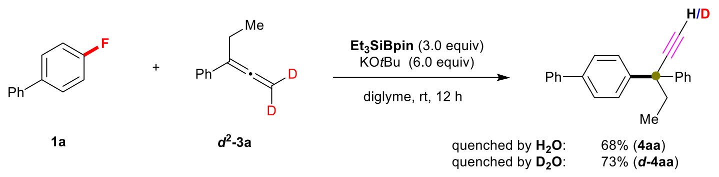

Figure S15. Model reaction of **1a** and ***d*²-3a** quenched by H_2O or D_2O.

Following the **General procedure F**, charging **1a** (17.2 mg, 0.1 mmol), ***d*²-3a** (43.8 mg, 0.3 mmol), silyl boronate $Et_3SiBpin$ (72.6 mg, 0.3 mmol), KO*t*Bu (67.2 mg, 0.6 mmol), and then anhydrous diglyme (1.0 mL) sequentially into a flame-dried screw-capped test tube. And the dark blue mixture was then move out from glovebox and stirred at room temperature for 12 h. The reaction tube was diluted with Et_2O (5 mL), and water (3 mL) or D_2O (3 mL) while stirring for 10 min, then extracted with Et_2O, washed with brine, dried over Na_2SO_4, filtered and concentrated *in vacuo*. The residue was purified by silica gel column chromatography (*n*-hexane/DCM: 10/1) to afford corresponding product **4aa** (20.2 mg, yield: 68%) or ***d*-4aa** (21.7 mg, yield: 73%). The deuterated product ***d*-4aa** was not observed might be due to the hydrogen–deuterium (H/D) exchange process when quenched by water under strong base conditions.

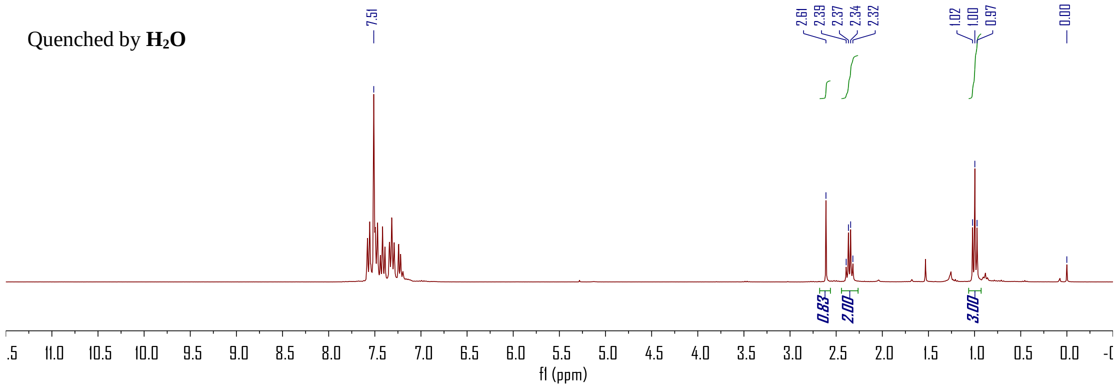

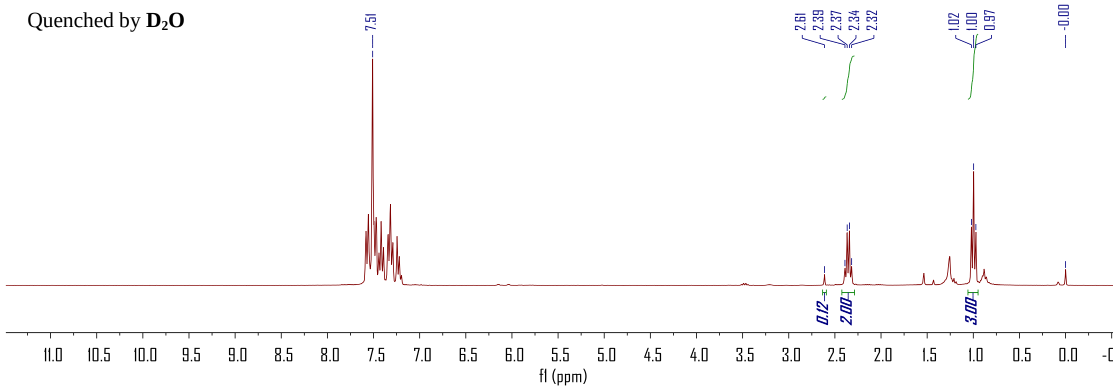

Figure S16. ¹H NMR observation of the products of **1a** and ***d*²-3a** quenched by H_2O or D_2O.

The reaction of 1a and d^2-3g

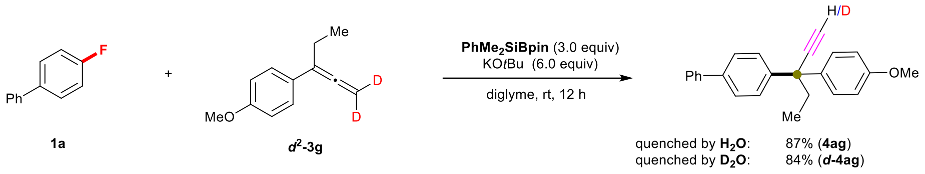

Figure S17. Model reaction of **1a** and ***d*²-3g** quenched by H_2O or D_2O.

Following the **General procedure F**, charging **1a** (17.2 mg, 0.1 mmol), ***d*²-3g** (52.8 mg, 0.3 mmol), silyl boronate $PhMe_2SiBpin$ (78.6 mg, 0.3 mmol), KO*t*Bu (67.2 mg, 0.6 mmol), and then anhydrous diglyme (1.0 mL) sequentially into a flame-dried screw-capped test tube. And the dark blue mixture was then move out from glovebox and stirred at room temperature for 12 h. The reaction tube was diluted with Et_2O (5 mL), and water (3 mL) or D_2O (3 mL) while stirring for 10 min, then extracted with Et_2O, washed with brine, dried over Na_2SO_4, filtered and concentrated *in vacuo*. The residue was purified by silica gel column chromatography (*n*-hexane/DCM: 5/1) to afford corresponding product **4ag** (28.3 mg, yield: 87%) or ***d*-4ag** (27.4 mg, yield: 84%). The deuterated product ***d*-4ag** was not observed might be also due to the H/D exchange process when quenched by water under strong base conditions.

Quenched by $\mathbf{H_2O}$

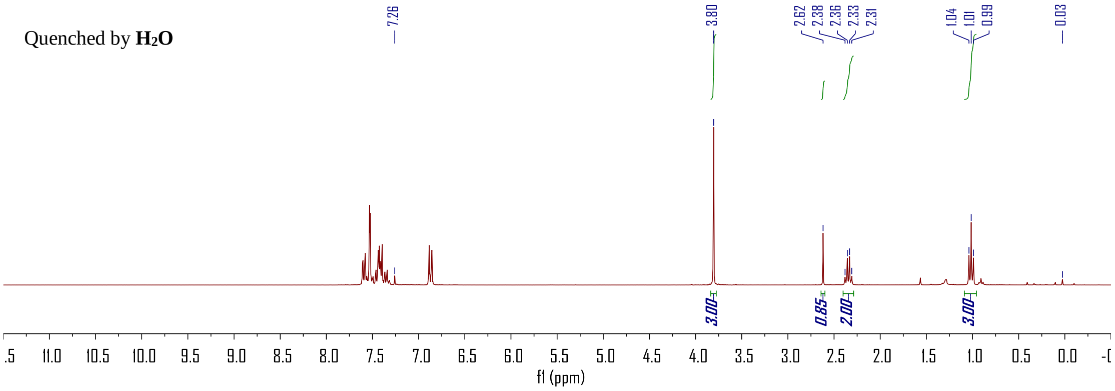

Quenched by $\mathbf{D_2O}$

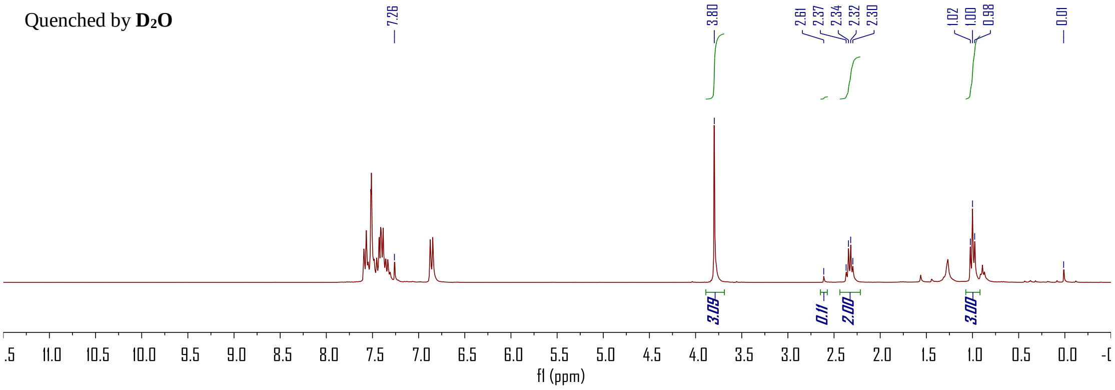

Figure S18. ¹H NMR observation of the products of **1a** and ***d*²-3g** quenched by H_2O or D_2O.

The reaction of 1a and *d*-3g

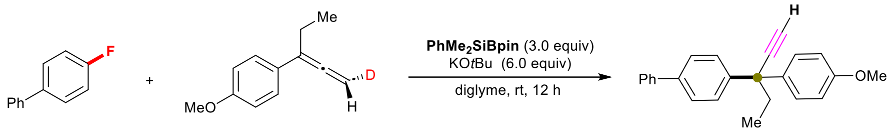

Figure S19. Model reaction of **1a** and ***d*-3g** quenched by ice H_2O.

Following the **General procedure F**, charging **1a** (17.2 mg, 0.1 mmol), ***d*-3g** (52.8 mg, 0.3 mmol), silyl boronate $PhMe_2SiBpin$ (78.6 mg, 0.3 mmol), KO*t*Bu (67.2 mg, 0.6 mmol), and then anhydrous diglyme (1.0 mL) sequentially into a flame-dried screw-capped test tube. And the dark blue mixture was then move out from glovebox and stirred at room temperature for 12 h. The reaction was poured into ice water (30 mL) while stirring for 10 min, then extracted with Et_2O, washed with brine, dried over Na_2SO_4, filtered and concentrated *in vacuo*. The residue was purified by silica gel column chromatography (*n*-hexane/DCM: 5/1) to afford corresponding product **4ag** (30.0 mg, yield: 92%).

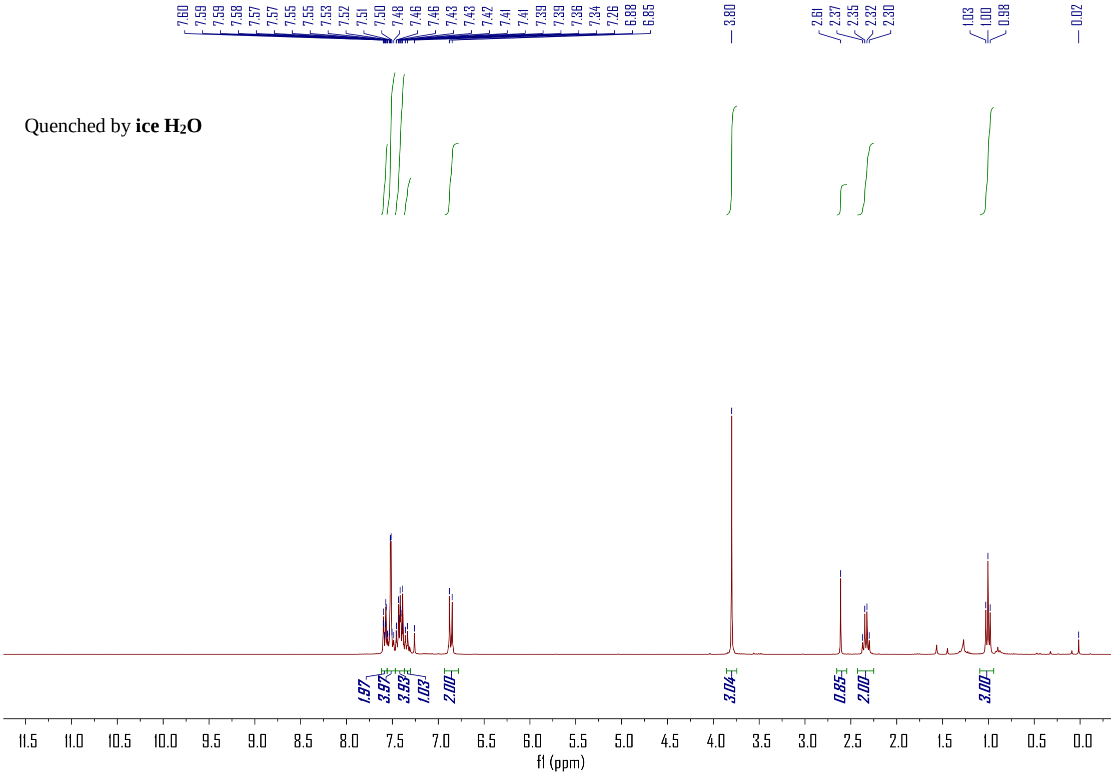

Figure S20. ^{1}H NMR observation of the product of **1a** and ***d*-3g** quenched by ice H_2O.

5.7 ESR experiment

Electron spin resonance (ESR) was performed on a JEOL FA200 ESR spectrometer. ESR spectra were obtained at a microwave power level of 0.0997~0.998 mW and 100 kHz filed modulation at room temperature (~288 K). The magnetic field was calibrated with the well-known splitting constants of Mn^{2+} in MgO. The *g*-values were determined by comparison with the spectrum of Mn^{2+} in MgO. Tri-*tert*-butyl nitrosobenzene (TTBNB, Aldrich) was purified by sublimation under reduced pressure and was used as spin-trapping reagent in this experiment.

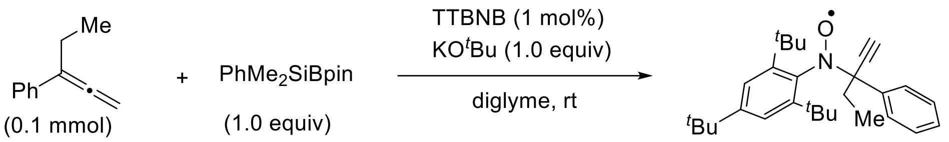

Figure S21. ESR experiment of **3a** in the presence of TTBNB.

In a N_2 filled glovebox, to a flame-dried screw-capped test tube was added KO^{t}Bu (11.2 mg, 0.1 mmol), dry diglyme (0.25 mL), and silyl boronates $PhMe_2SiBpin$ (26.2 mg, 0.1 mmol), the resulting light-yellow mixture was stirring for 2 minutes followed by the addition of allene **3a** (16.7 μL, 0.1 mmol), dry diglyme (0.25 mL) sequentially. Stir the mixture till it turns to deep blue. Then 100 μL of TTBNB (0.01 M in diglyme) solution was added, dilute the mixture to 1.0 mL by adding diglyme (0.5 mL). Transfer 30 μL of the mixture into a capillary tube that furtherly be put in the ESR test tube, the tube then was sealed and removed from the glovebox for conducting ESR measurement.

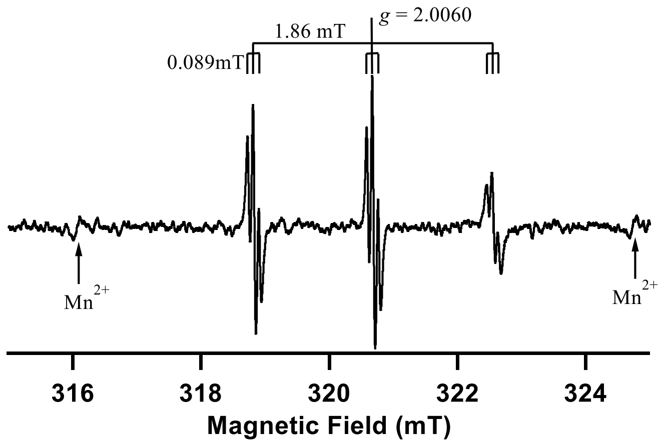

Figure S22. ESR spectra of spin-adduct.

ESR experiment was carried out to confirm the generation of radicals under the optimized conditions using the spin trapping method with TTBNB. The ESR spectrum (triple–triplet) for the reaction of $PhMe_2SiBpin$, KO^{t}Bu and allene **3a** in diglyme at room temperature corresponded to that of the spin adduct of the *tert*-propargyl radical (•CEtPhC≡CH) trapped by TTBNB. The hyperfine splitting (*hfs*) constant due to nitrogen (A_N; spin quantum number I = 1) was 1.86 mT, and the small splitting constant due to the two hydrogens at the *meta*- position of the TTBNB benzene ring (A_{Hm}; I = 1/2) was 0.089 mT. The *g*-value of 2.006 was assigned to a nitroxide-type radical.[28]

6. Unsuccessful Substrates

Unsuccessful aryl halide substrates:

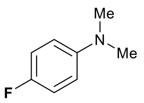

1ab: complex mixture

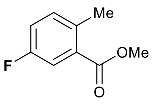

1ac: complex mixture

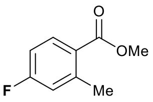

complex mixture

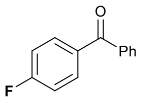

complex mixture

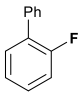

less reactive

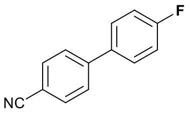

complex mixture

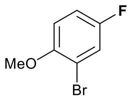

complex mixture

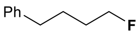

2i: less reactive

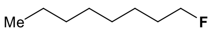

2j: less reactive

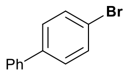

11b: complex mixture

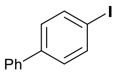

11c: complex mixture

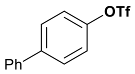

11d: complex mixture

Figure S23. Unsuccessful substrate of organic fluorides.

Unsuccessful allene substrates:

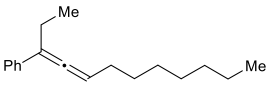

3p: less reactive

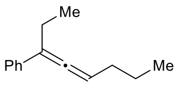

3q: less reactive

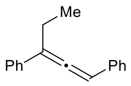

3r: complex mixture

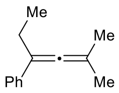

3s: complex mixture

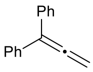

3t: complex mixture

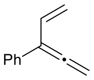

3u: complex mixture

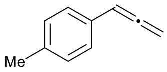

3v: complex mixture

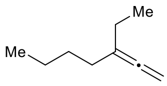

3w: less reactive

Figure S24. Unsuccessful substrate of allenes.

7. Copies of NMR Data (^{1}H NMR, ^{13}C NMR and ^{19}F NMR)

1-Fluoro-3-phenoxybenzene (1d)

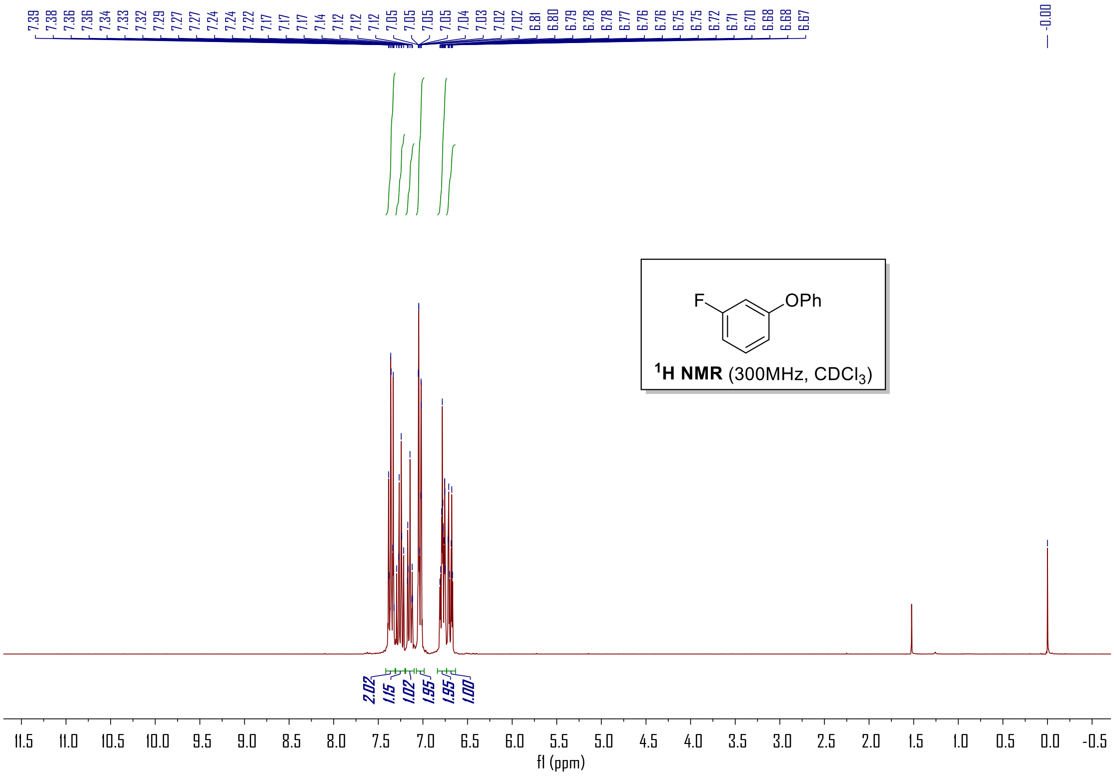

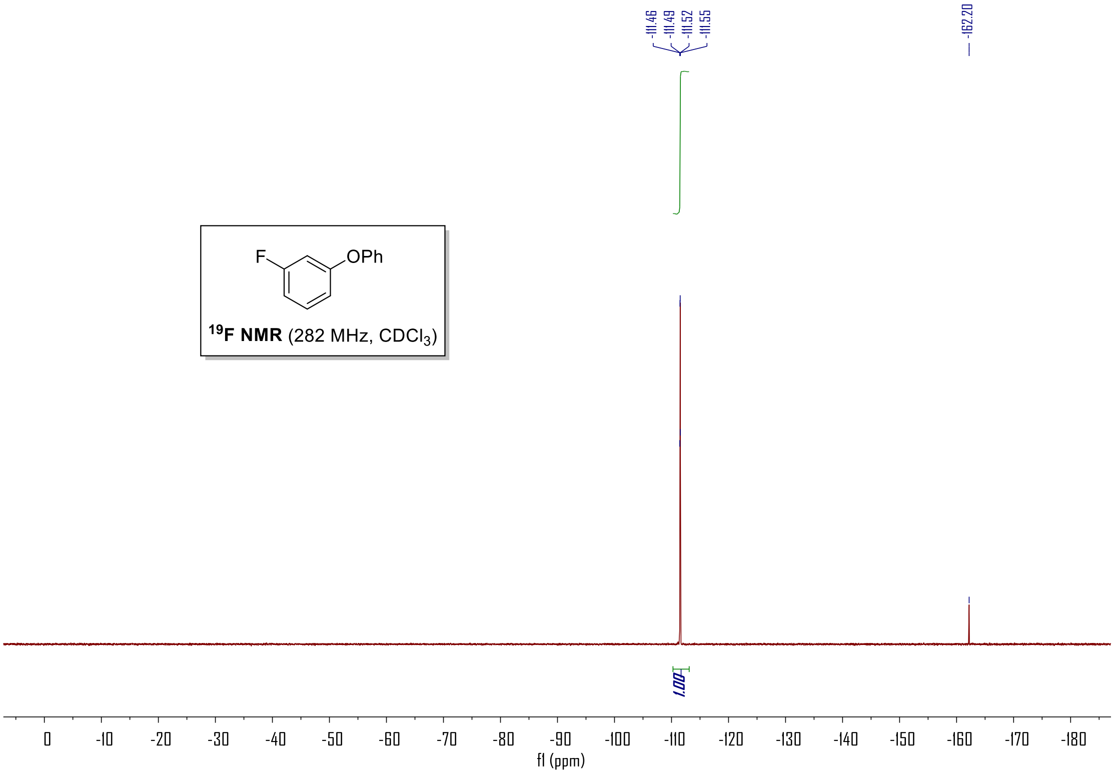

1-Fluoro-4-phenoxybenzene (1e)

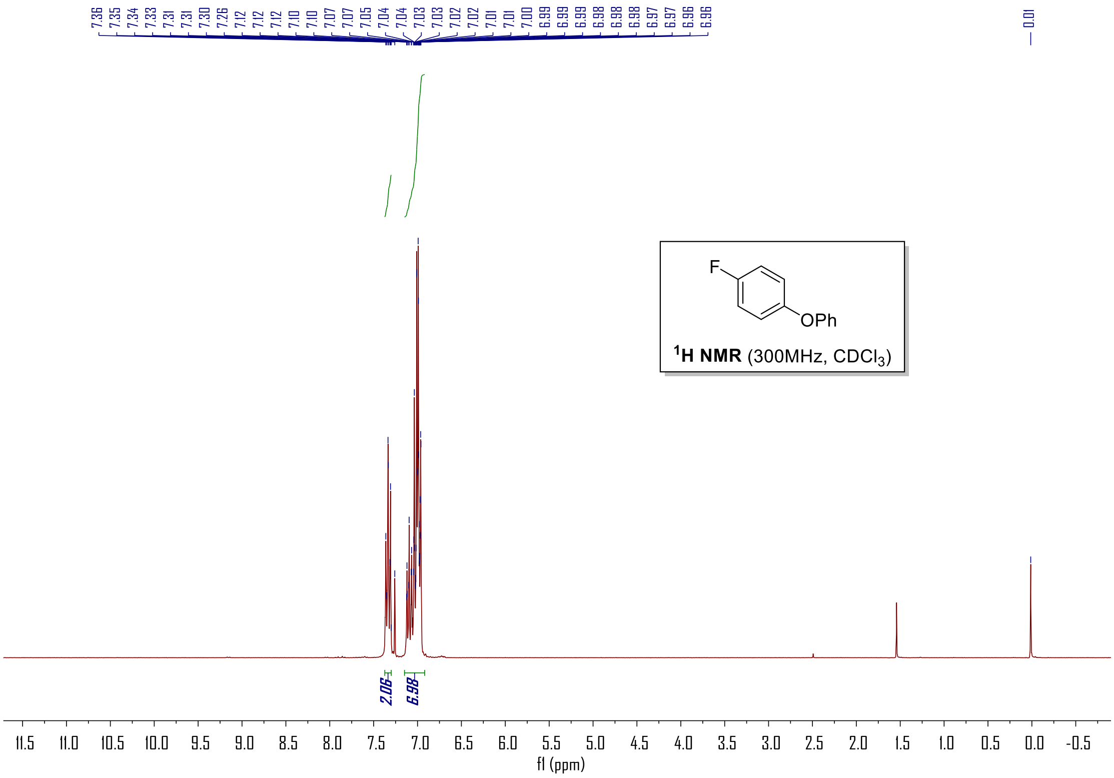

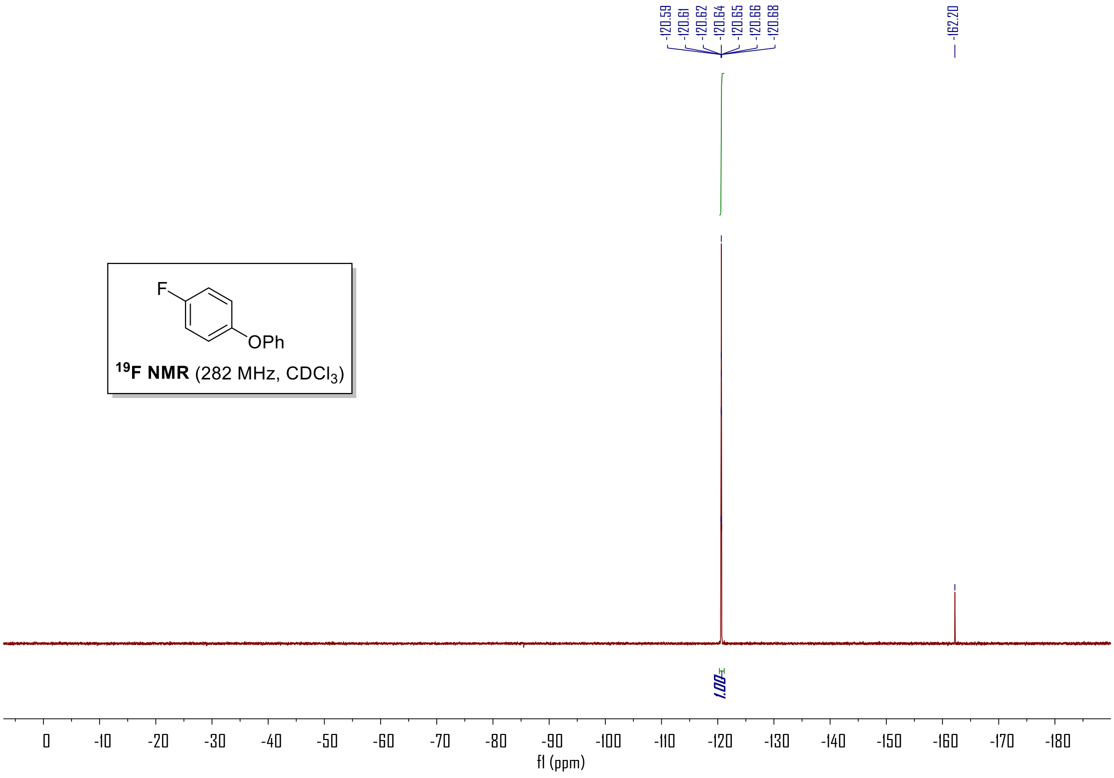

2-Fluorobenzofuran (1u)

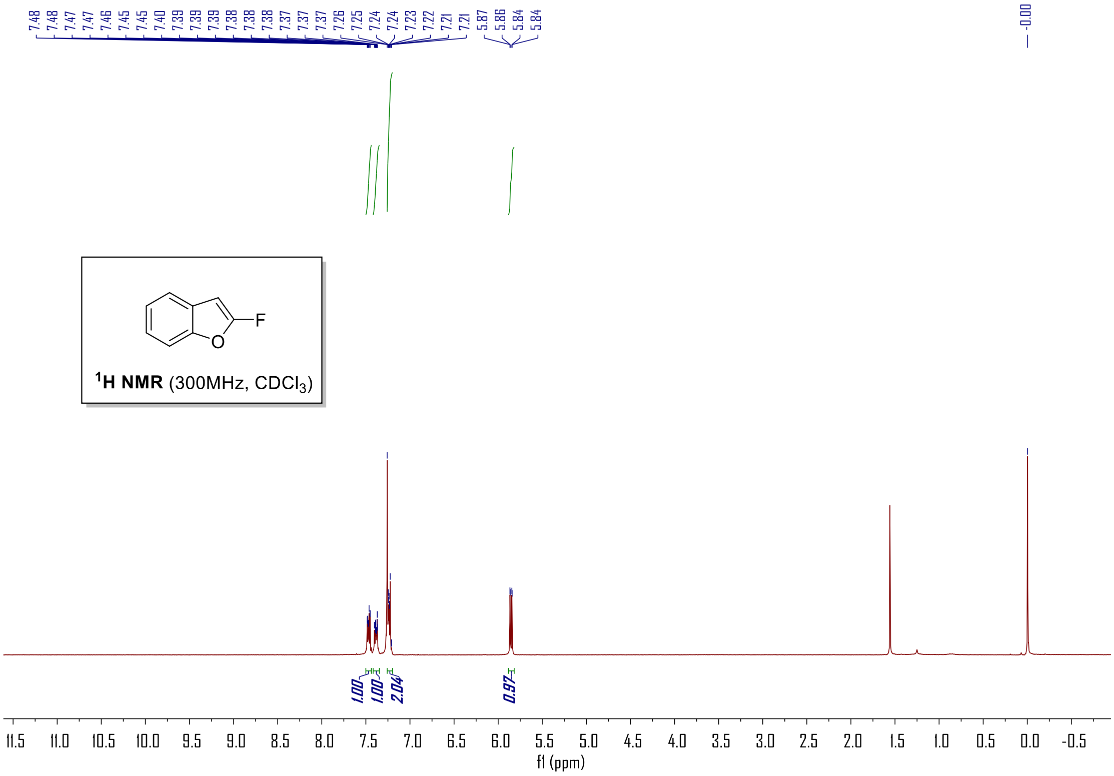

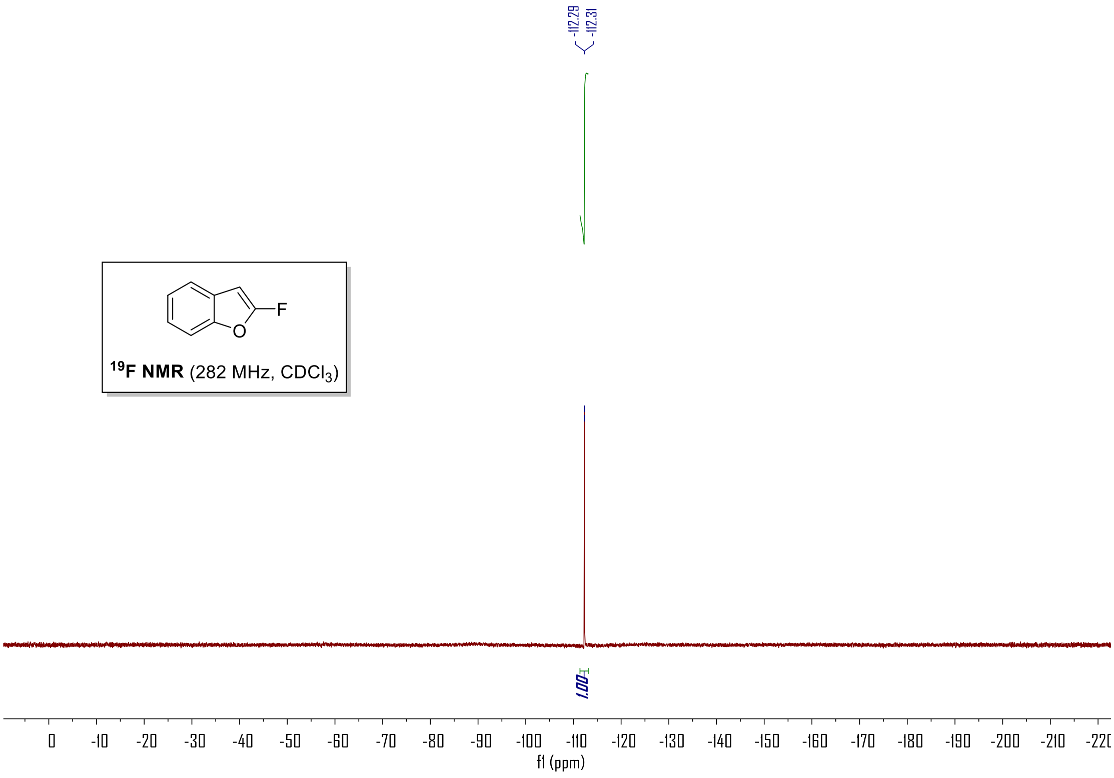

1-(5-(6-Fluoronaphthalen-2-yl)-2-methoxyphenyl)adamantane (1ad)

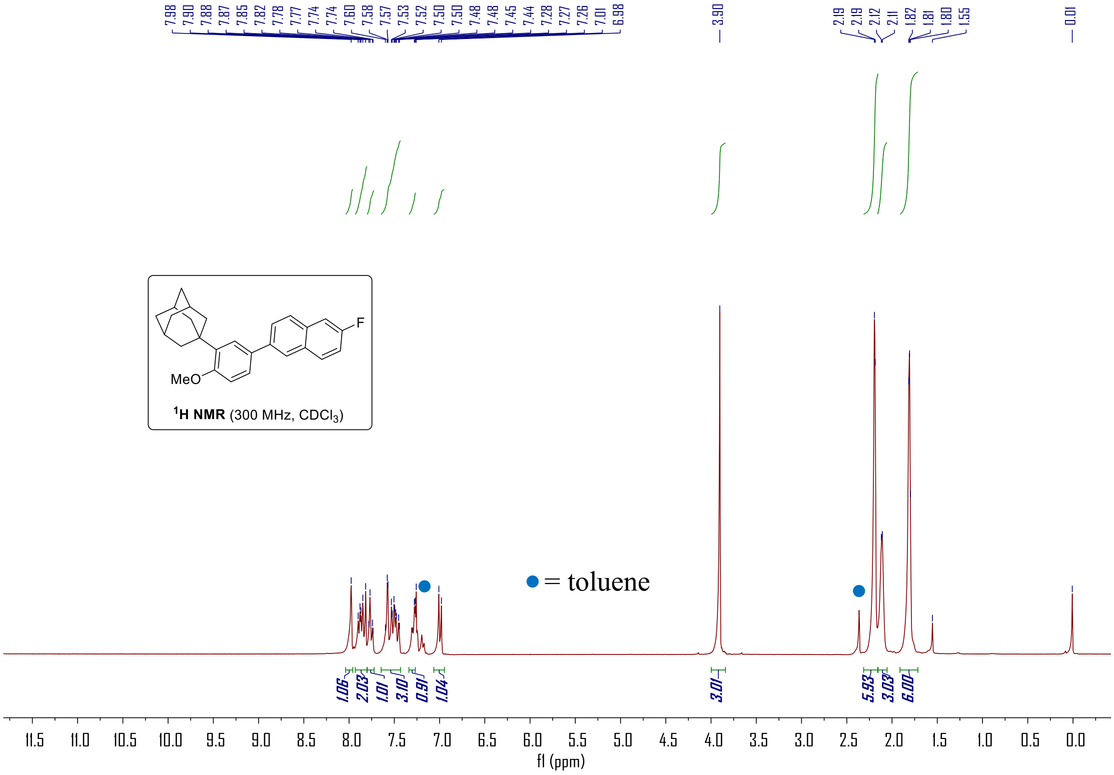

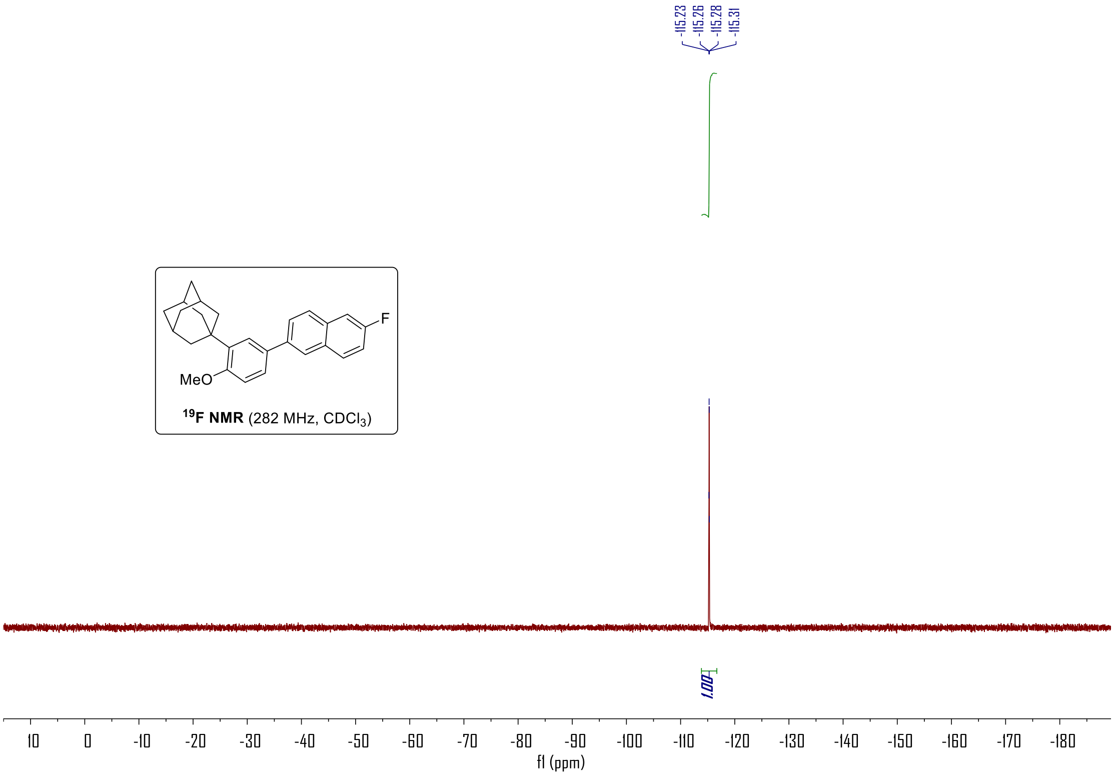

Blonanserin (1af)

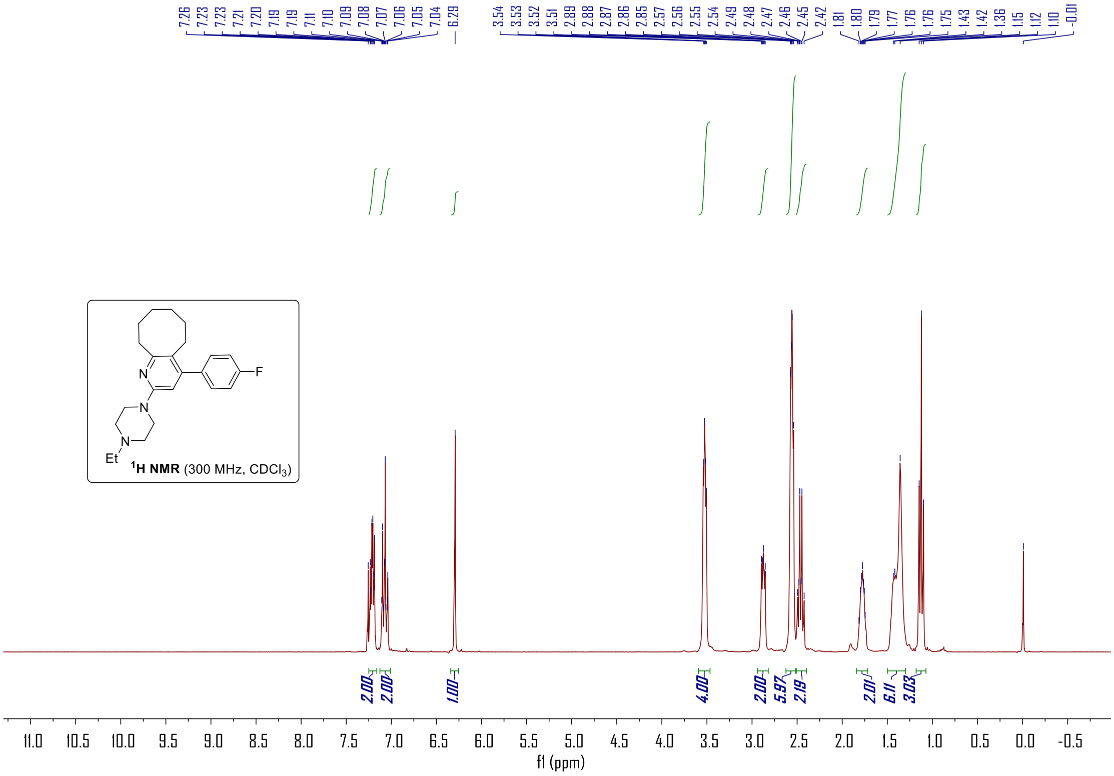

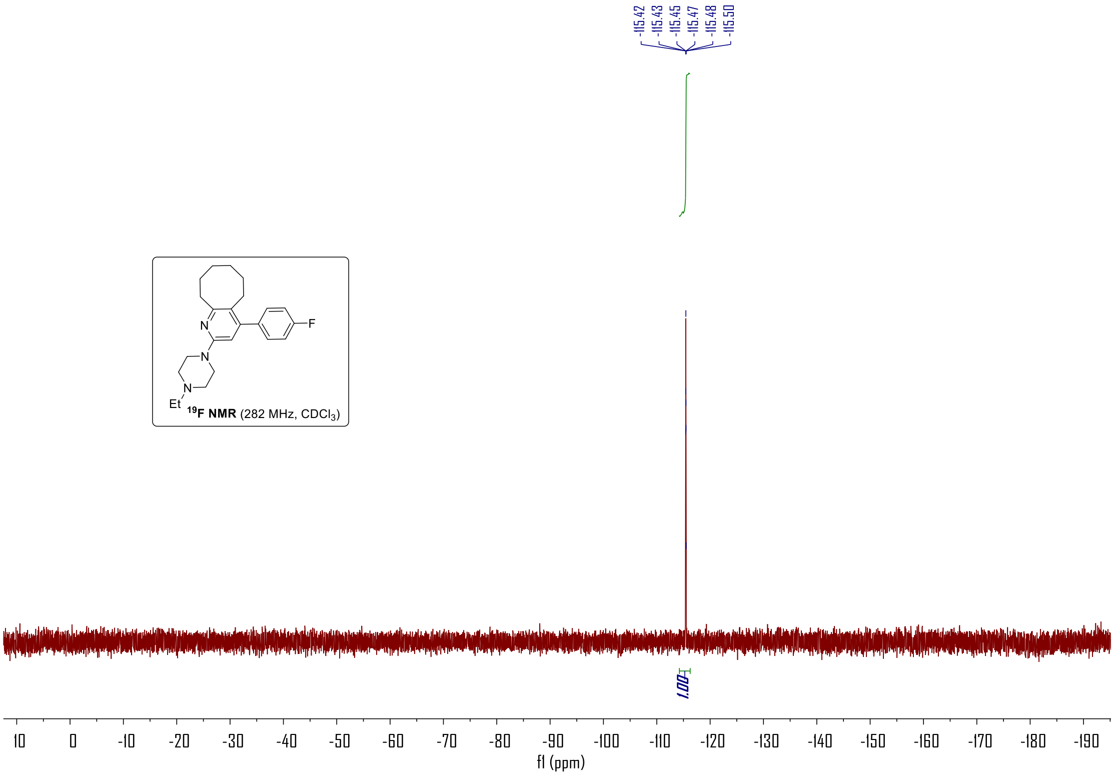

1-Chloro-4-(fluoromethyl)benzene (2c)

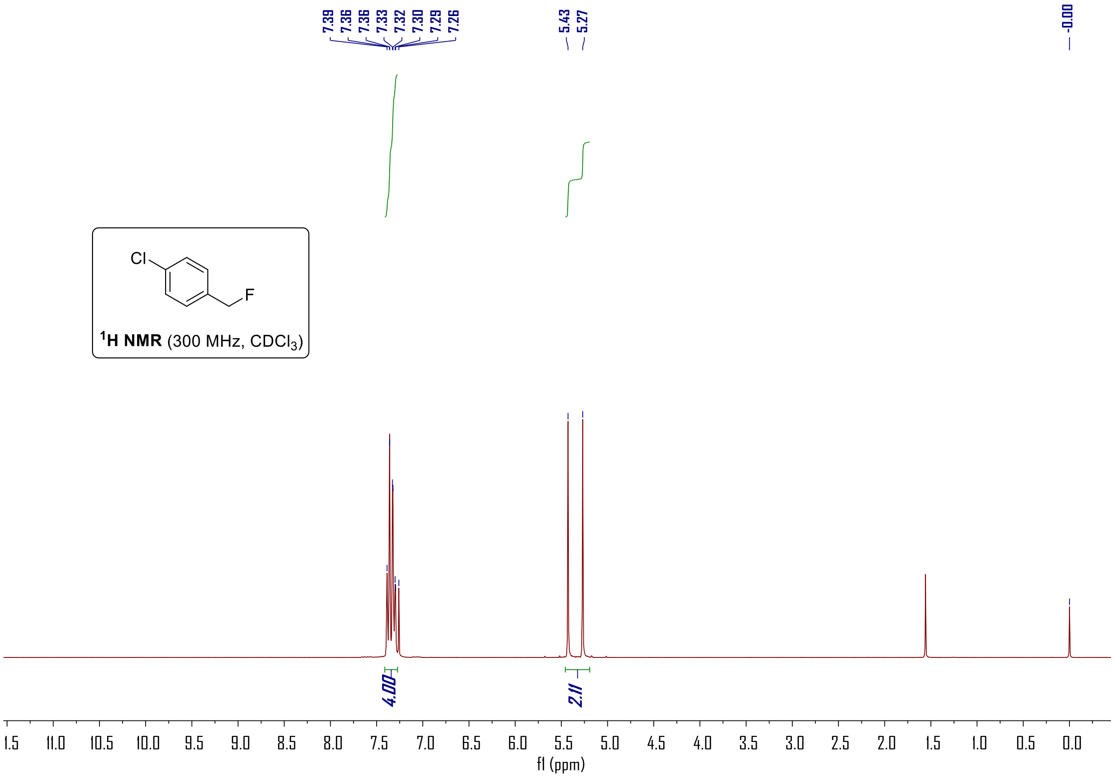

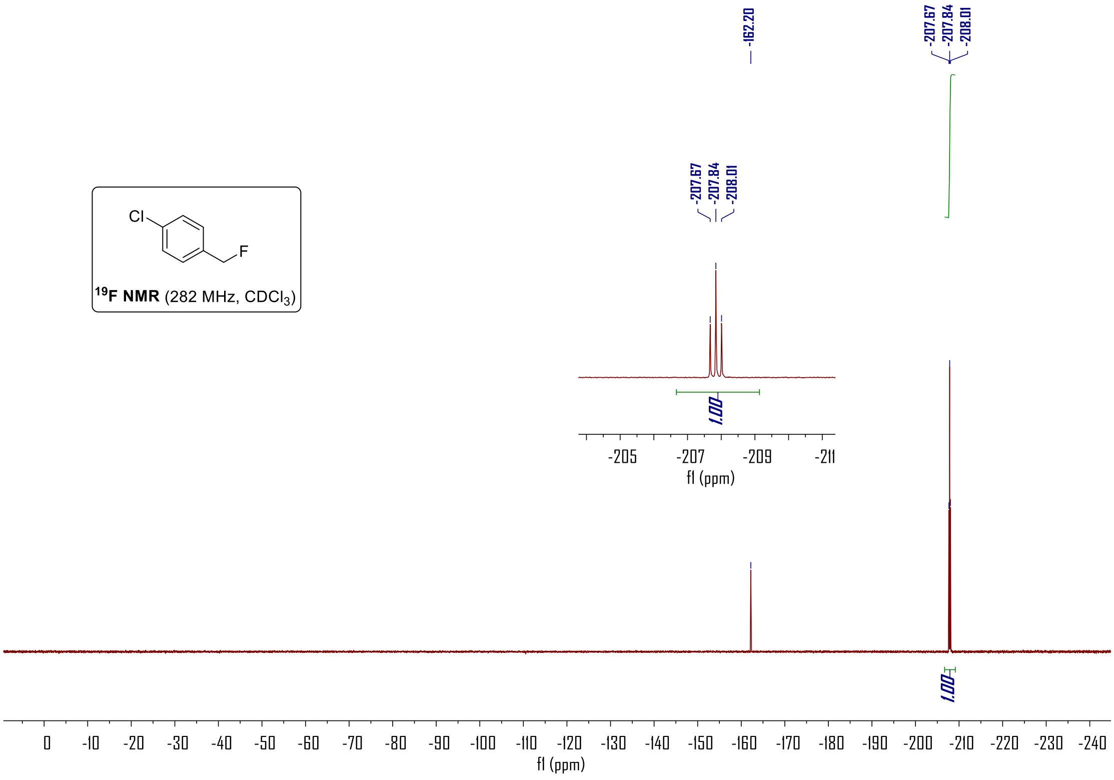

1-Bromo-4-(fluoromethyl)benzene (2d)

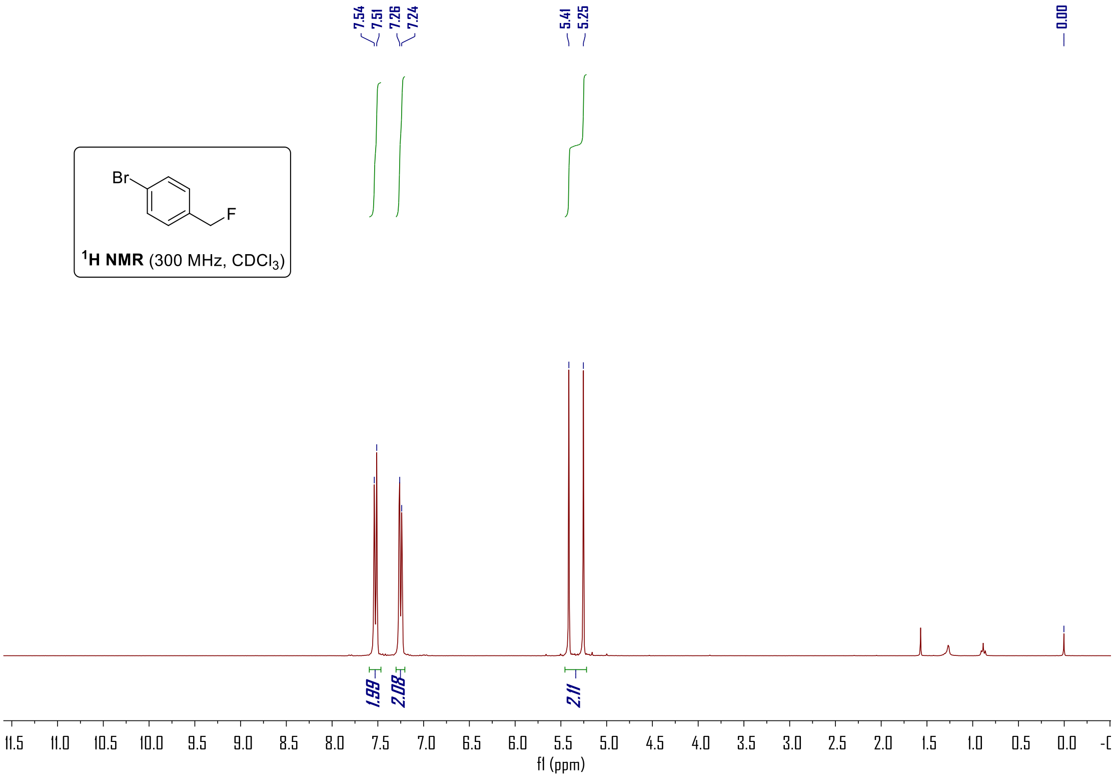

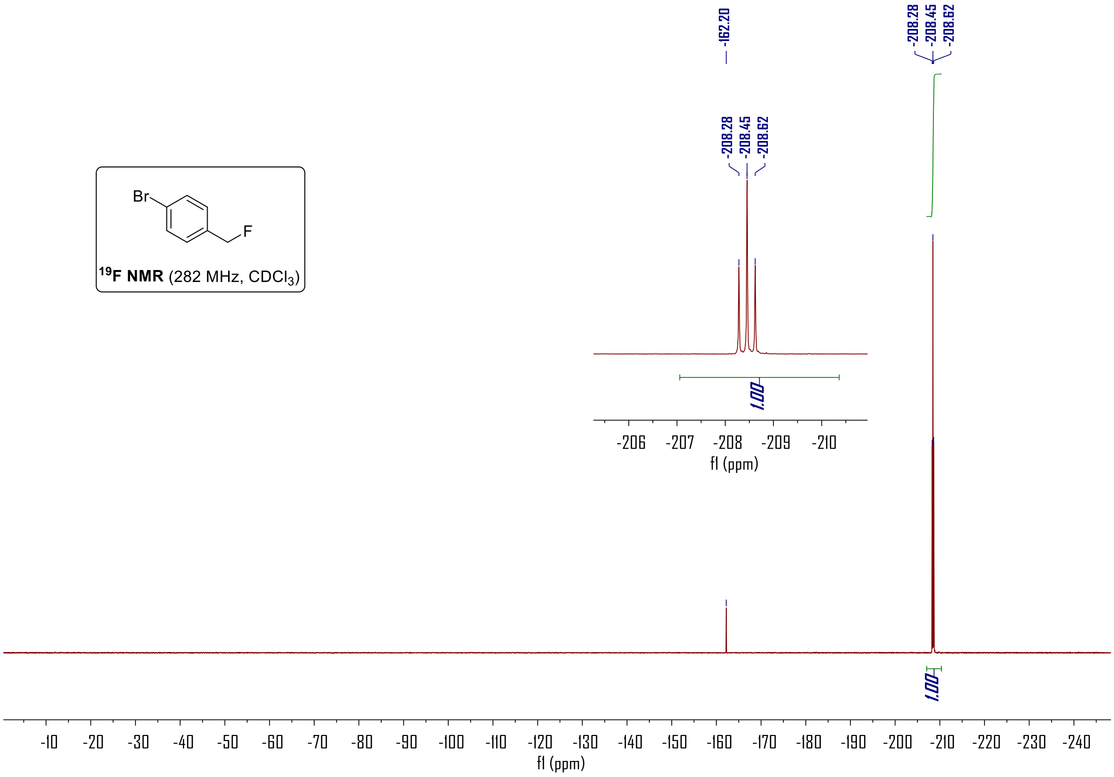

1-(*tert*-Butyl)-4-(fluoromethyl)benzene (2e)

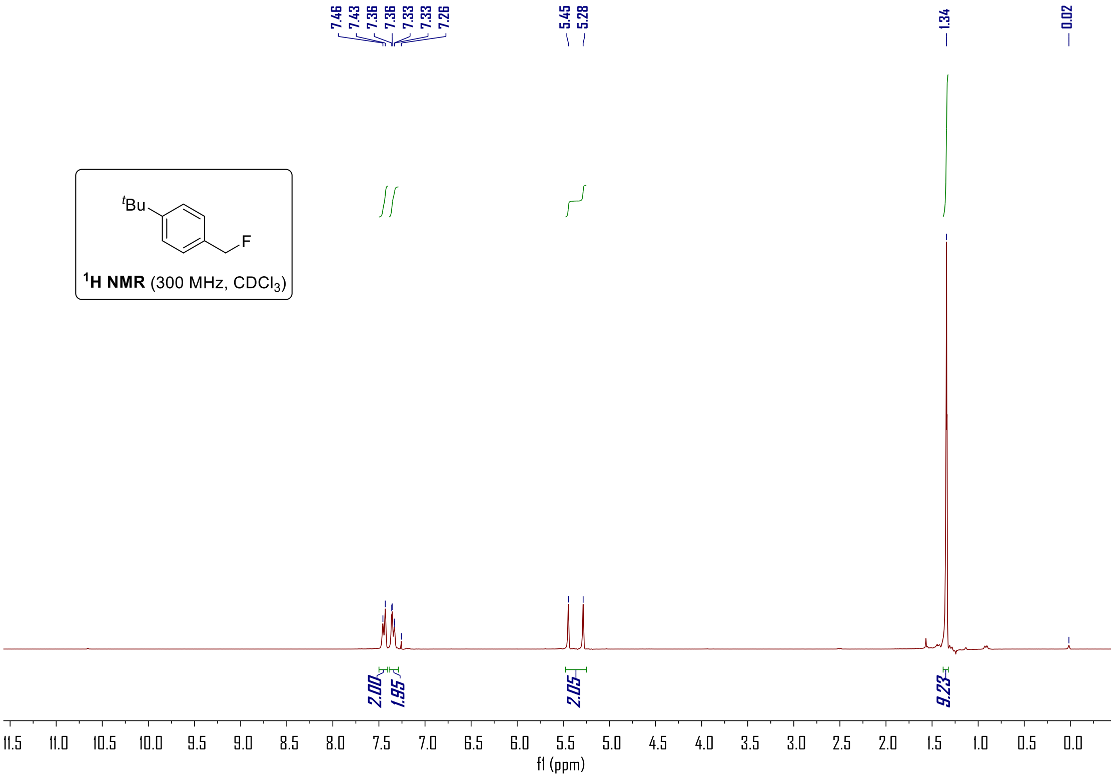

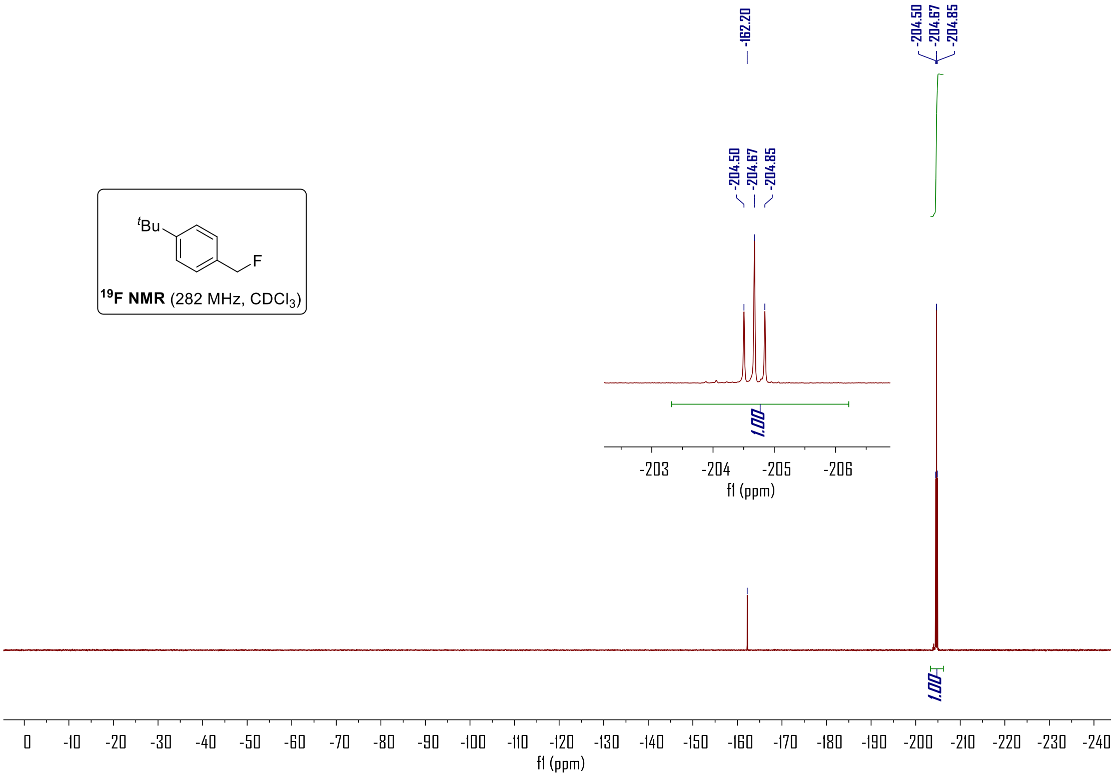

2-(Fluoromethyl)naphthalene (2f)

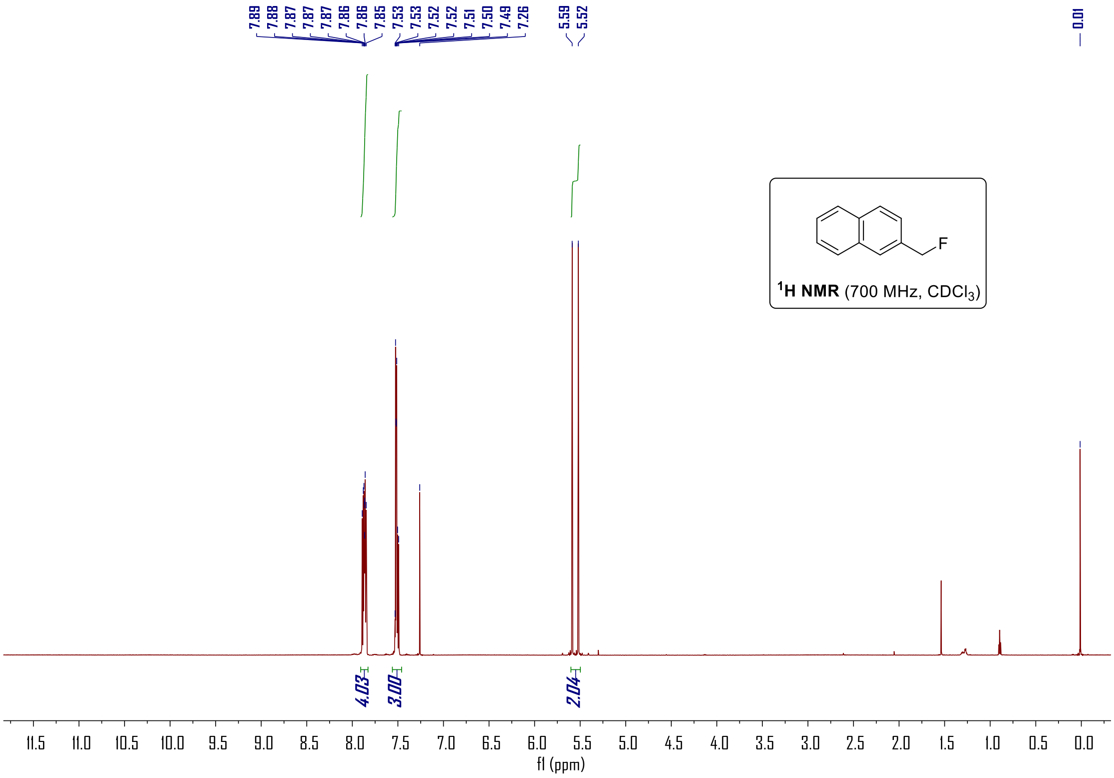

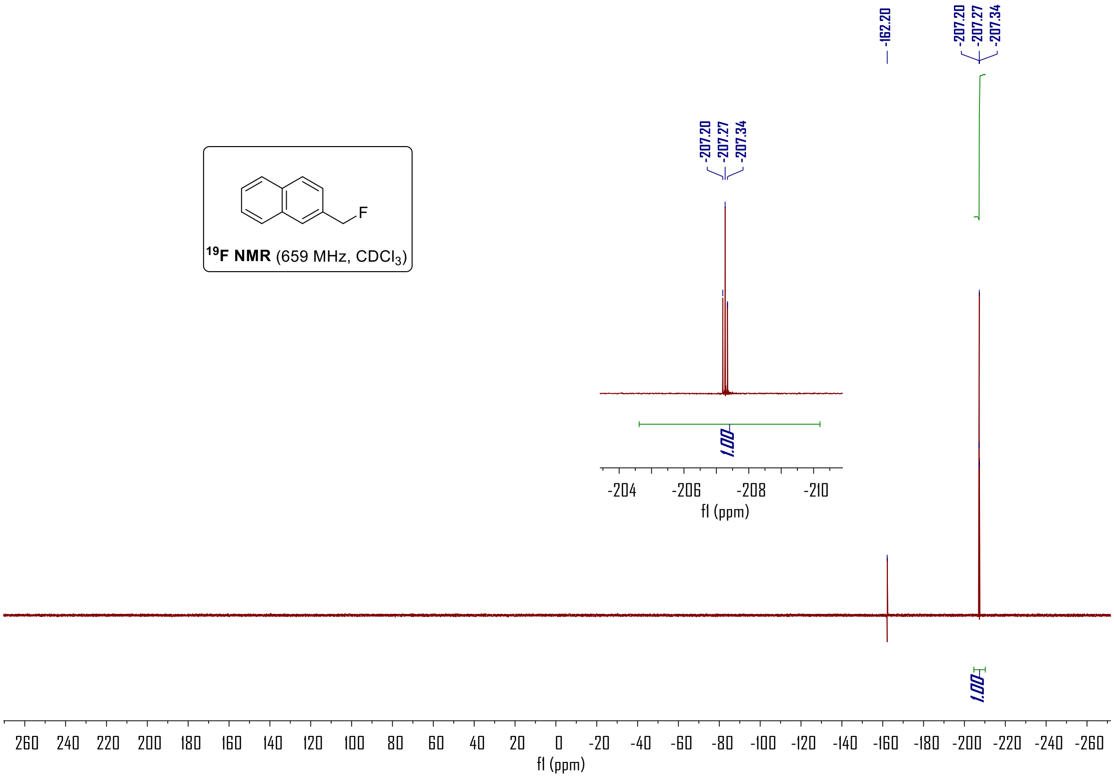

Penta-1,2-dien-3-ylbenzene (3a)

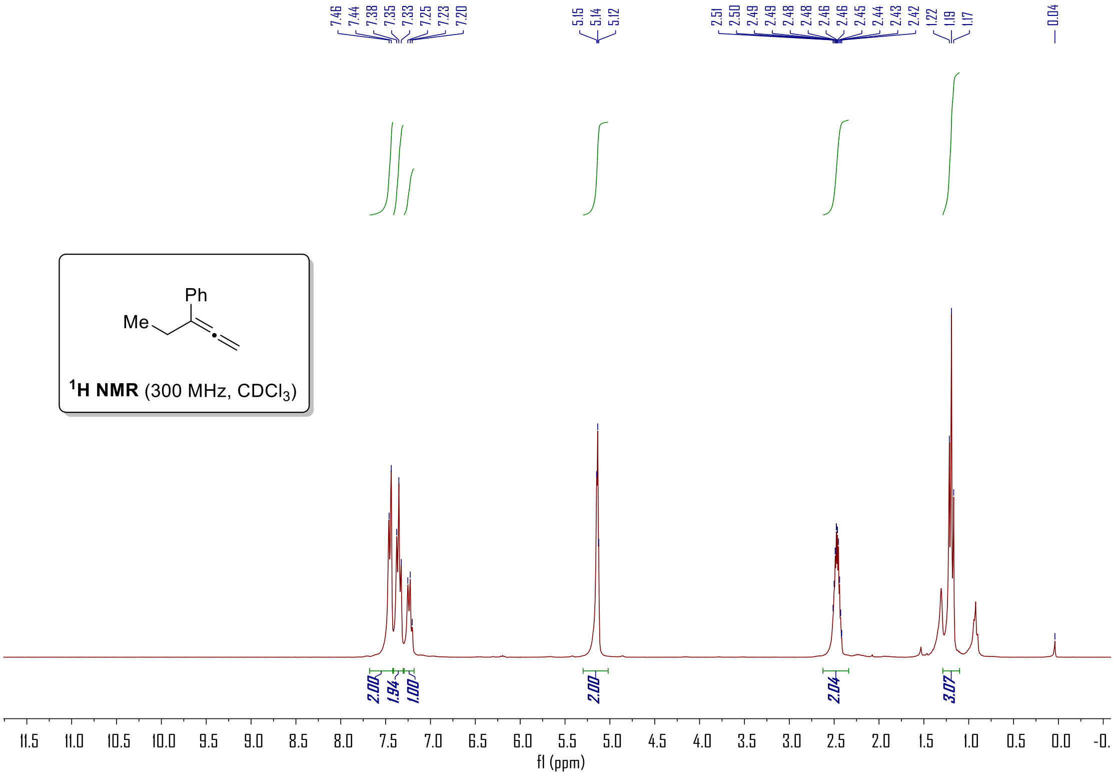

Buta-2,3-dien-2-ylbenzene (3b)

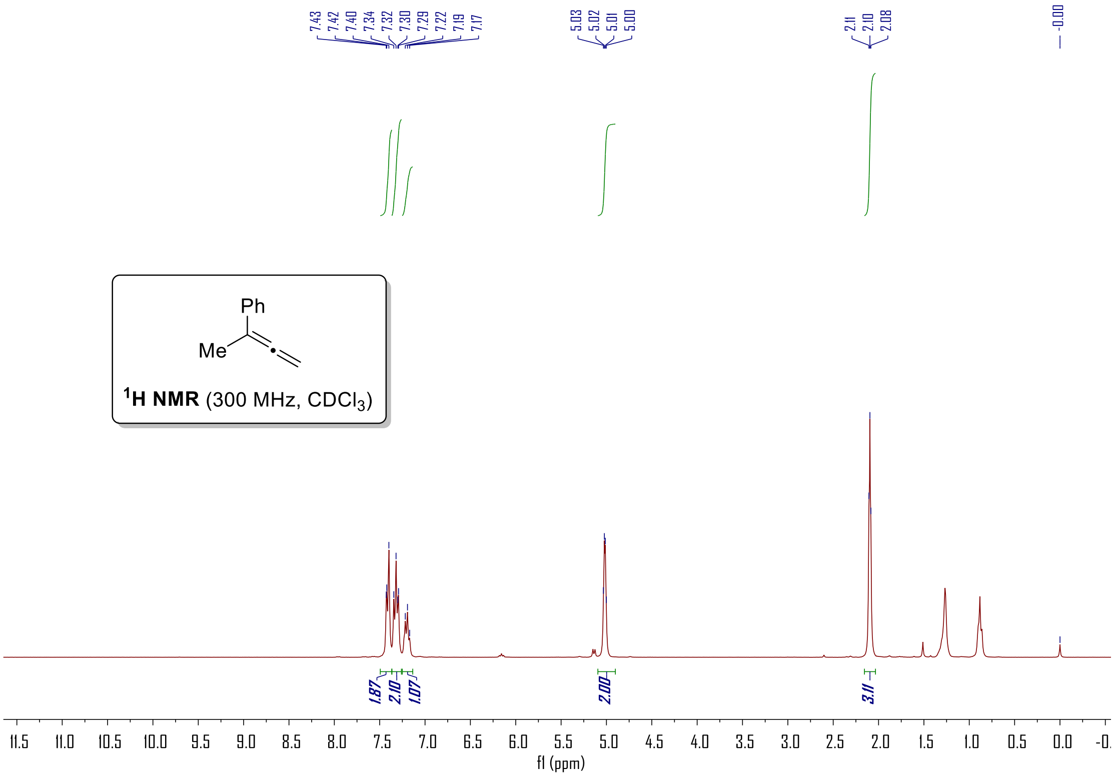

Octa-1,2-dien-3-ylbenzene (3c)

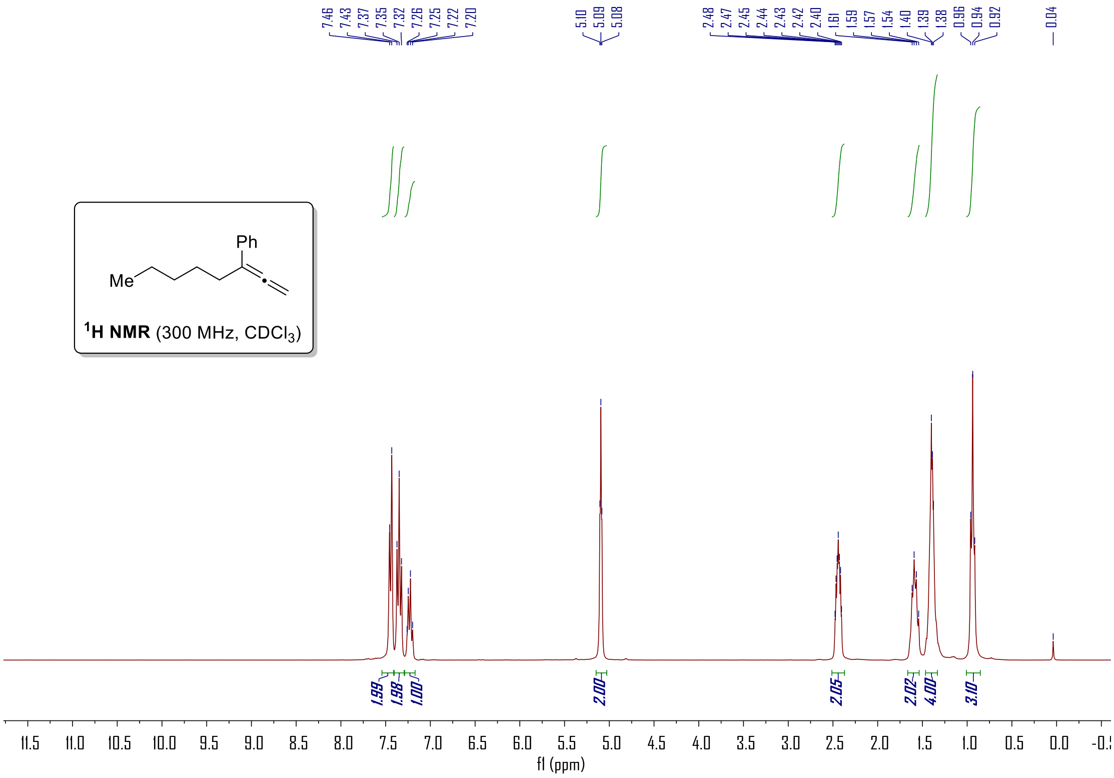

(5-Methylhexa-1,2-dien-3-yl)benzene (3d)

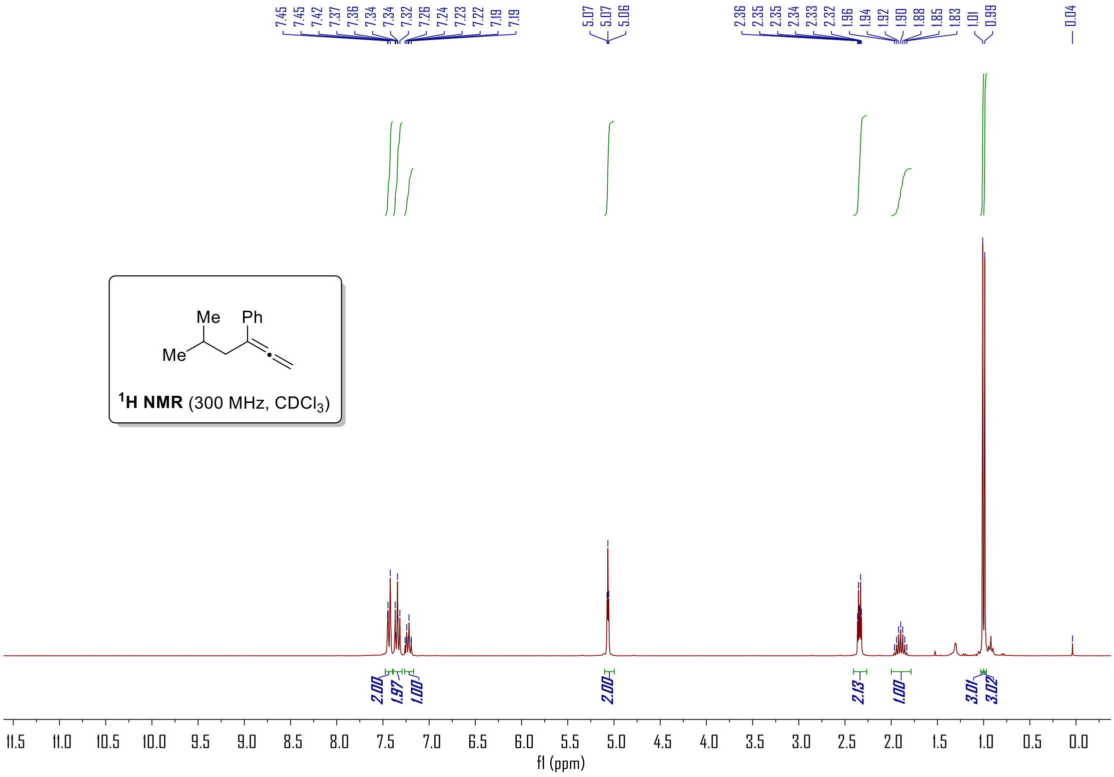

(4-Methylpenta-1,2-dien-3-yl)benzene (3e)

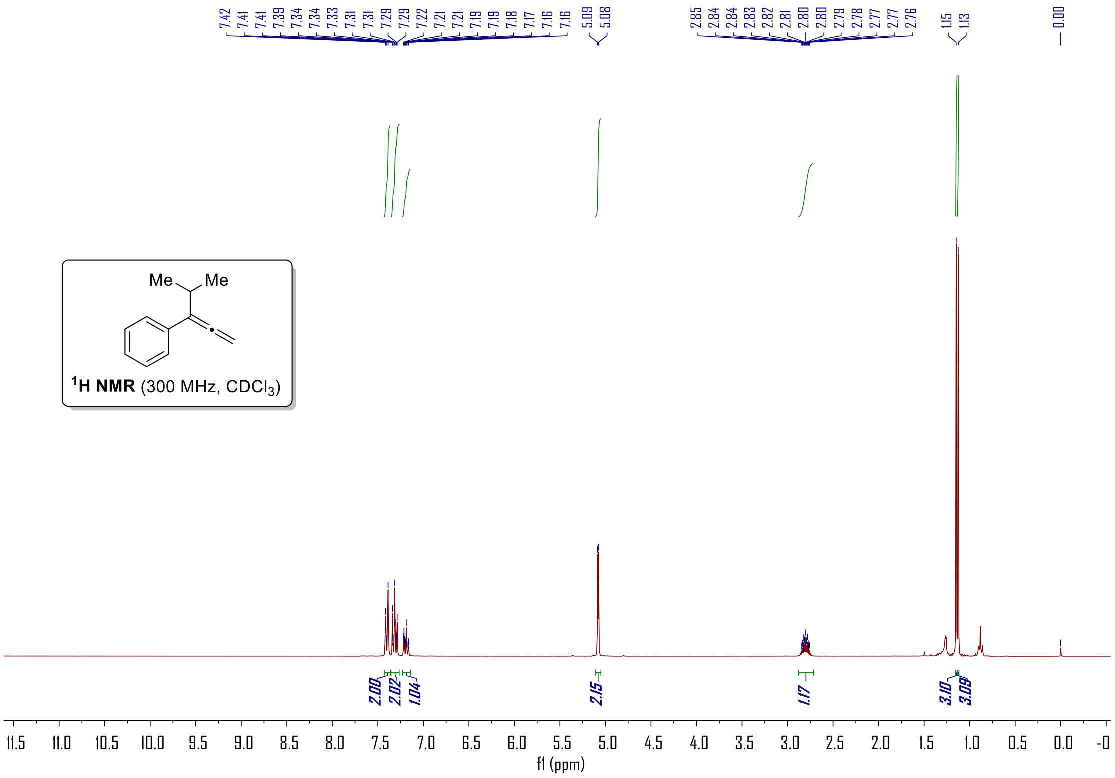

1-(Penta-1,2-dien-3-yl)-2-(trifluoromethoxy)benzene (3f)

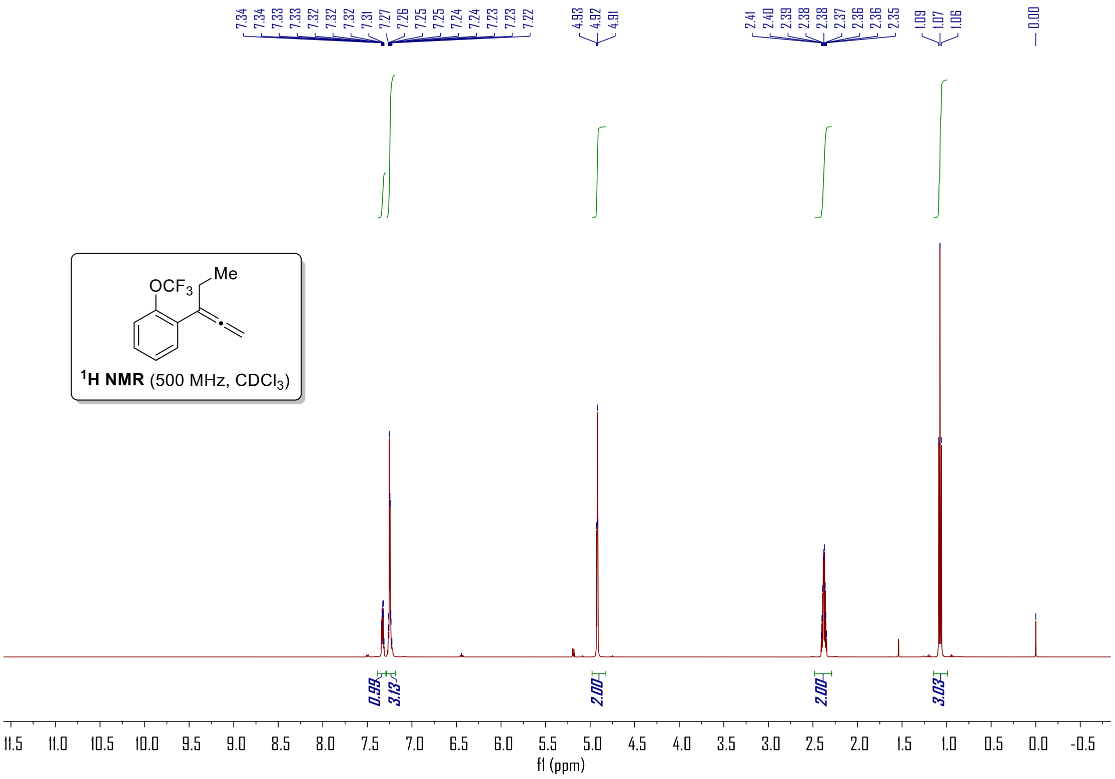

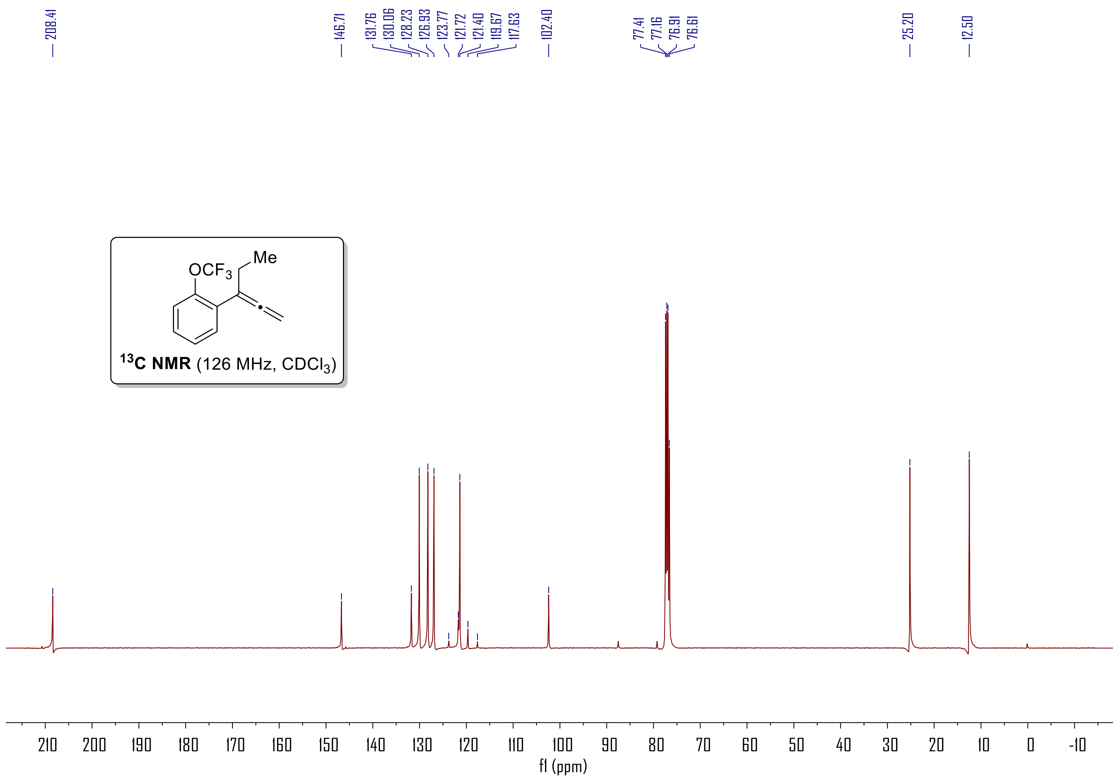

208.41
146.71
131.76
130.06
128.23
126.93
123.77
121.72
121.40
119.67
117.63
102.40
77.41
77.16
76.91
76.61
25.20
12.50
OCF3
Me
13C NMR (126 MHz, CDCl3)
fl (ppm)

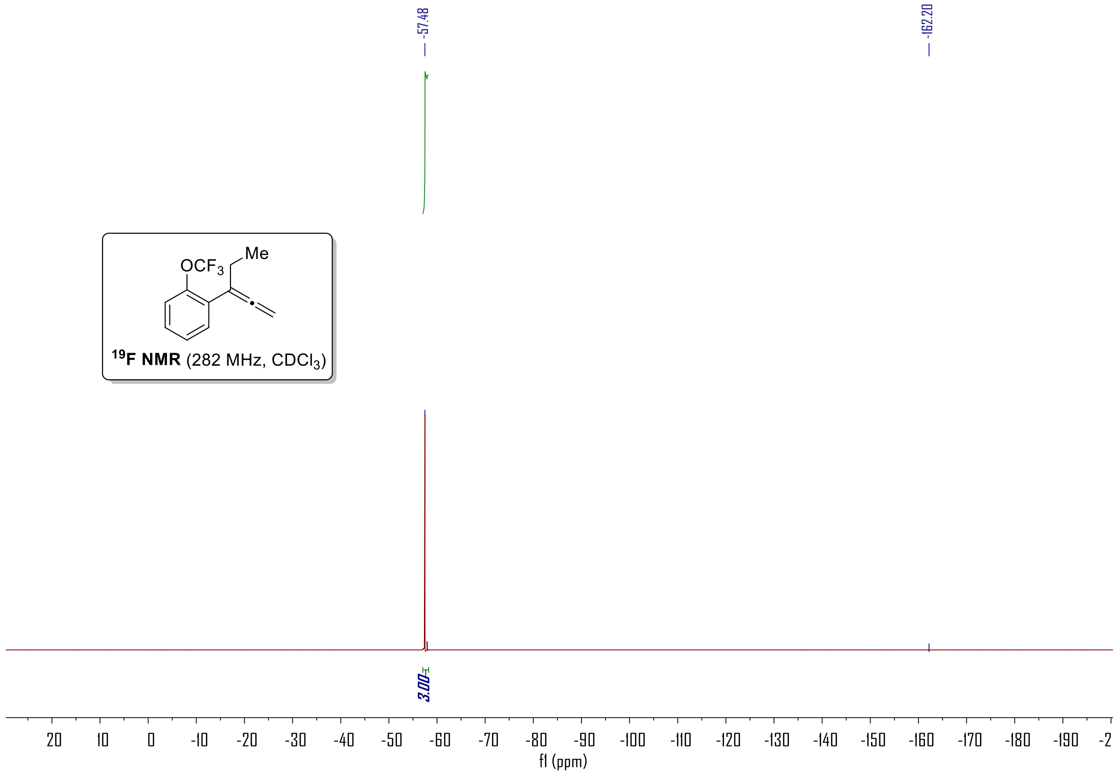

-57.48
-162.20
OCF3
Me
19F NMR (282 MHz, CDCl3)
3.00
fl (ppm)

1-Methoxy-4-(penta-1,2-dien-3-yl)benzene (3g)

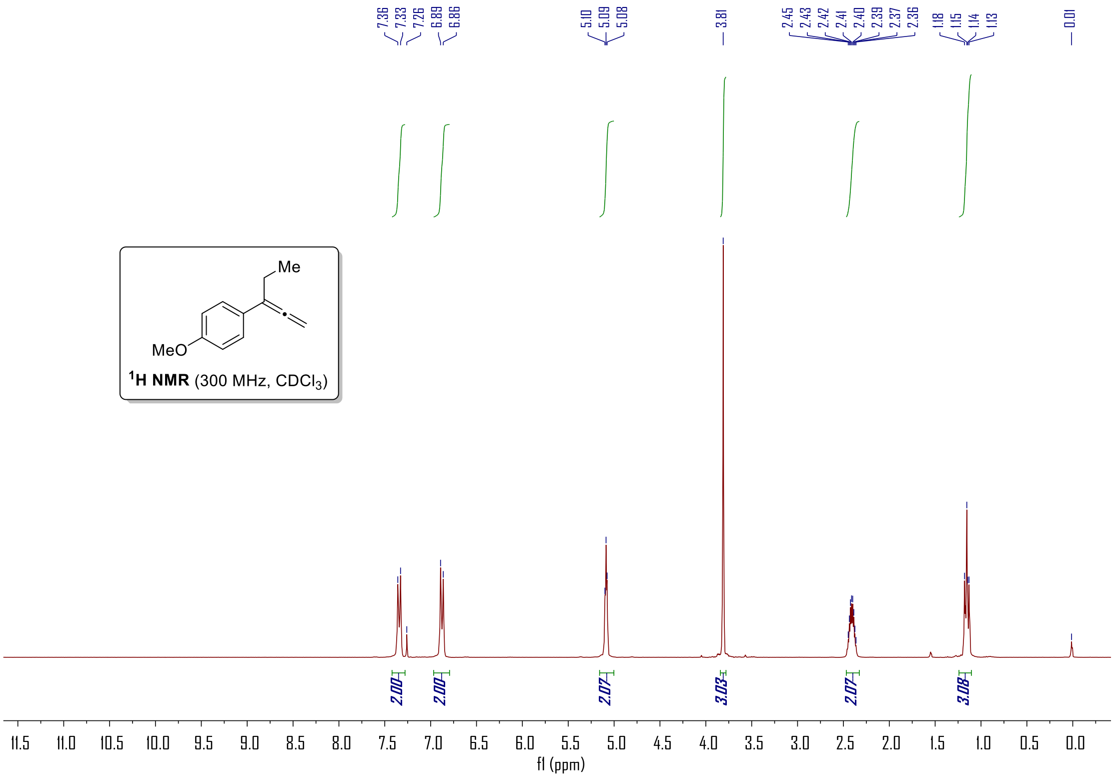

1-Methoxy-3-(penta-1,2-dien-3-yl)benzene (3h)

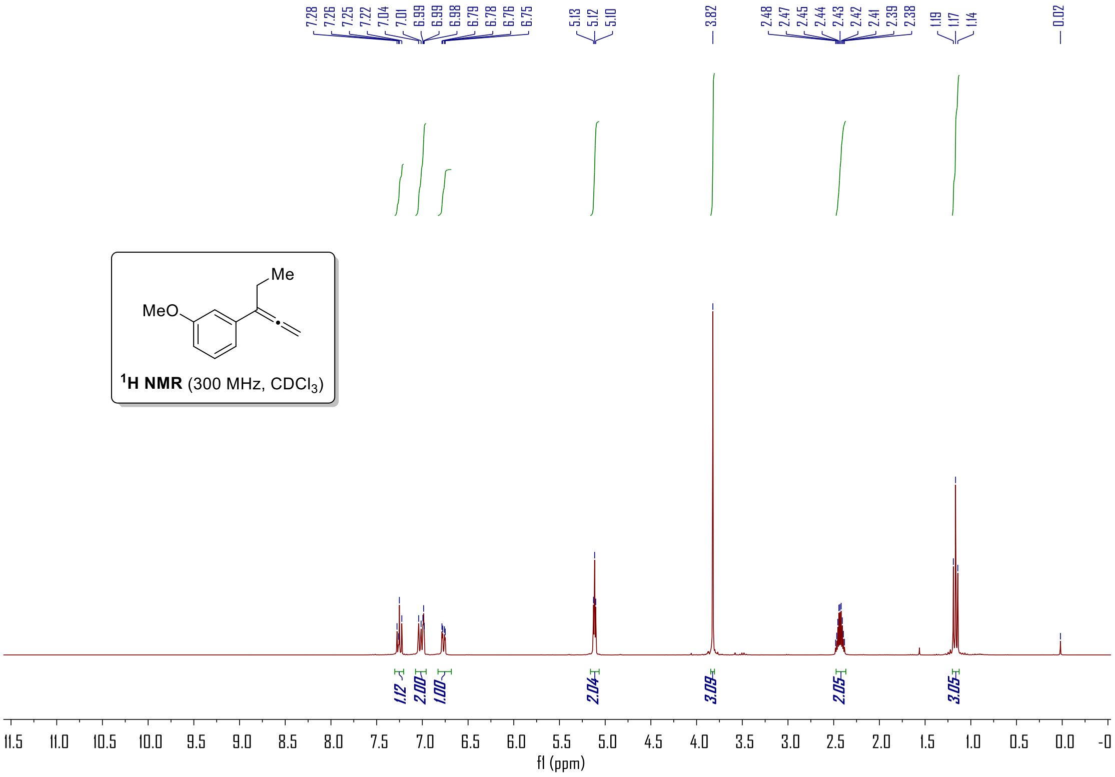

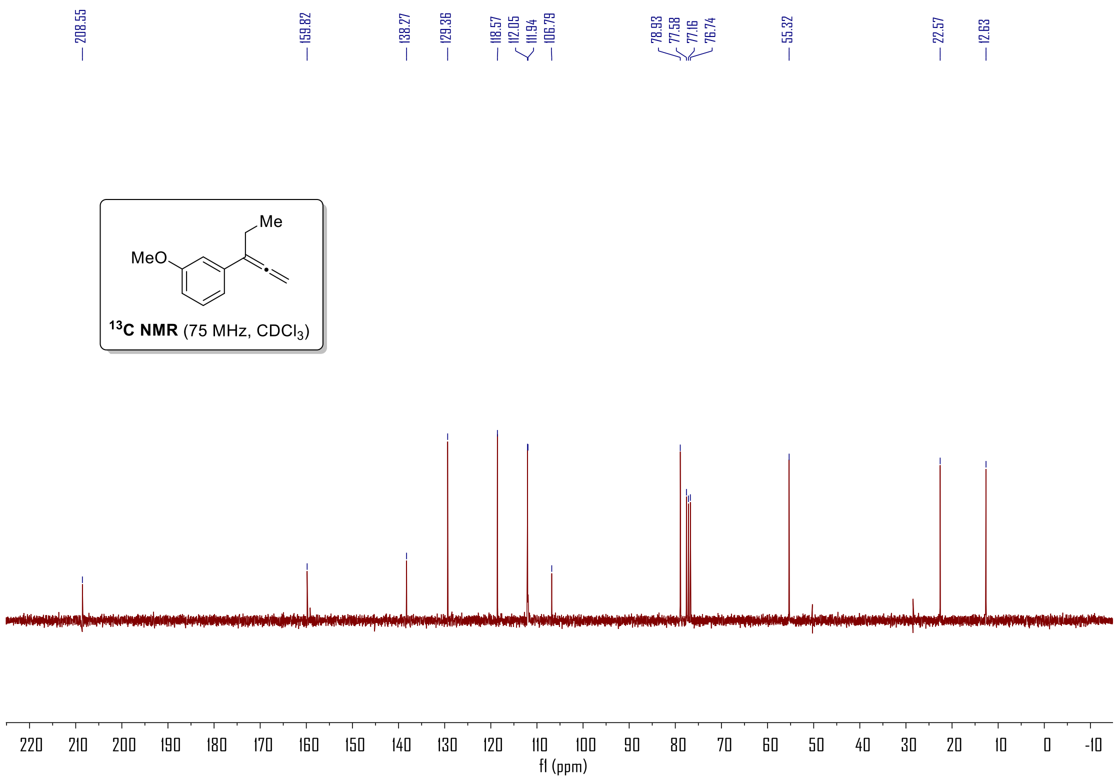

1-Methyl-4-(penta-1,2-dien-3-yl)benzene (3i)

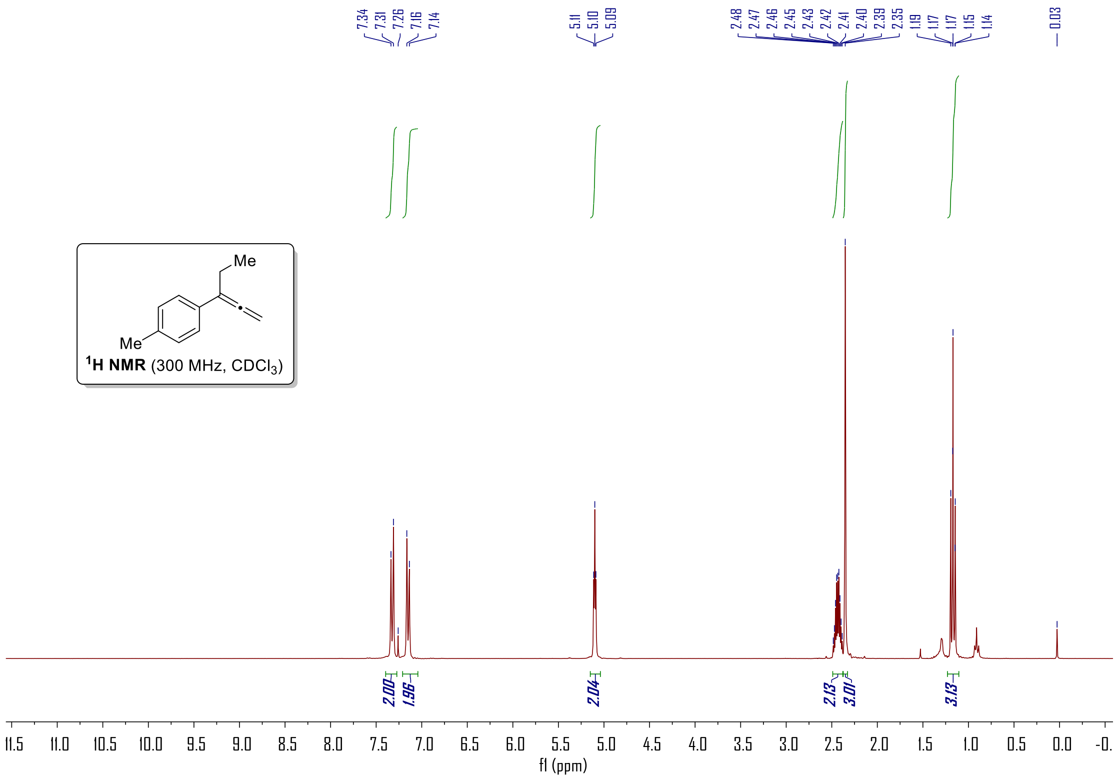

1-Methyl-3-(penta-1,2-dien-3-yl)benzene (3j)

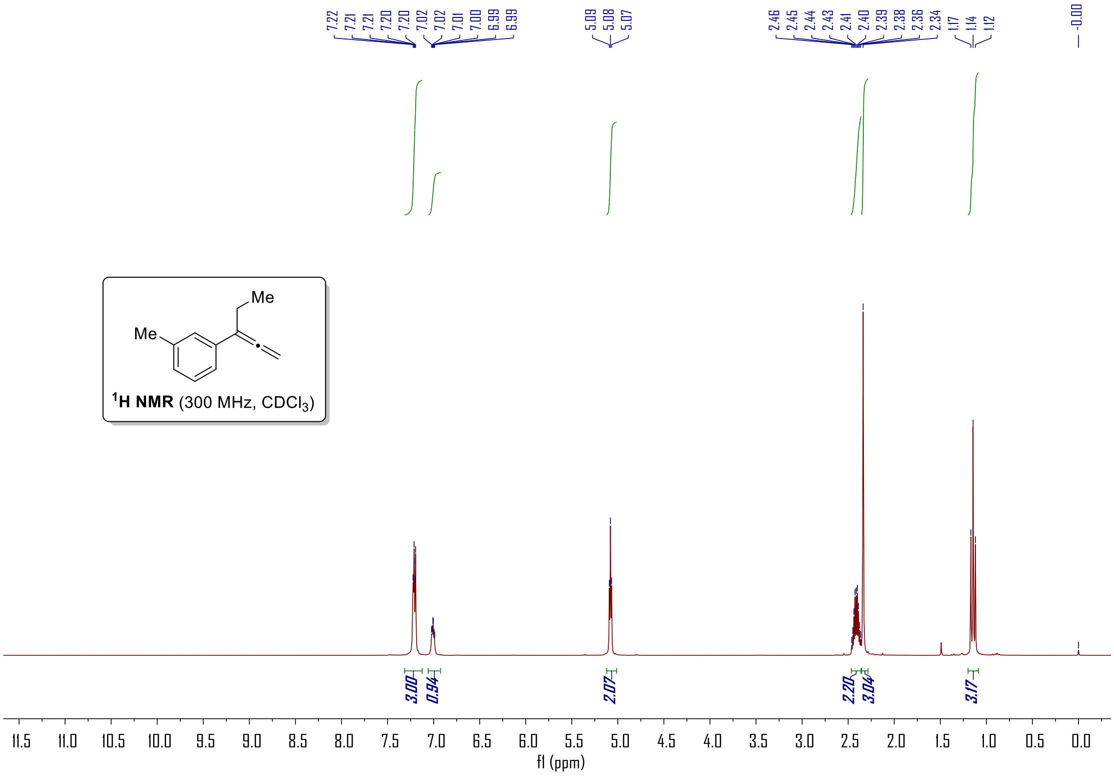

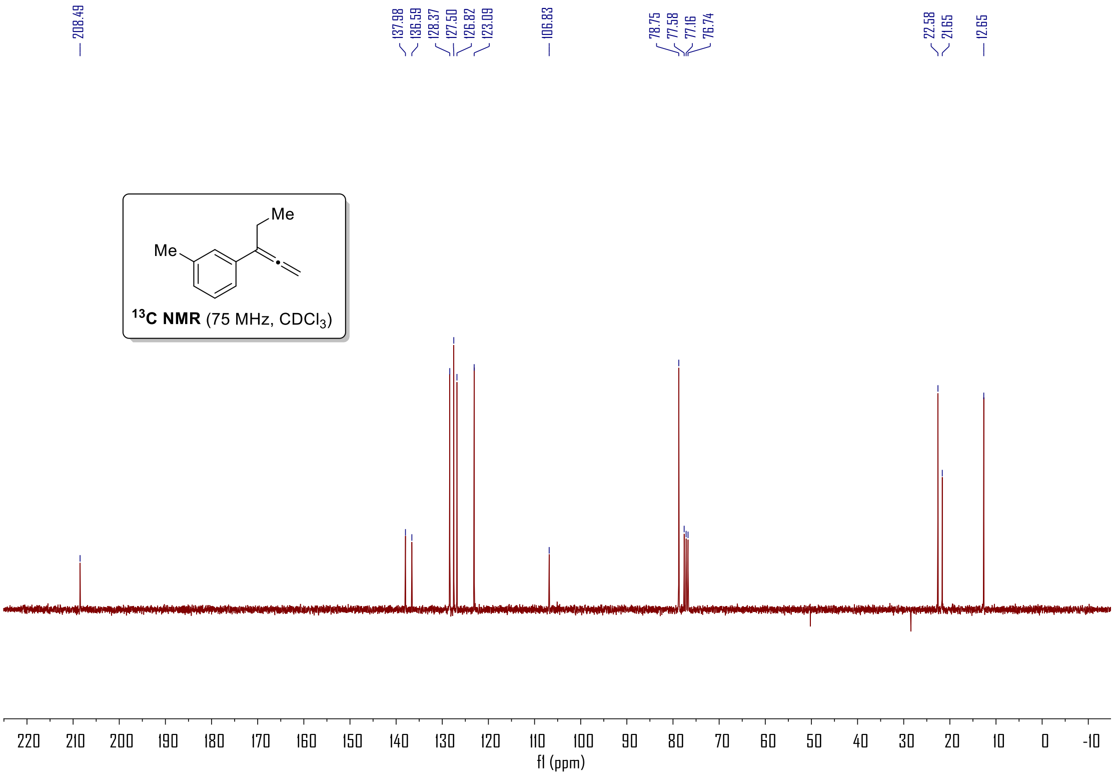

1,3-Dimethyl-5-(penta-1,2-dien-3-yl)benzene (3k)

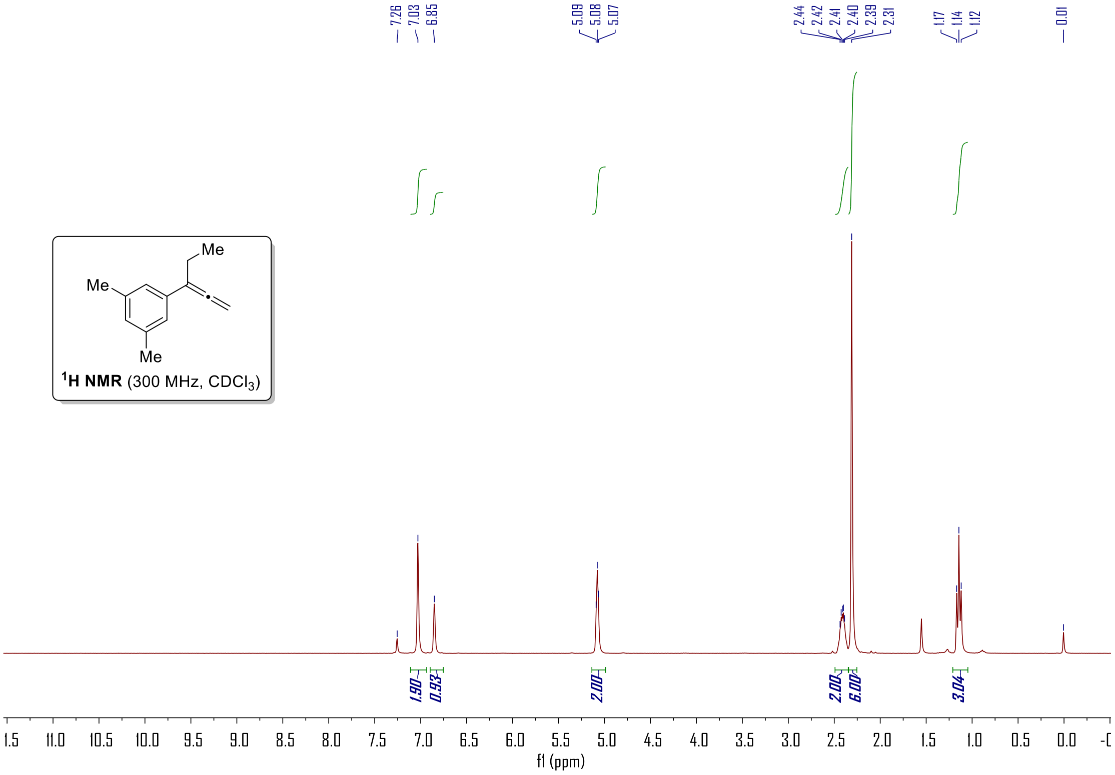

1-Chloro-4-(penta-1,2-dien-3-yl)benzene (3l)

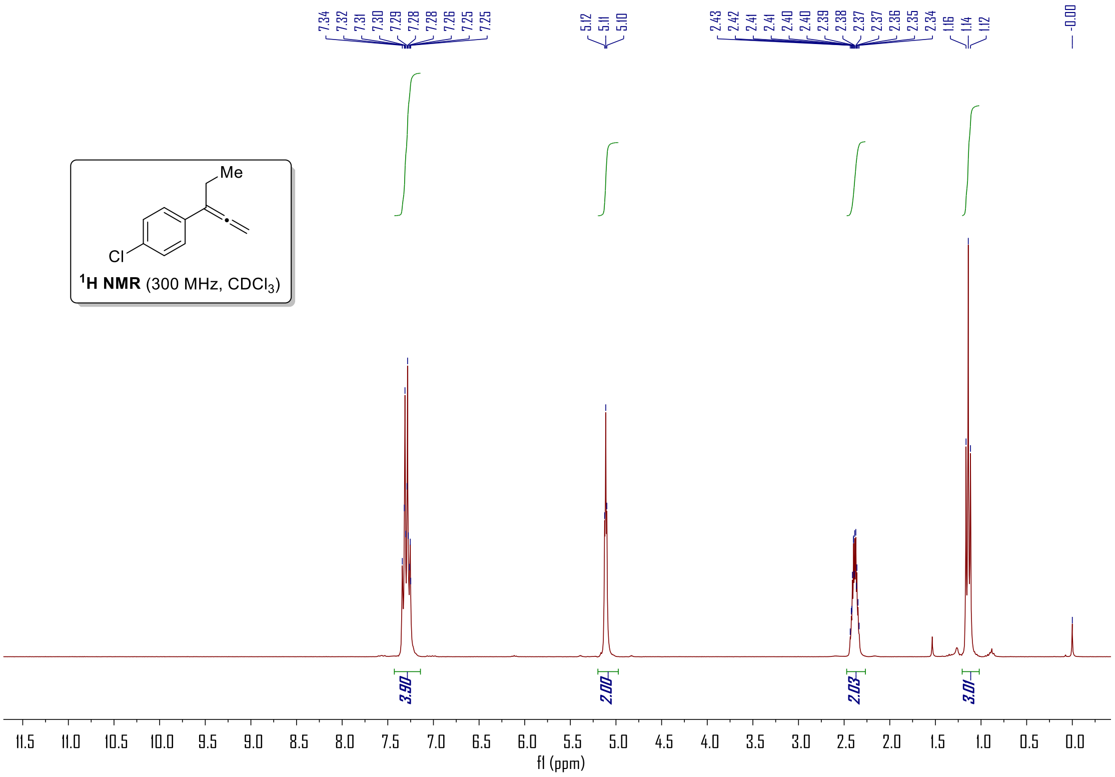

2-(Penta-1,2-dien-3-yl)naphthalene (3m)

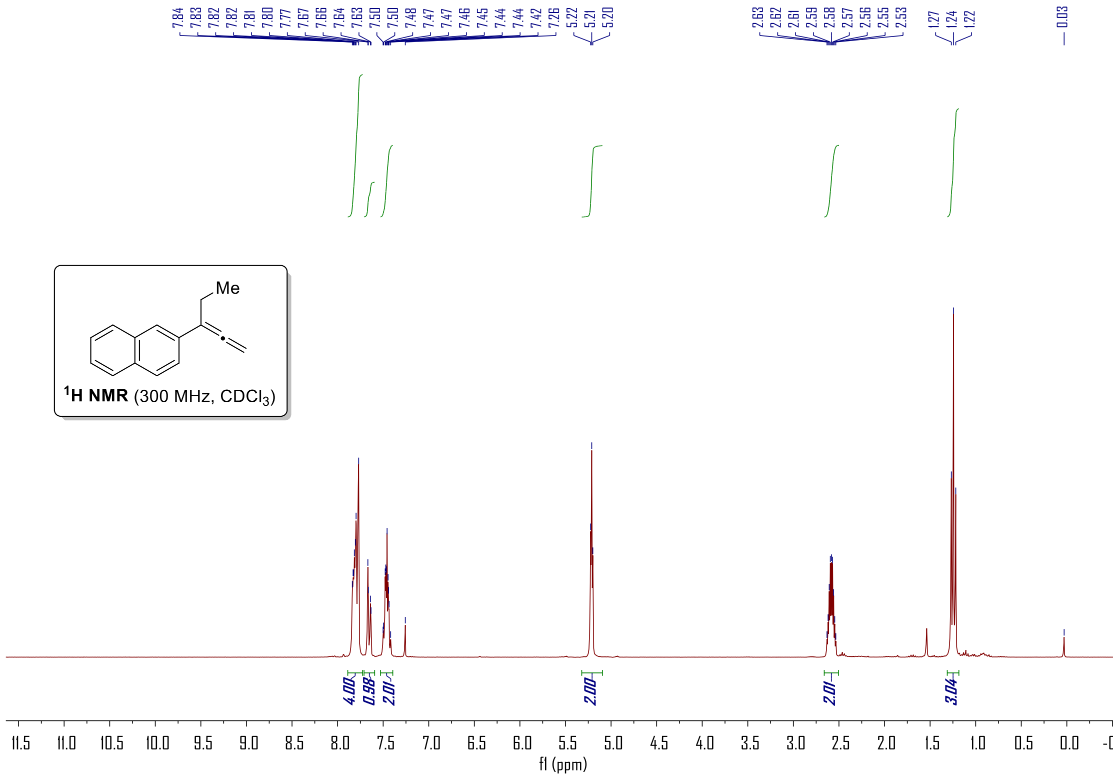

(1-Cyclopropylpropa-1,2-dien-1-yl)benzene (3o)

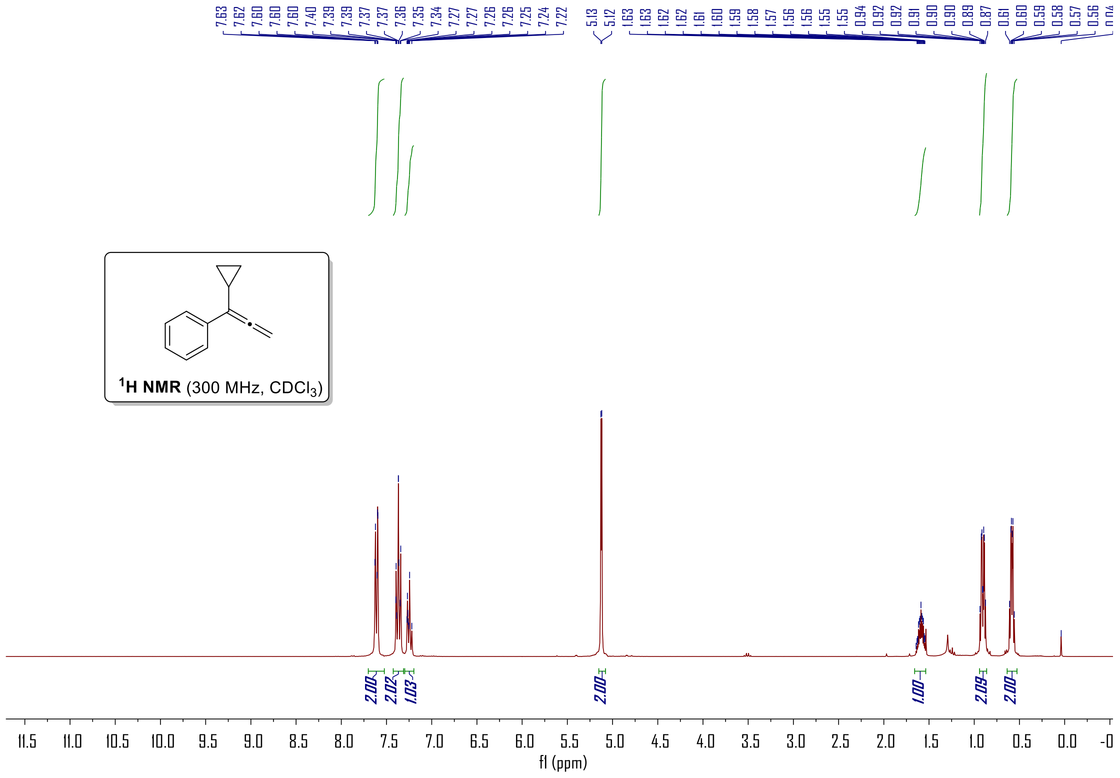

Propa-1,2-diene-1,1-diyldibenzene (3t)

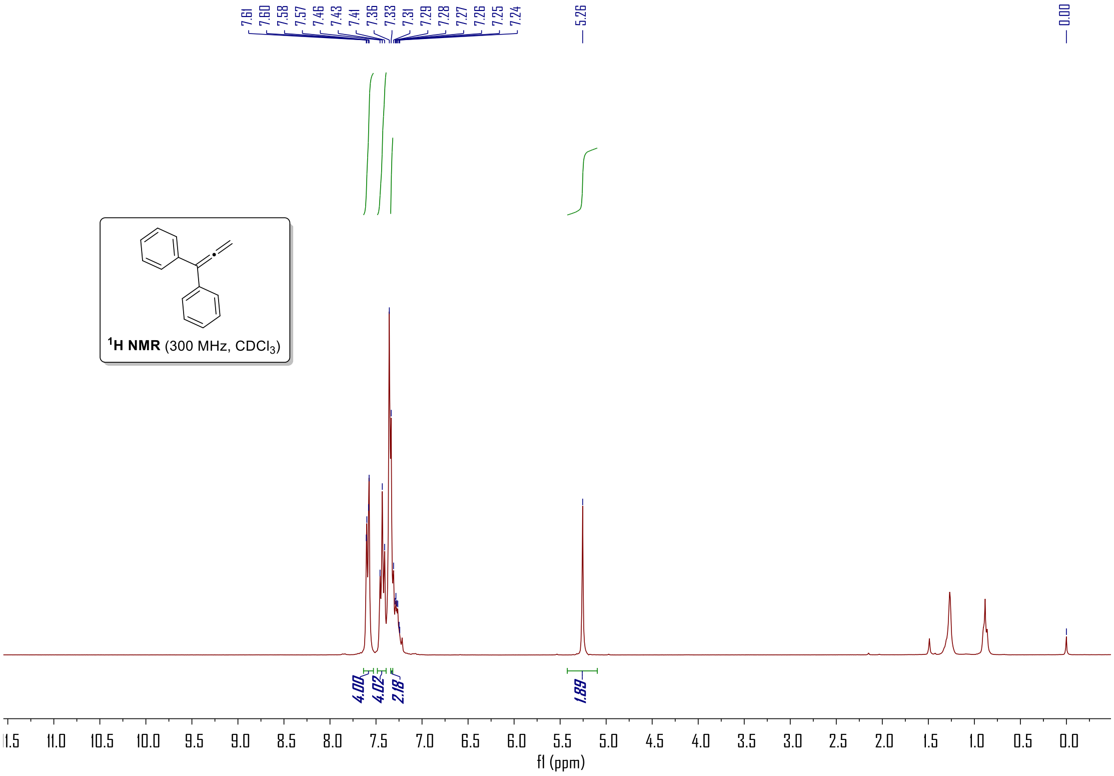

Penta-1,2,4-trien-3-ylbenzene (3u)

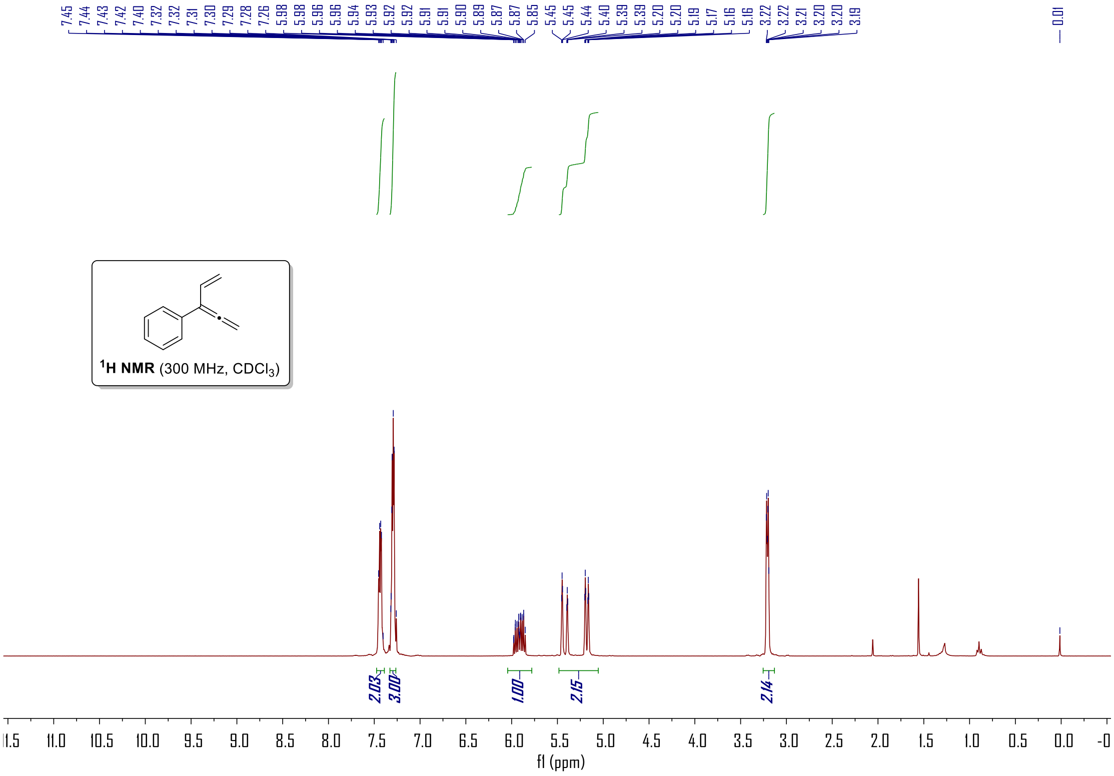

Triethyl(3-phenylpenta-1,2-dien-1-yl)silane (3n)

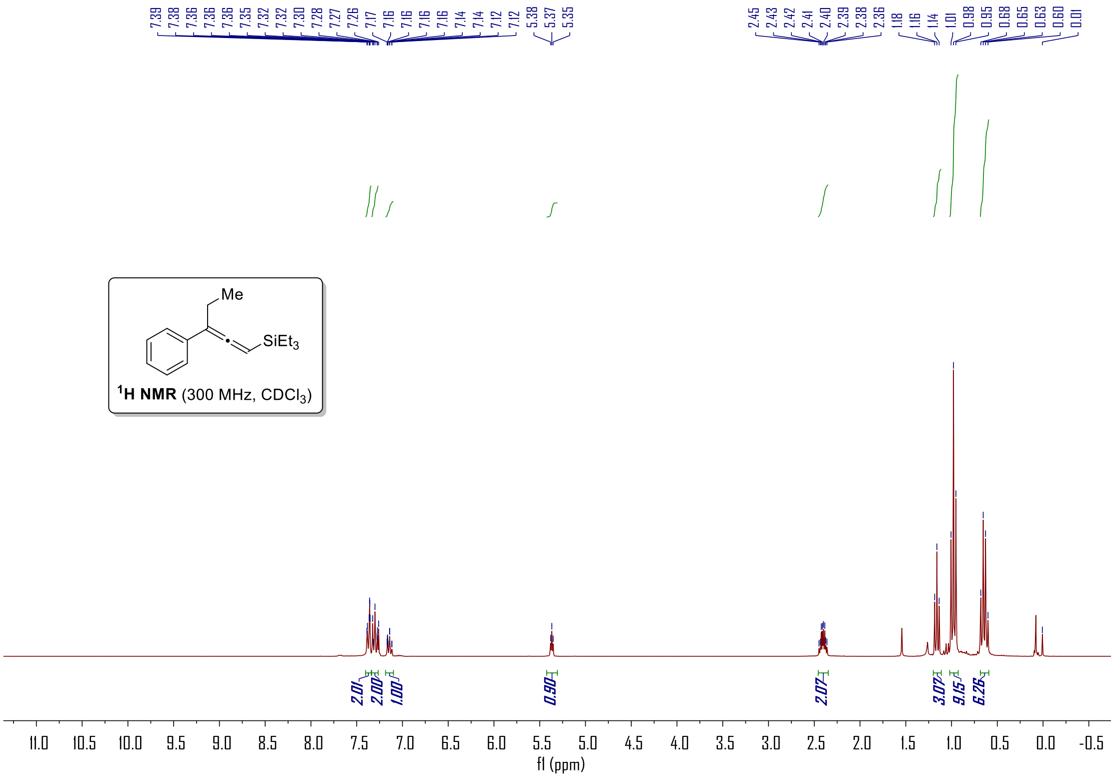

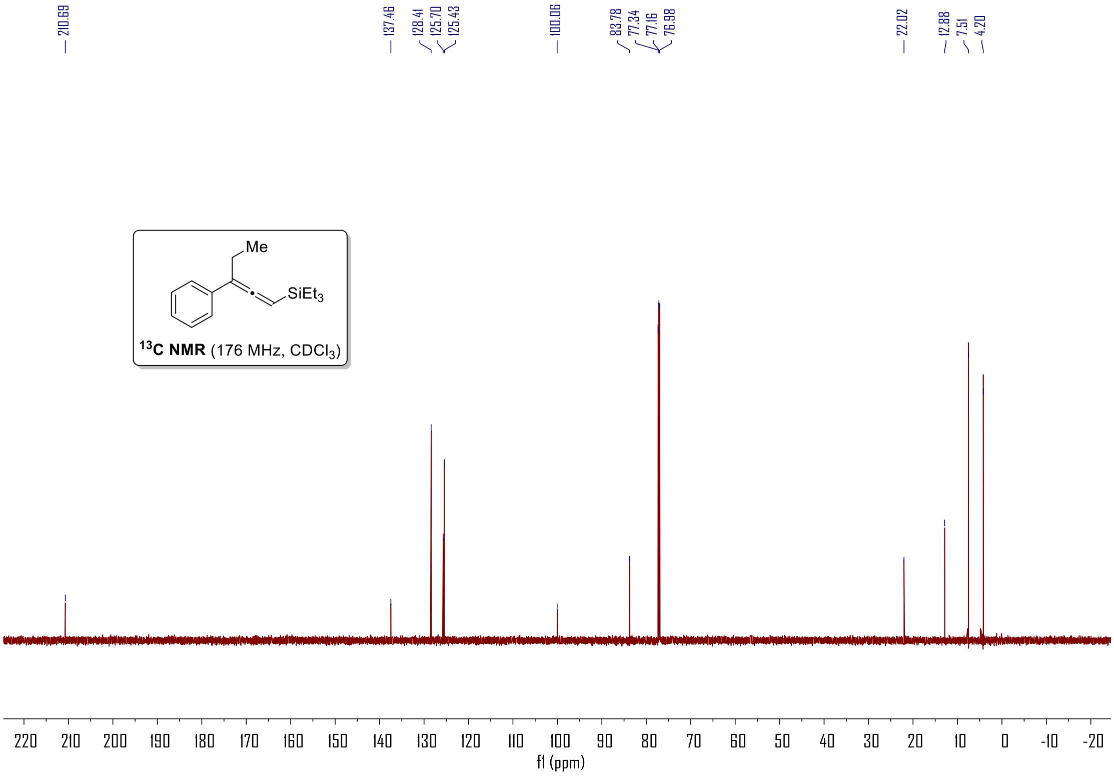

Dodeca-3,4-dien-3-ylbenzene (3p)

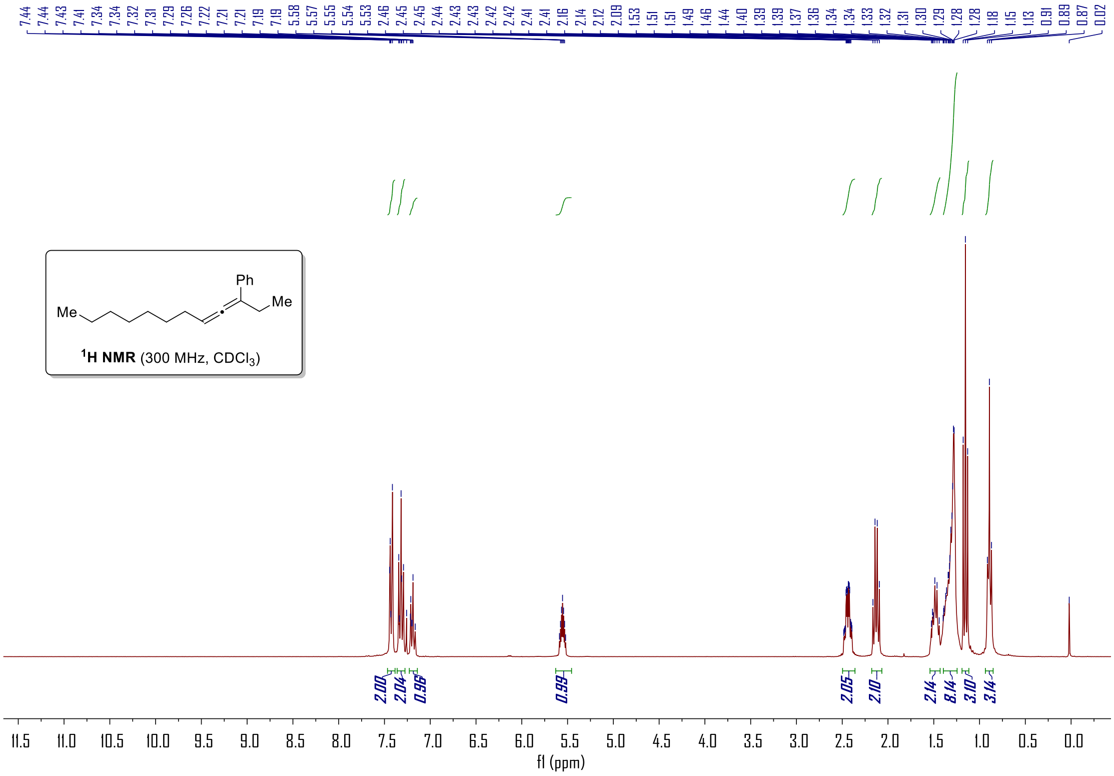

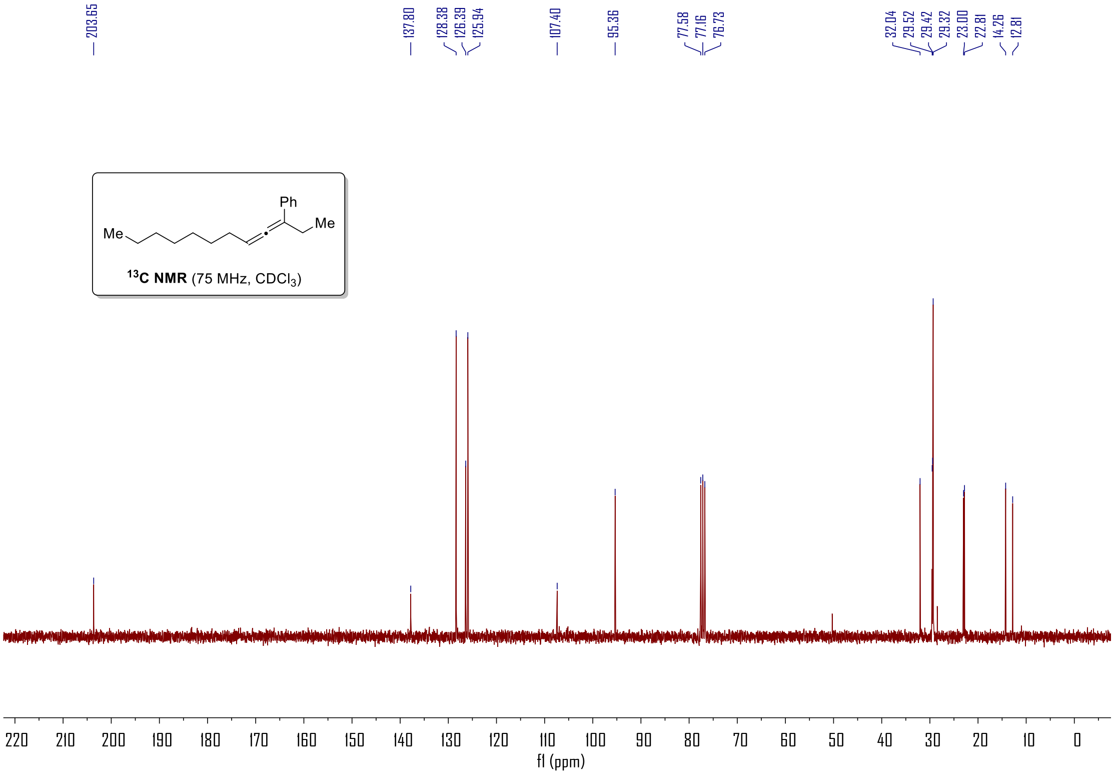

Penta-1,2-diene-1,3-diyldibenzene (3r)

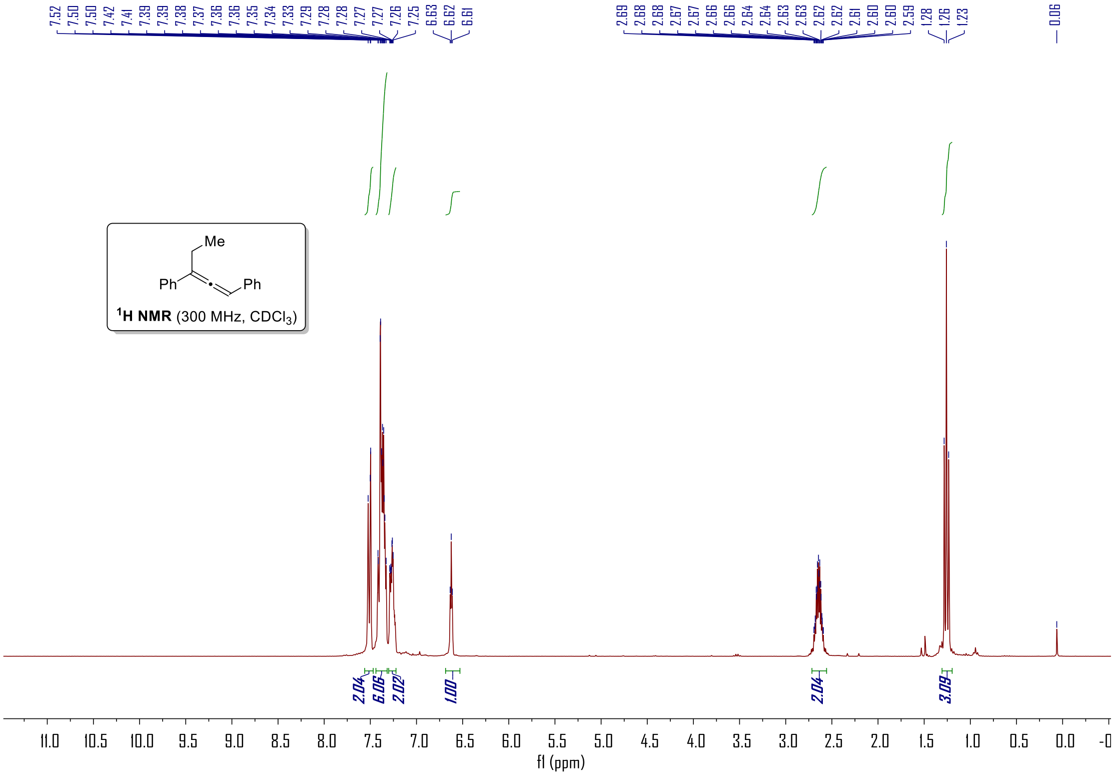

3-Ethylhepta-1,2-diene (3w)

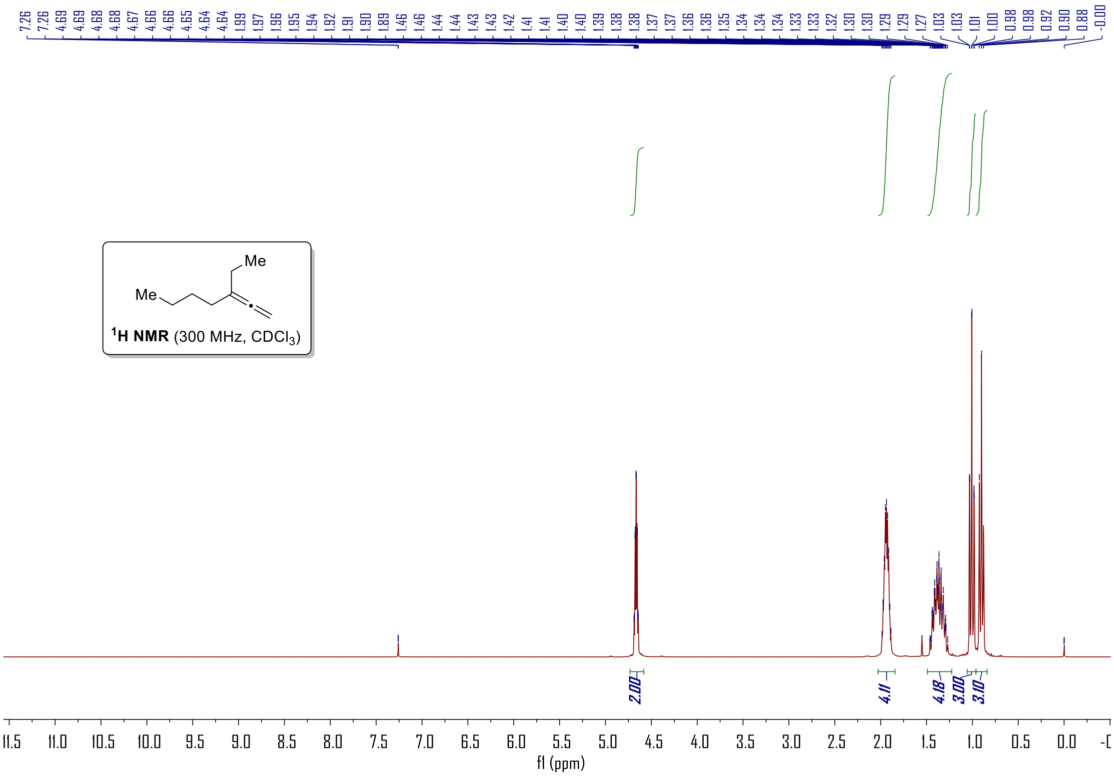

1-Methoxy-4-(penta-1,2-dien-3-yl-1,1-*d*2)benzene (d^2-3g)

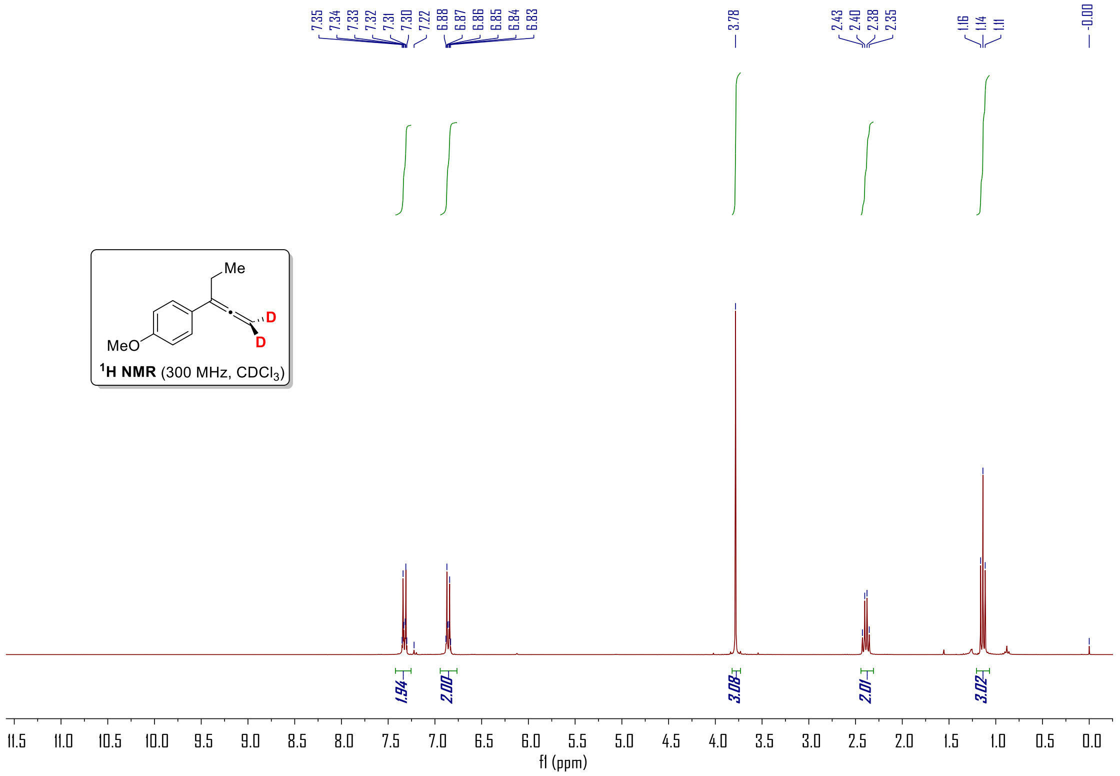

1-Methoxy-4-(penta-1,2-dien-3-yl-1-d)benzene (*d*-3g)

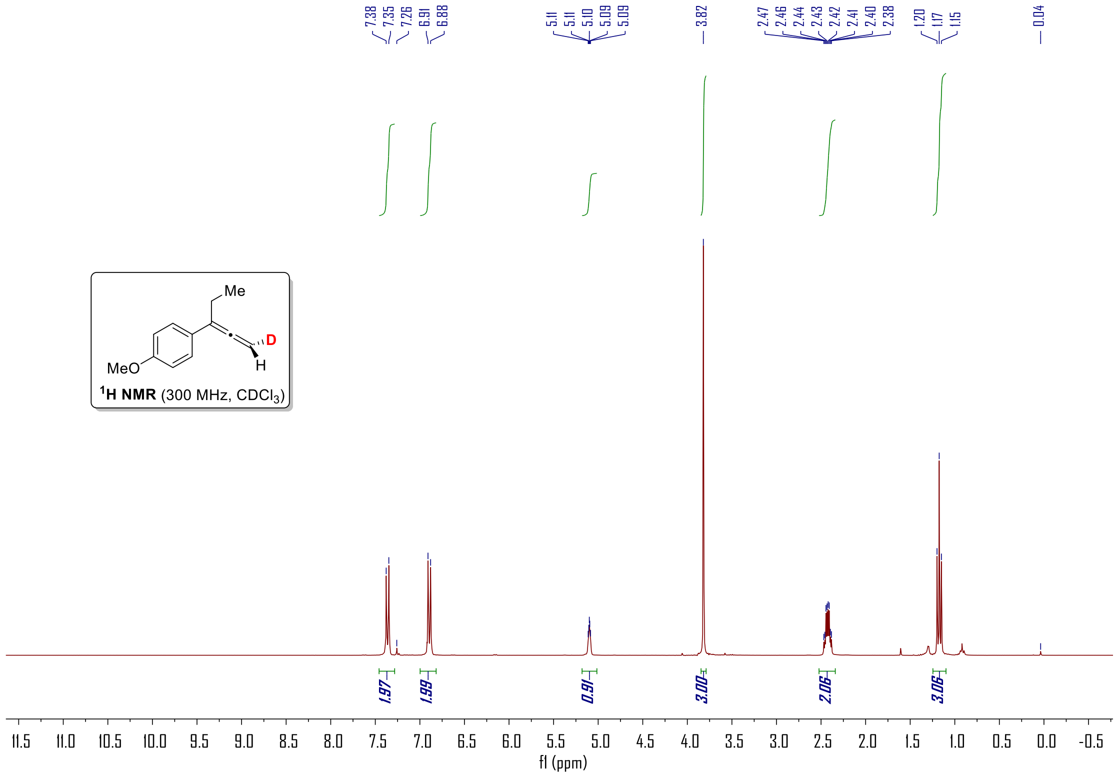

(Penta-1,2-dien-3-yl-1-d)benzene (d^2-3a)

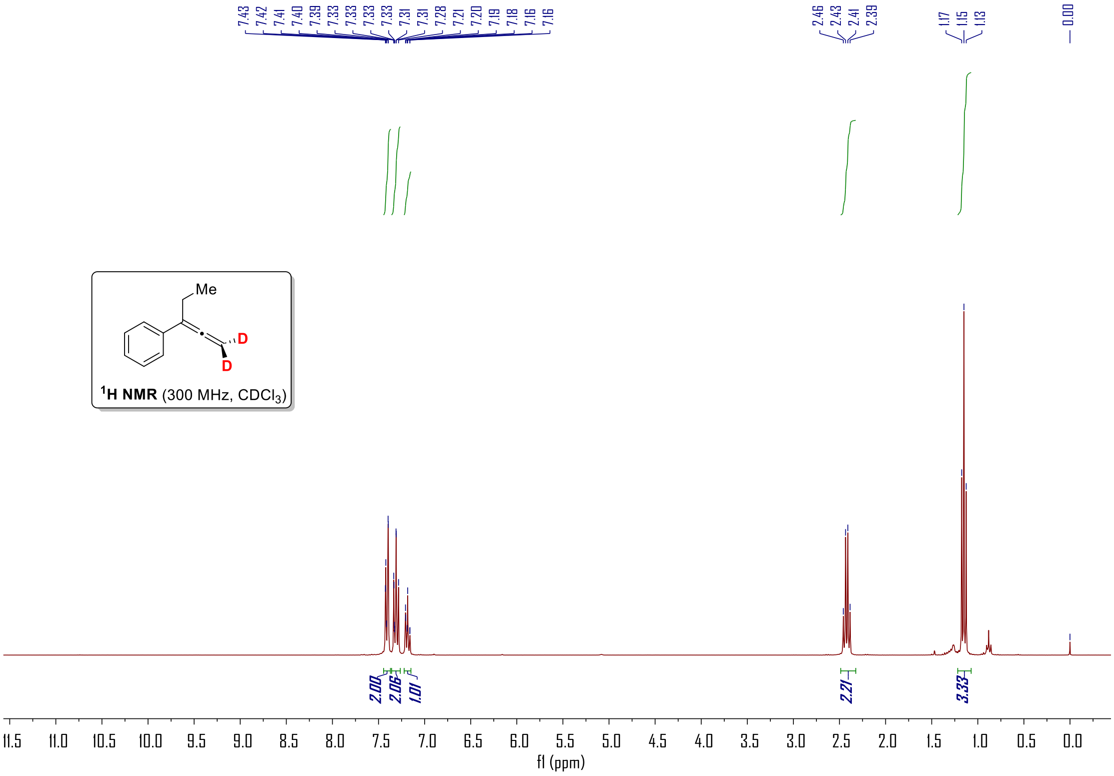

Octa-3,4-dien-3-ylbenzene (3q)

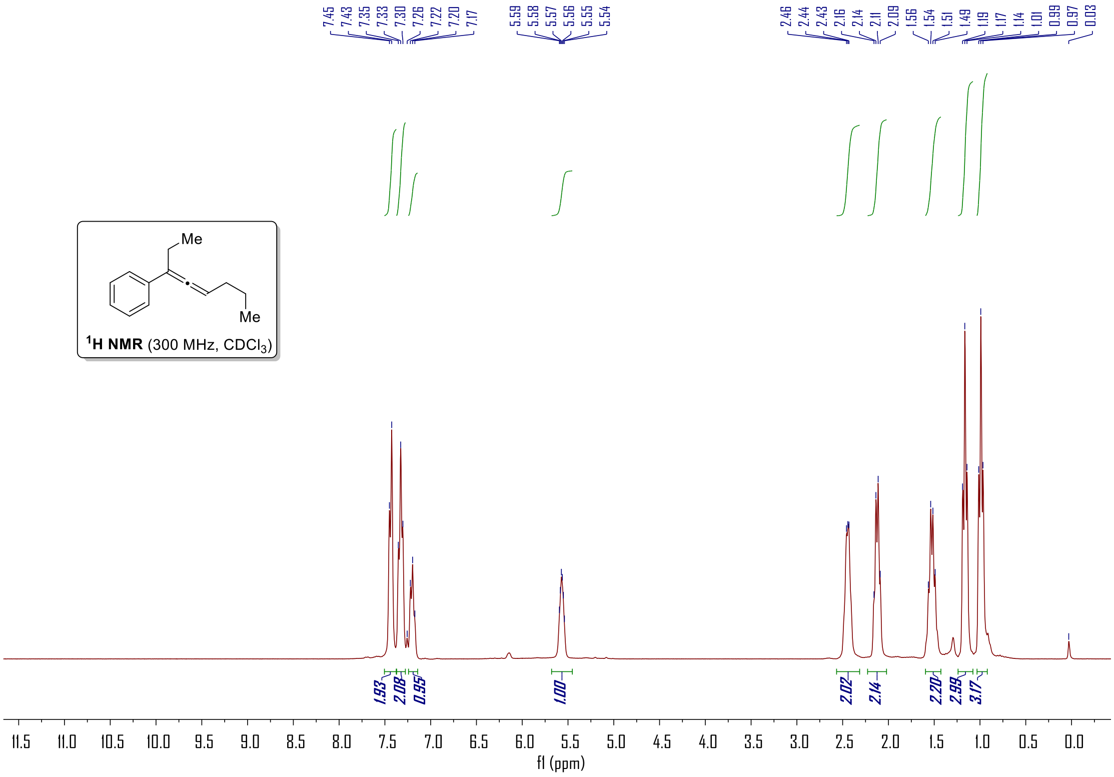

(5-Methylhexa-3,4-dien-3-yl)benzene (3s)

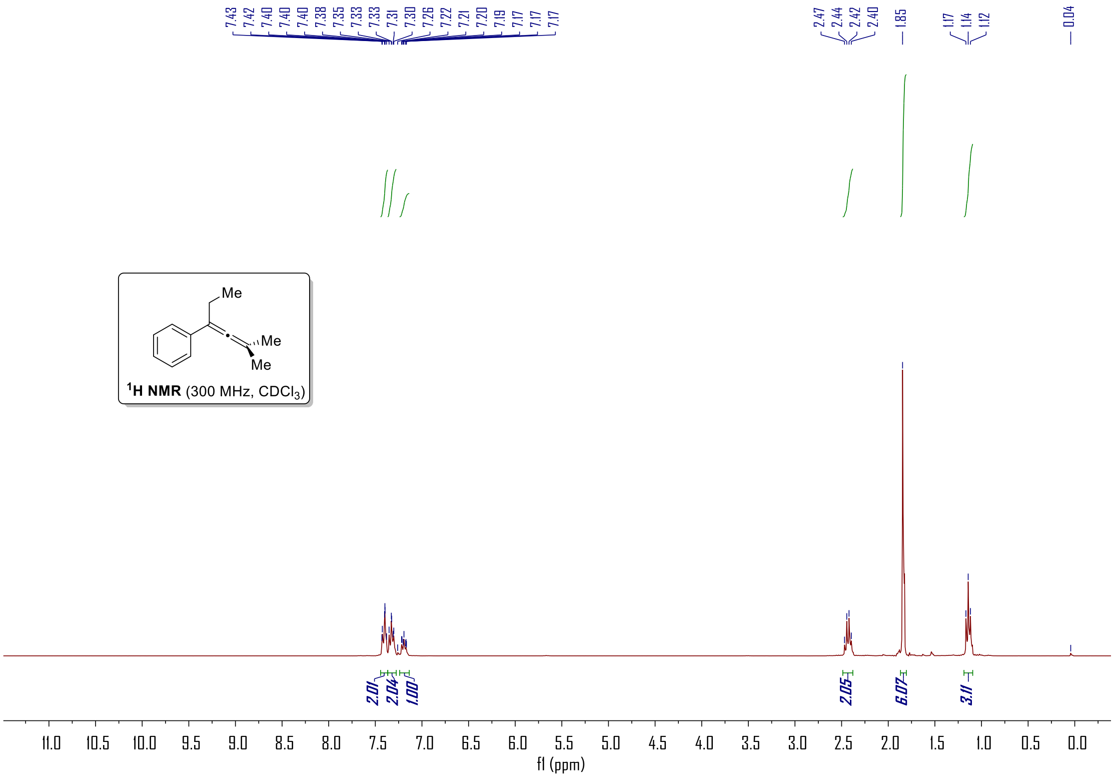

Pent-1-yn-3-ylbenzene (12)

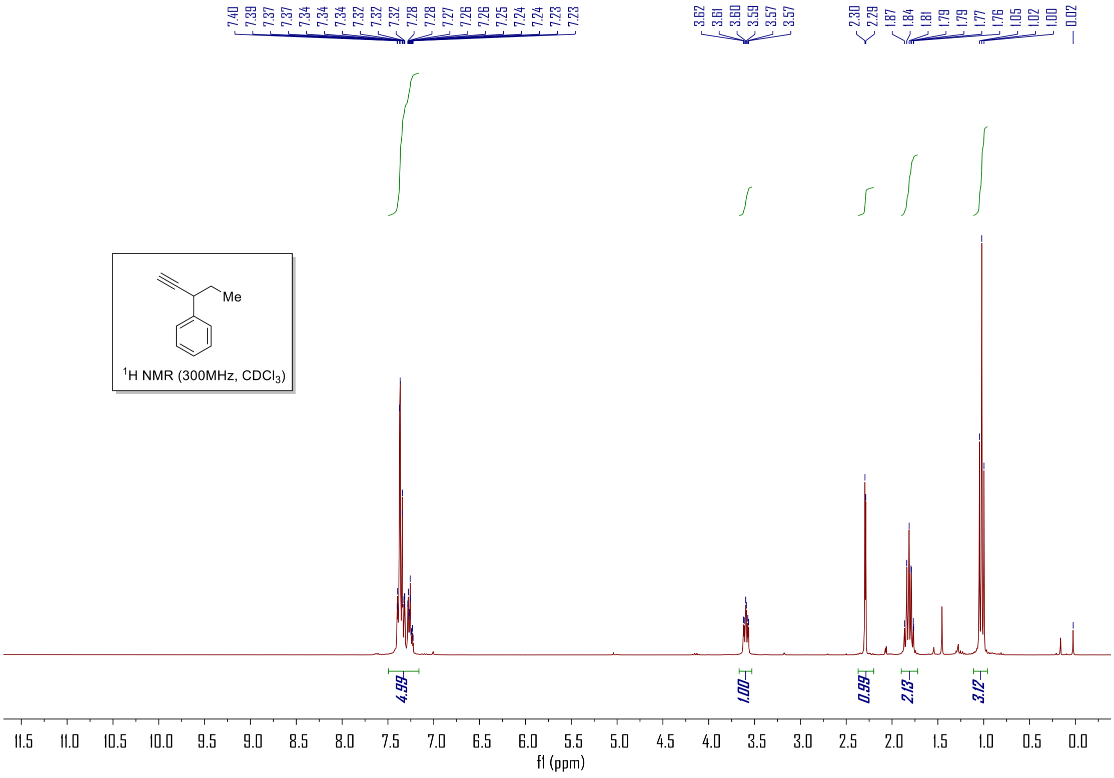

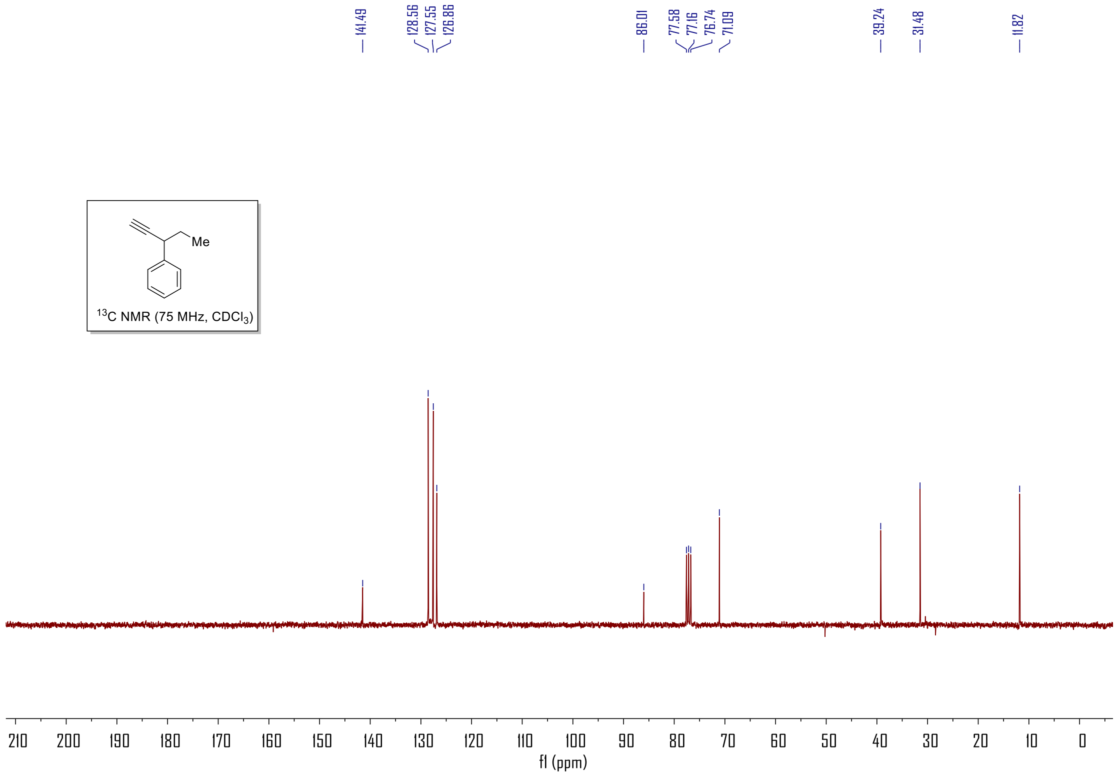

4-(3-Phenylpent-1-yn-3-yl)biphenyl (4aa)

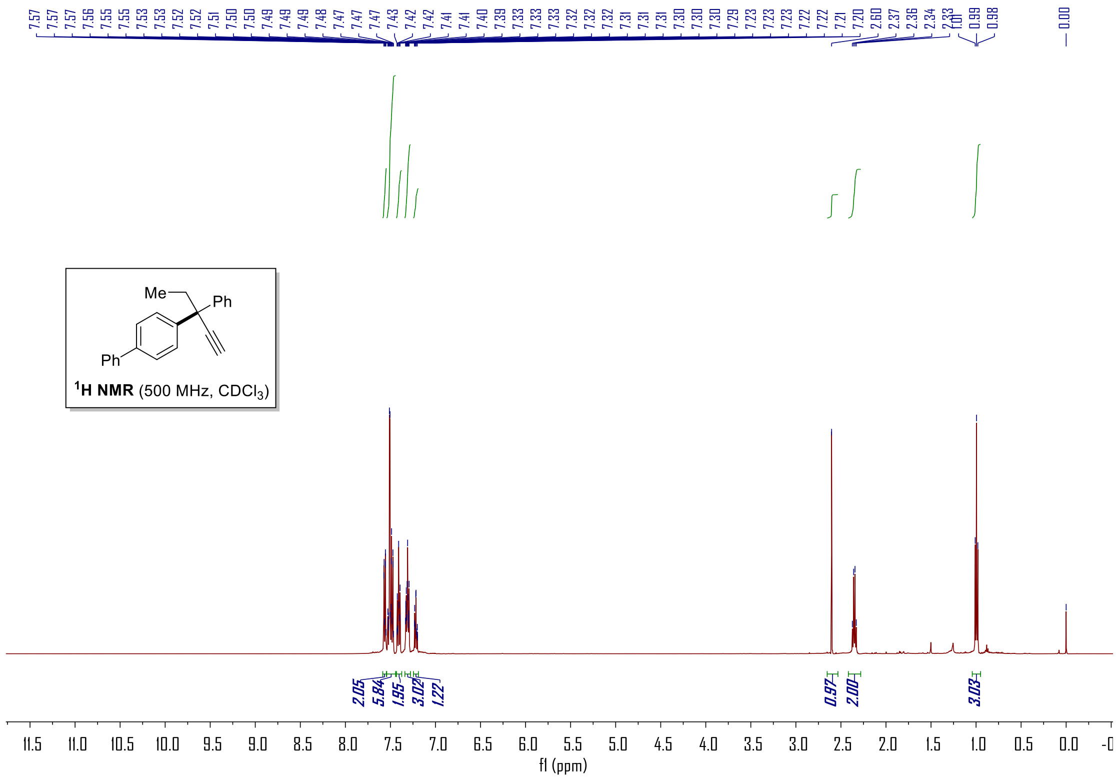

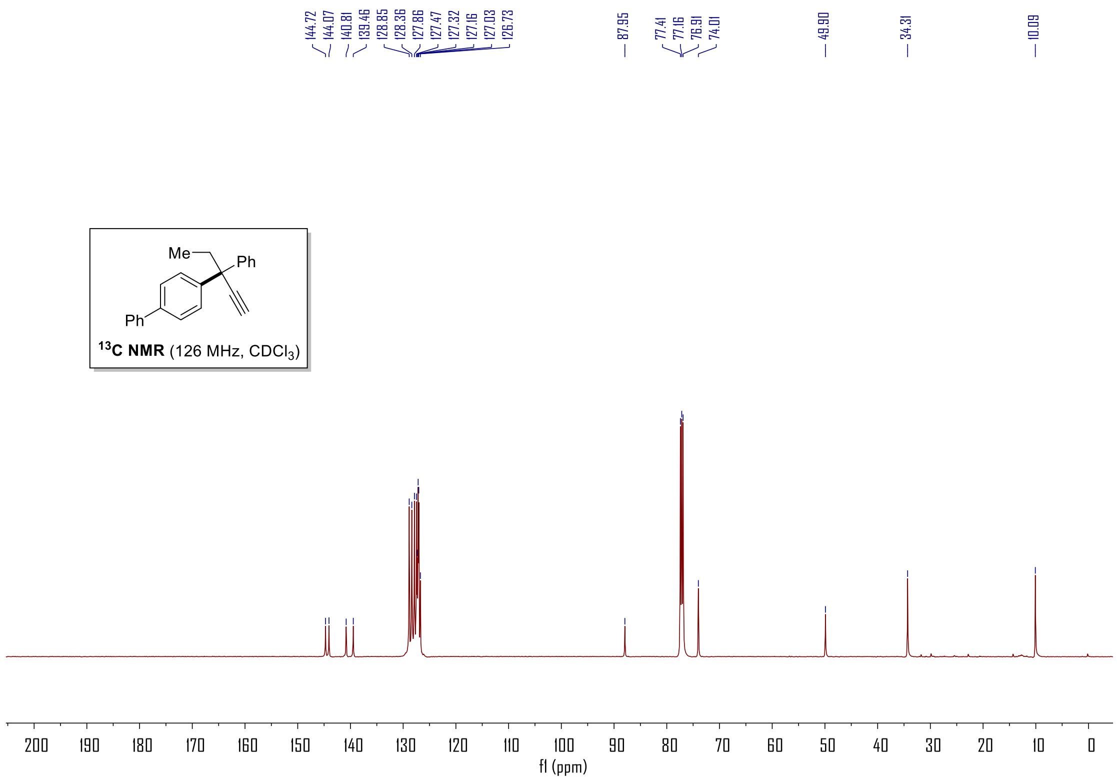

3-(3-Phenylpent-1-yn-3-yl)biphenyl (4ba)

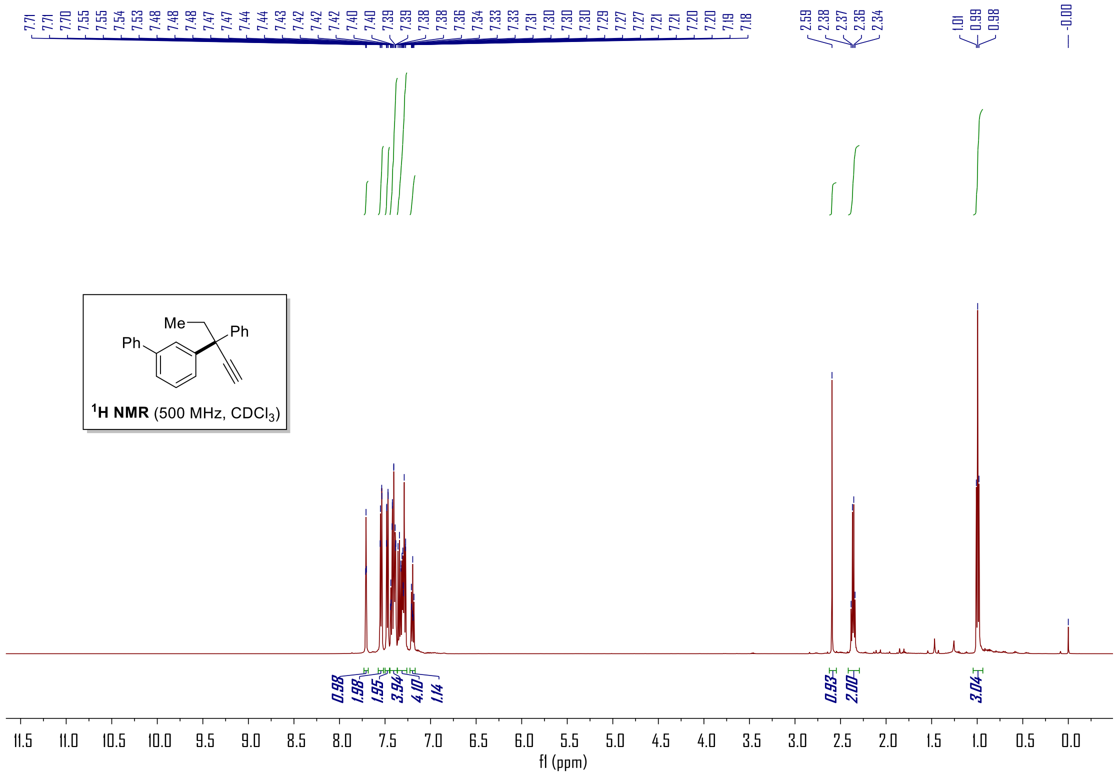

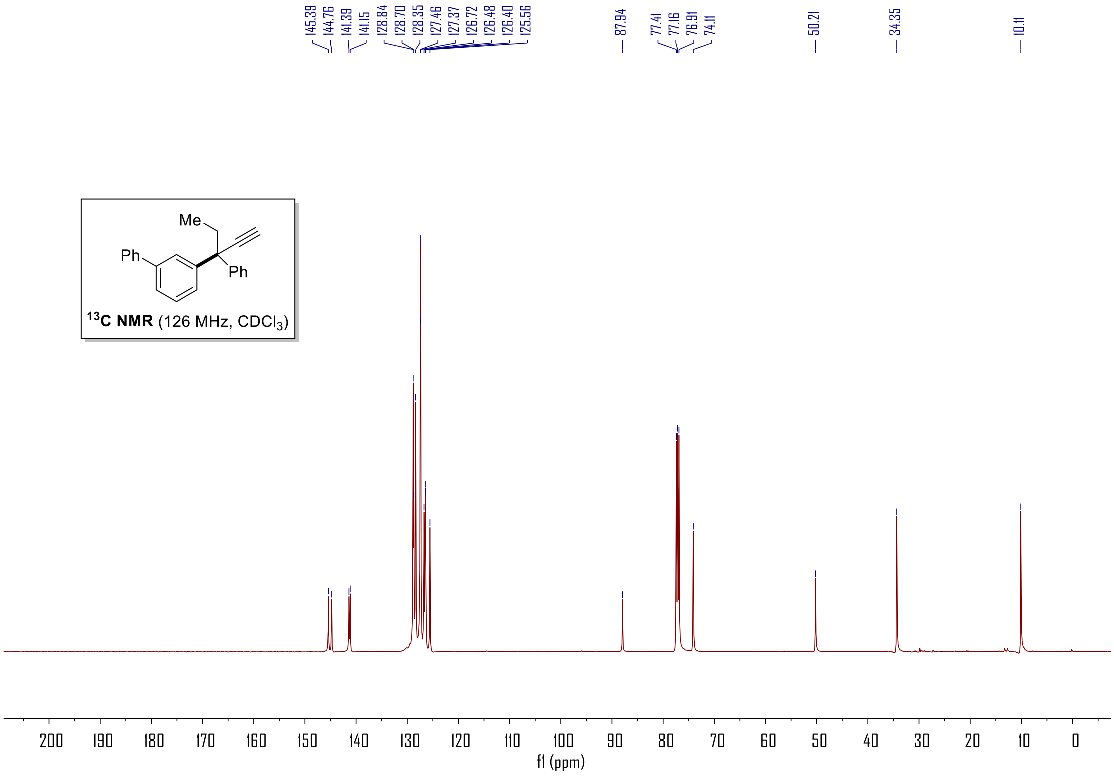

1-Methoxy-2-(3-phenylpent-1-yn-3-yl)benzene (4ca)

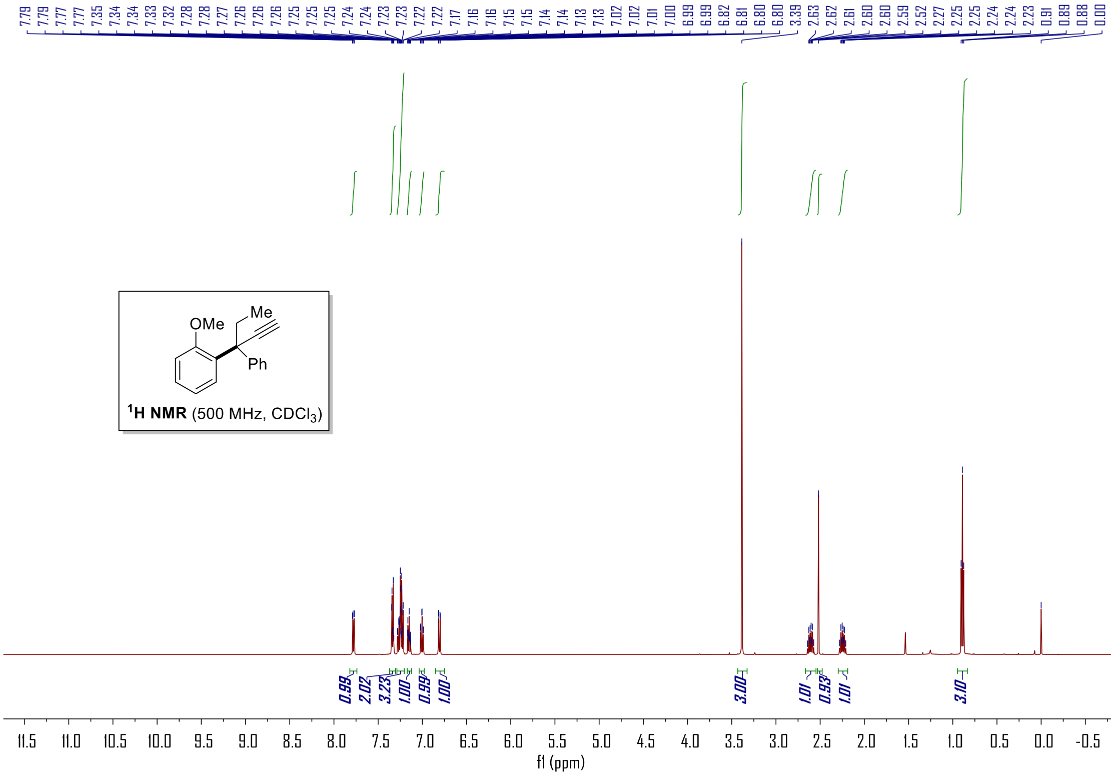

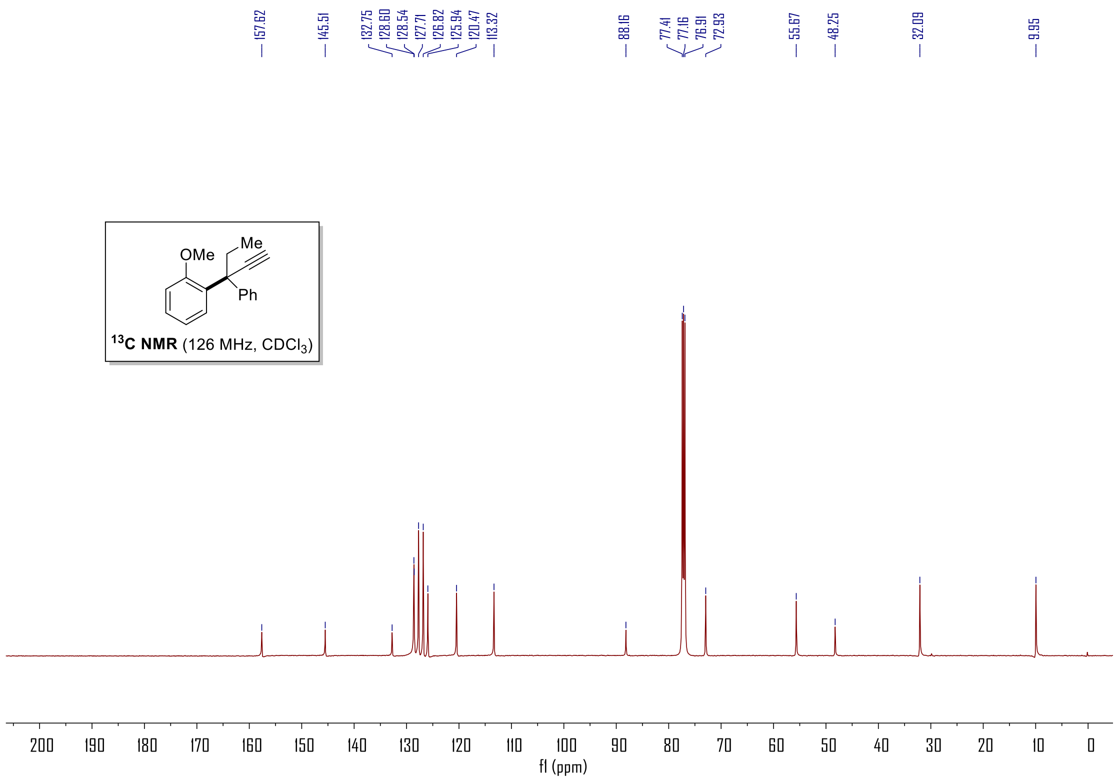

1-Phenoxy-3-(3-phenylpent-1-yn-3-yl)benzene (4da)

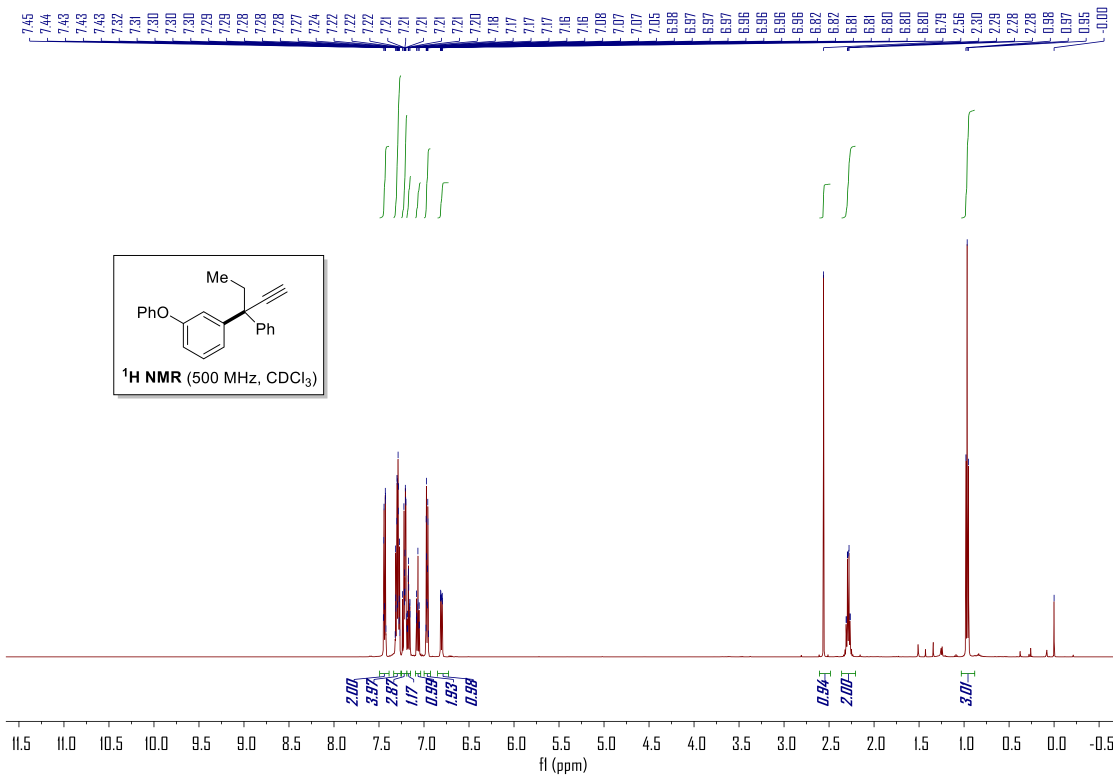

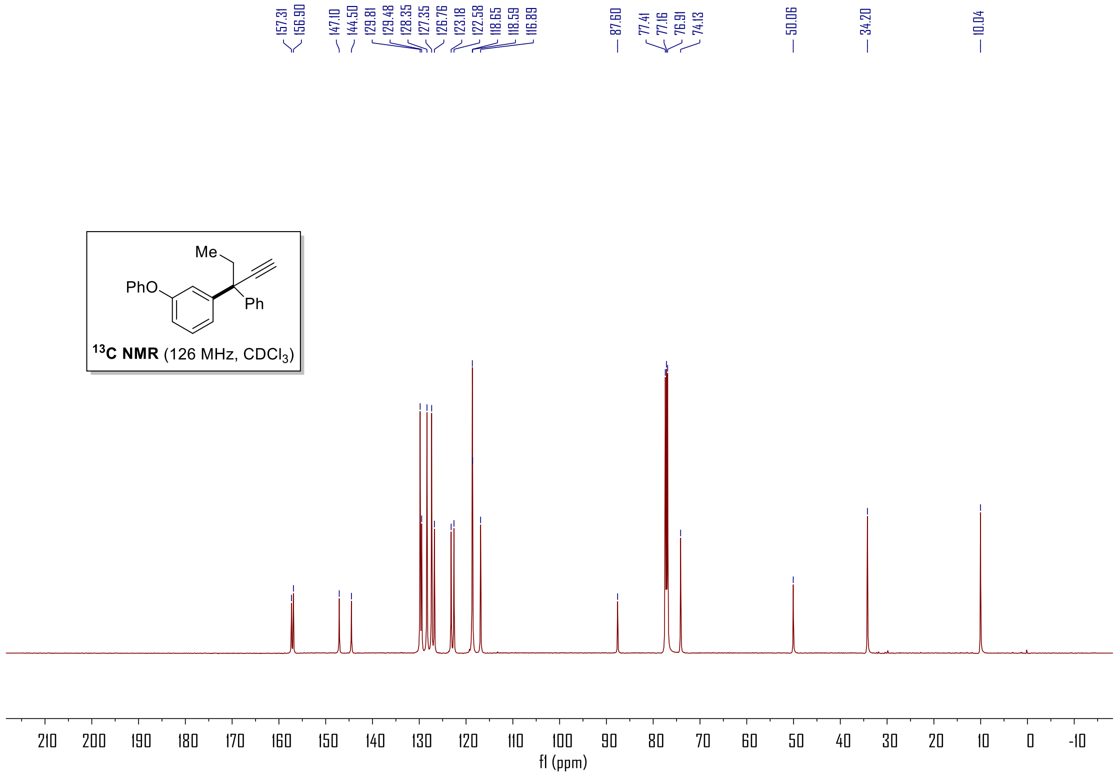

1-Phenoxy-4-(3-phenylpent-1-yn-3-yl)benzene (4ea)

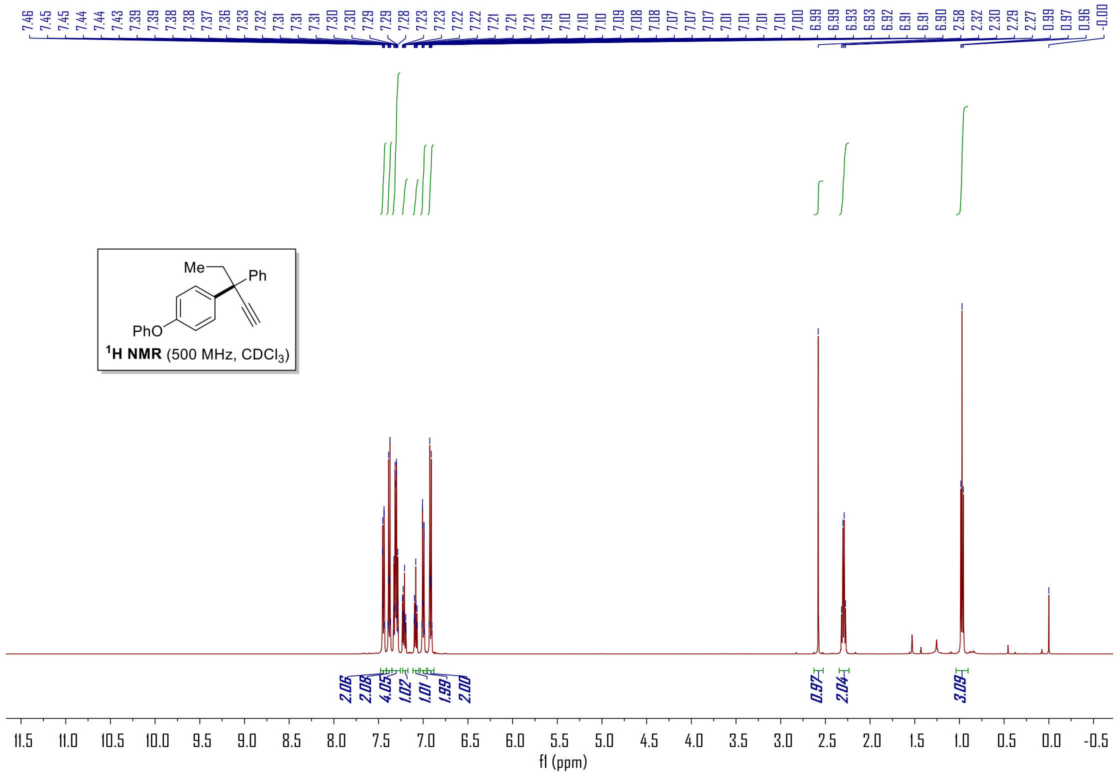

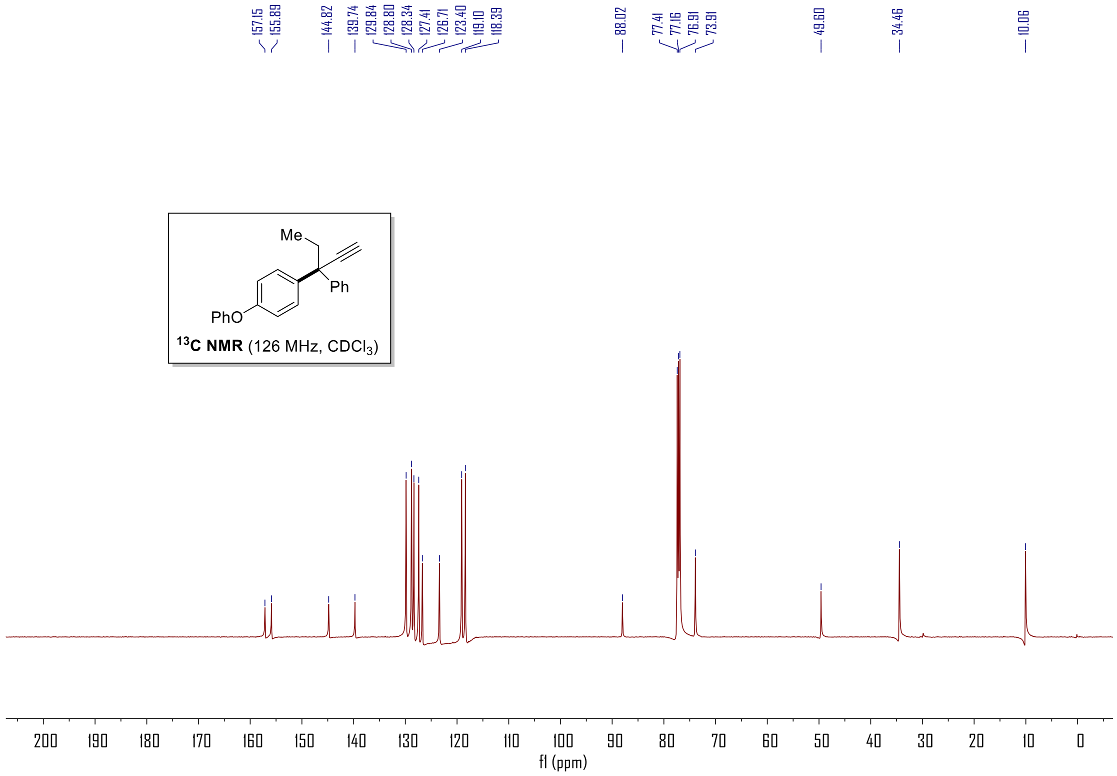

1-(4-(3-Phenylpent-1-yn-3-yl)phenyl)naphthalene (4fa)

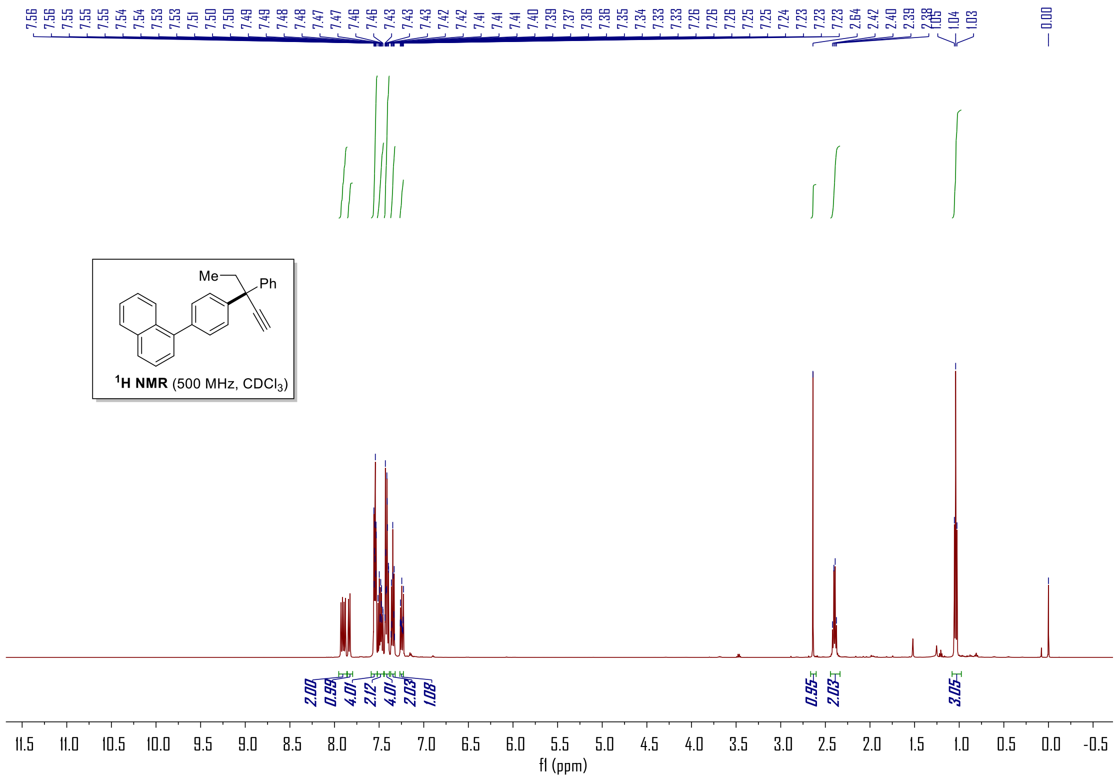

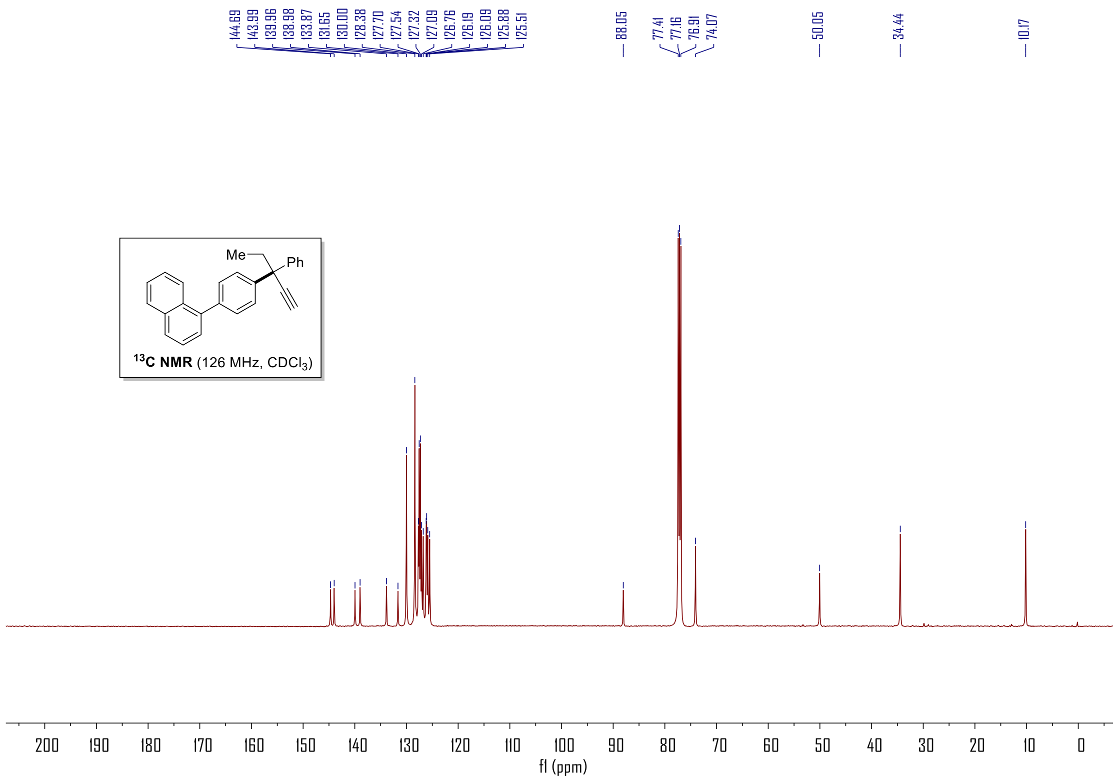

4-Methyl-4'-(3-phenylpent-1-yn-3-yl)biphenyl (4ga)

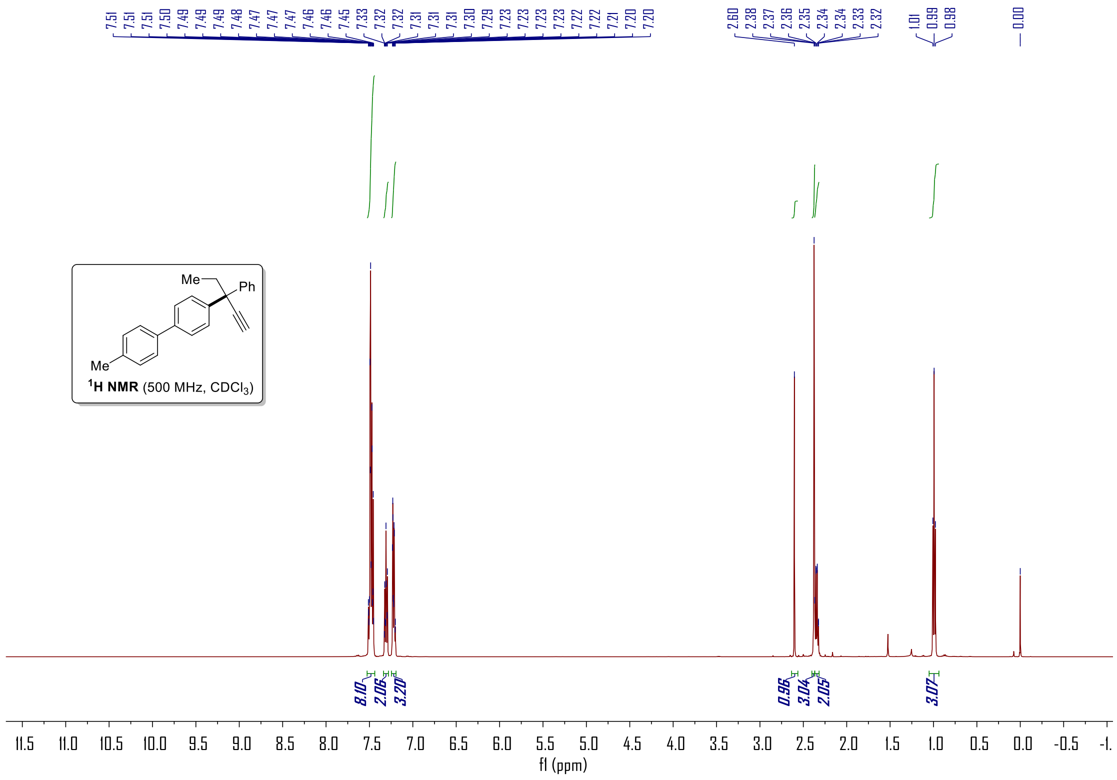

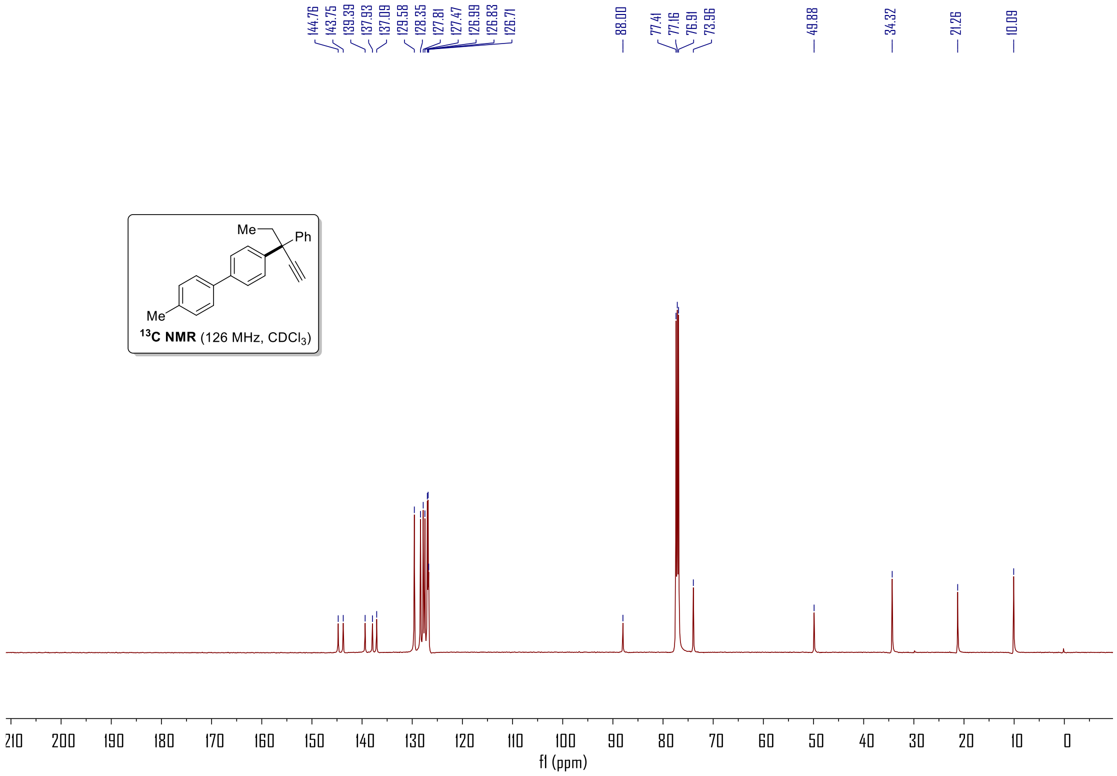

4-Methoxy-4'-(3-phenylpent-1-yn-3-yl)biphenyl (4ha)

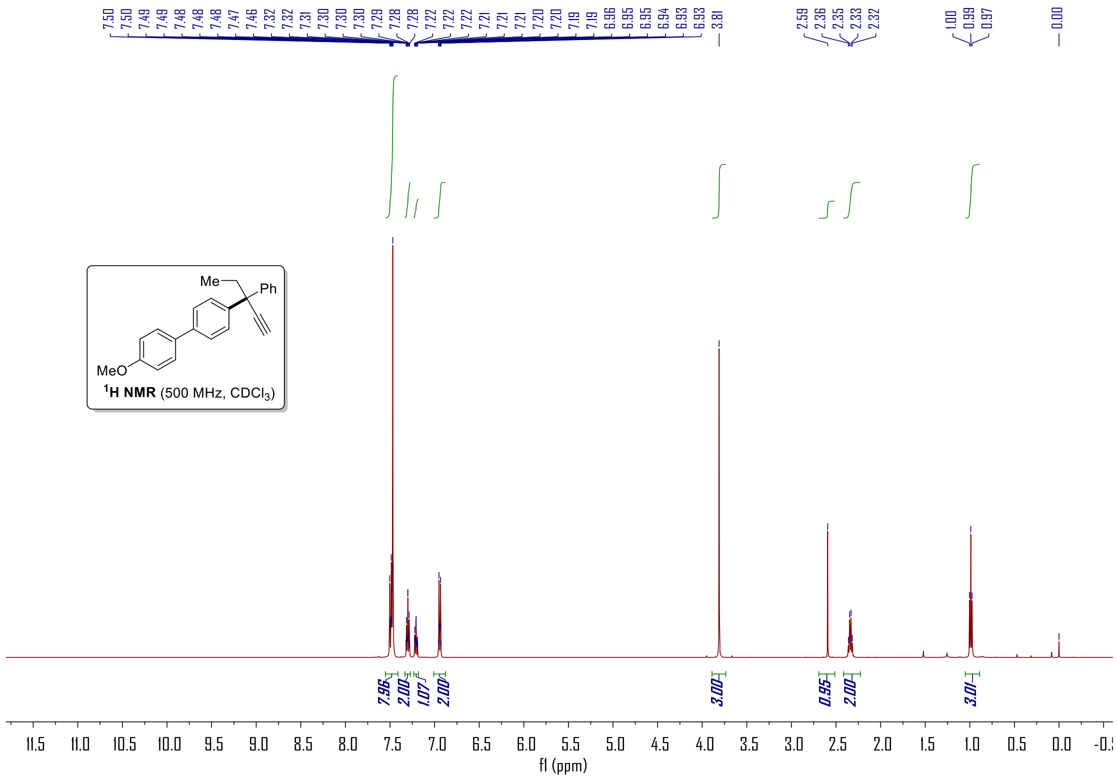

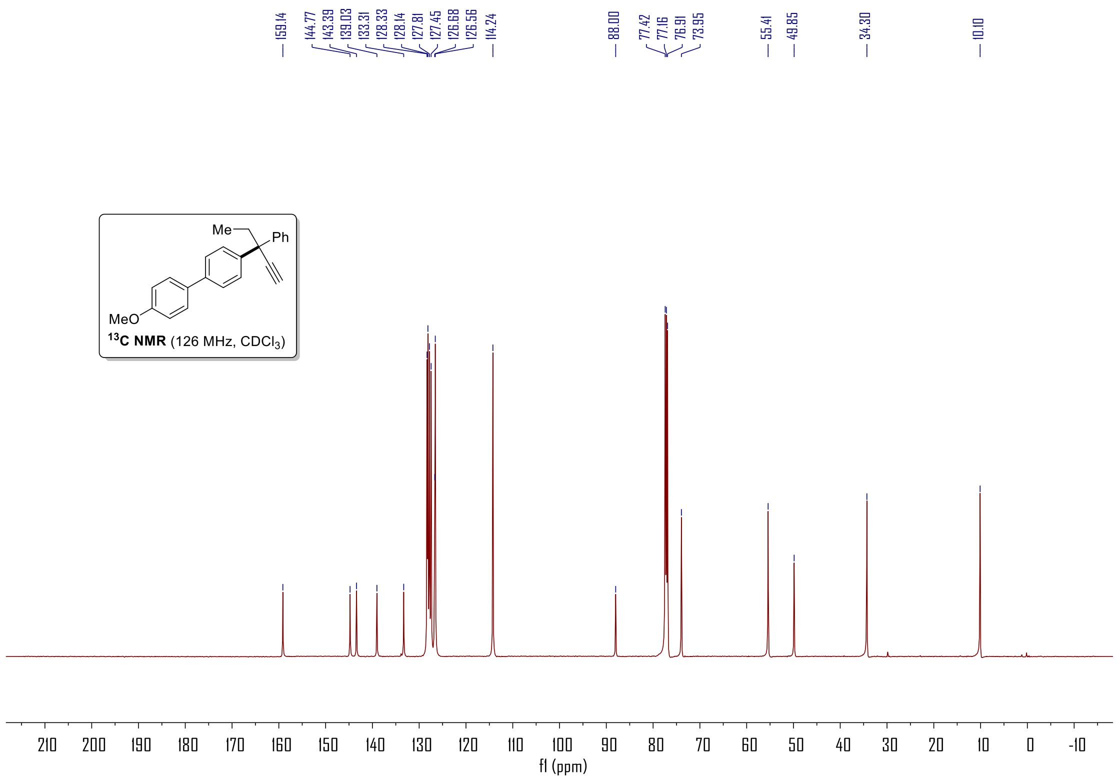

4-(Benzyloxy)-4'-(3-phenylpent-1-yn-3-yl)biphenyl (4ia)

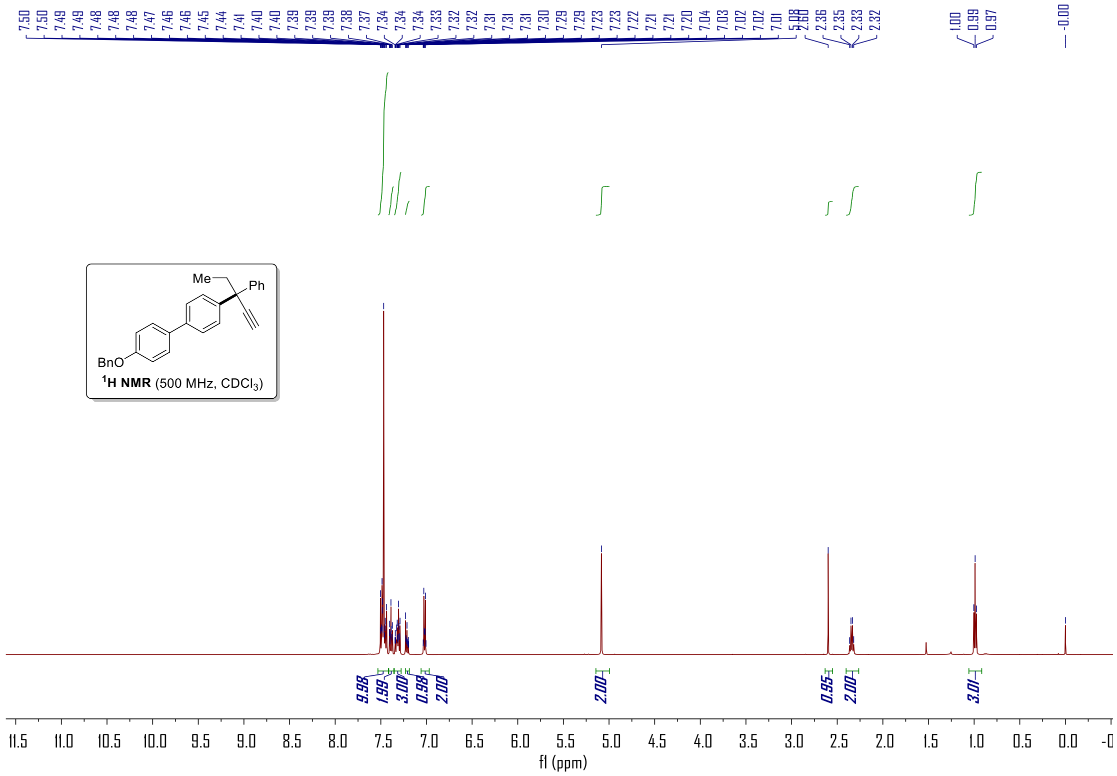

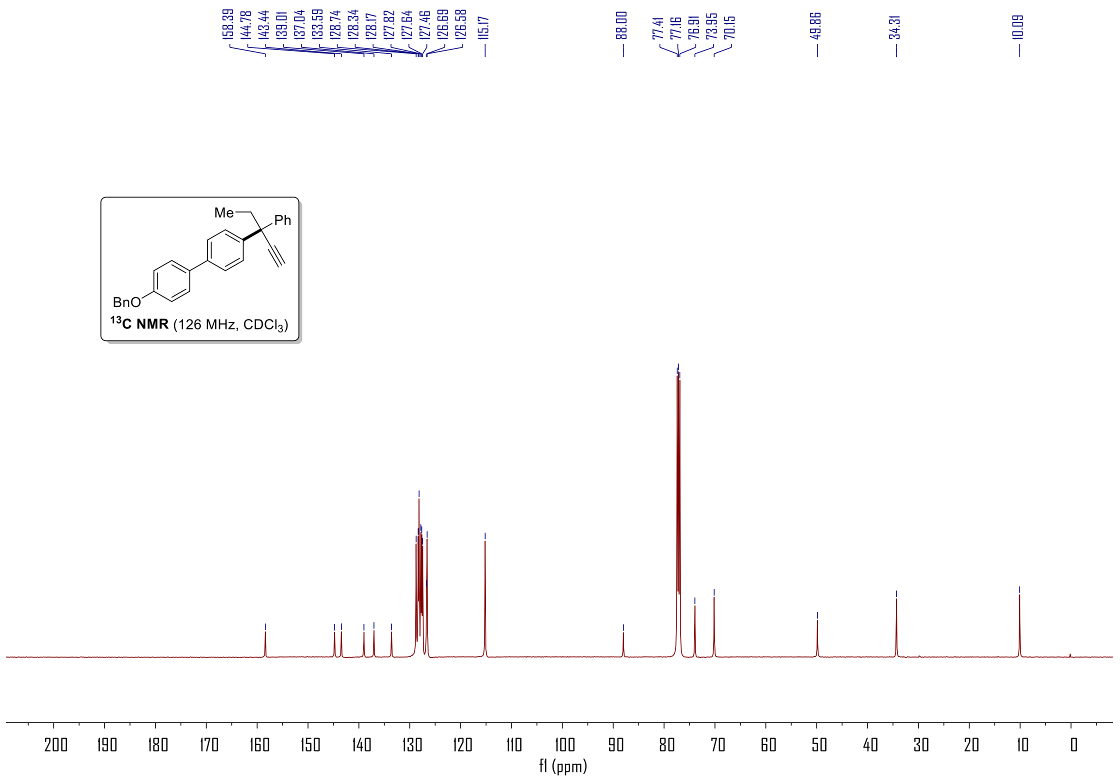

5-(4-(3-Phenylpent-1-yn-3-yl)phenyl)benzo[*d*][1,3]dioxole (4ja)

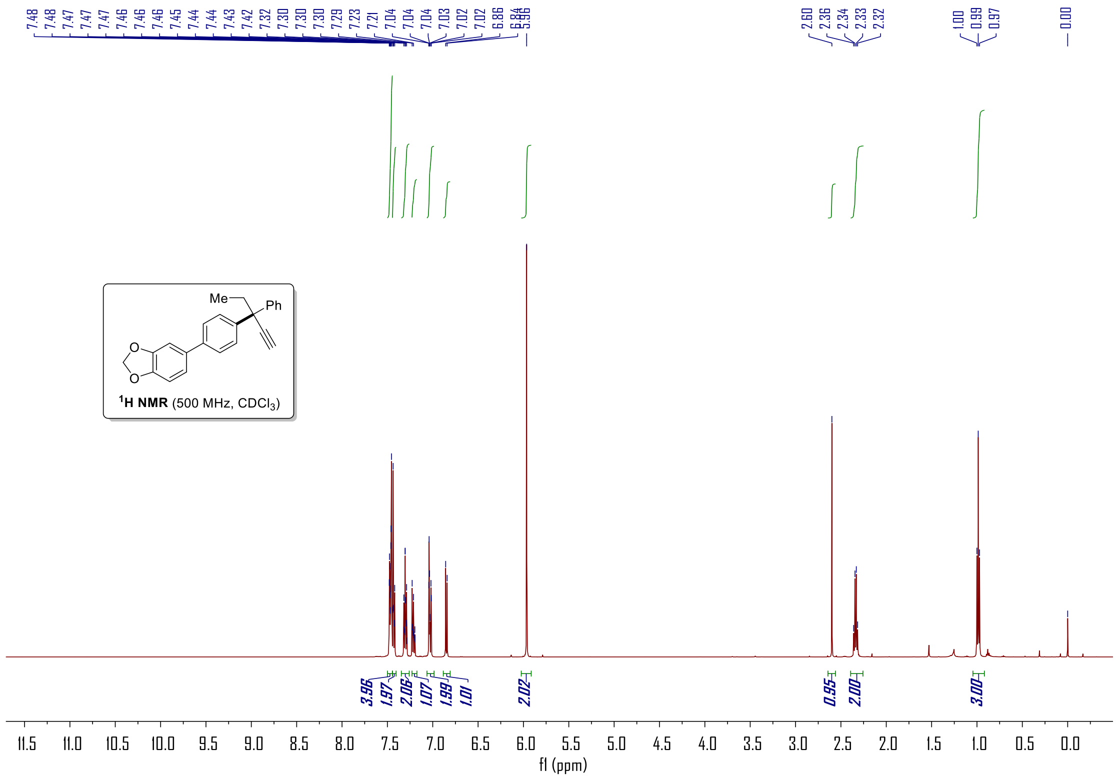

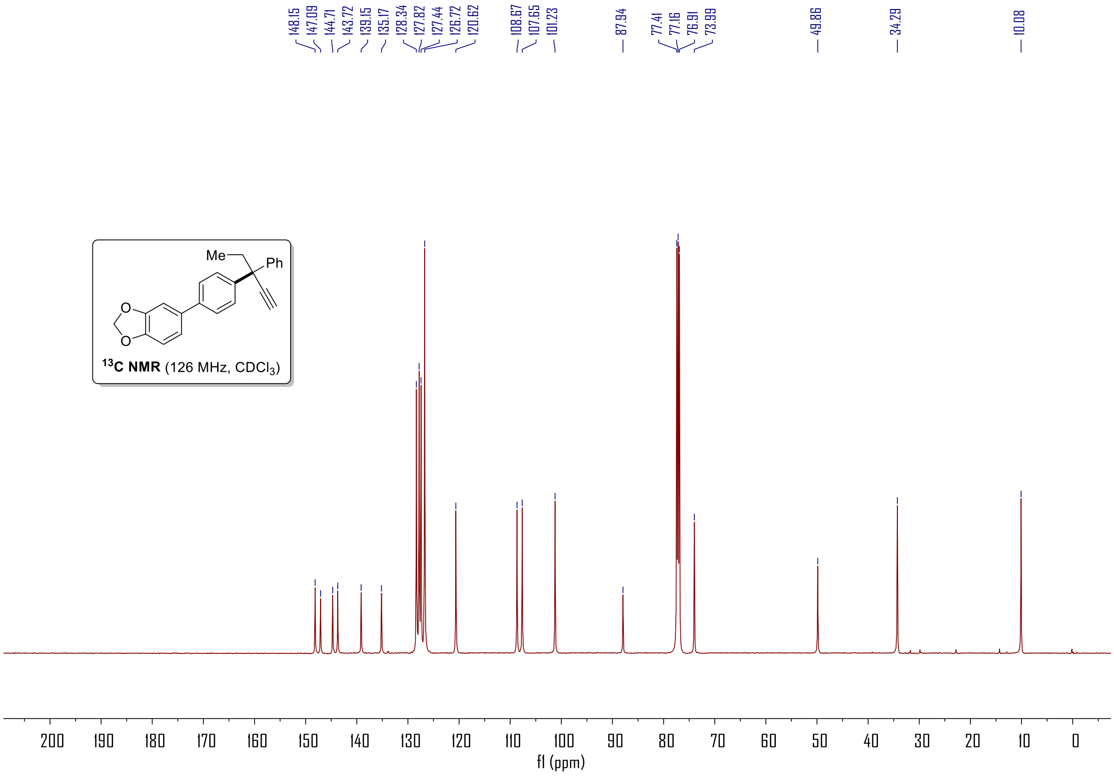

4-Chloro-4'-(3-phenylpent-1-yn-3-yl)biphenyl (4ka)

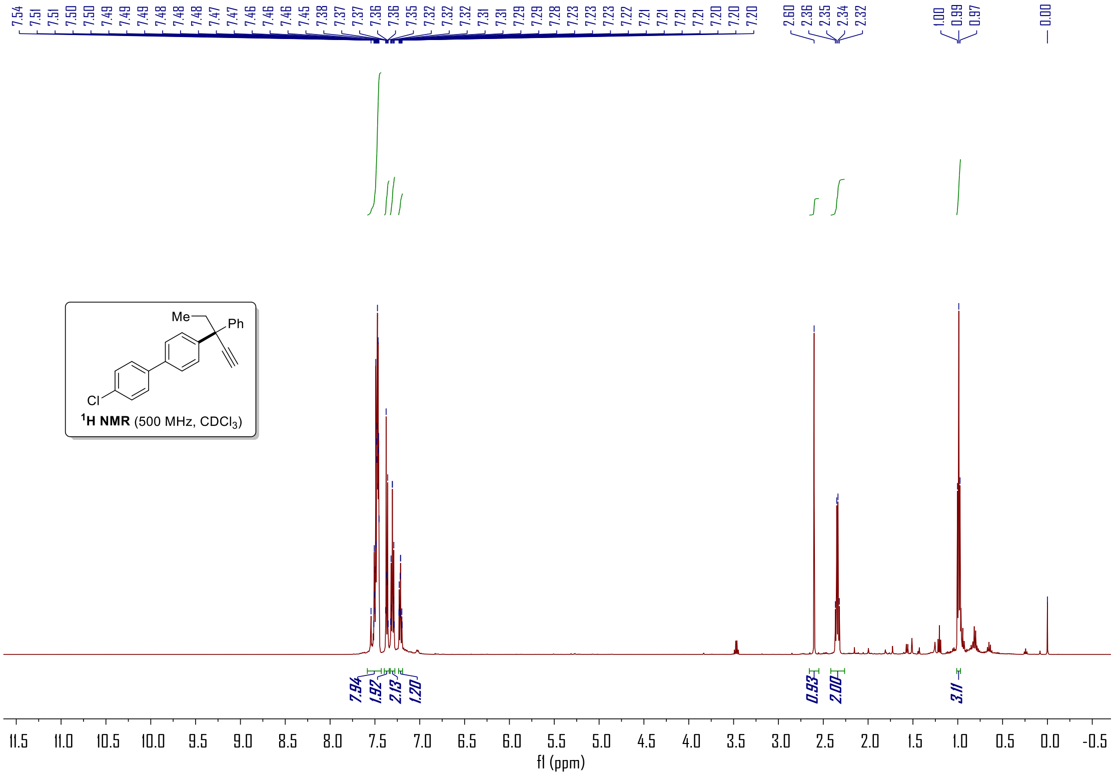

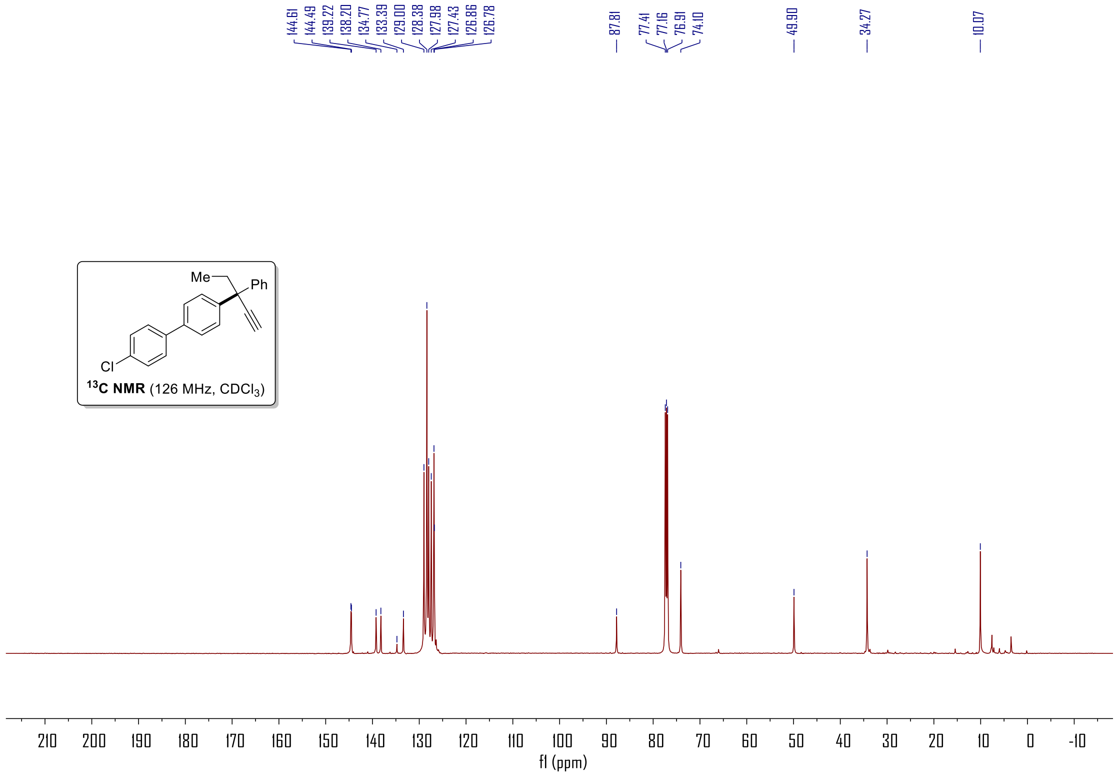

4-(3-Phenylpent-1-yn-3-yl)-4'-(trifluoromethyl)biphenyl (4la)

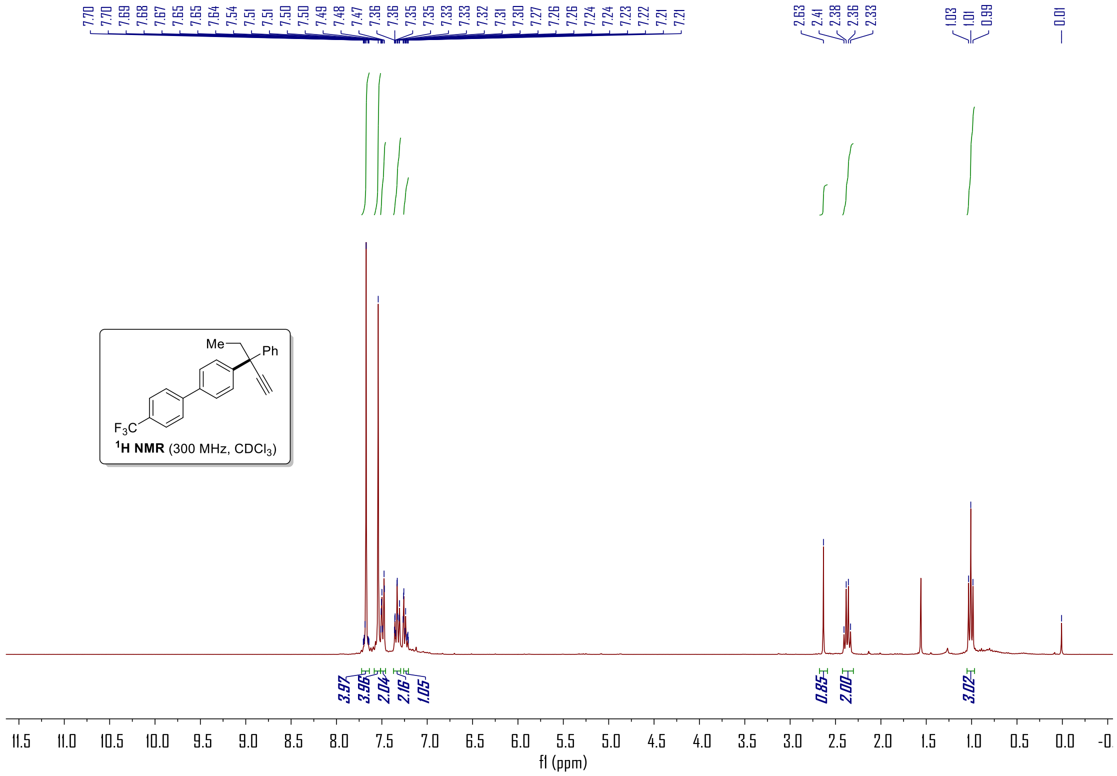

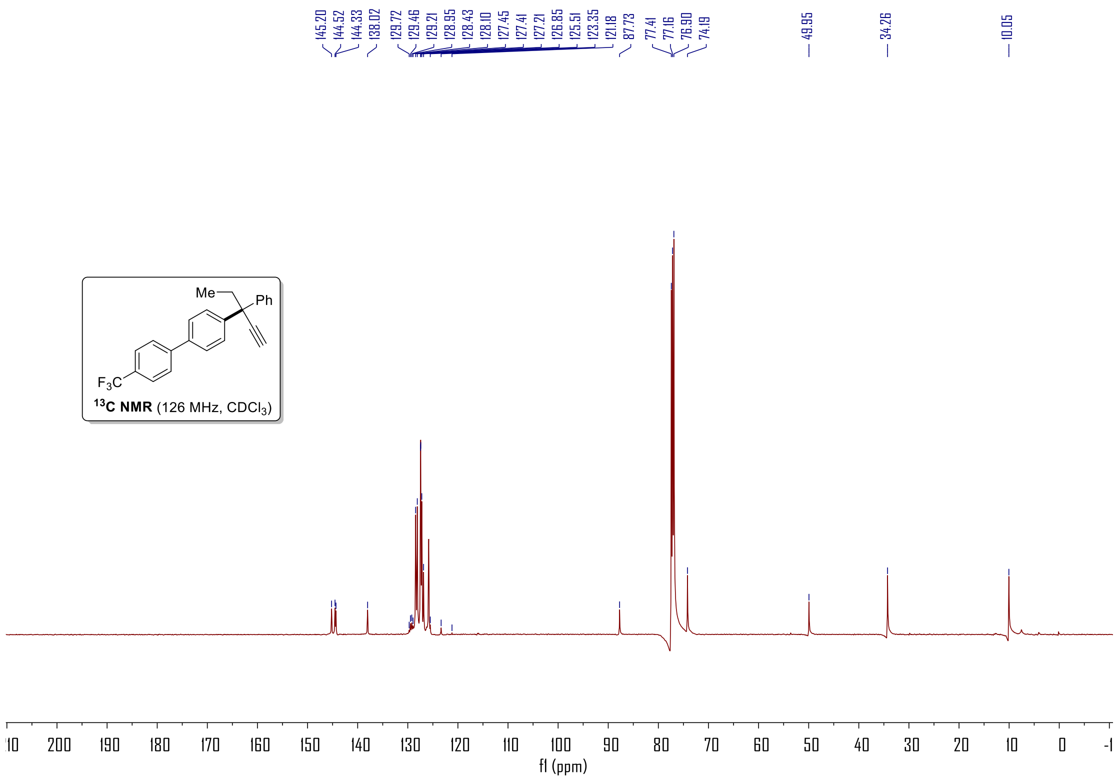
145.20
144.52
144.33
138.02
129.72
129.46
129.21
128.95
128.43
128.10
127.45
127.41
127.21
126.85
125.51
123.35
121.18
87.73
77.41
77.16
76.90
74.19
49.95
34.26
10.05
Me
Ph
F_3C
^{13}C NMR (126 MHz, $CDCl_3$)
f1 (ppm)

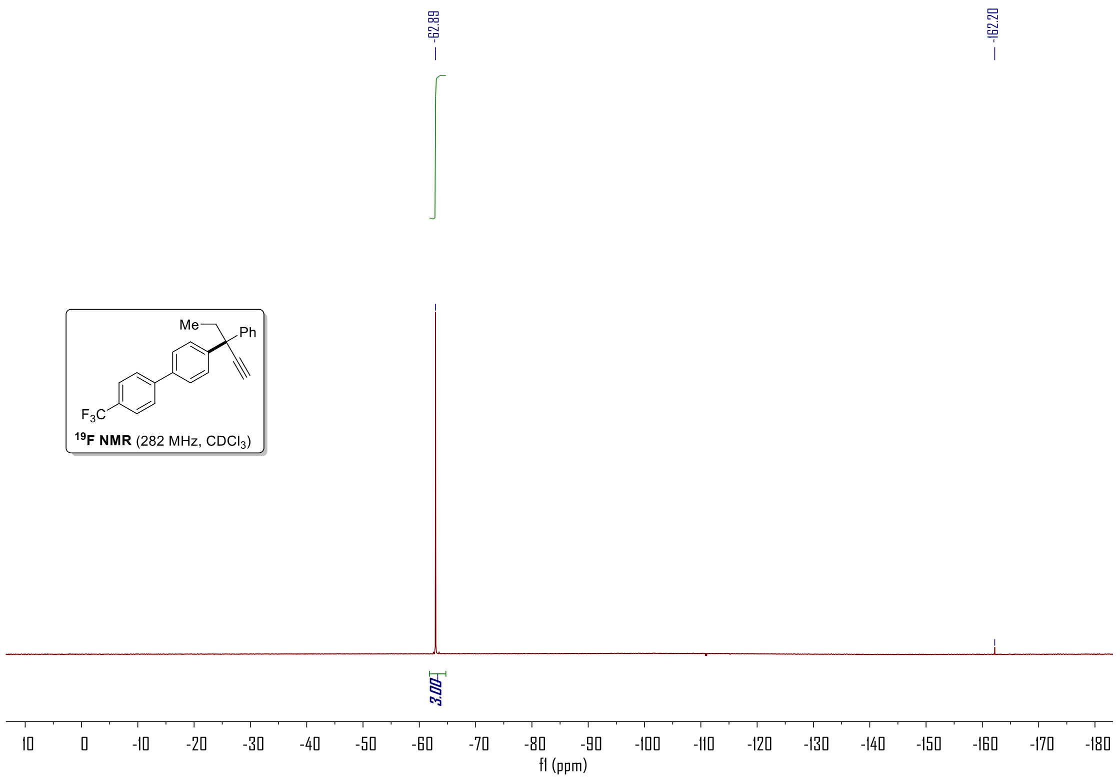
-62.89
-162.20
Me
Ph
F_3C
^{19}F NMR (282 MHz, $CDCl_3$)
3.00
f1 (ppm)

1-(3-Methoxypropyl)-3-(3-phenylpent-1-yn-3-yl)benzene (4ma)

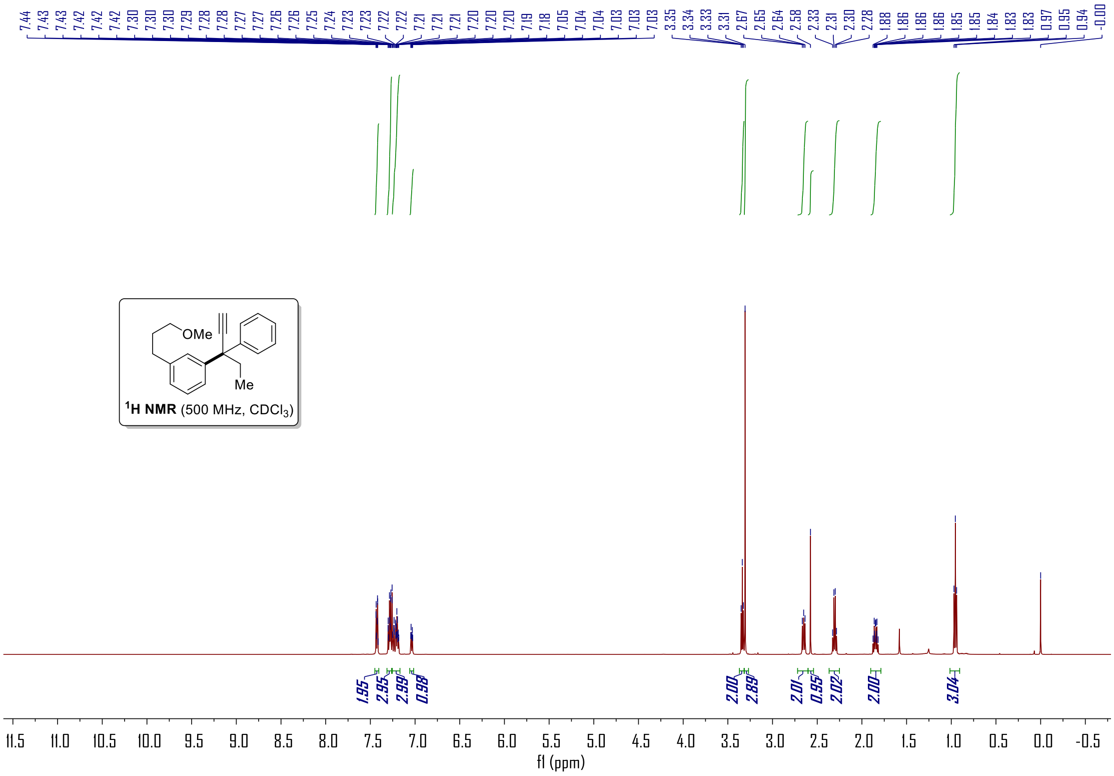

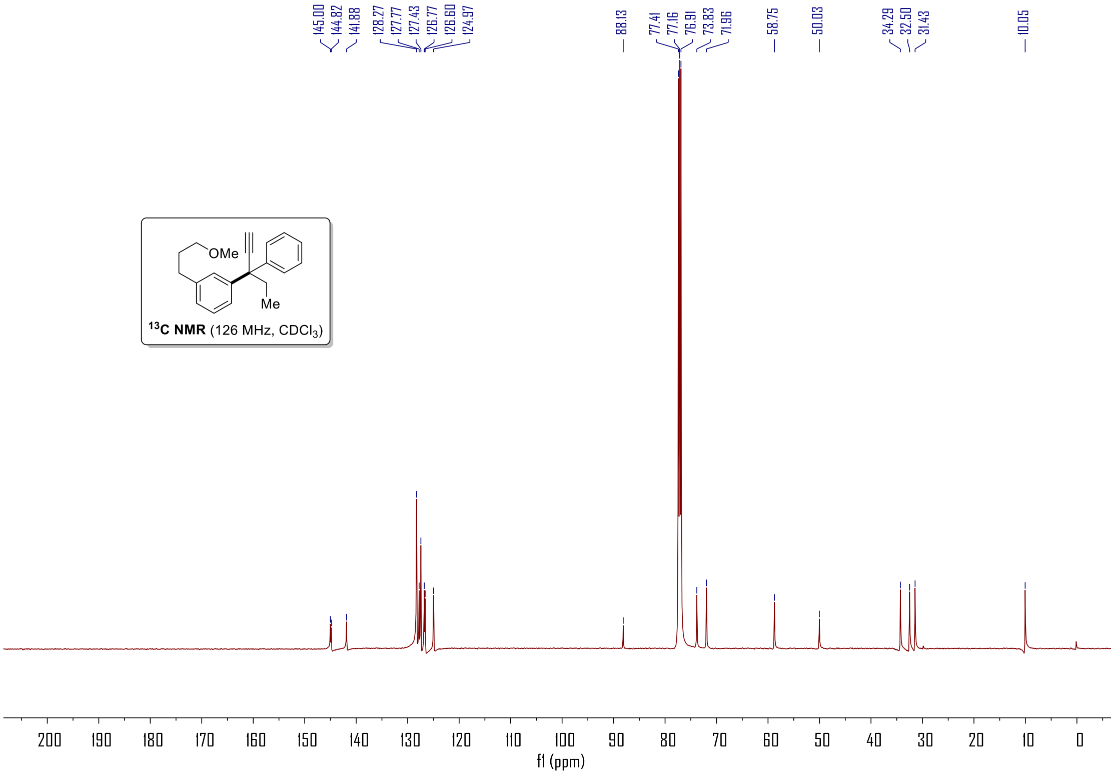

4-(4-(3-Phenylpent-1-yn-3-yl)phenyl)pyridine (4na)

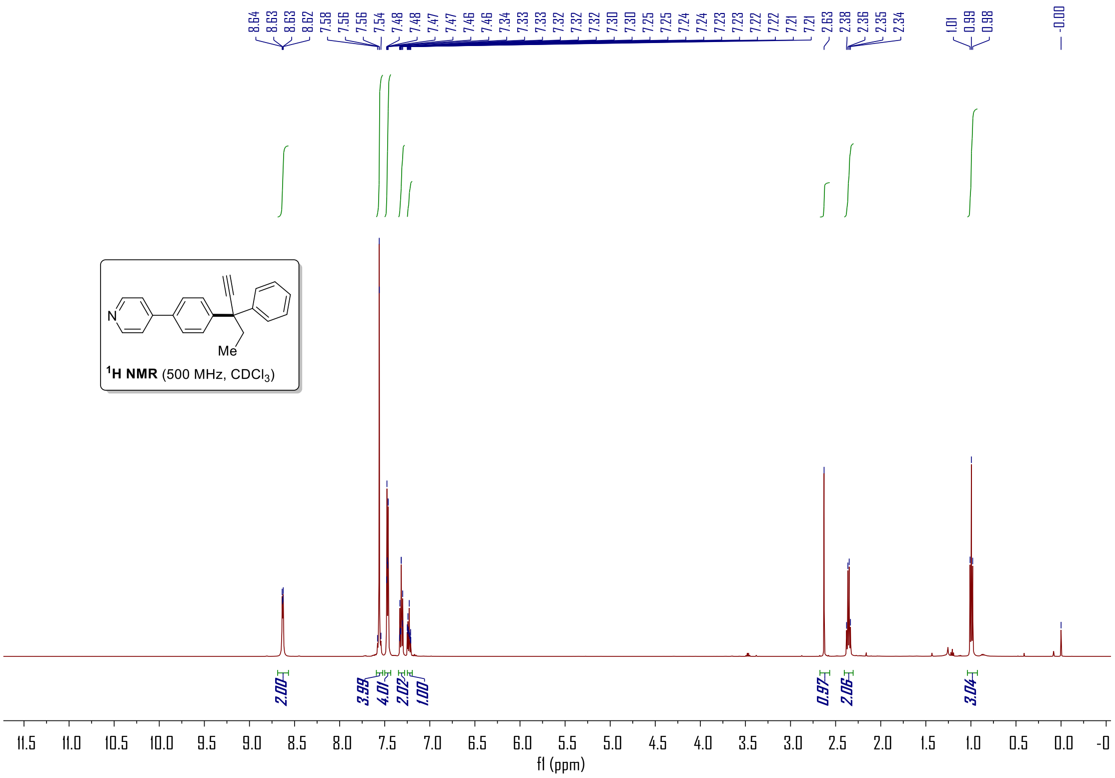

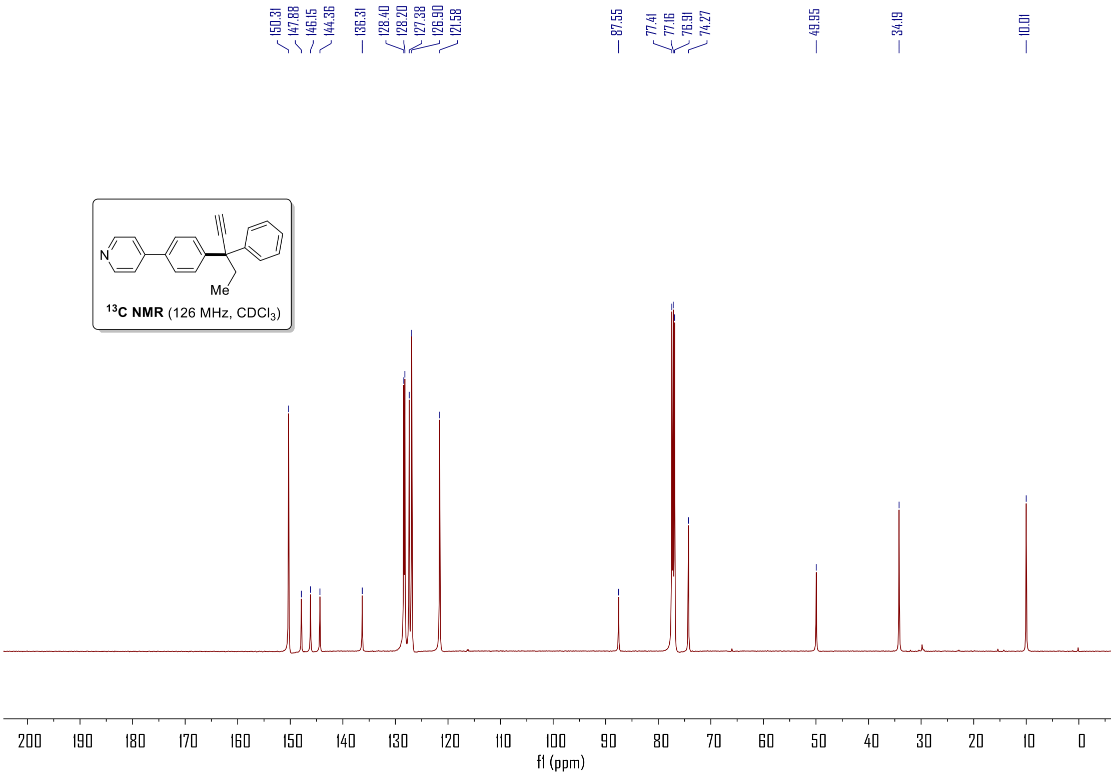

2-Phenyl-5-(3-phenylpent-1-yn-3-yl)pyridine (4oa)

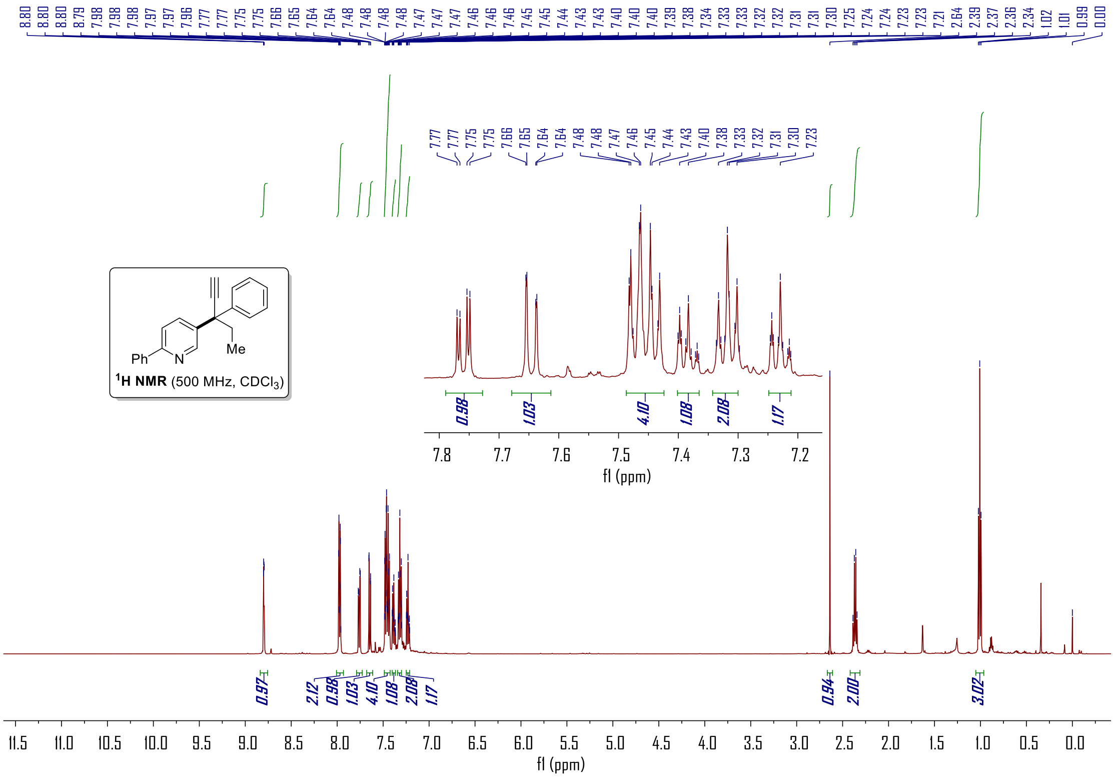

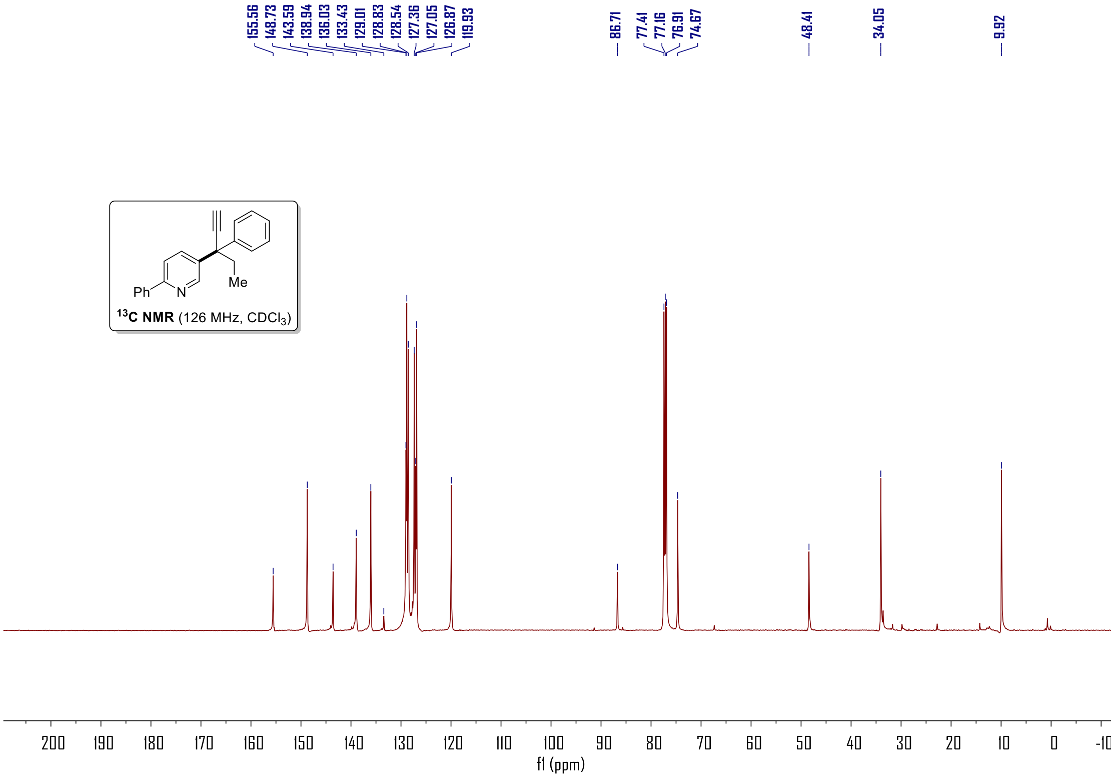

2-Phenyl-4-(3-phenylpent-1-yn-3-yl)pyridine (4pa)

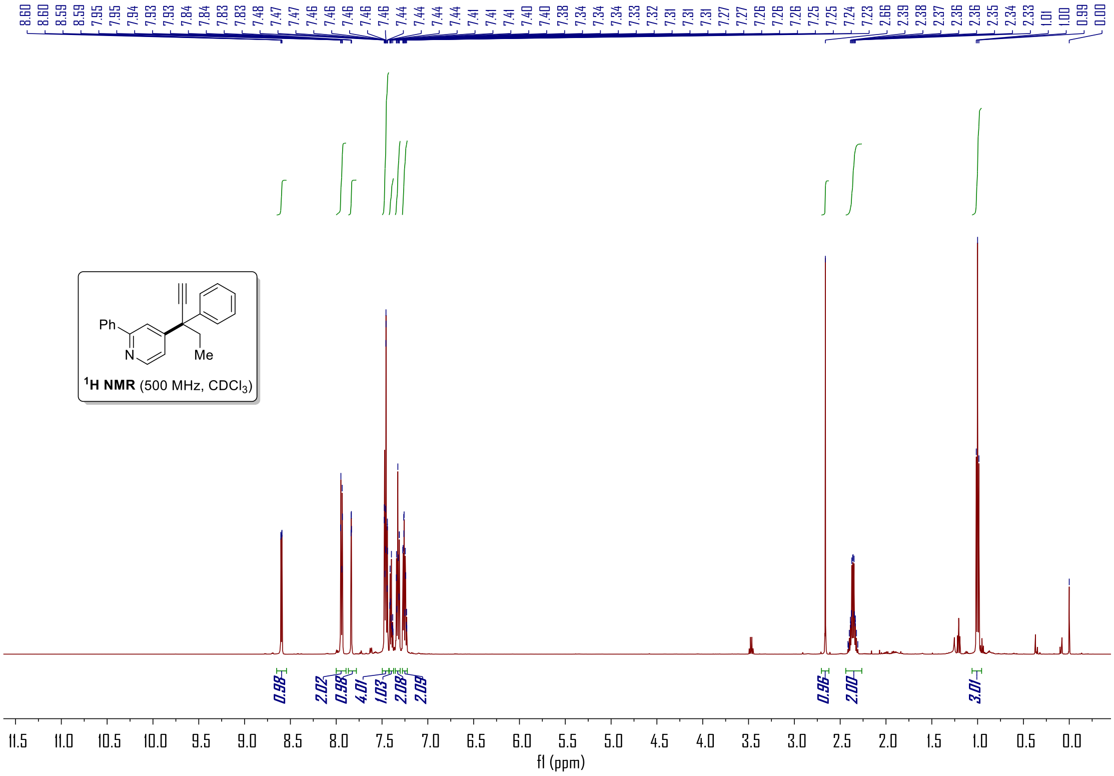

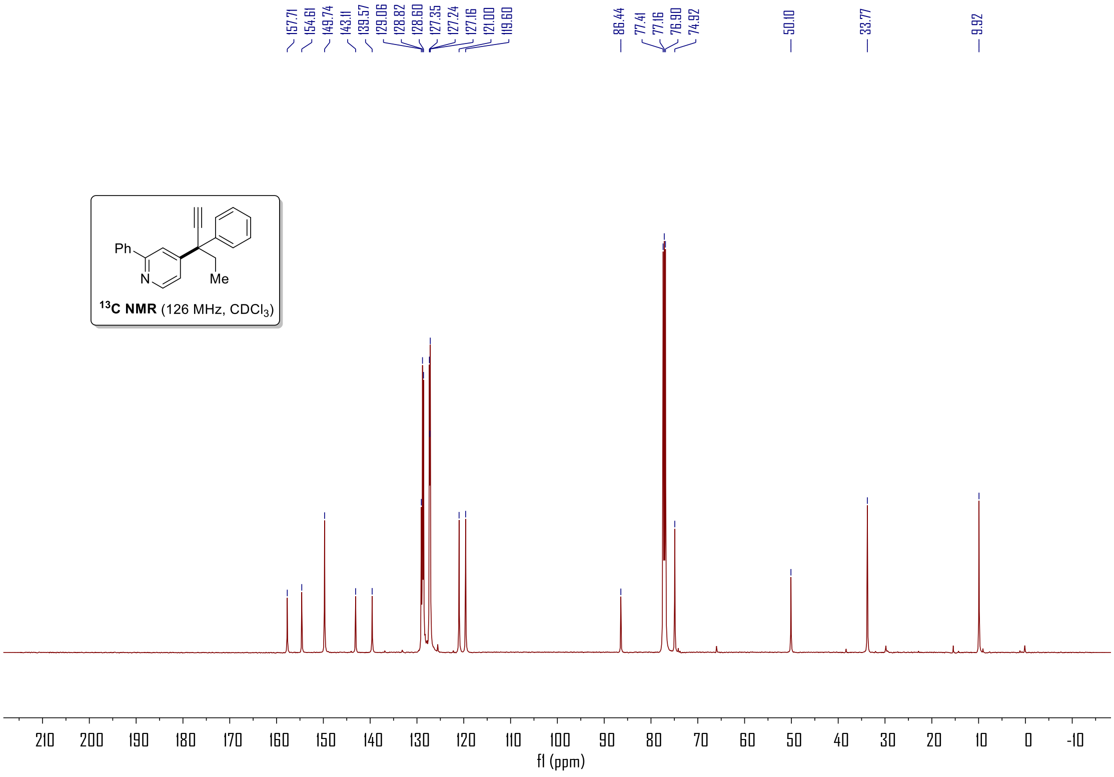

1-(4-(3-Phenylpent-1-yn-3-yl)phenyl)-1*H*-pyrrole (4qa)

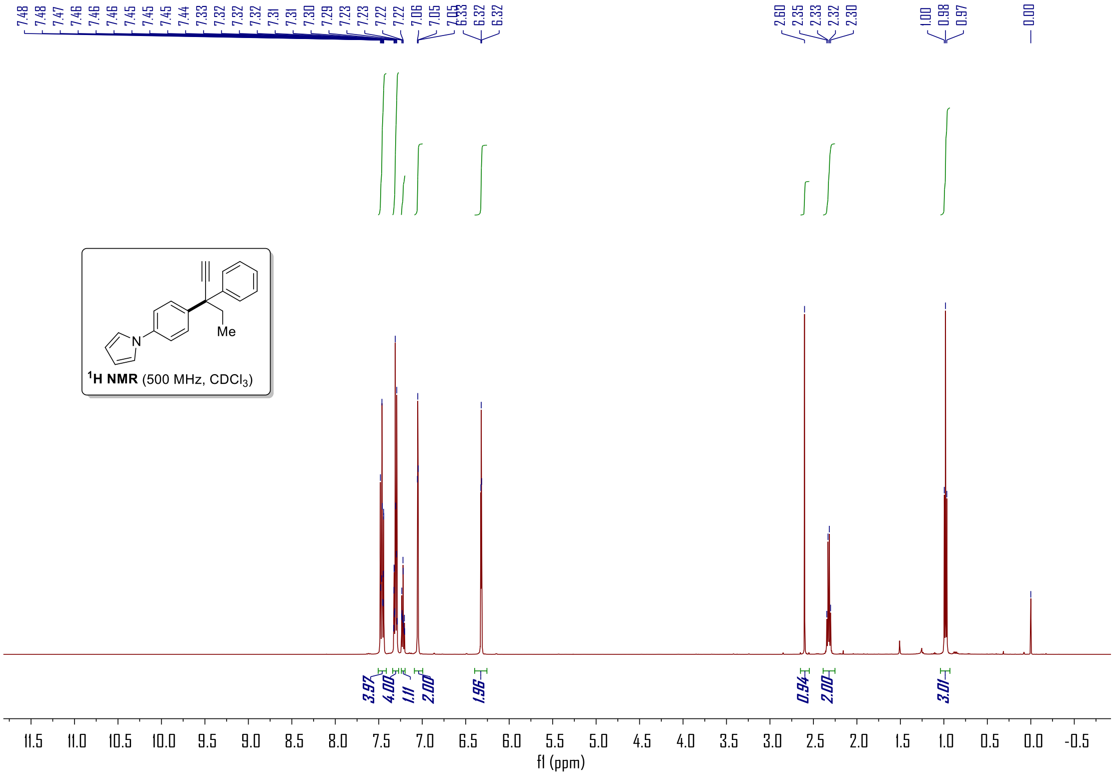

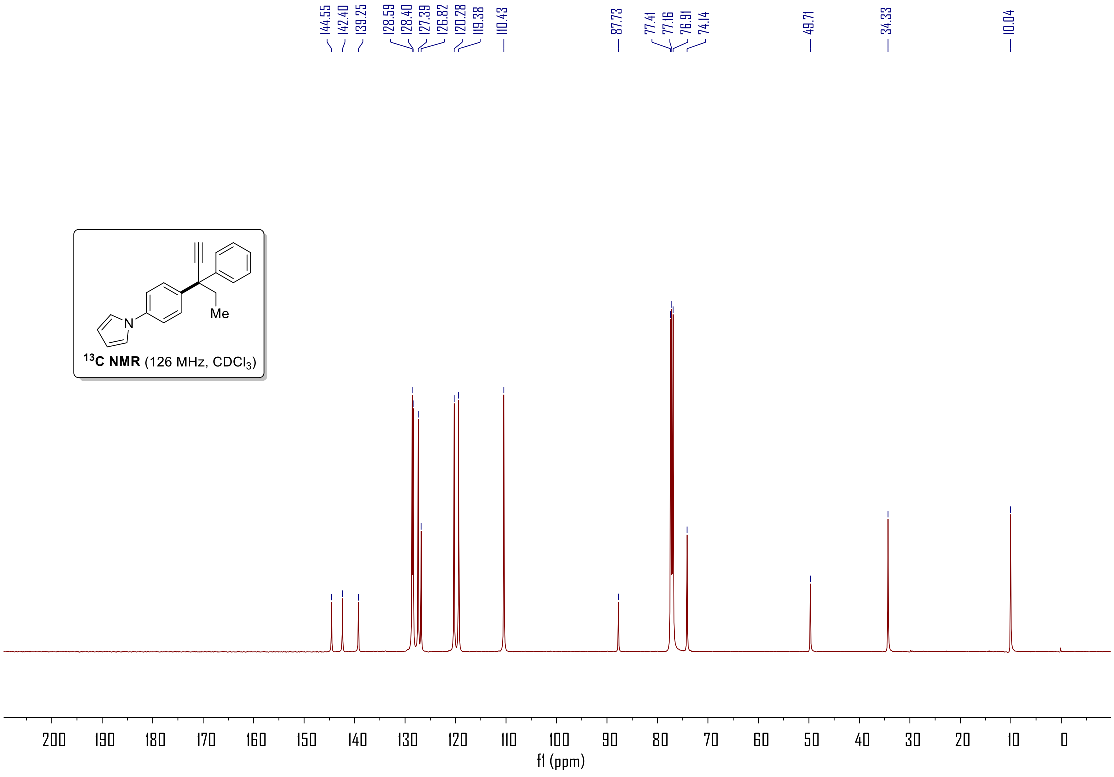

1-Methyl-2-(4-(3-phenylpent-1-yn-3-yl)phenyl)-1*H*-indole (4ra)

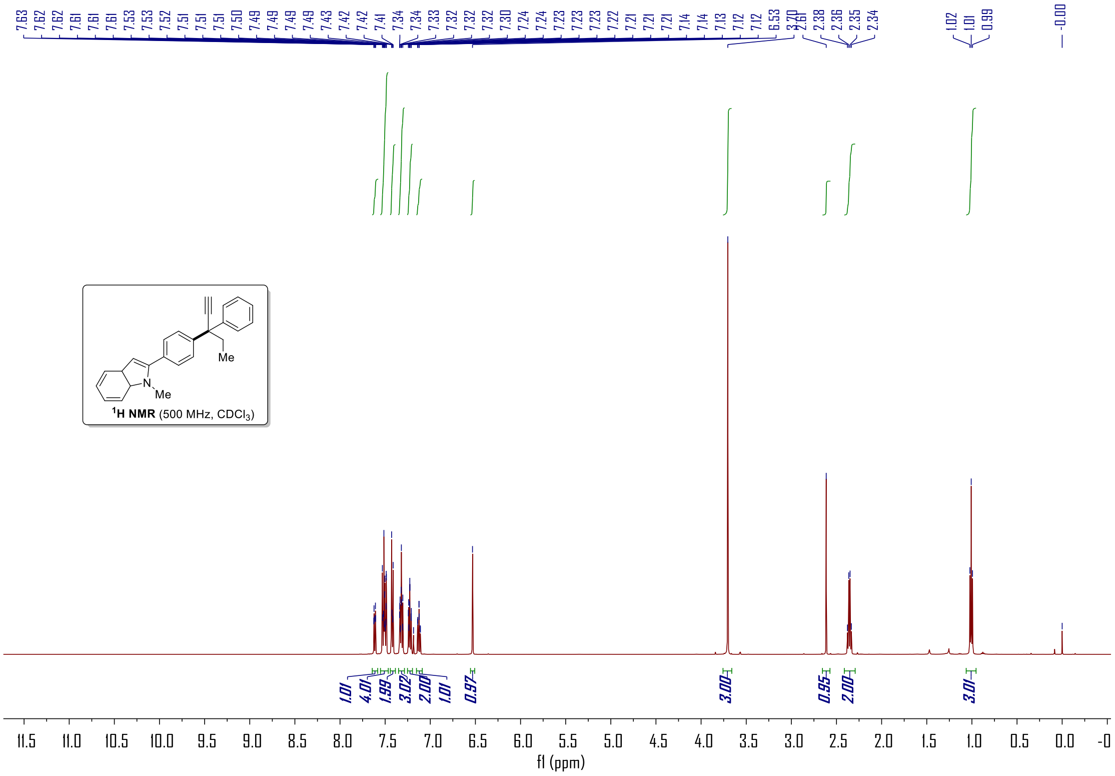

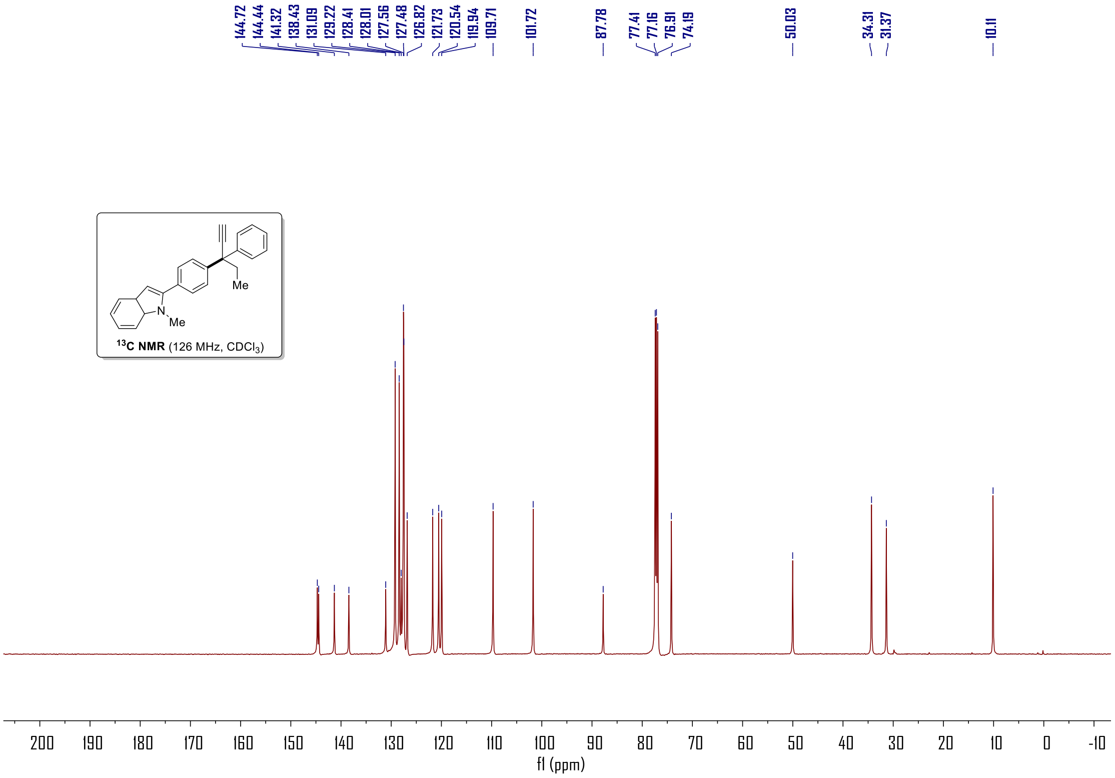

2-(4-(3-Phenylpent-1-yn-3-yl)phenyl)benzofuran (4sa)

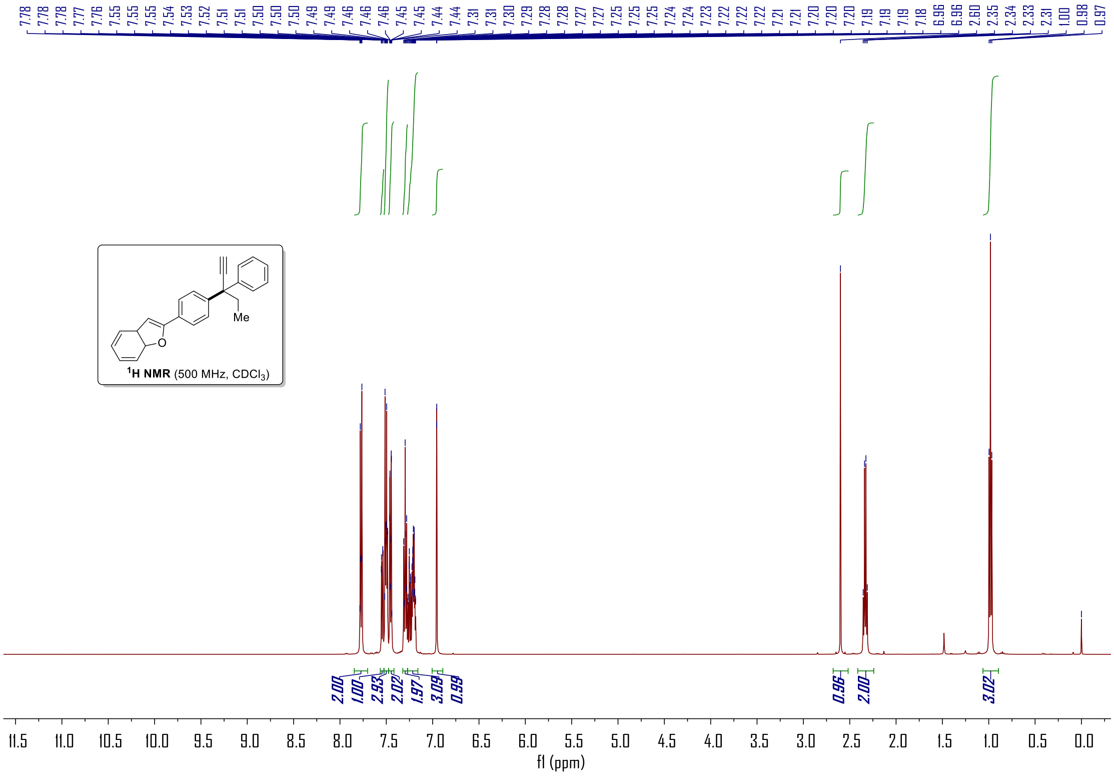

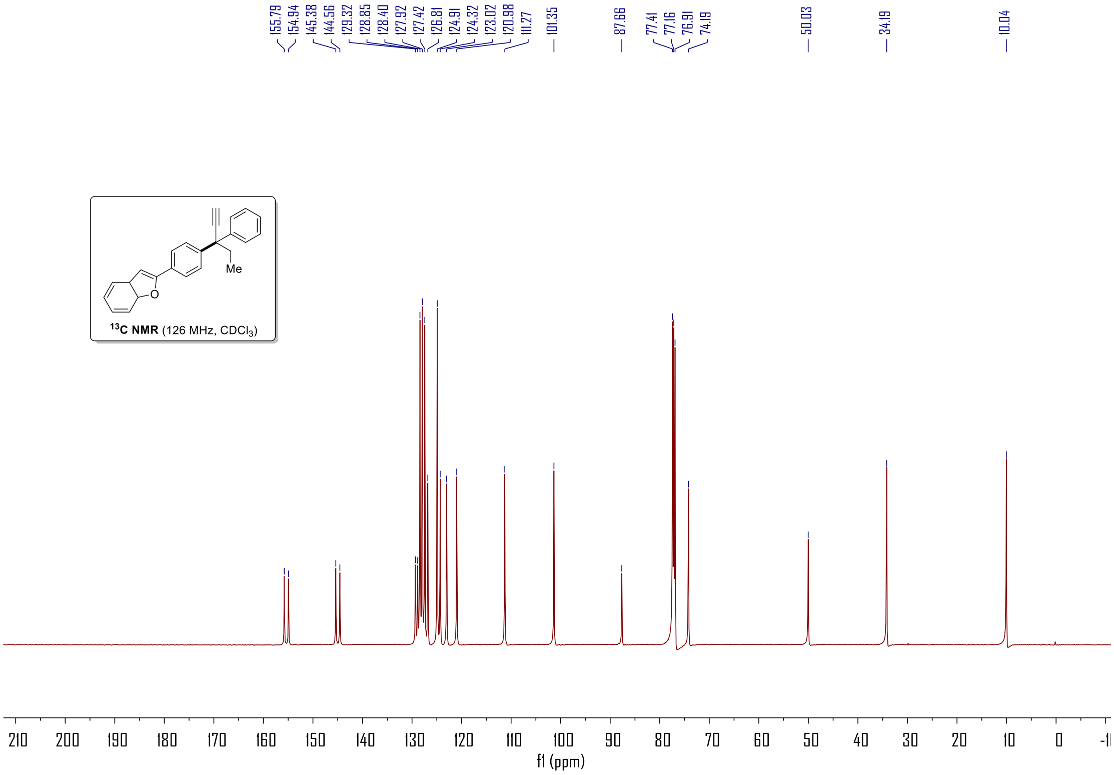

4-(2-Phenylbut-3-yn-2-yl)biphenyl (4ab)

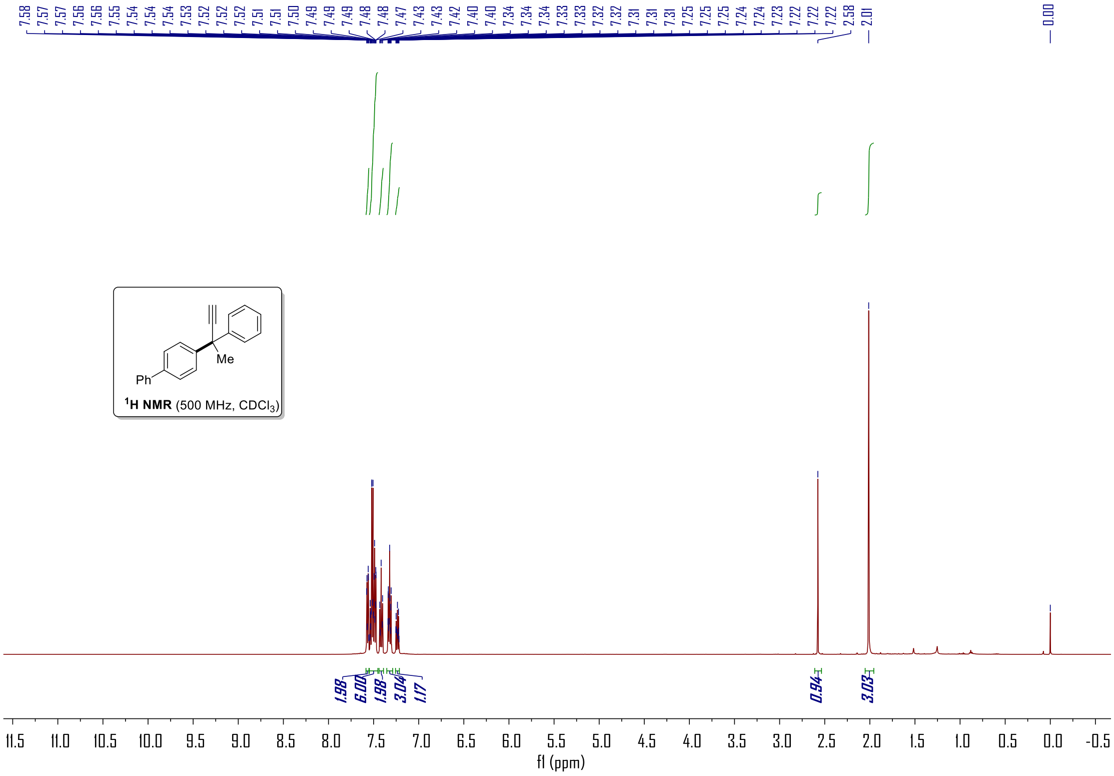

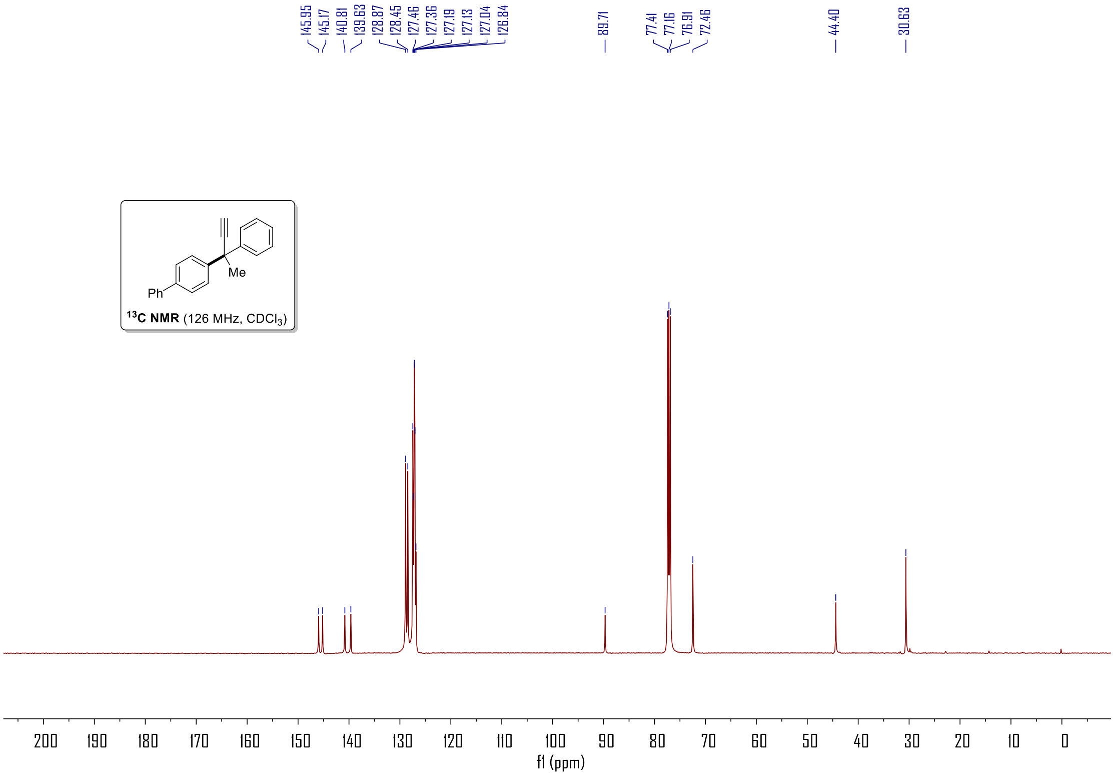

4-(3-Phenyloct-1-yn-3-yl)biphenyl (4ac)

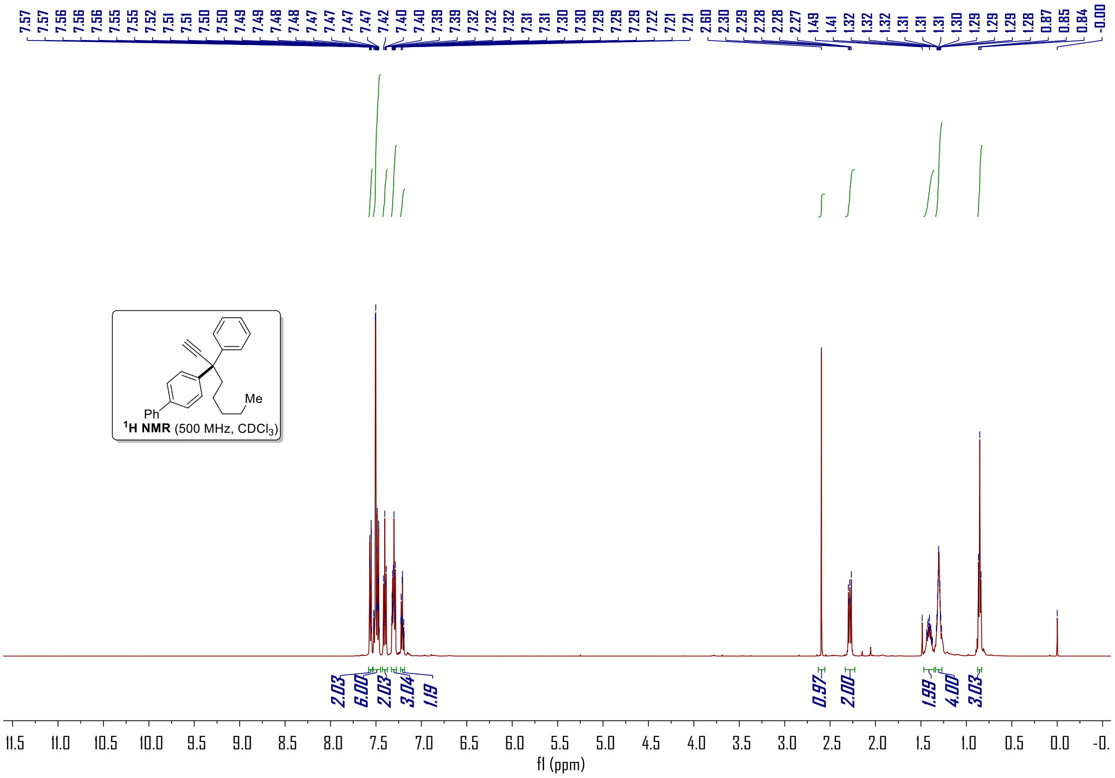

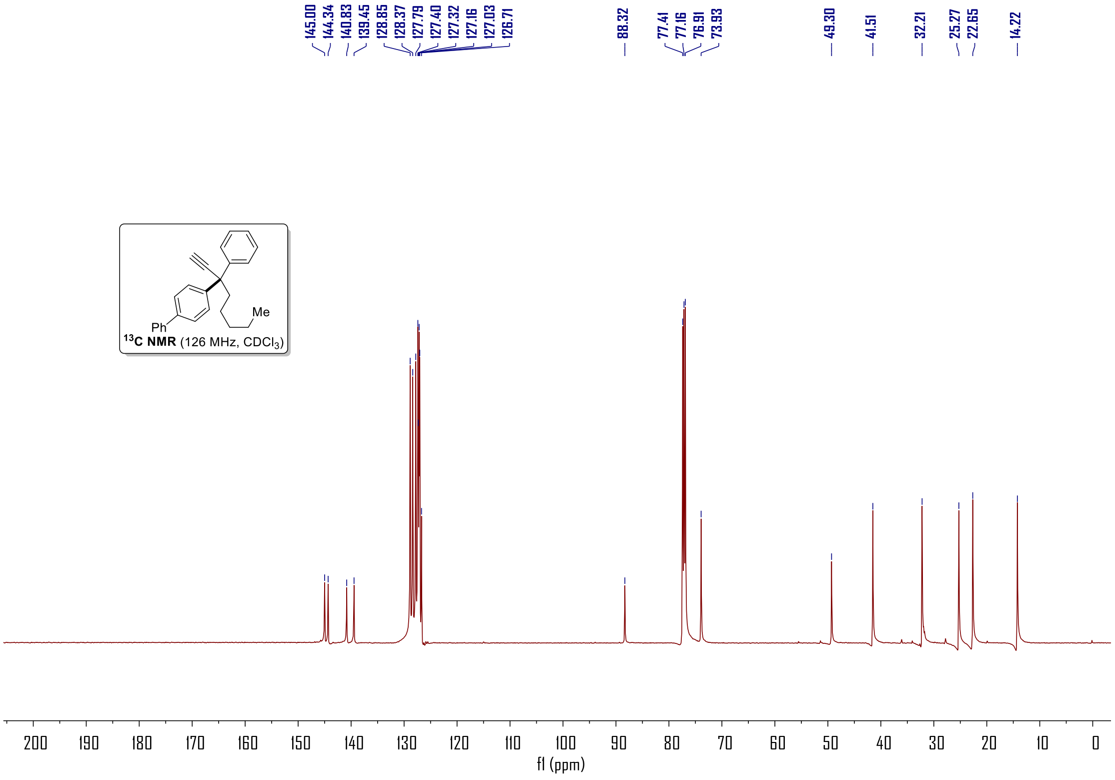

4-(5-Methyl-3-phenylhex-1-yn-3-yl)biphenyl (4ad)

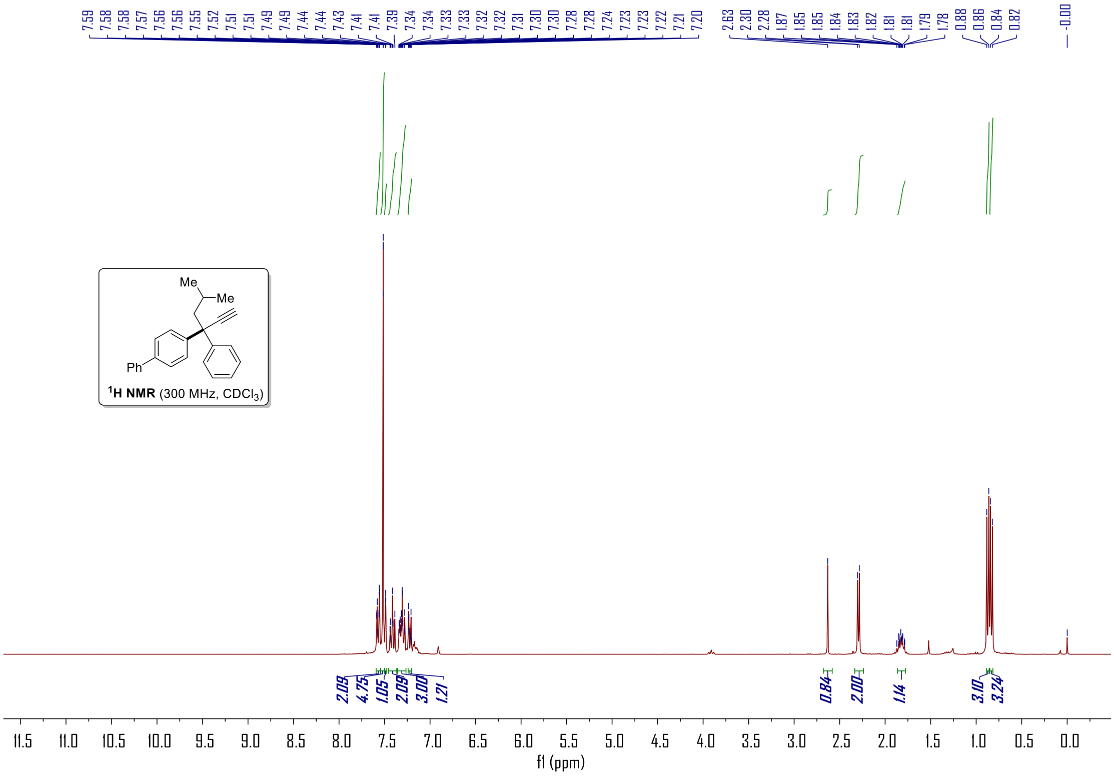

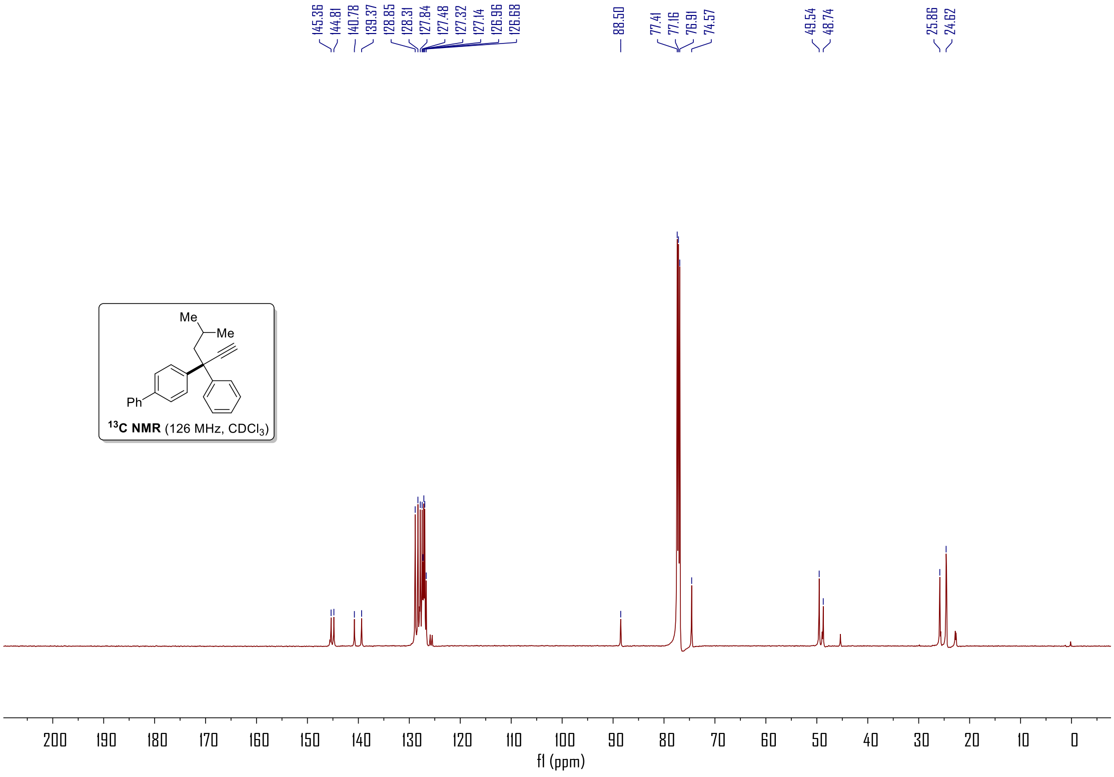

4-(4-Methyl-3-phenylpent-1-yn-3-yl)biphenyl (4ae)

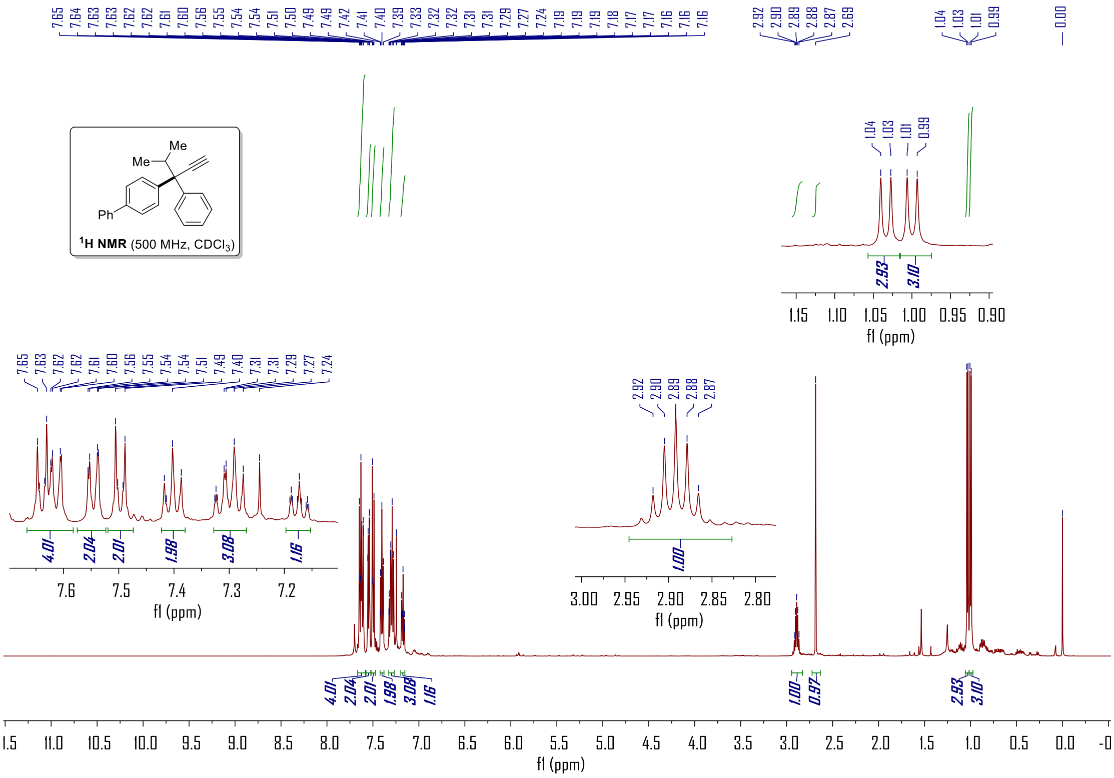

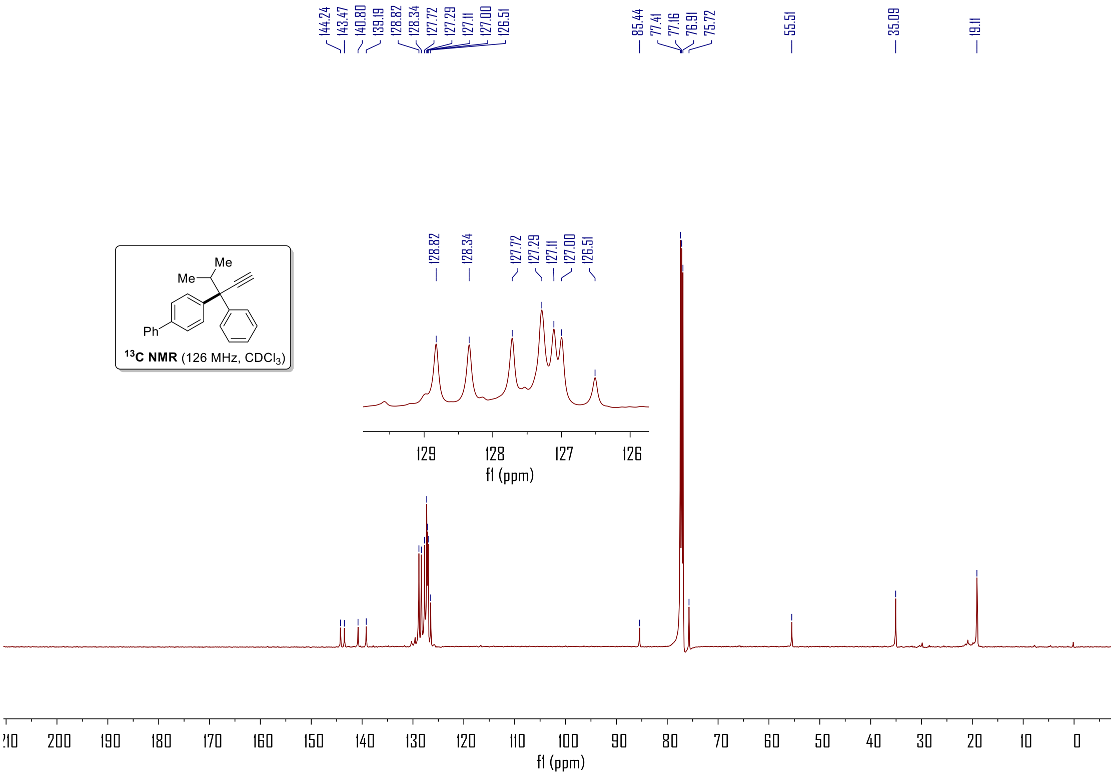

4-(3-(2-(Trifluoromethoxy)phenyl)pent-1-yn-3-yl)biphenyl (4af)

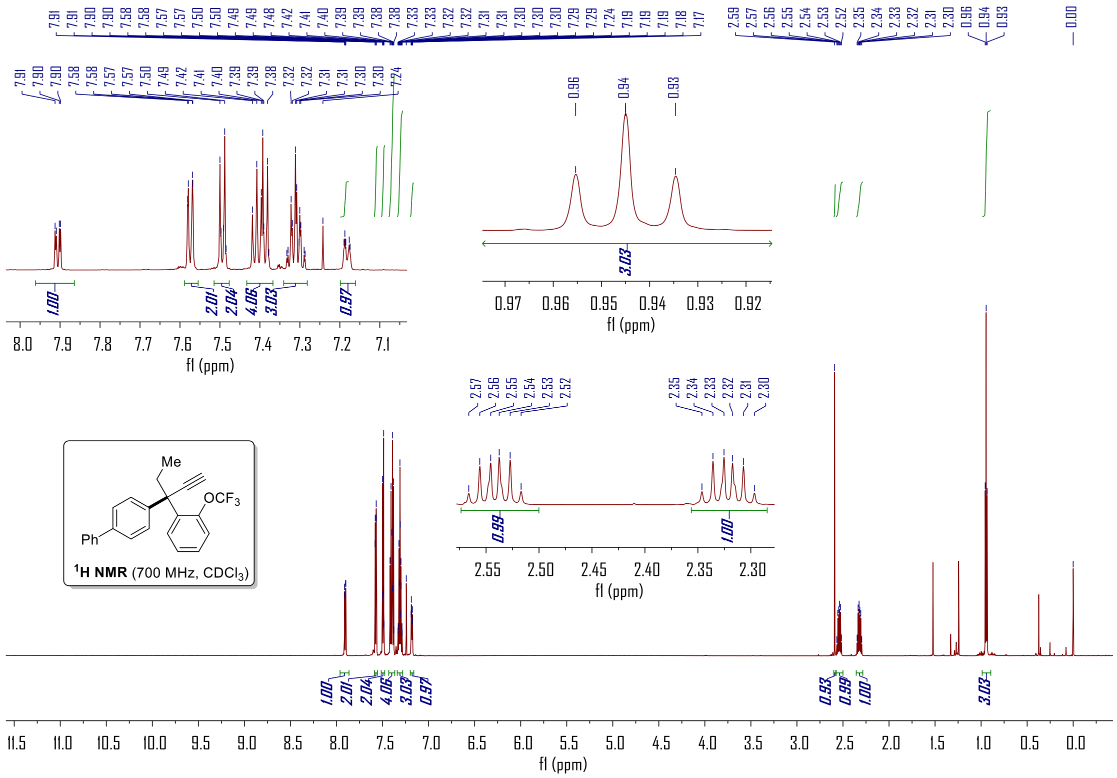

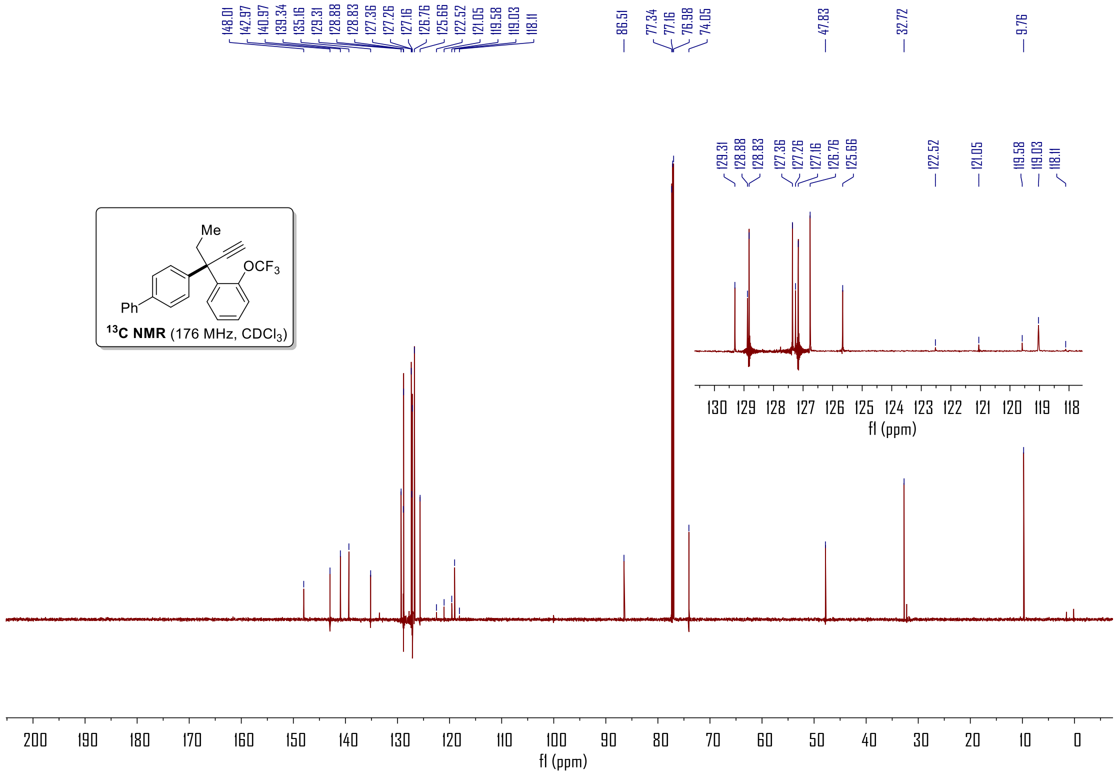

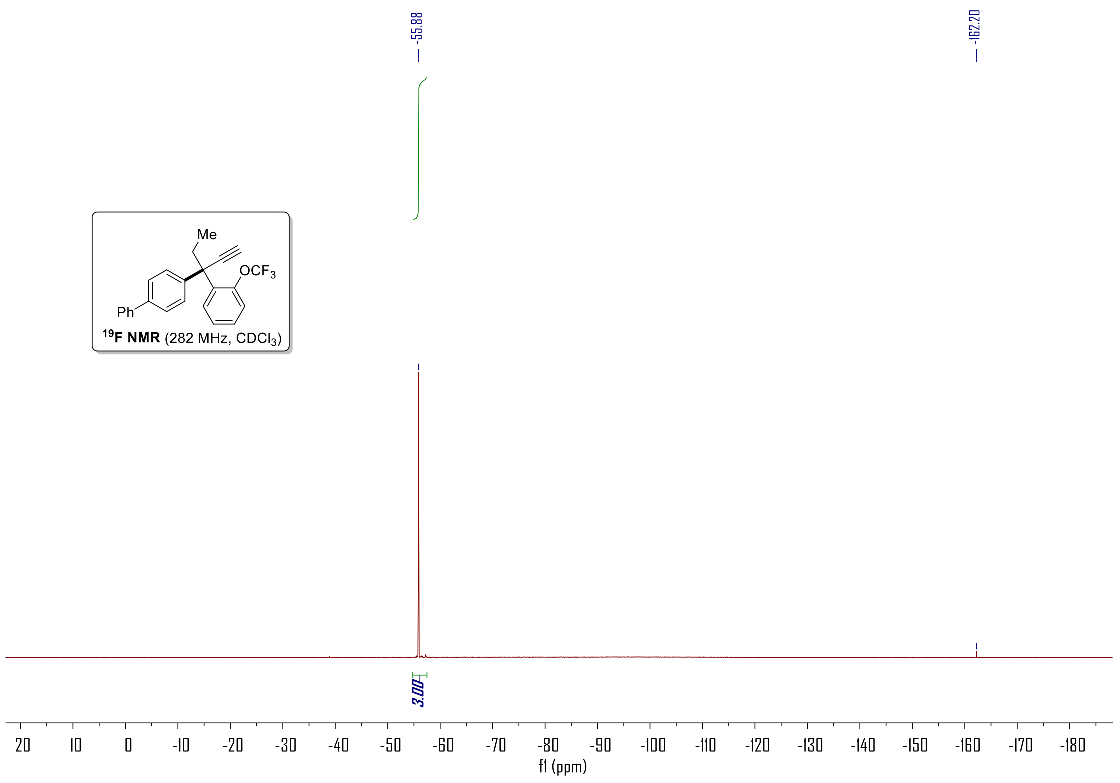

4-(3-(4-Methoxyphenyl)pent-1-yn-3-yl)biphenyl (4ag)

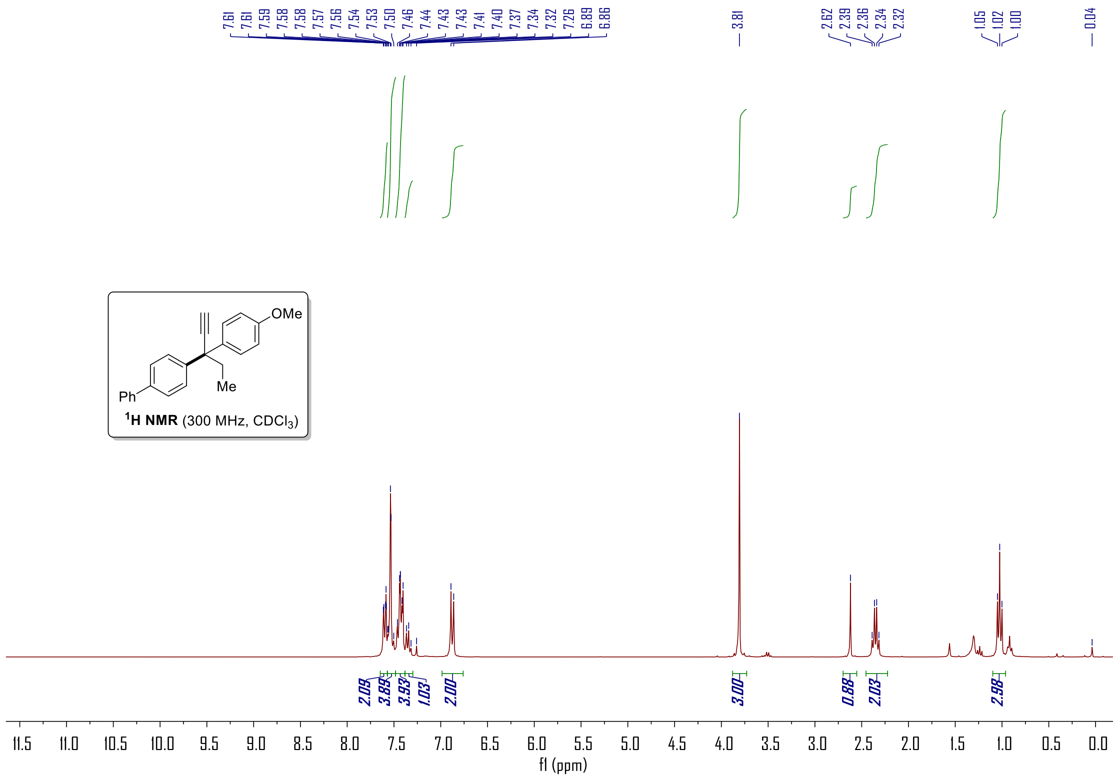

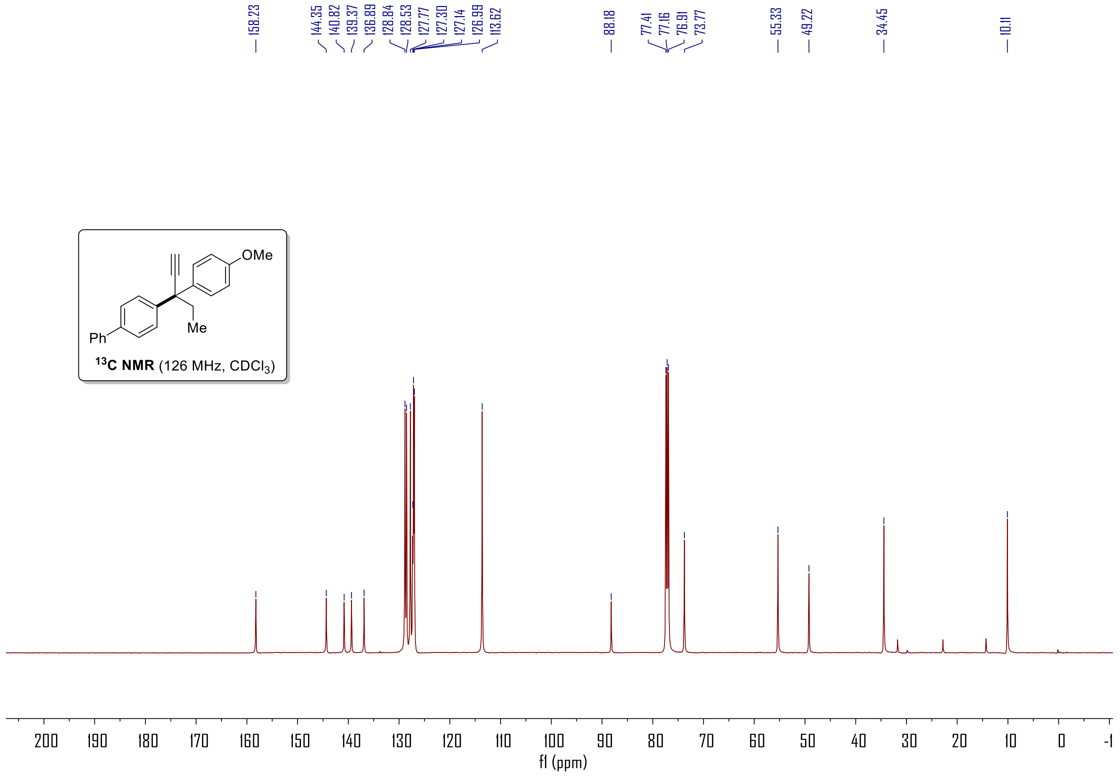

4-(3-(3-Methoxyphenyl)pent-1-yn-3-yl)biphenyl (4ah)

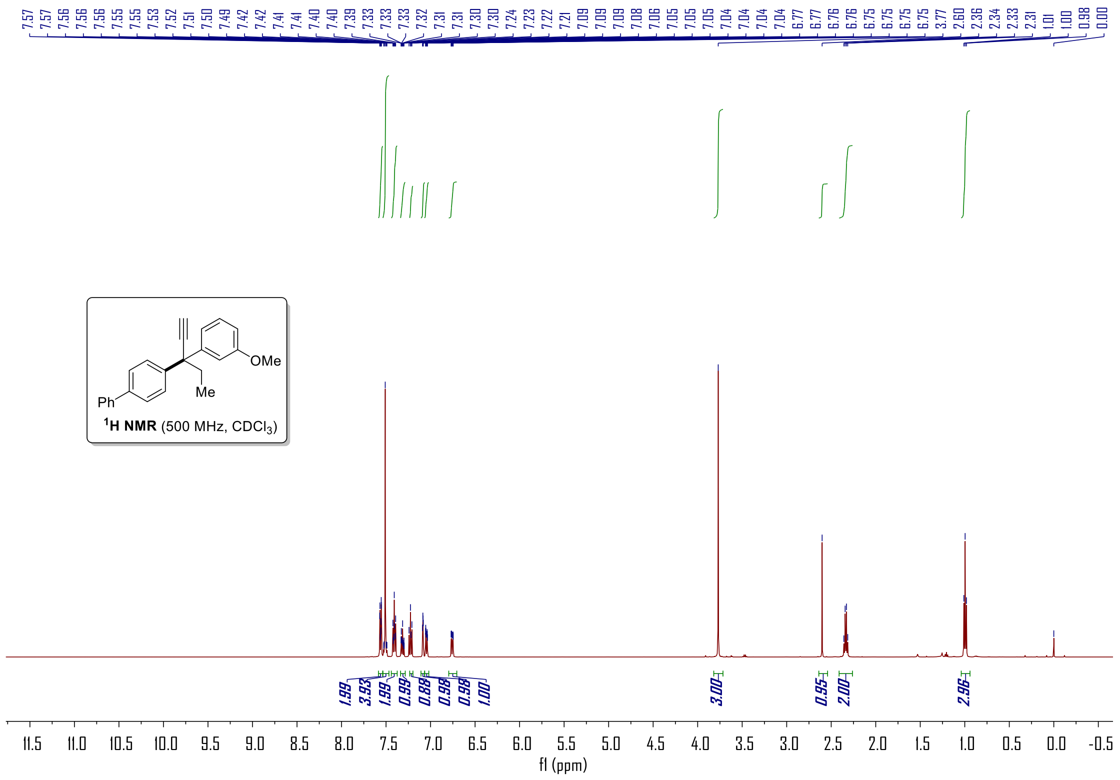

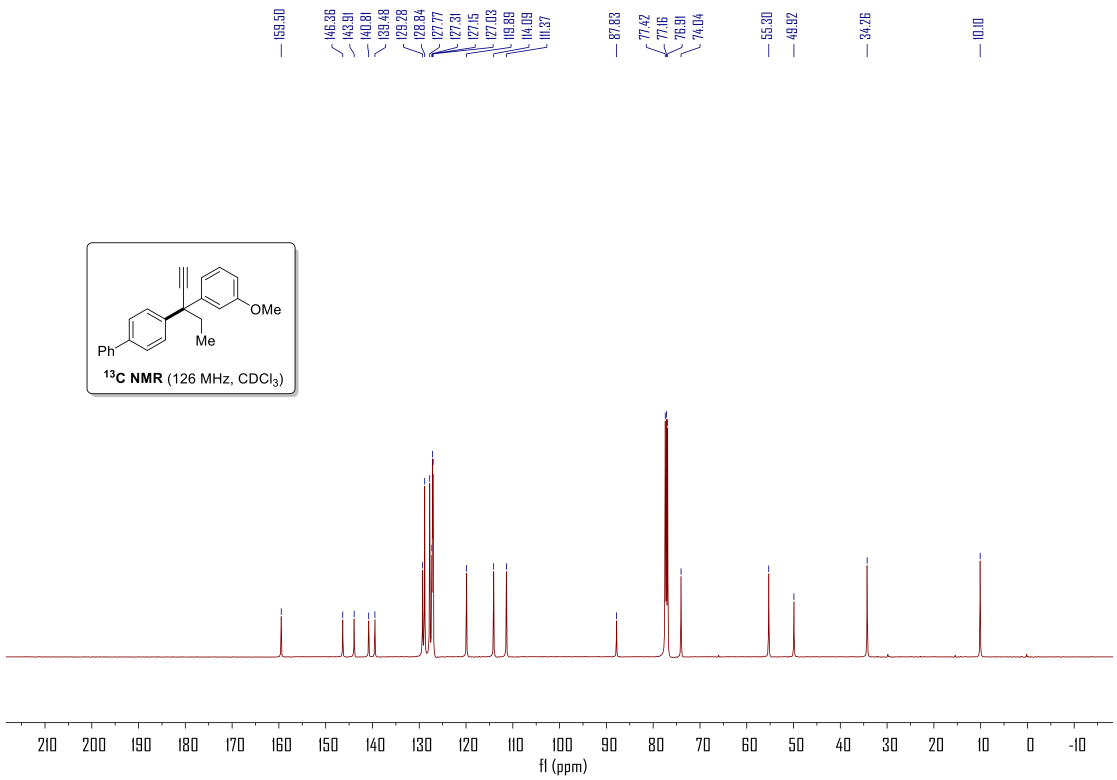

4-(3-(*p*-Tolyl)pent-1-yn-3-yl)biphenyl (4ai)

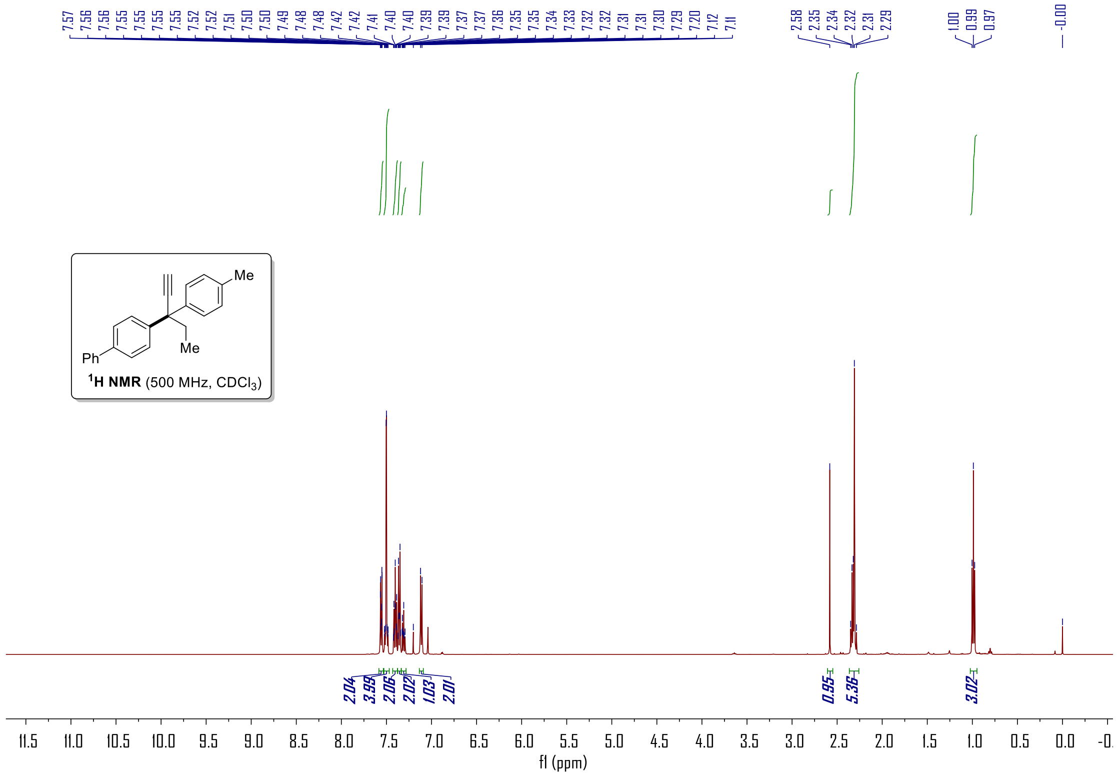

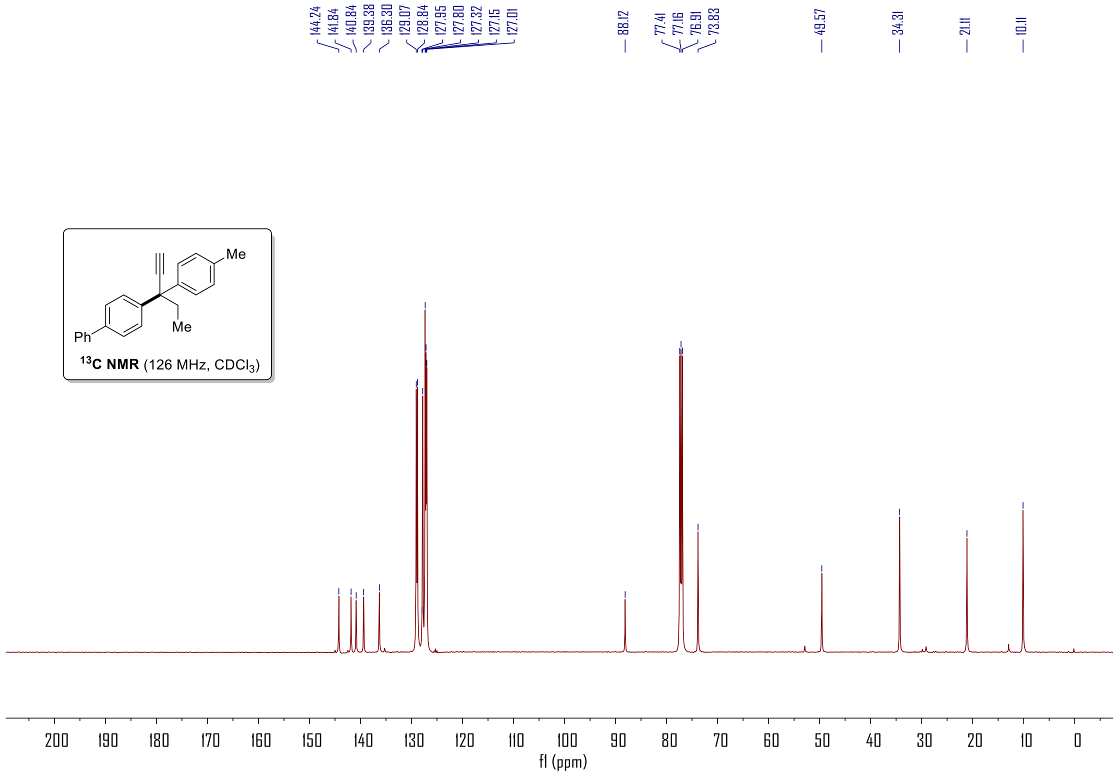

4-(3-(*m*-Tolyl)pent-1-yn-3-yl)biphenyl (4aj)

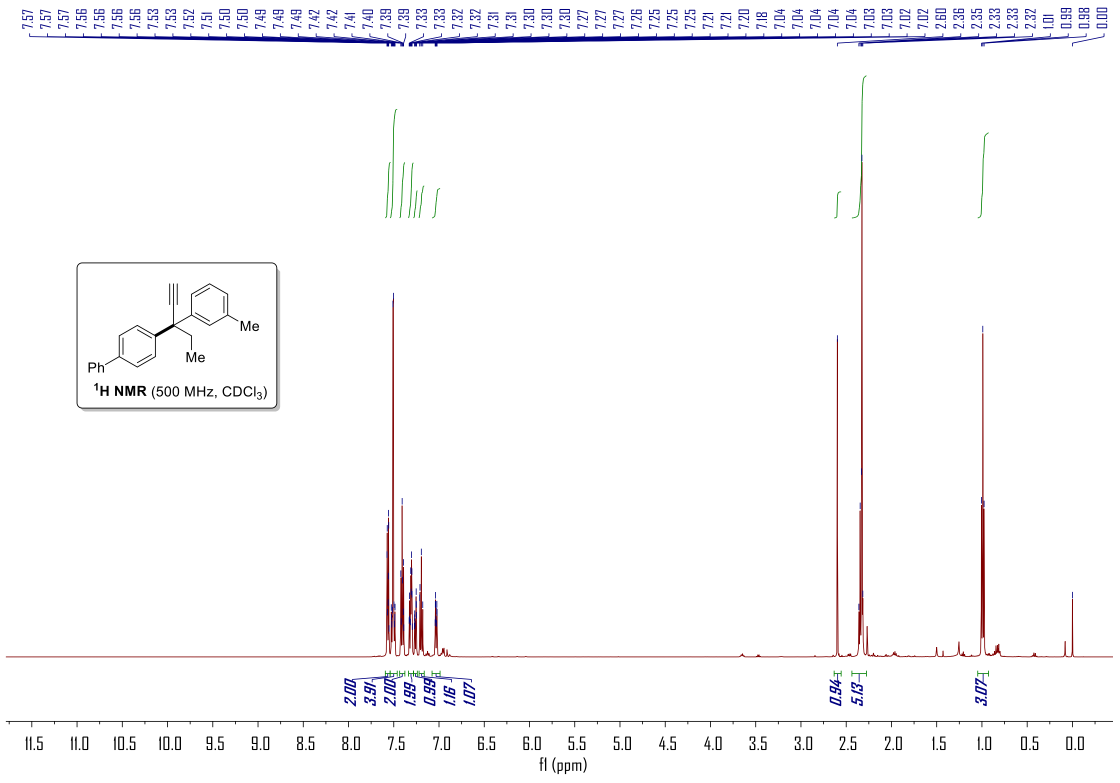

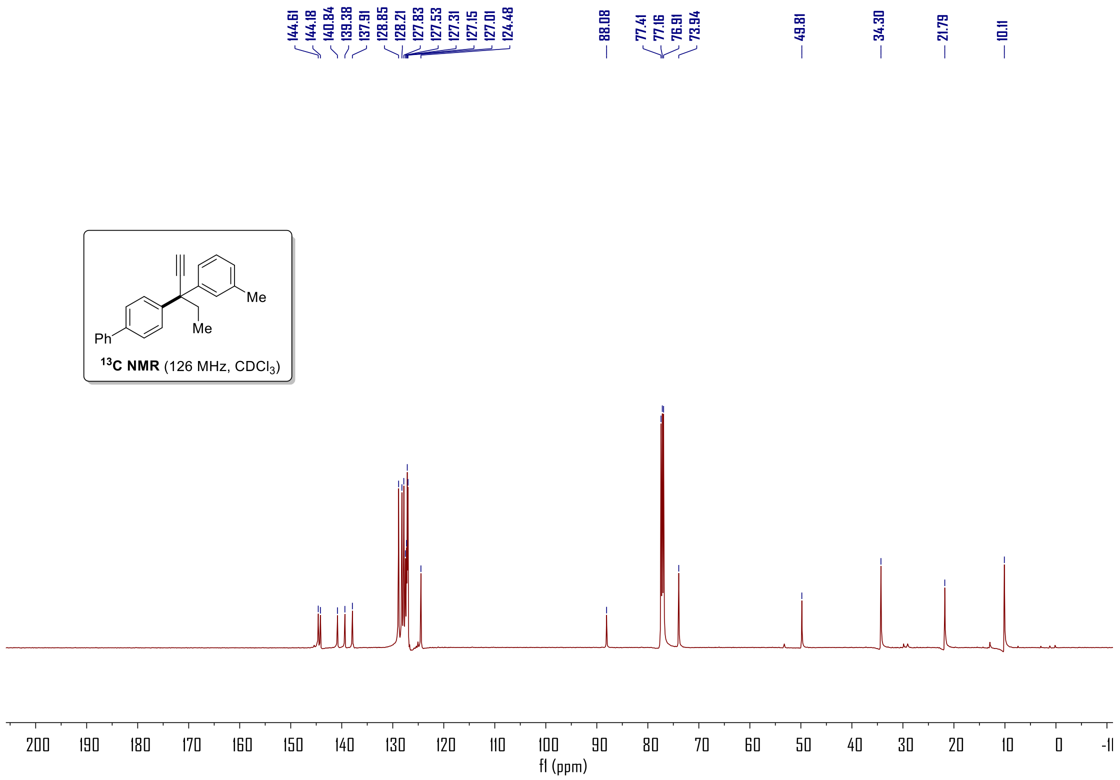

4-(3-(3,5-Dimethylphenyl)pent-1-yn-3-yl) biphenyl (4ak)

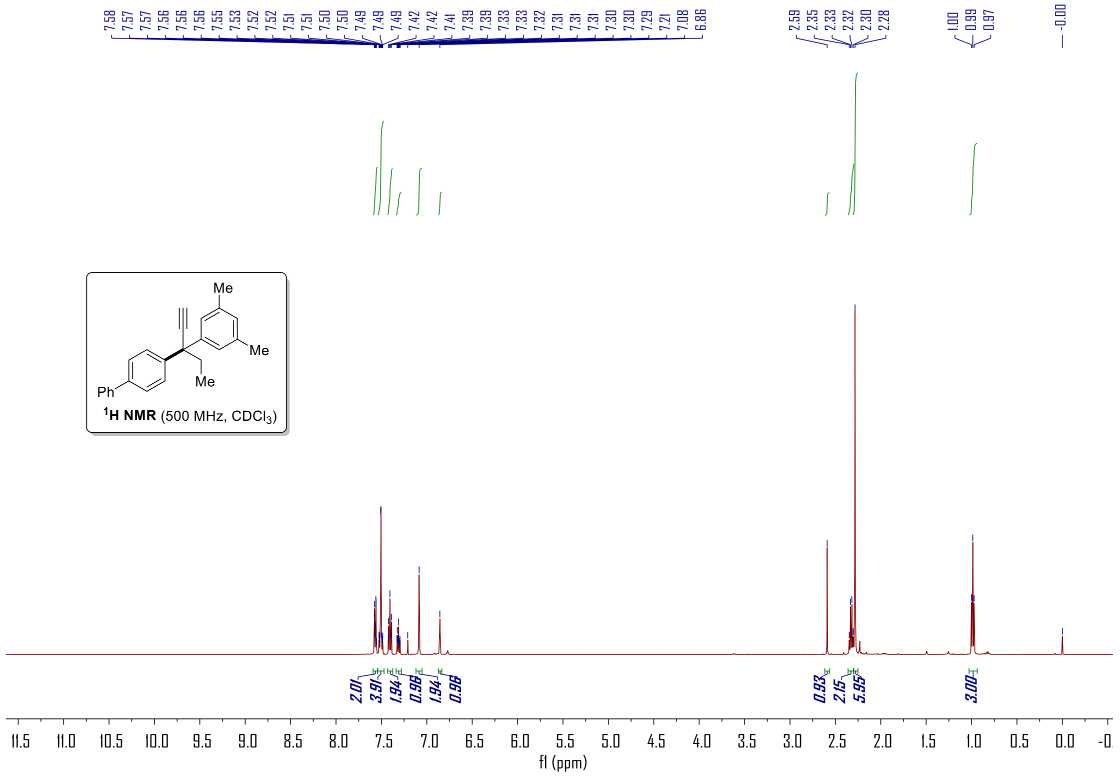

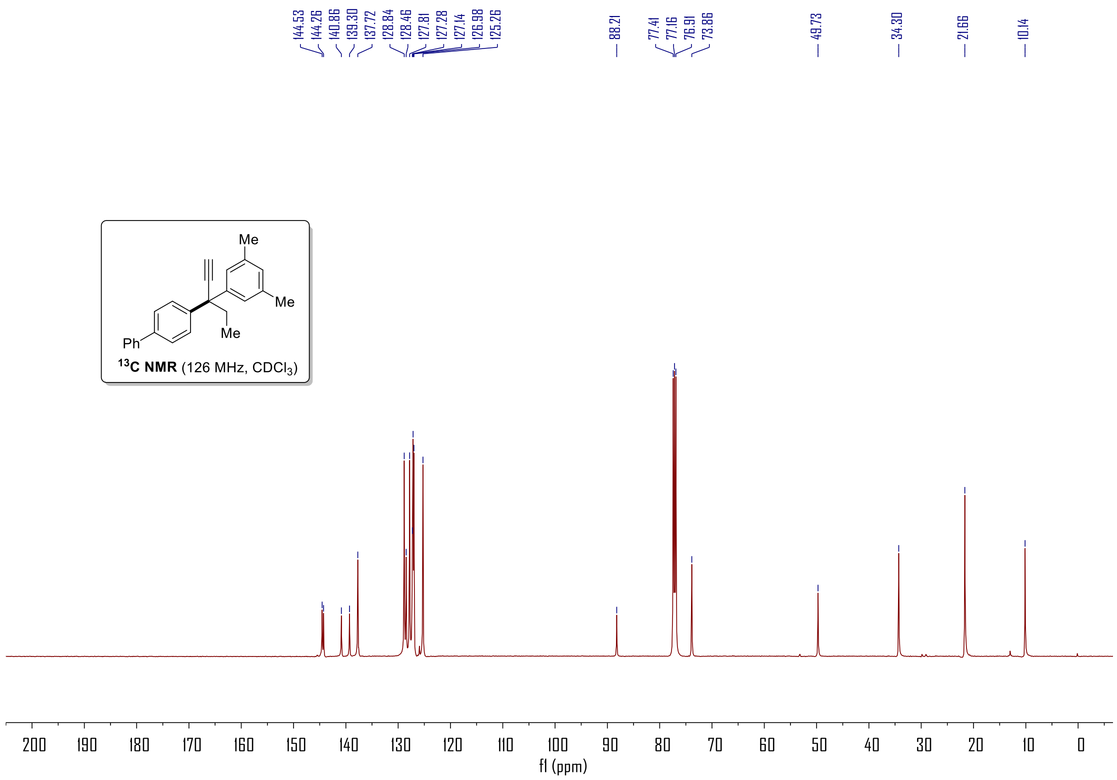

4-(3-(4-Chlorophenyl)pent-1-yn-3-yl)biphenyl (4al)

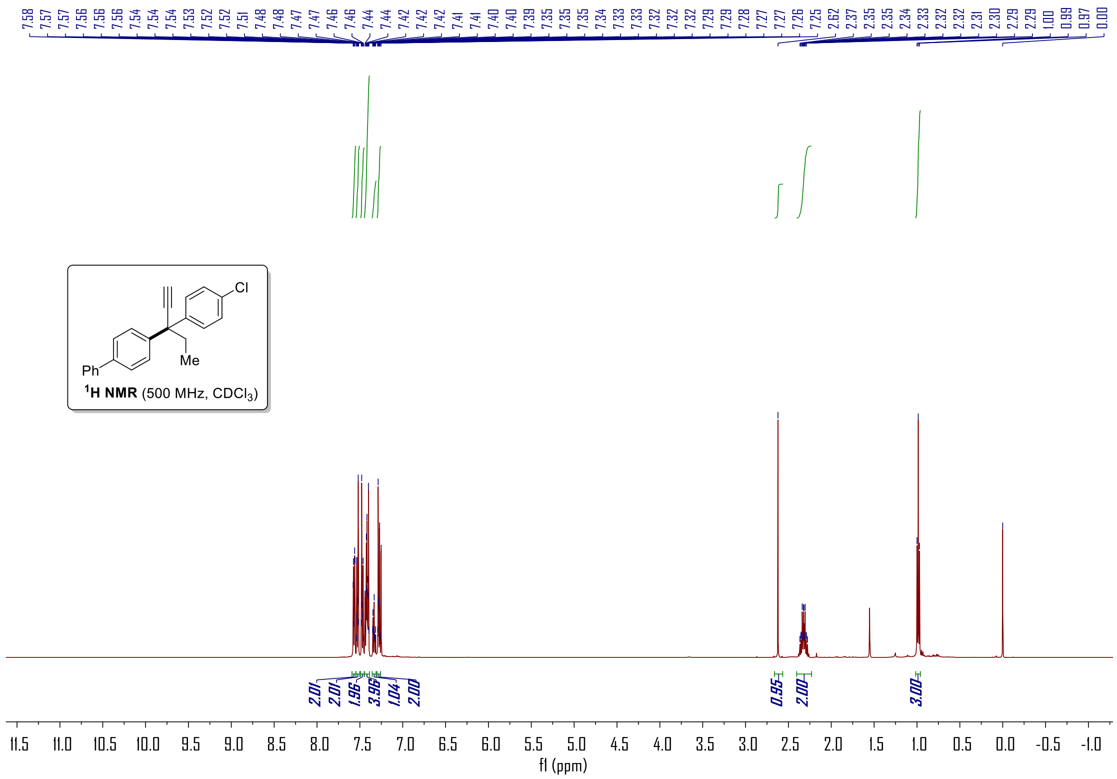

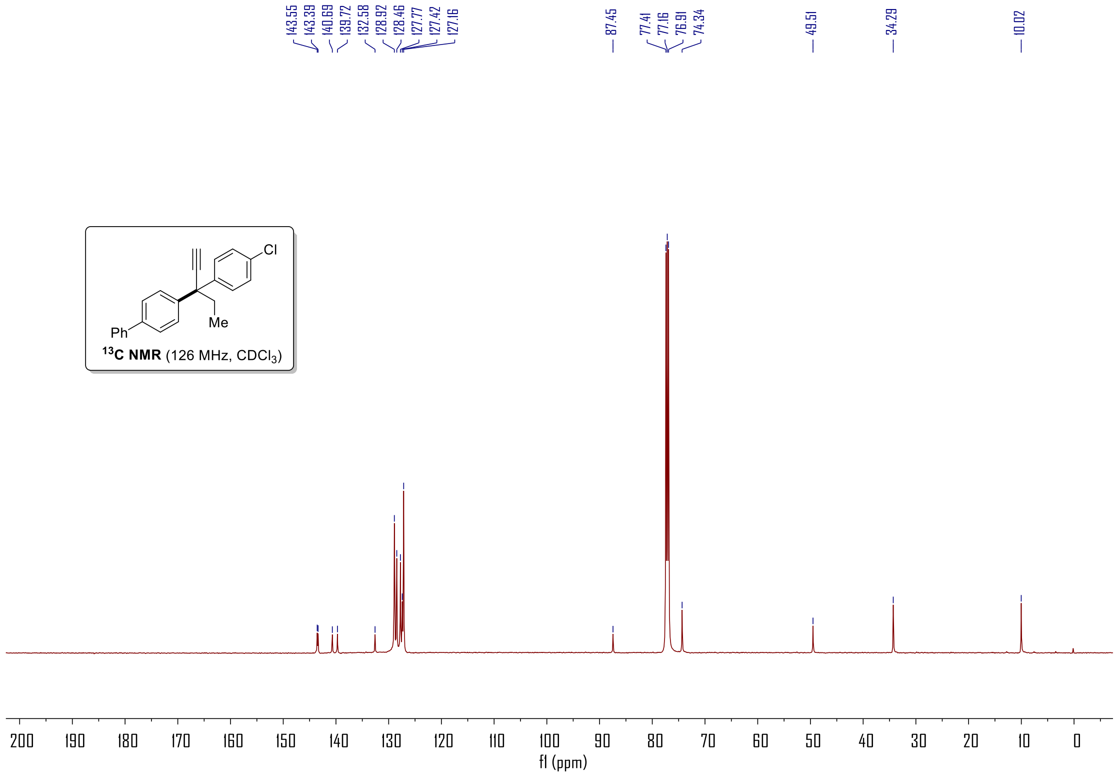

2-(3-(Biphenyl-4-yl)pent-1-yn-3-yl)naphthalene (4am)

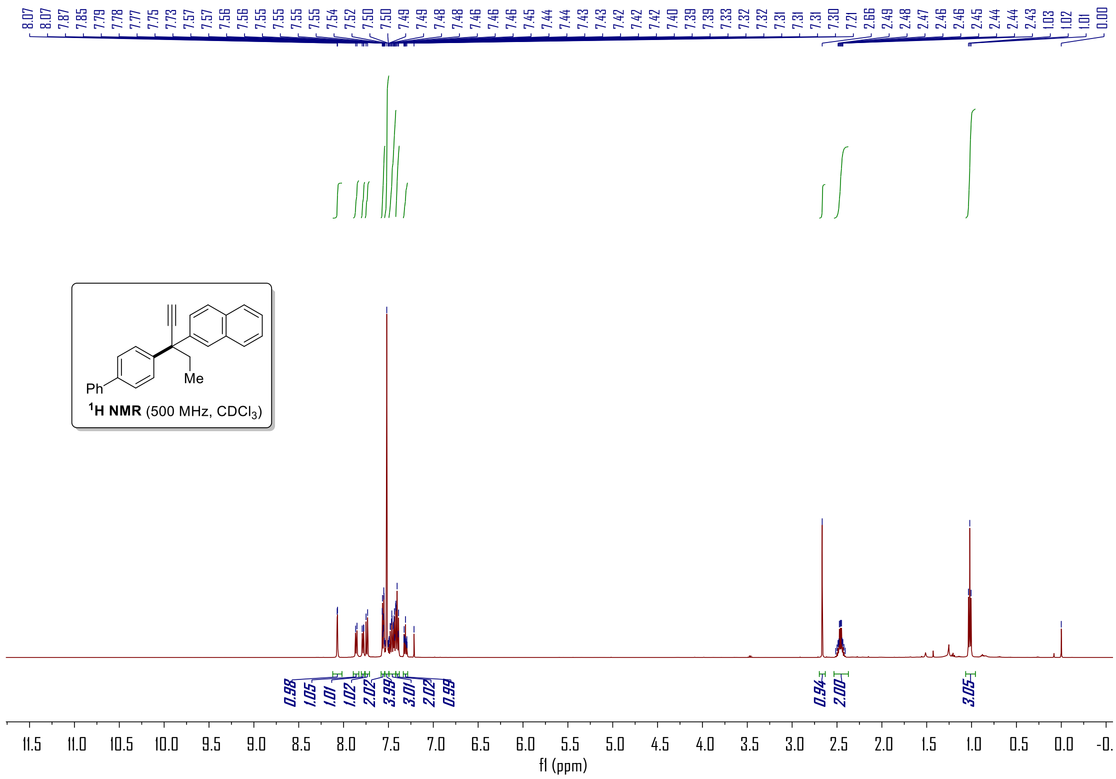

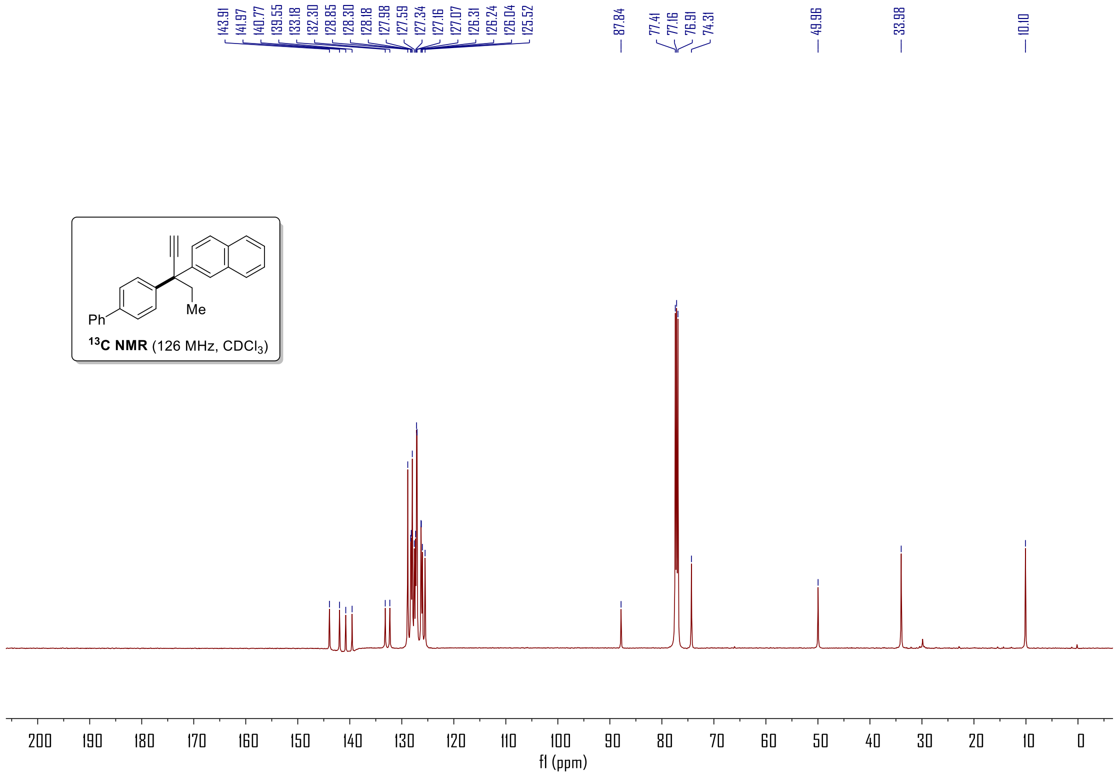

(3-(Biphenyl-4-yl)-3-phenylpent-1-yn-1-yl)triethylsilane (4an)

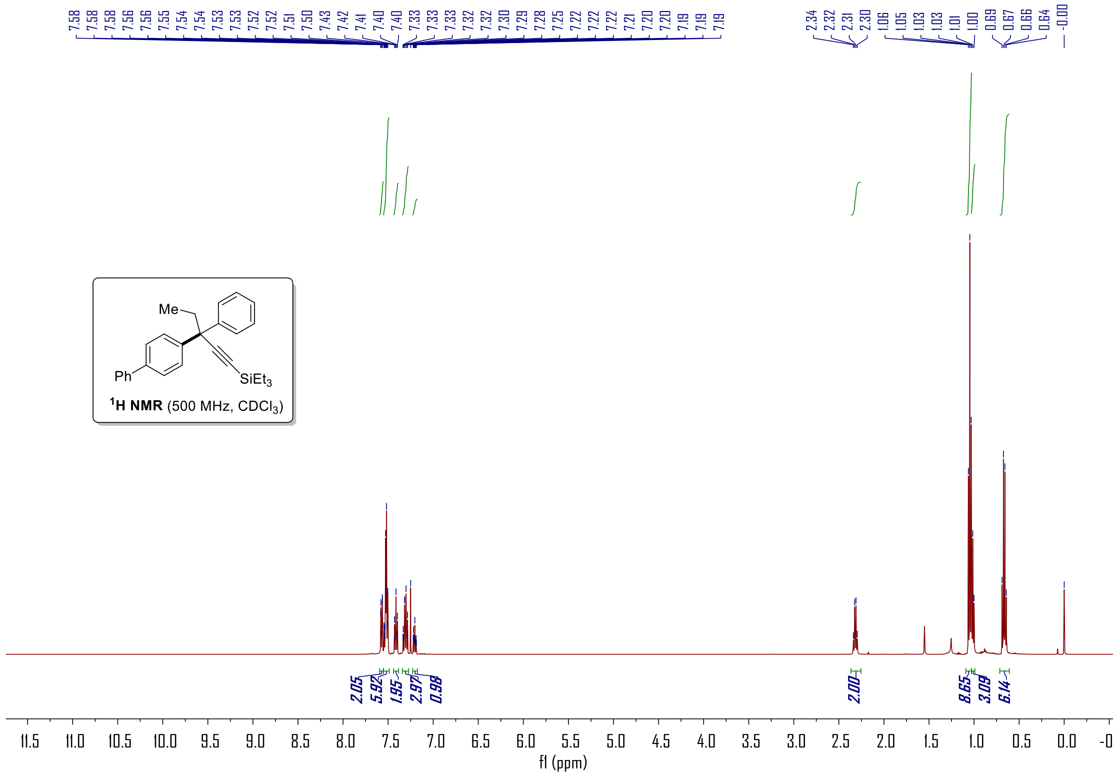

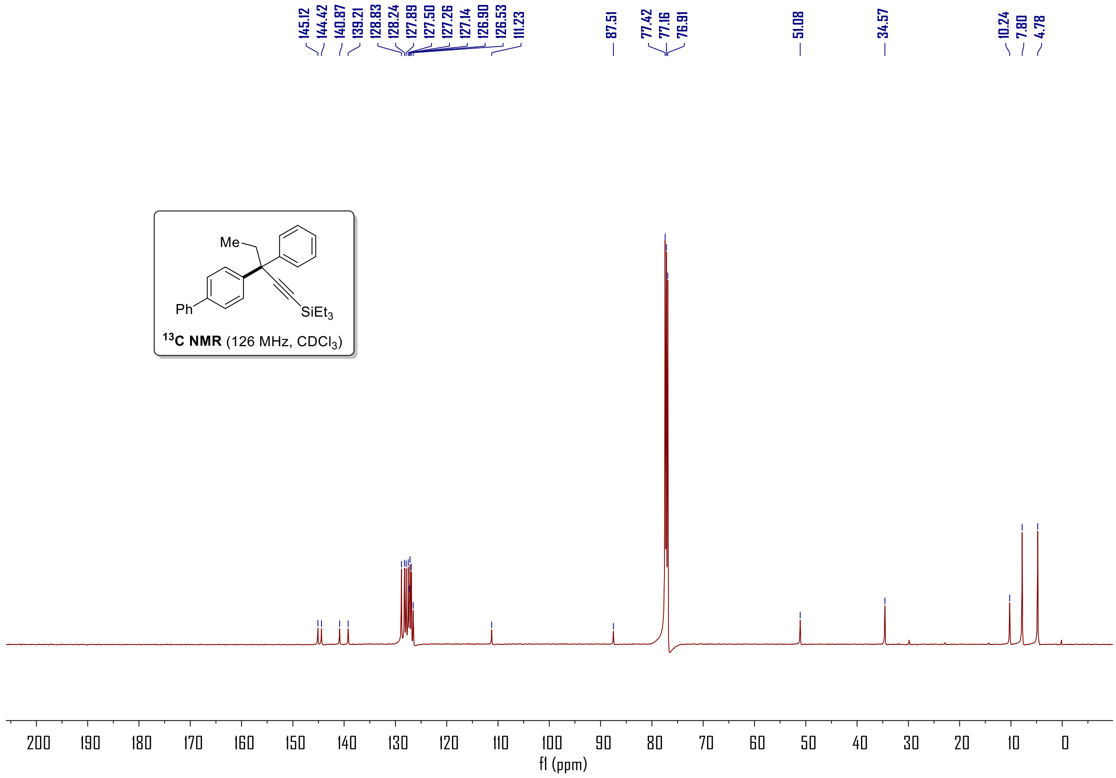

3-(3-(4-Methoxyphenyl)pent-1-yn-3-yl)biphenyl (4bg)

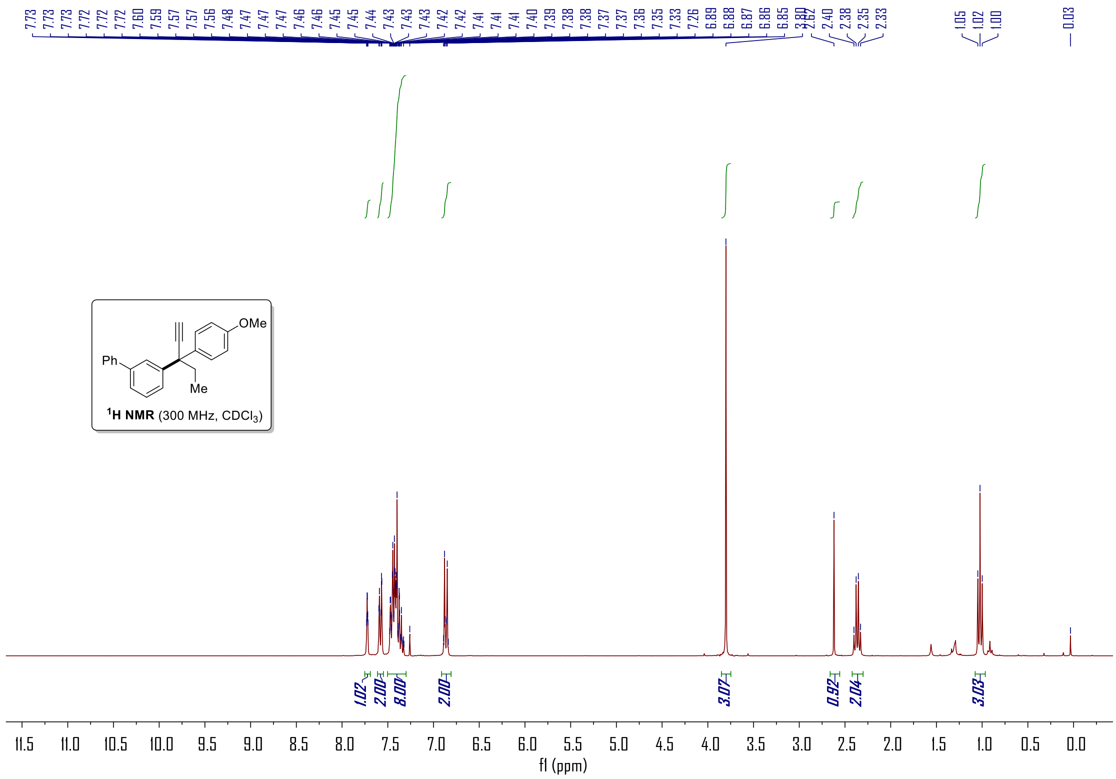

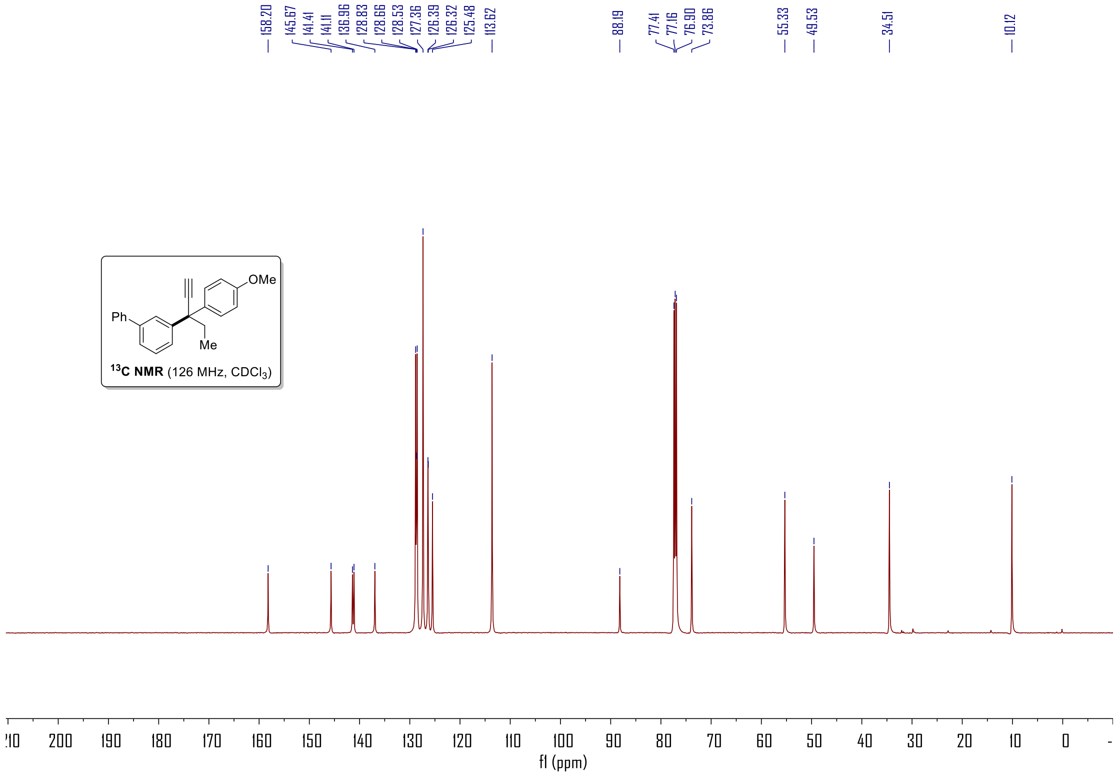

1-(3-(4-Methoxyphenyl)pent-1-yn-3-yl)-3-phenoxybenzene (4dg)

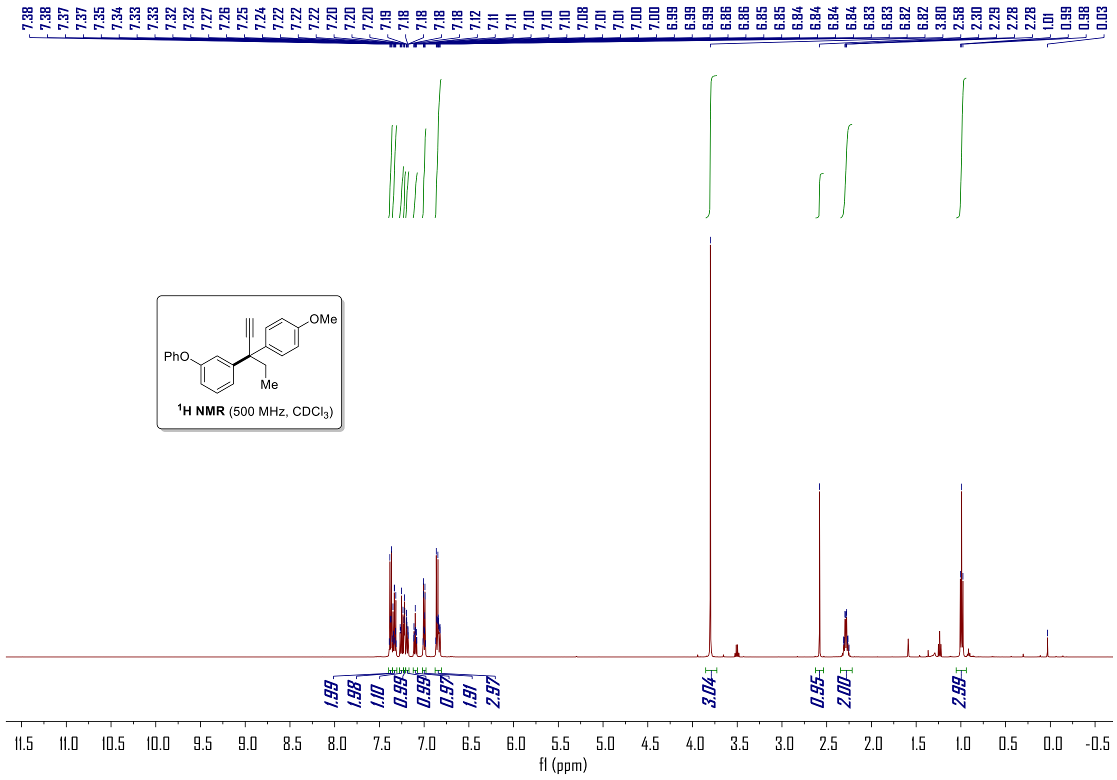

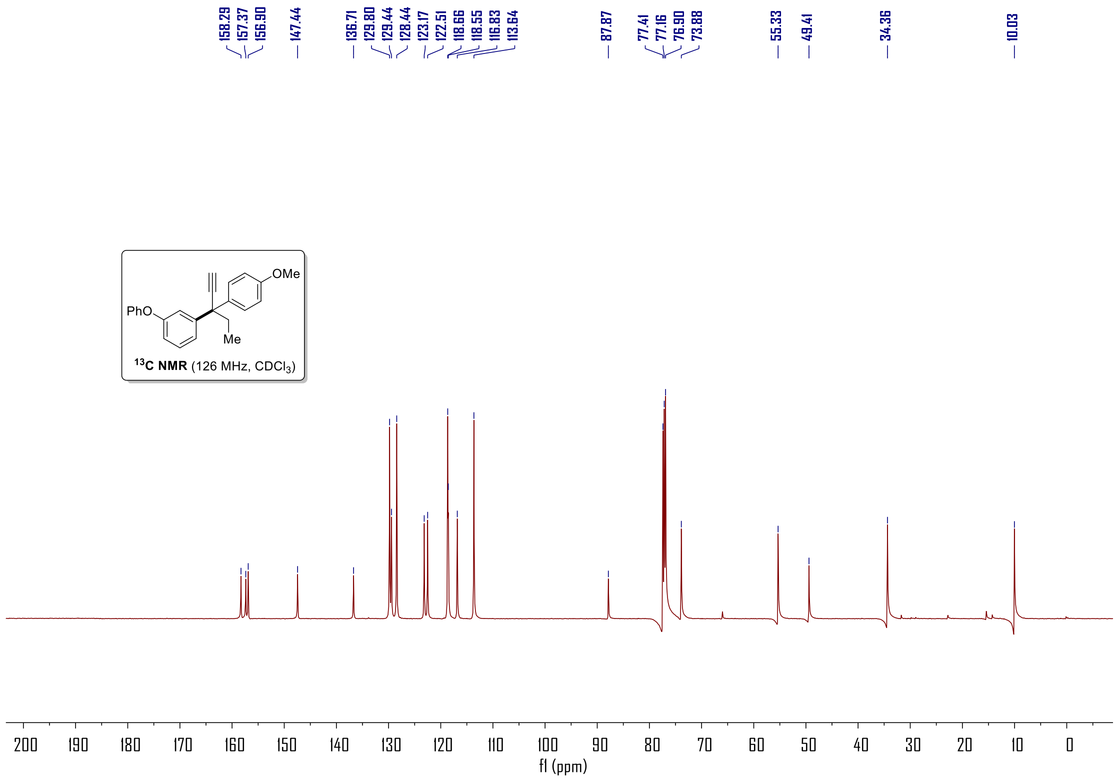

1-Phenoxy-3-(3-(p-tolyl)pent-1-yn-3-yl)benzene (4di)

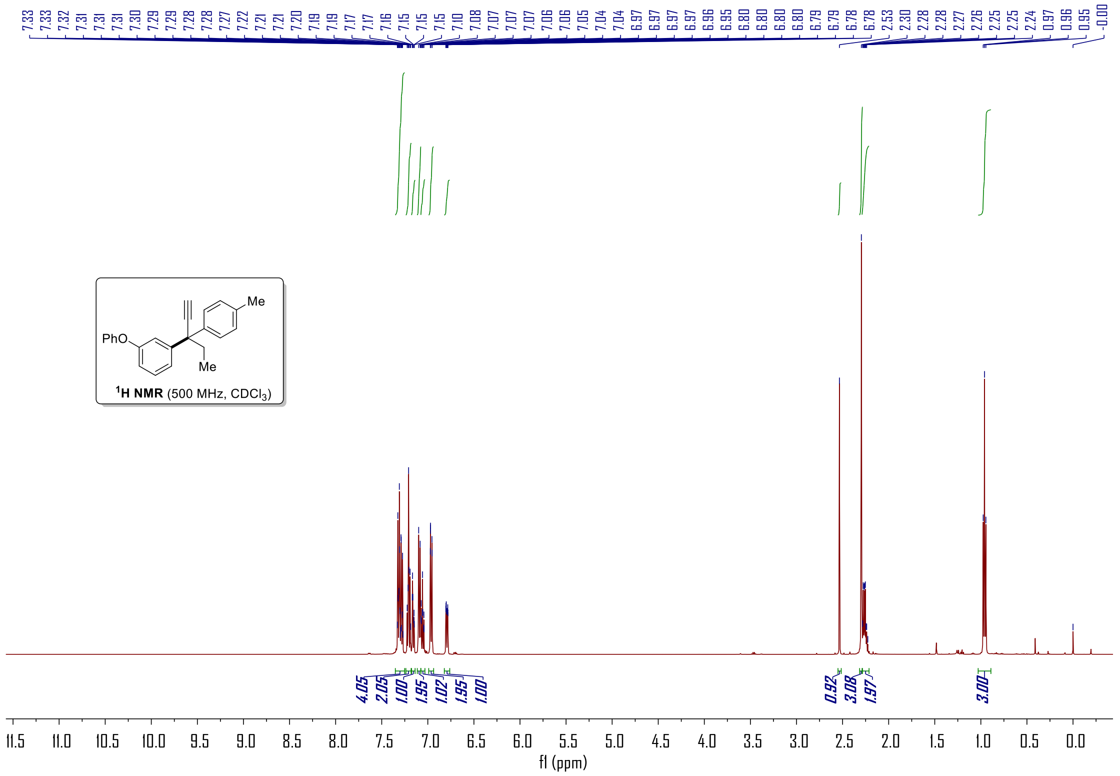

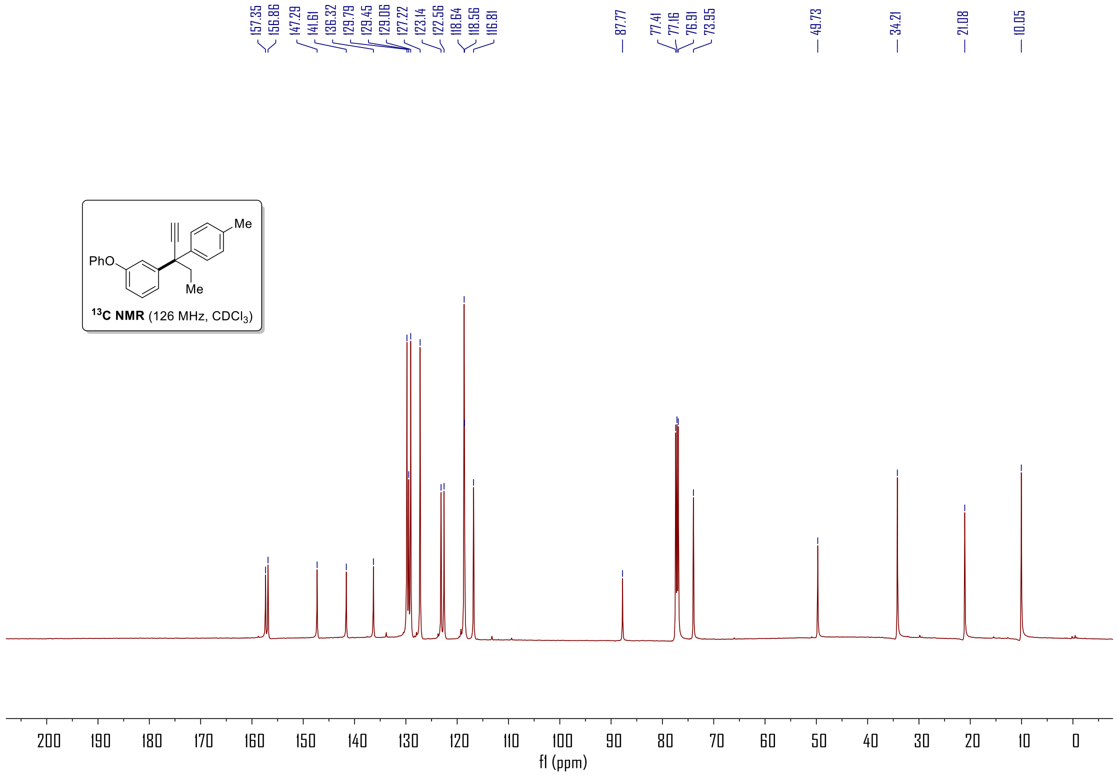

1,3-Dimethyl-5-(3-(4-phenoxyphenyl)pent-1-yn-3-yl)benzene (4ek)

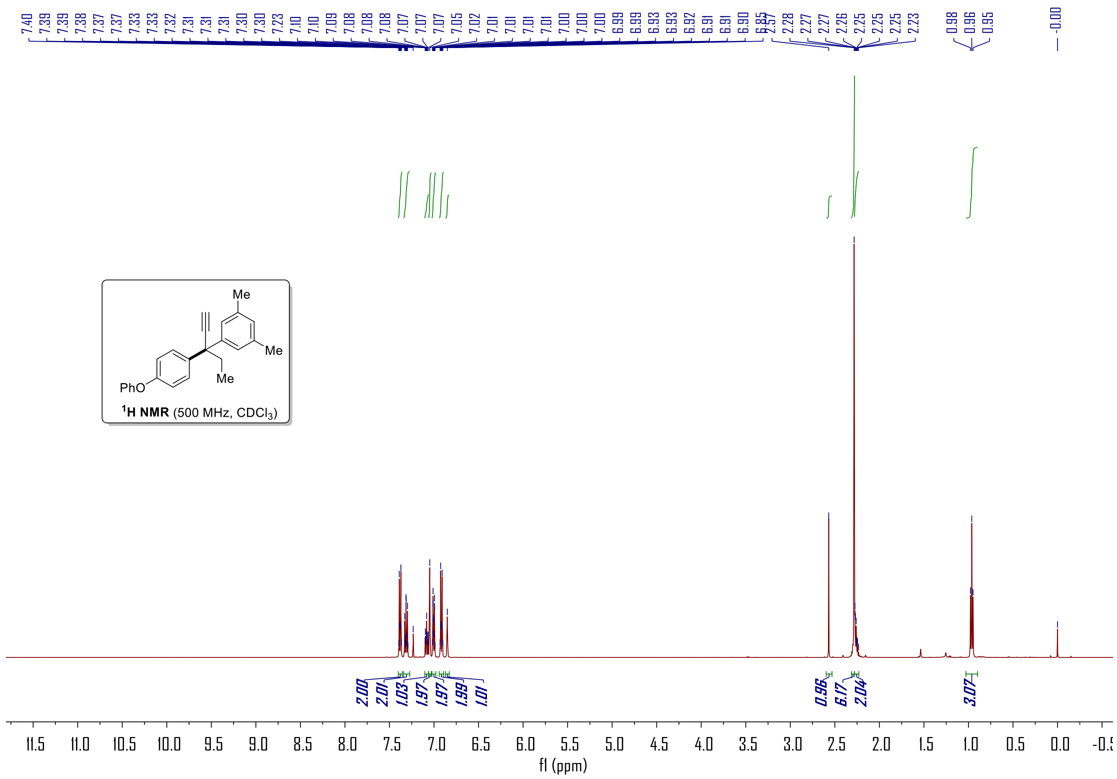

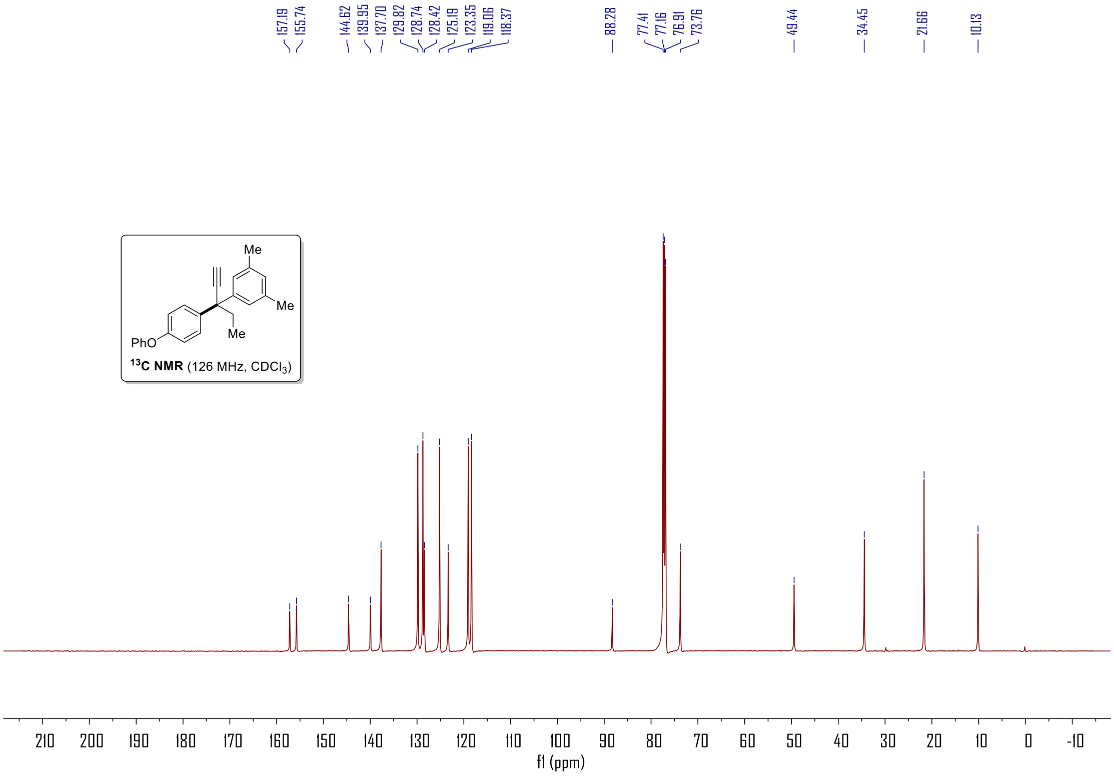

4-Methyl-4'-(3-phenyloct-1-yn-3-yl)biphenyl (4gc)

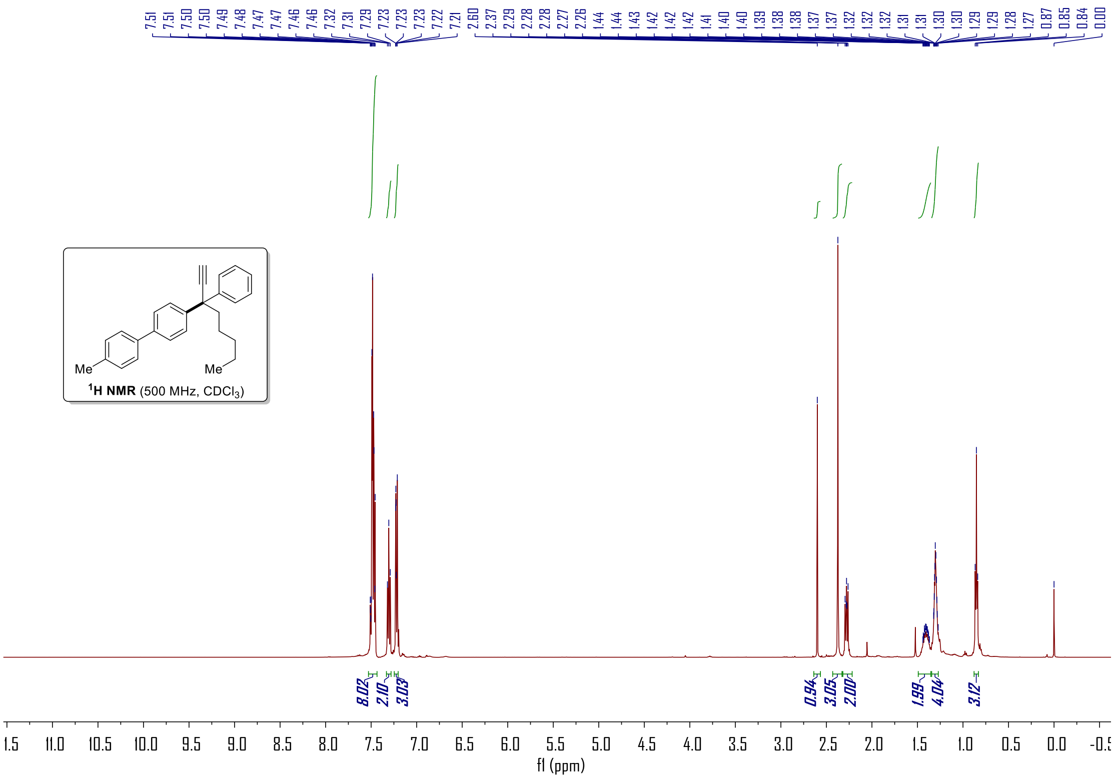

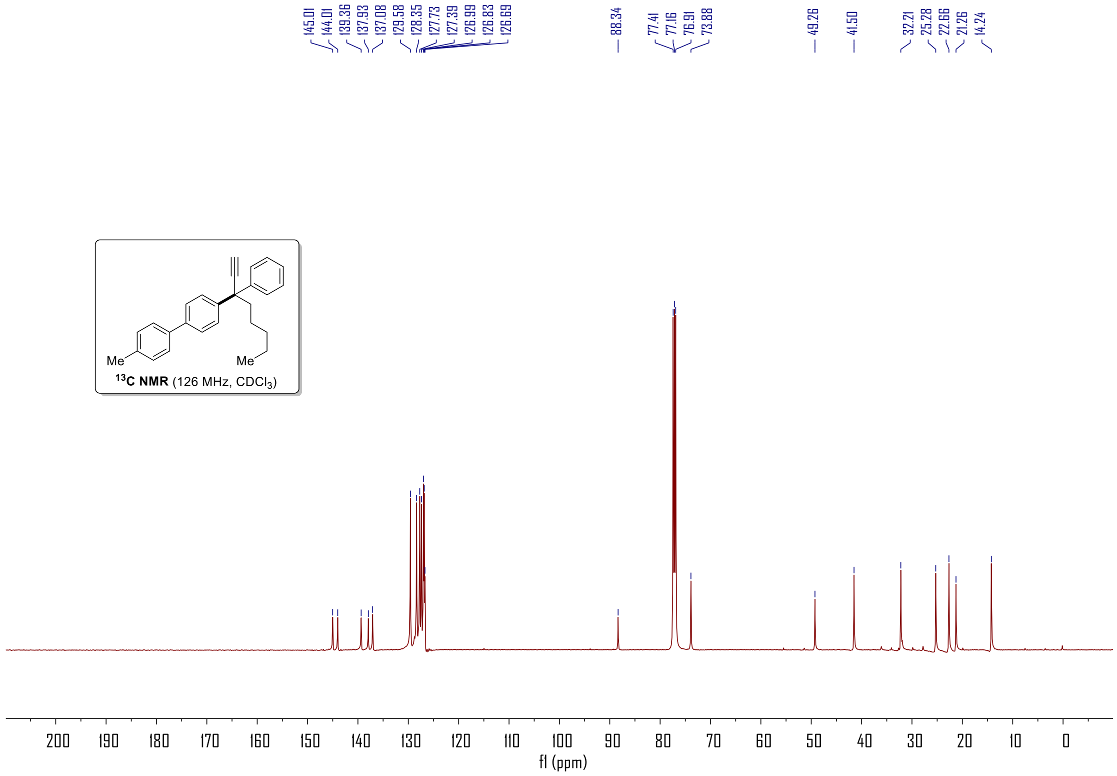

4-Methoxy-4'-(3-(4-methoxyphenyl)pent-1-yn-3-yl)biphenyl (4hg)

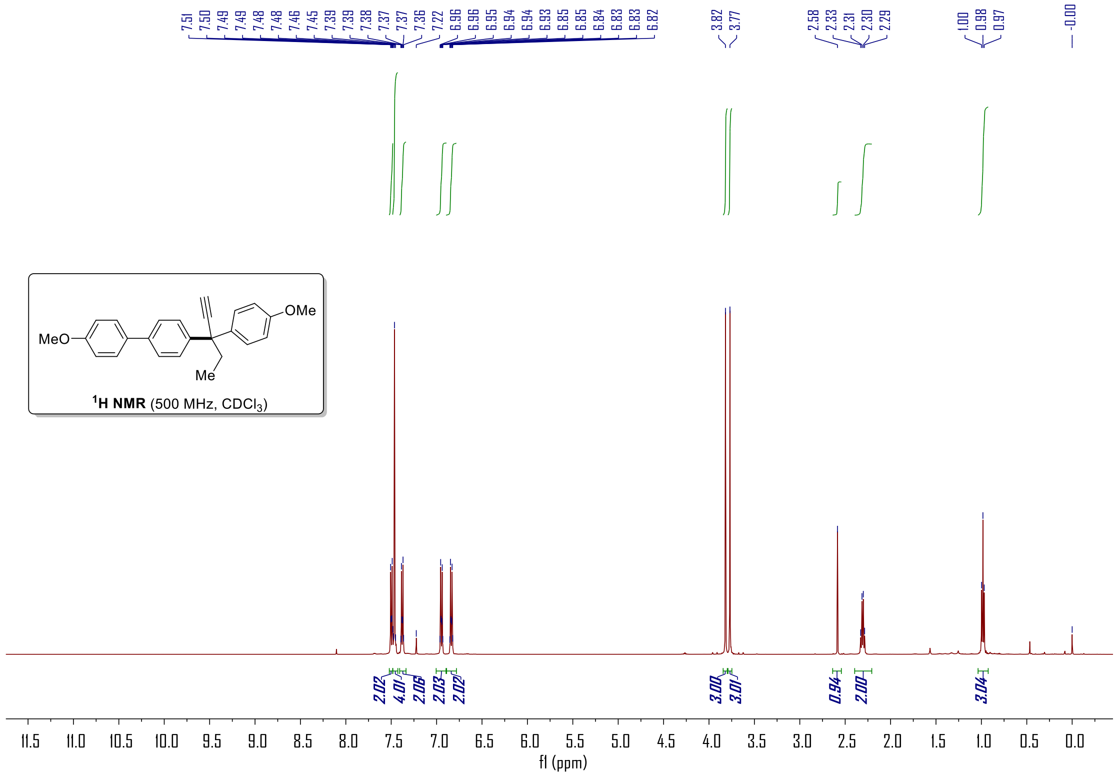

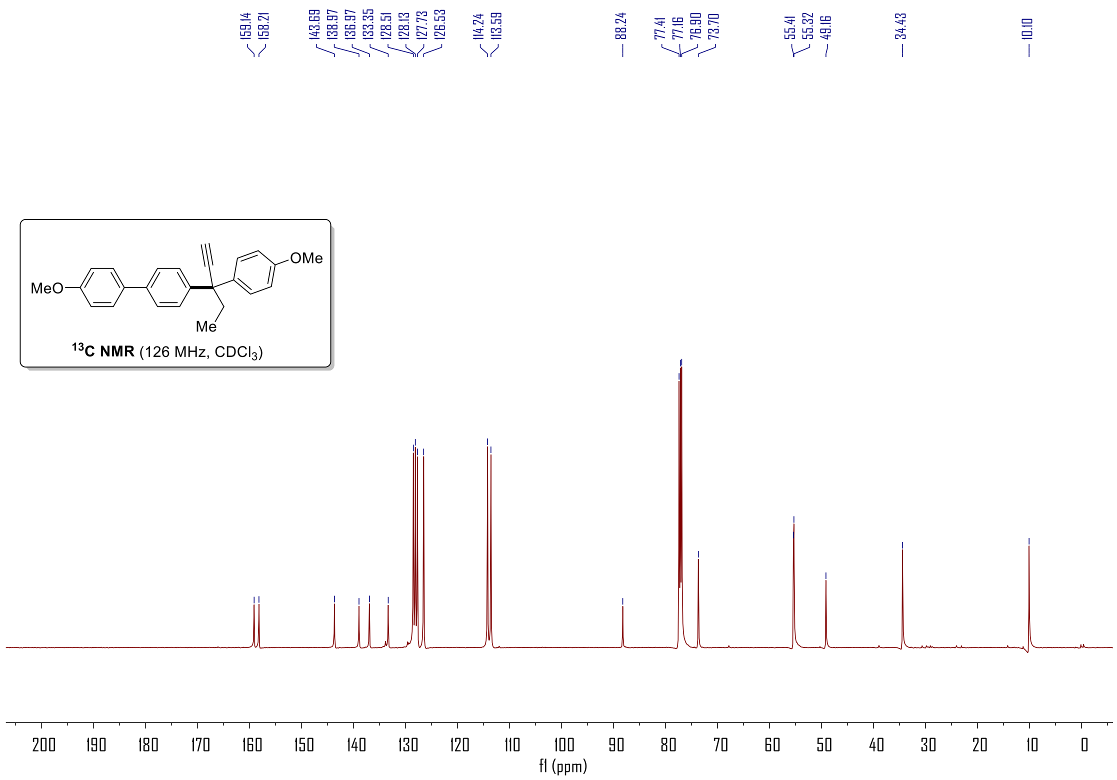

4-Methoxy-4'-(3-(3-methoxyphenyl)pent-1-yn-3-yl)biphenyl (4hh)

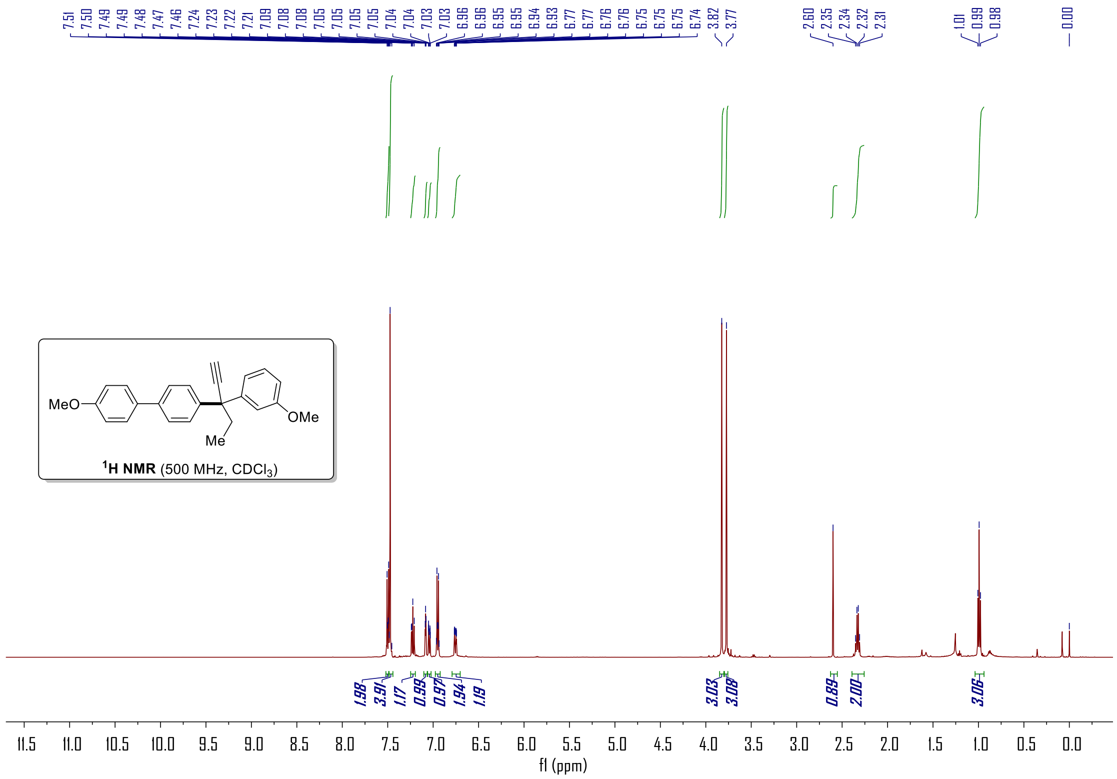

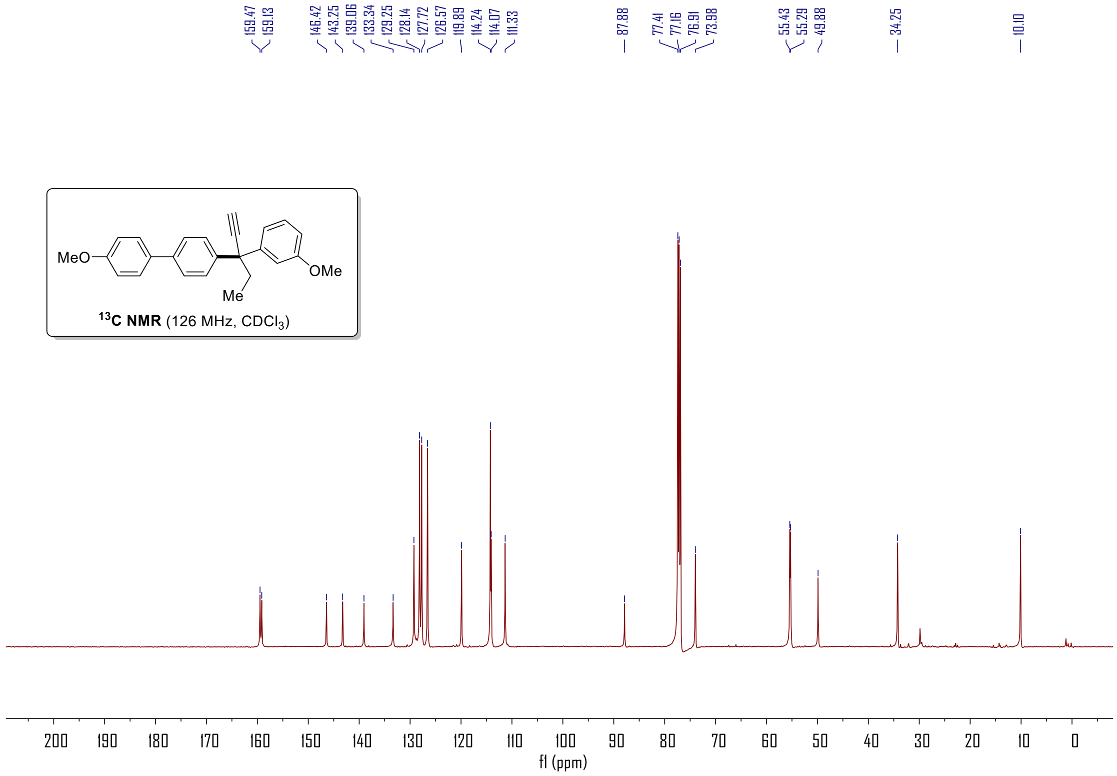

4-Chloro-4'-(3-(3-methoxyphenyl)pent-1-yn-3-yl)biphenyl (4kh)

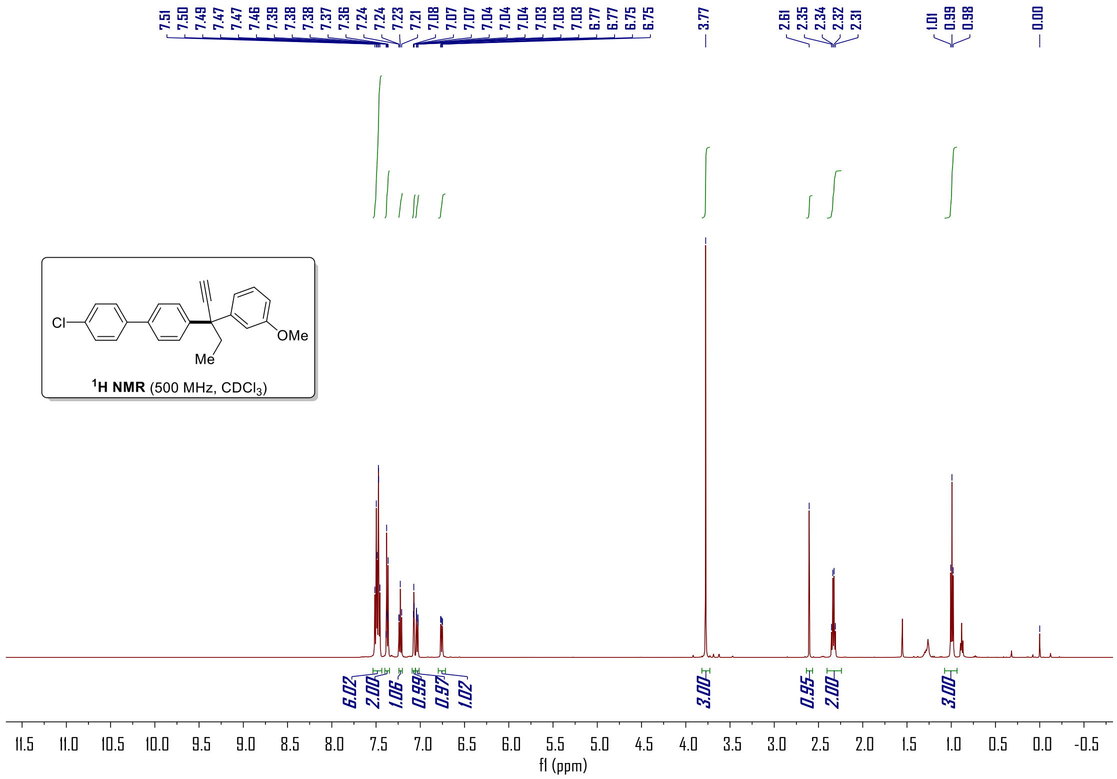

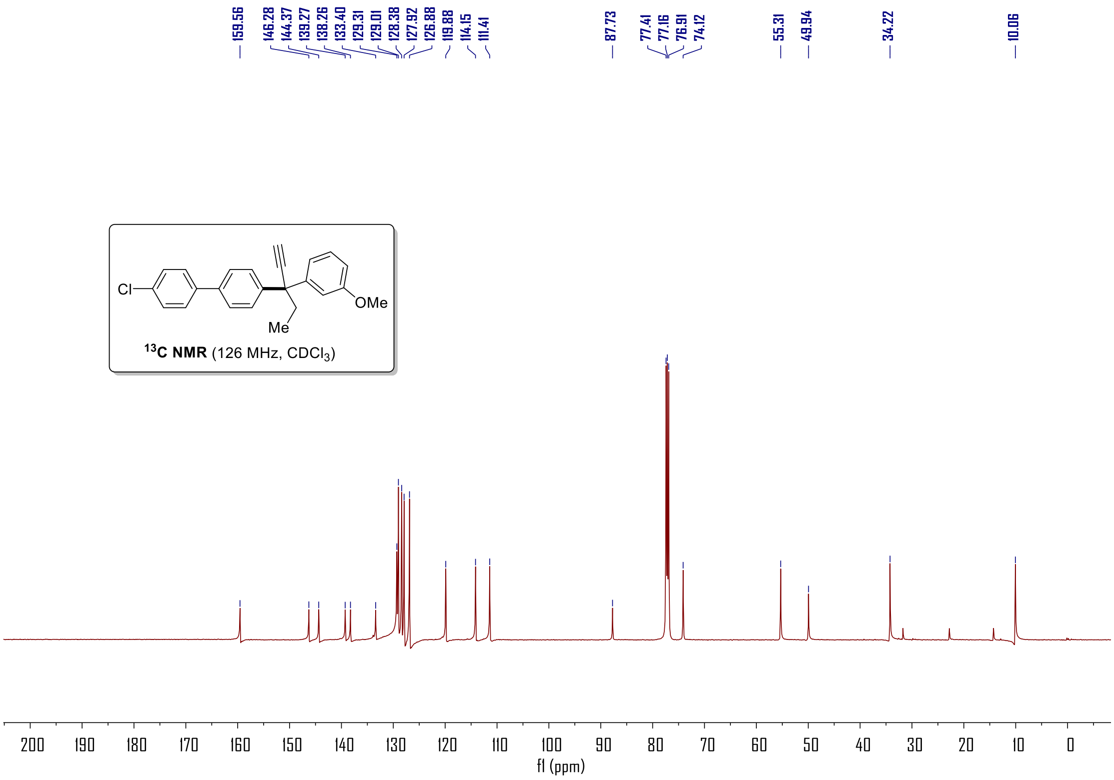

4-(3-(4-Methoxyphenyl)pent-1-yn-3-yl)-4'-(trifluoromethyl)-biphenyl (4lg)

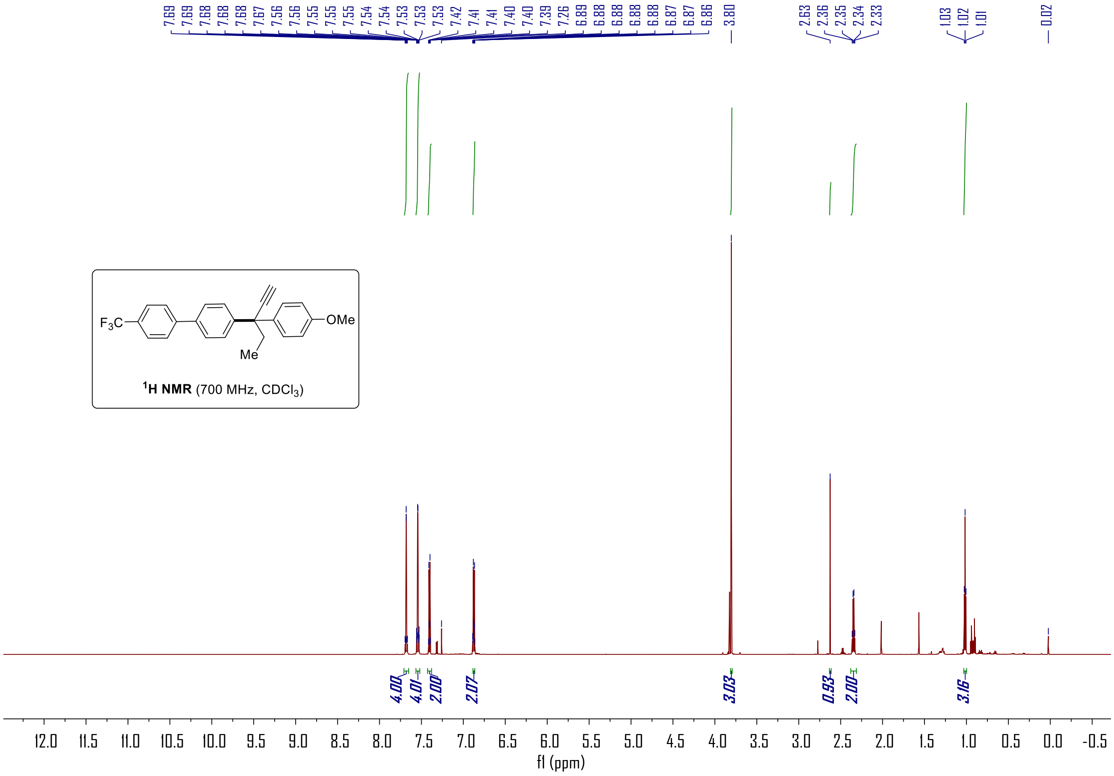

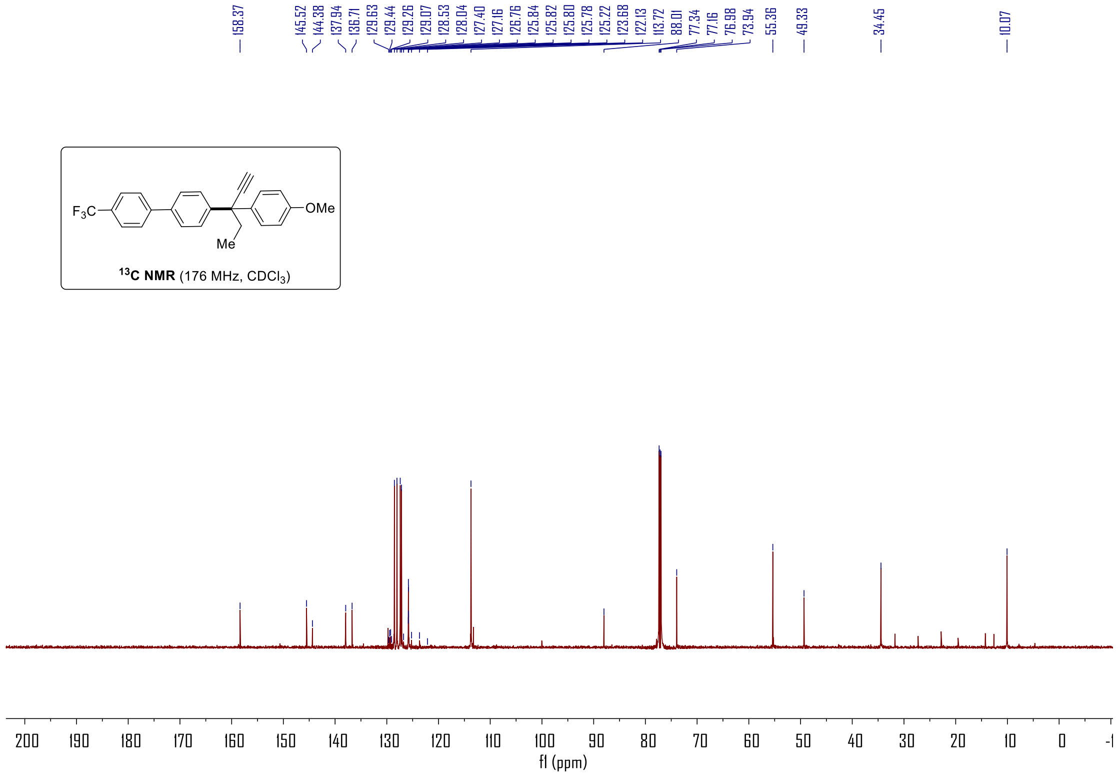
158.37
145.52
144.38
137.94
136.71
129.63
129.44
129.26
129.07
128.53
128.04
127.40
127.16
126.76
125.84
125.82
125.80
125.78
125.22
123.68
122.13
113.72
88.01
77.34
77.16
76.98
73.94
55.36
49.33
34.45
10.07
F3C
OMe
Me
13C NMR (176 MHz, CDCl3)
f1 (ppm)

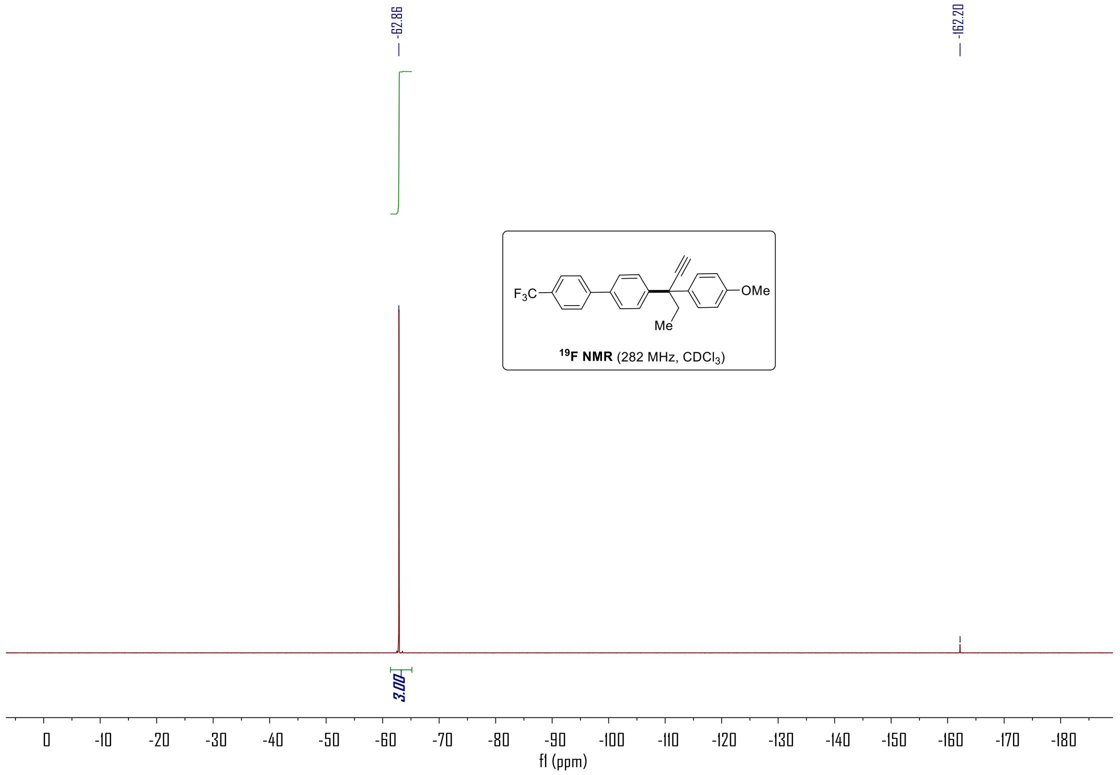
-62.86
-162.20
F3C
OMe
Me
19F NMR (282 MHz, CDCl3)
3.00
f1 (ppm)

4-(4-(3-Phenyloct-1-yn-3-yl)phenyl)pyridine (4nc)

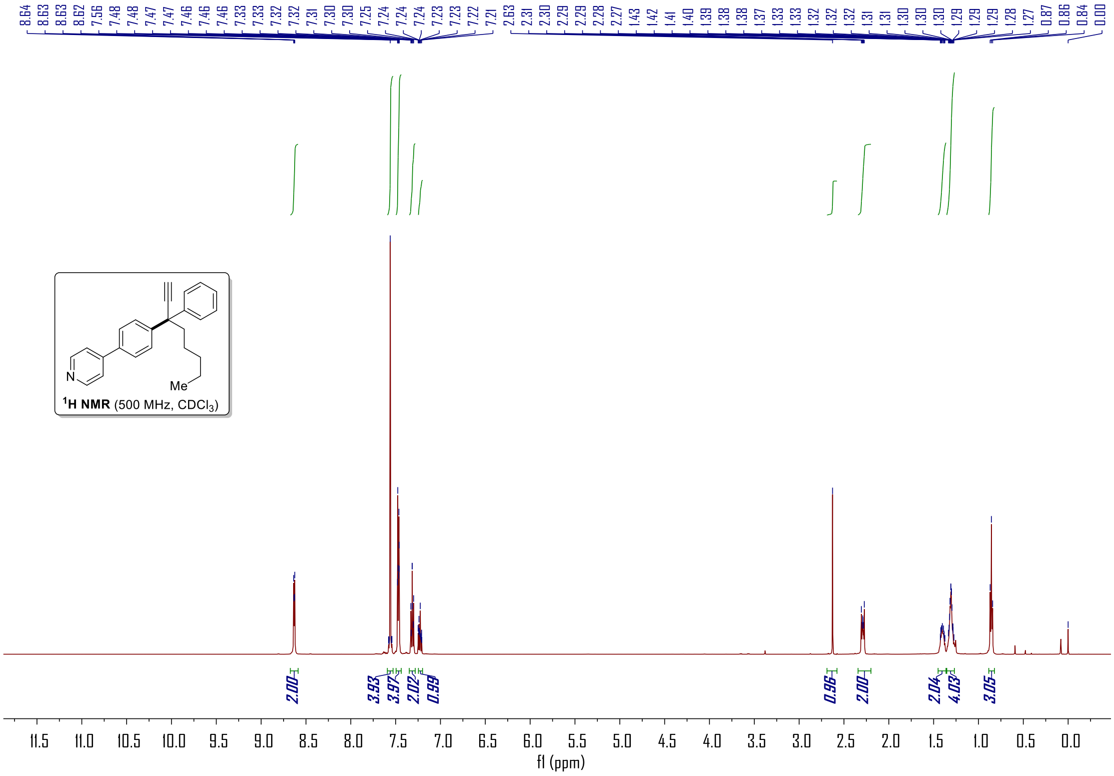

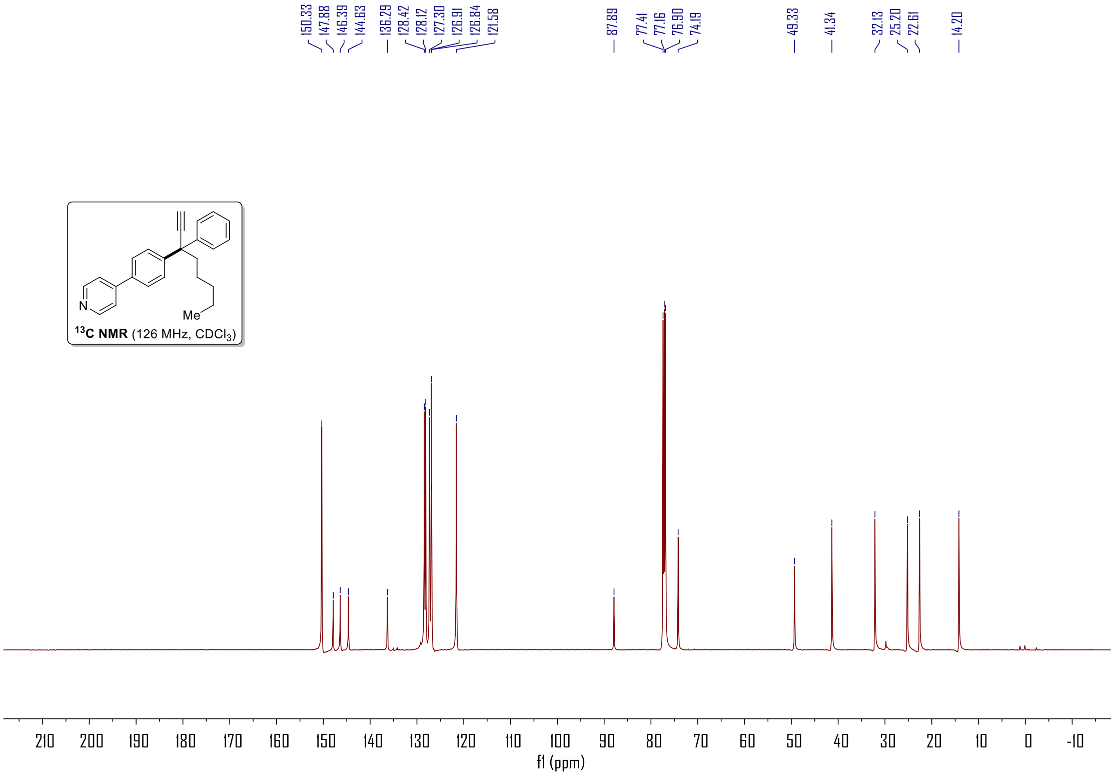

2-Phenyl-5-(3-(*p*-tolyl)pent-1-yn-3-yl)pyridine (4oi)

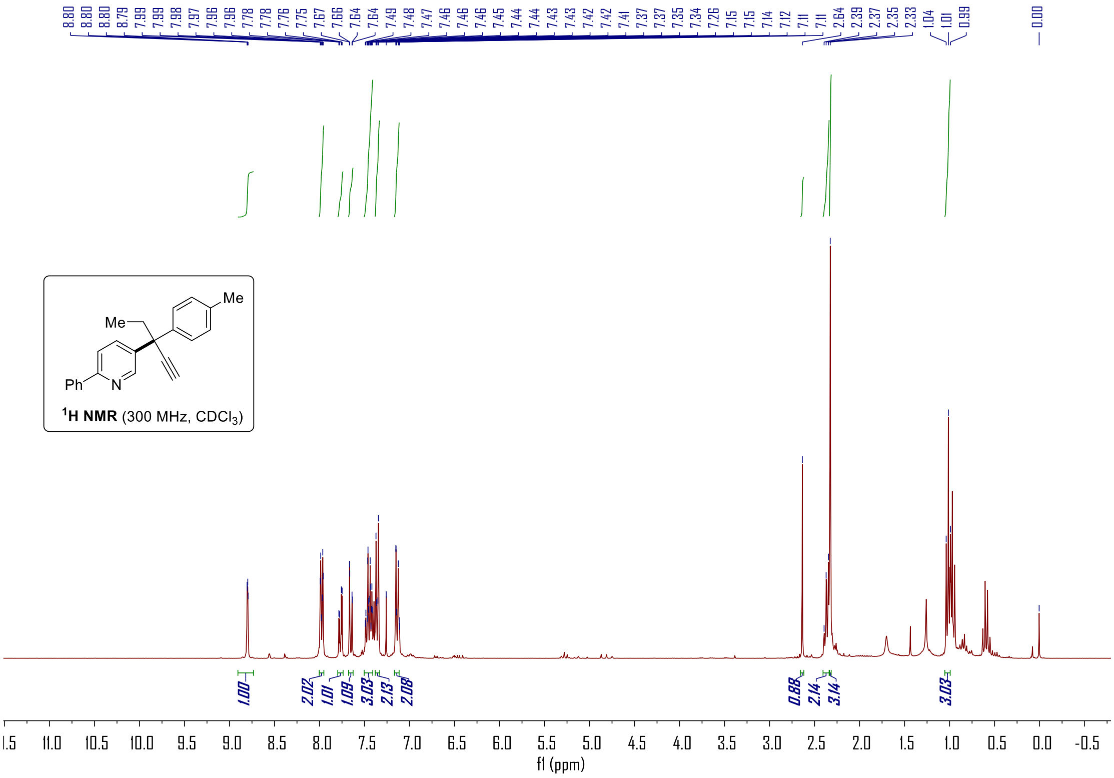

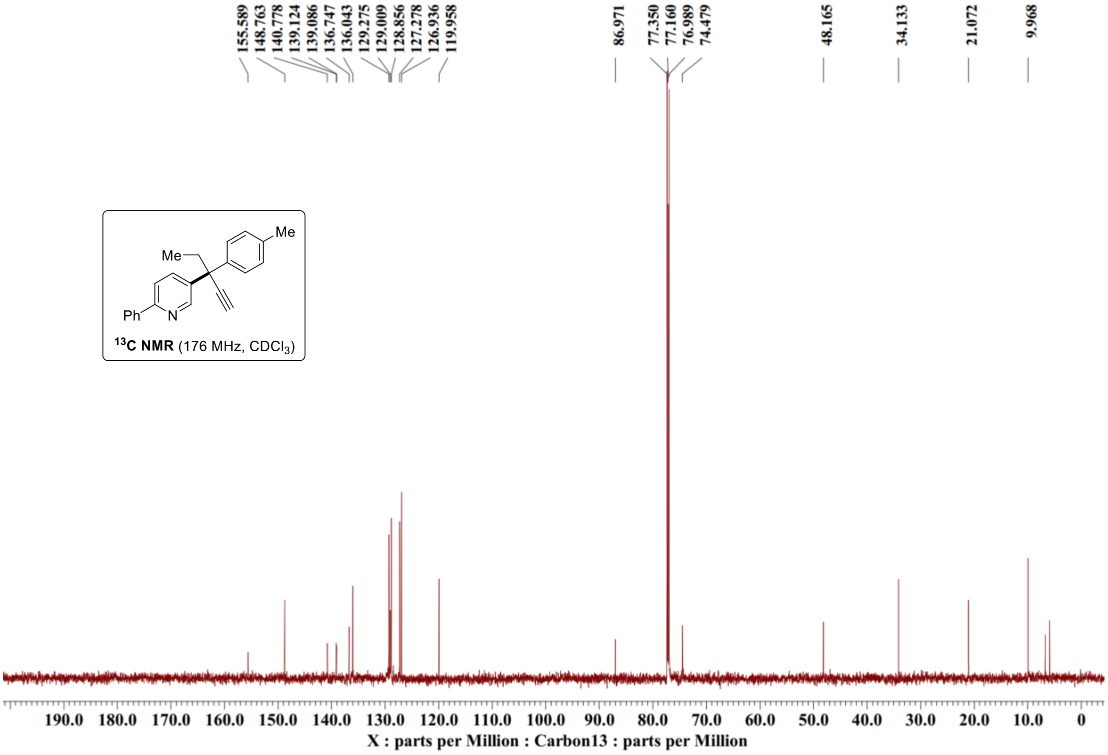

1-(4-(3-(4-Methoxyphenyl)pent-1-yn-3-yl)phenyl)-1*H*-pyrrole (4qg)

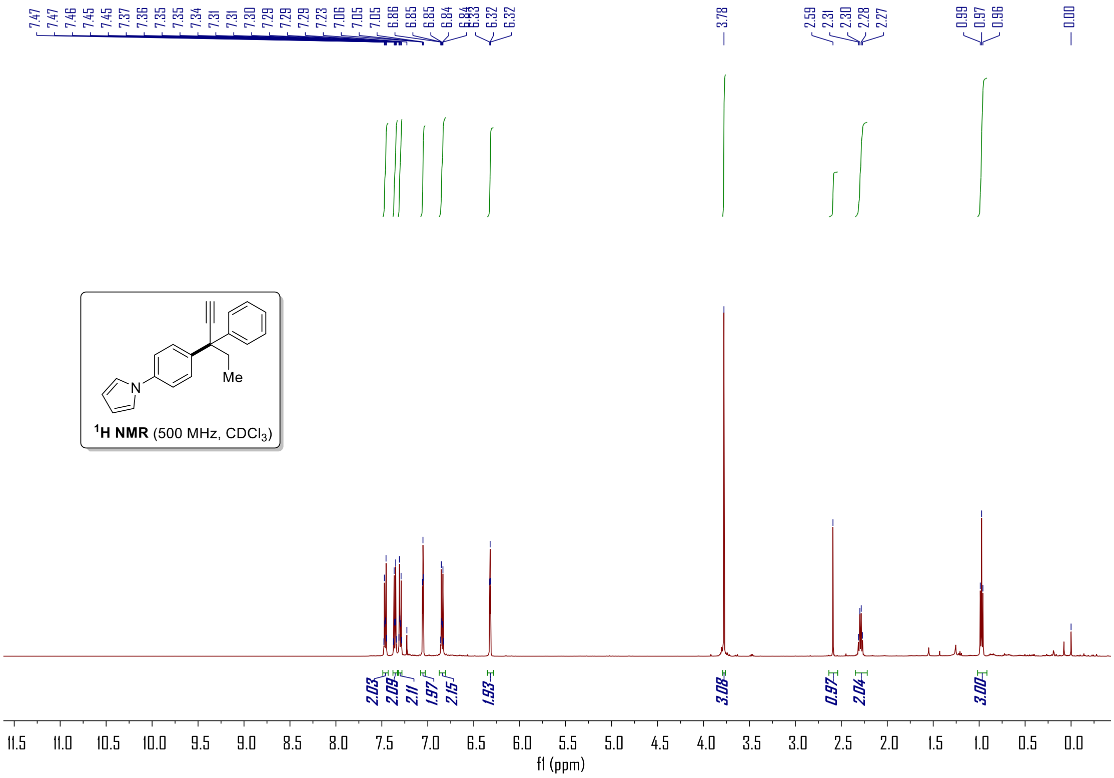

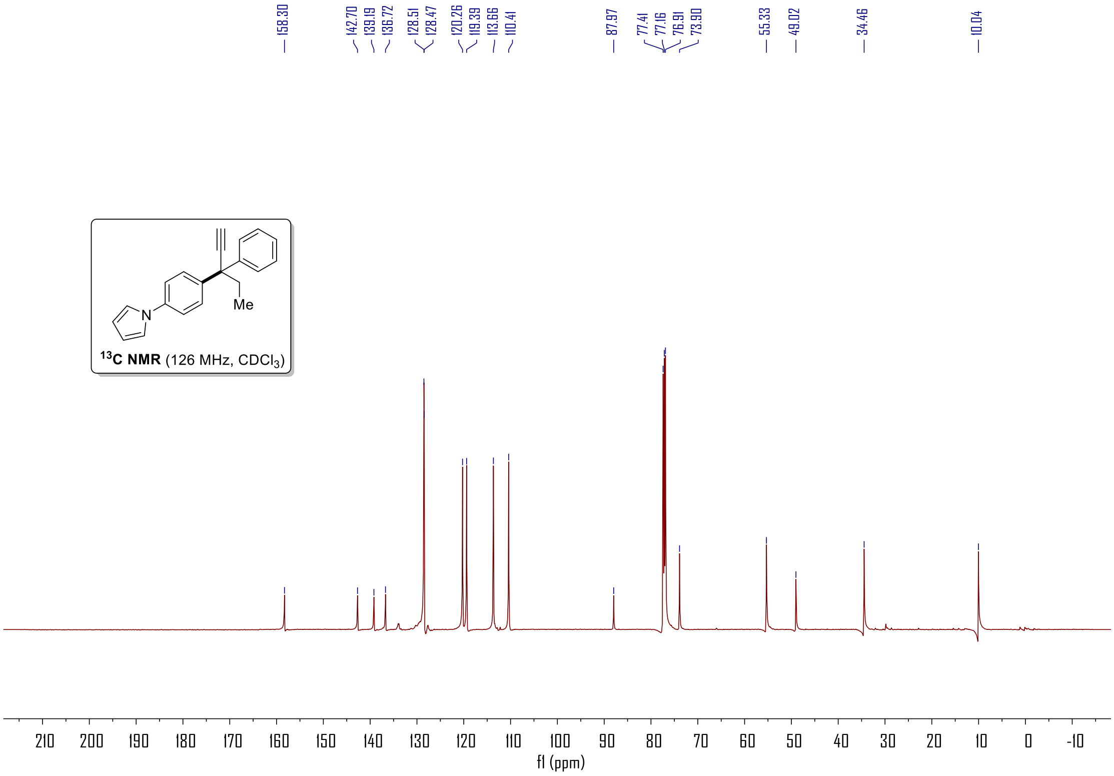

2-(4-(3-(3,5-Dimethylphenyl)pent-1-yn-3-yl)phenyl)-1-methyl-1*H*-indole (4rk)

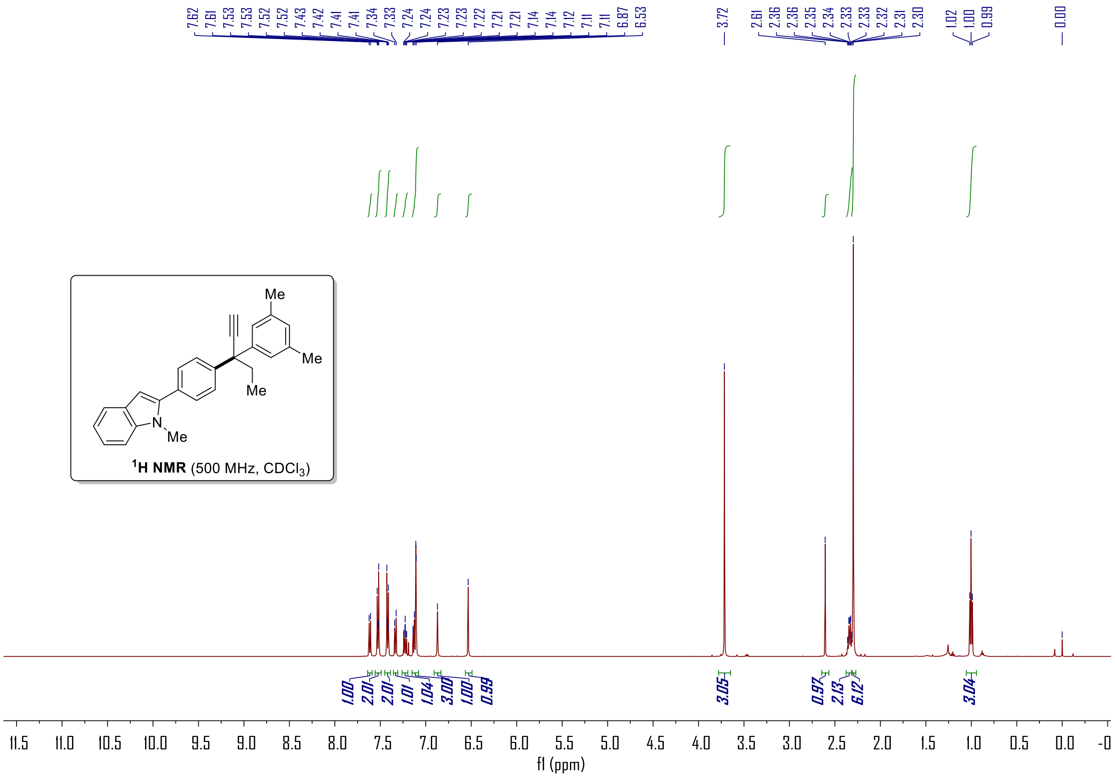

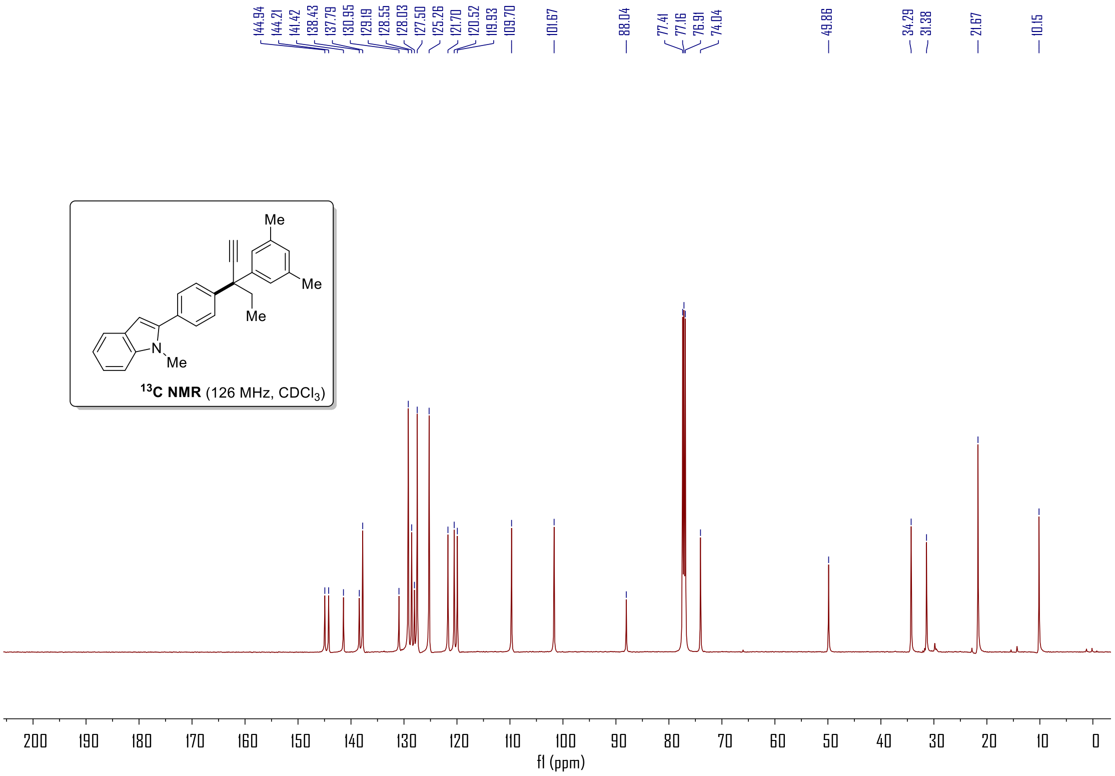

2-(3-(*p*-Tolyl)pent-1-yn-3-yl)benzofuran (4ui)

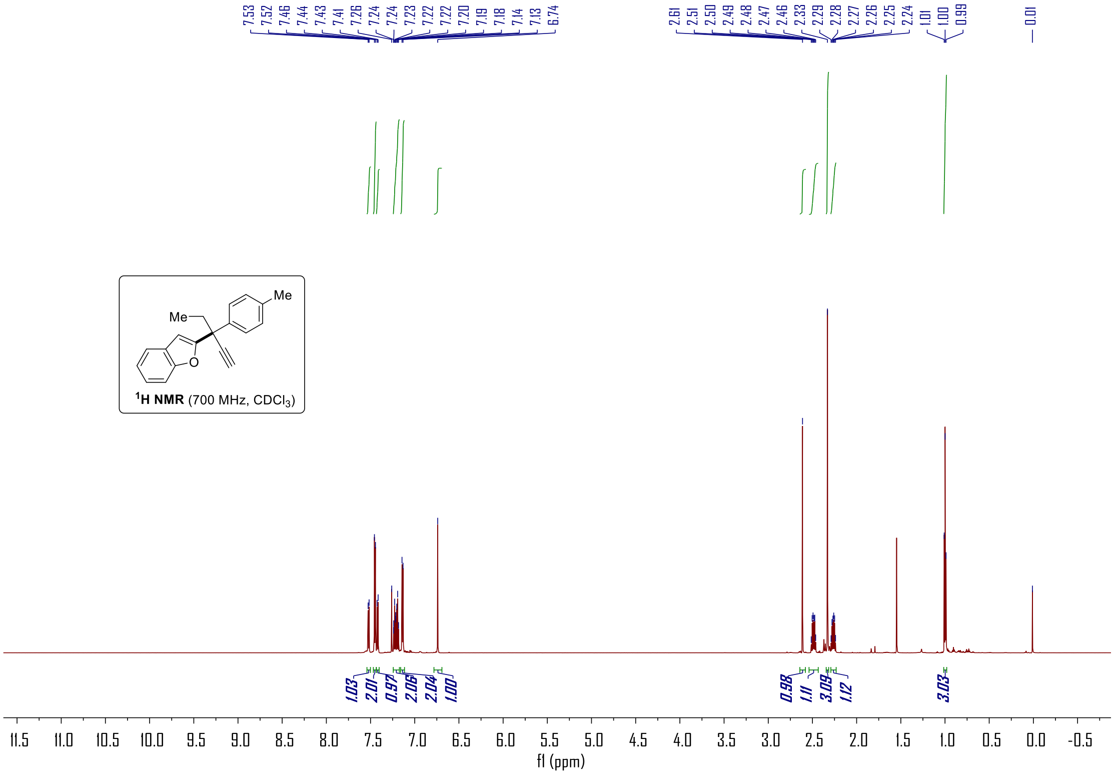

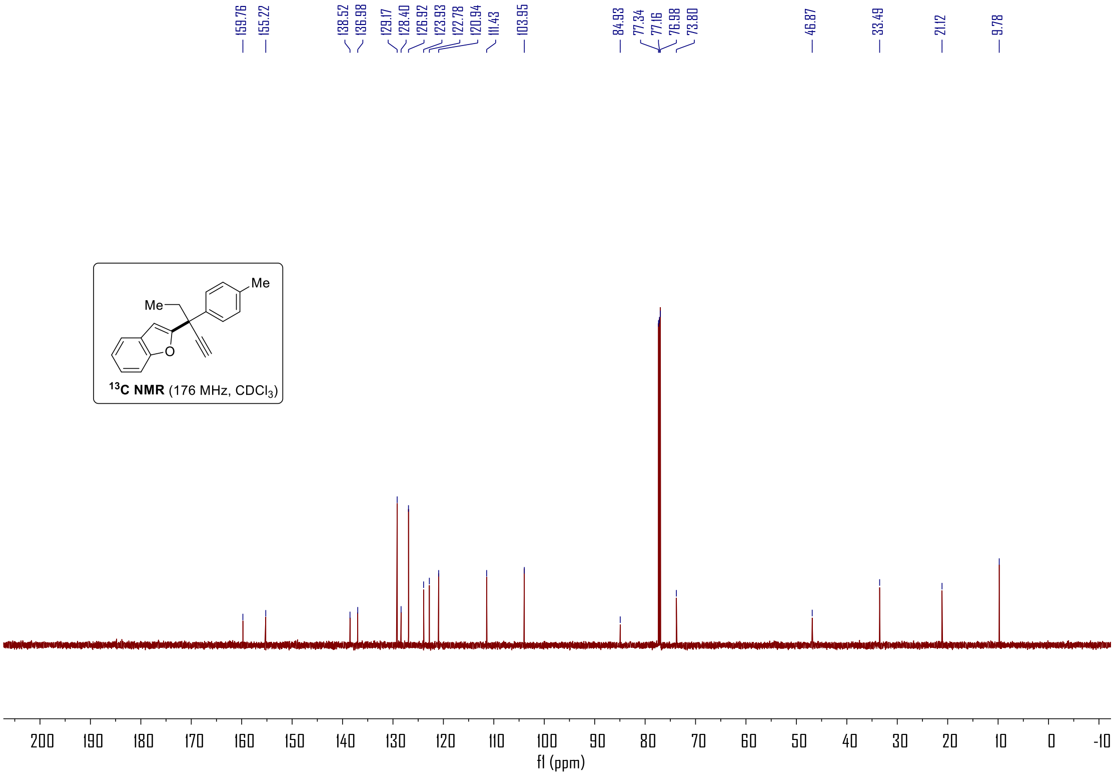

5-Phenyl-2-(3-phenylpent-1-yn-3-yl)pyridine (4ta)

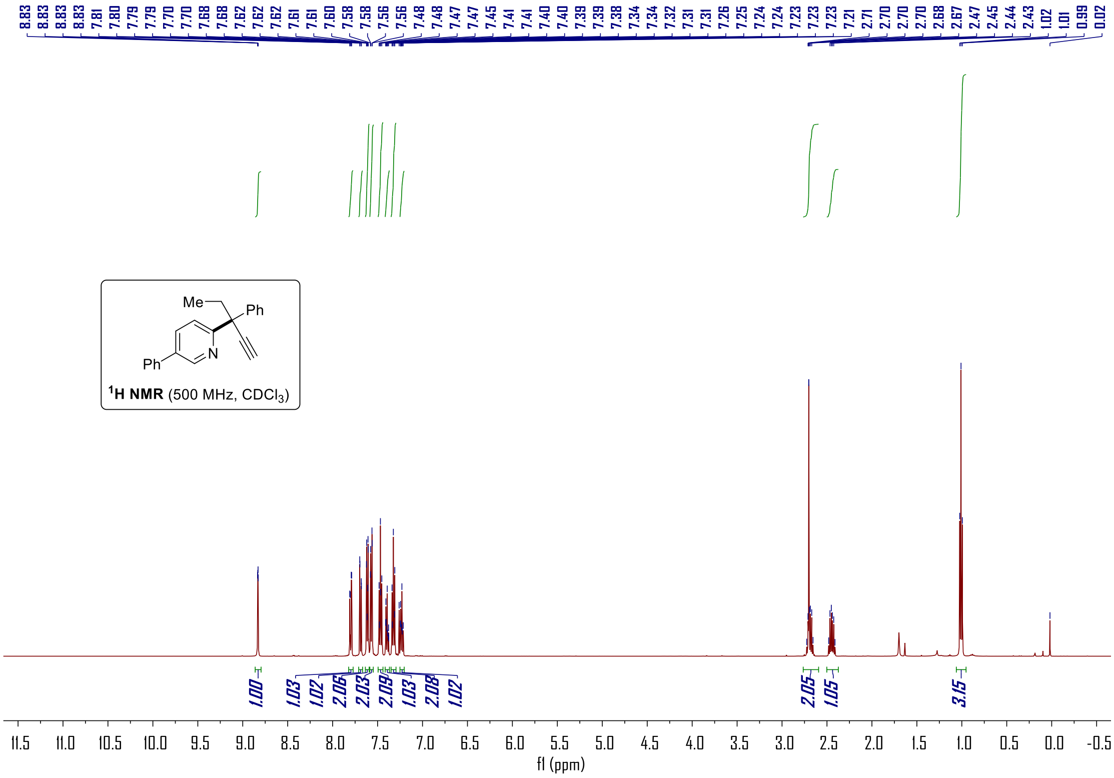

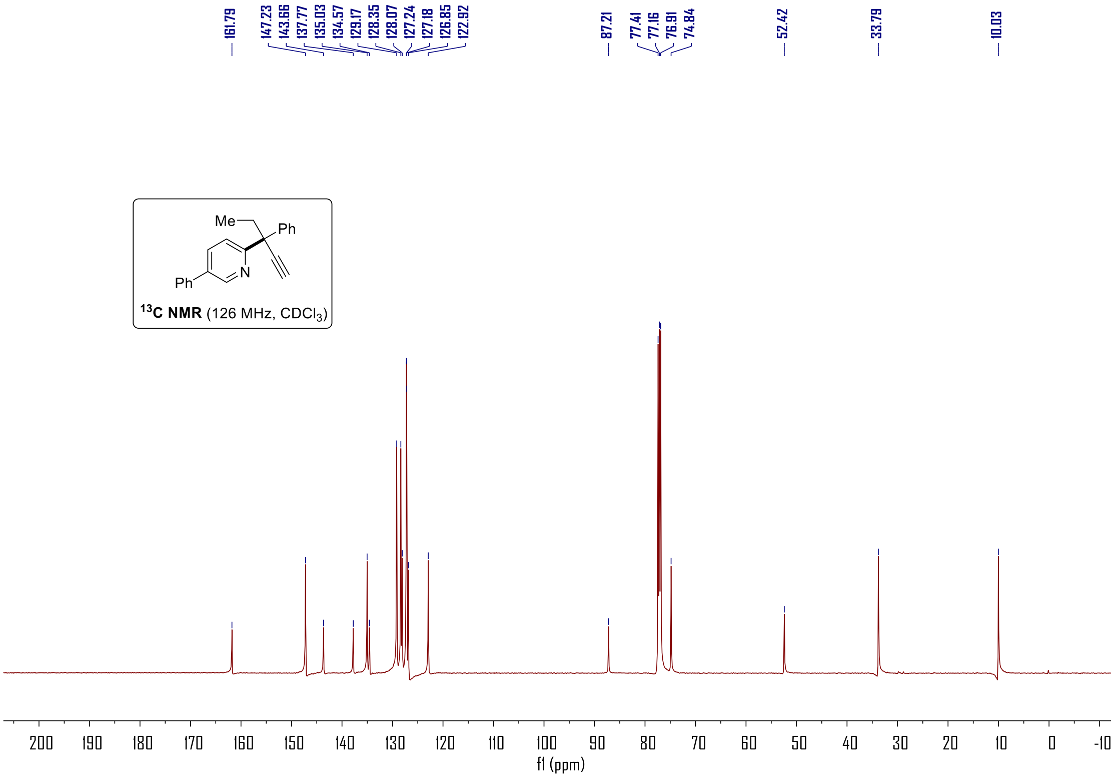

2-(3-Phenylpent-1-yn-3-yl)benzofuran (4ua)

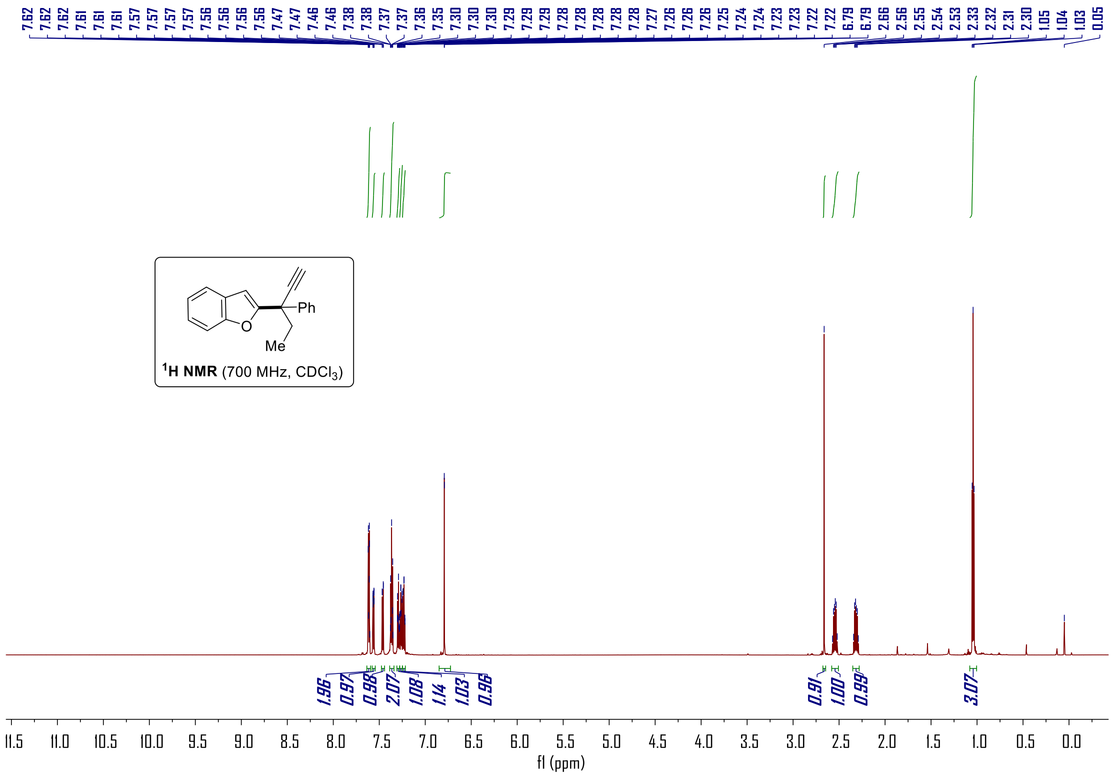

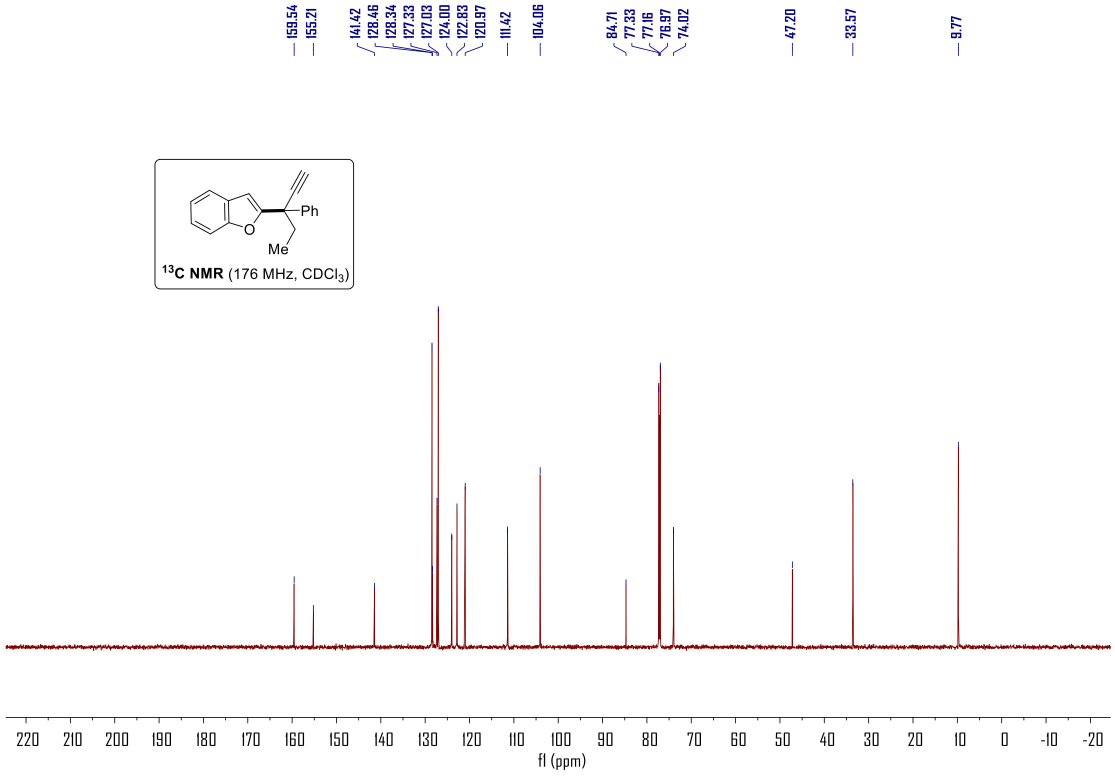

1-(3-Phenylpent-1-yn-3-yl)naphthalene (4va)

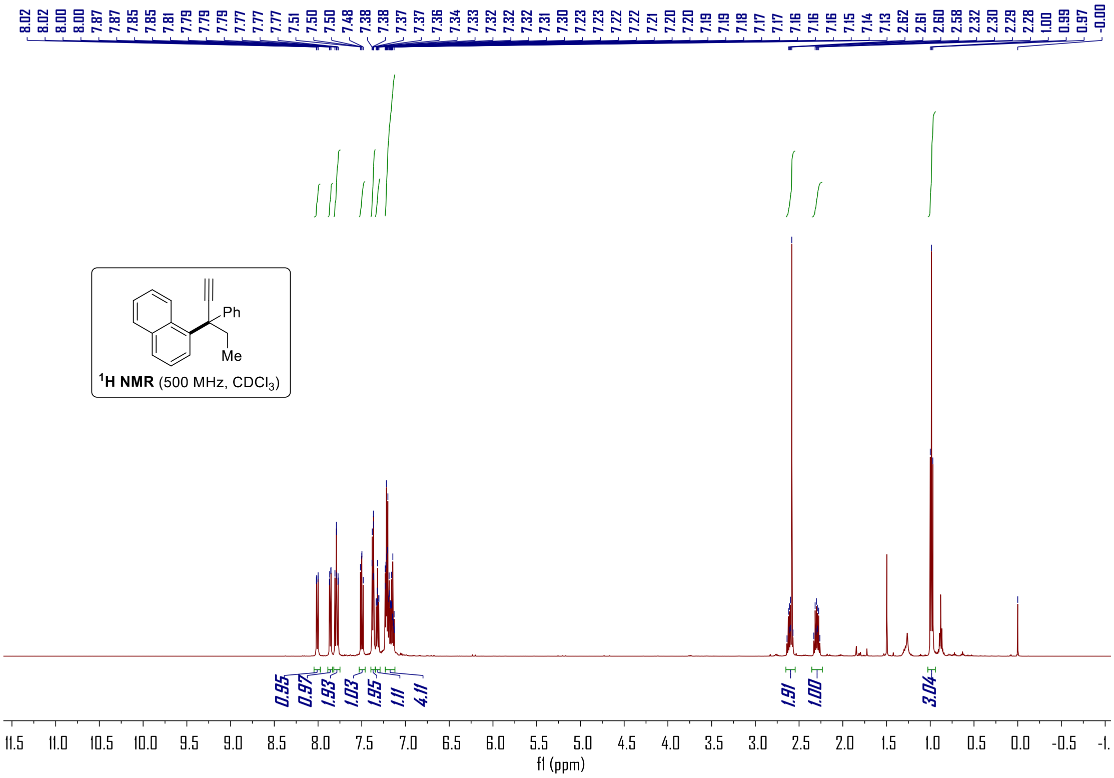

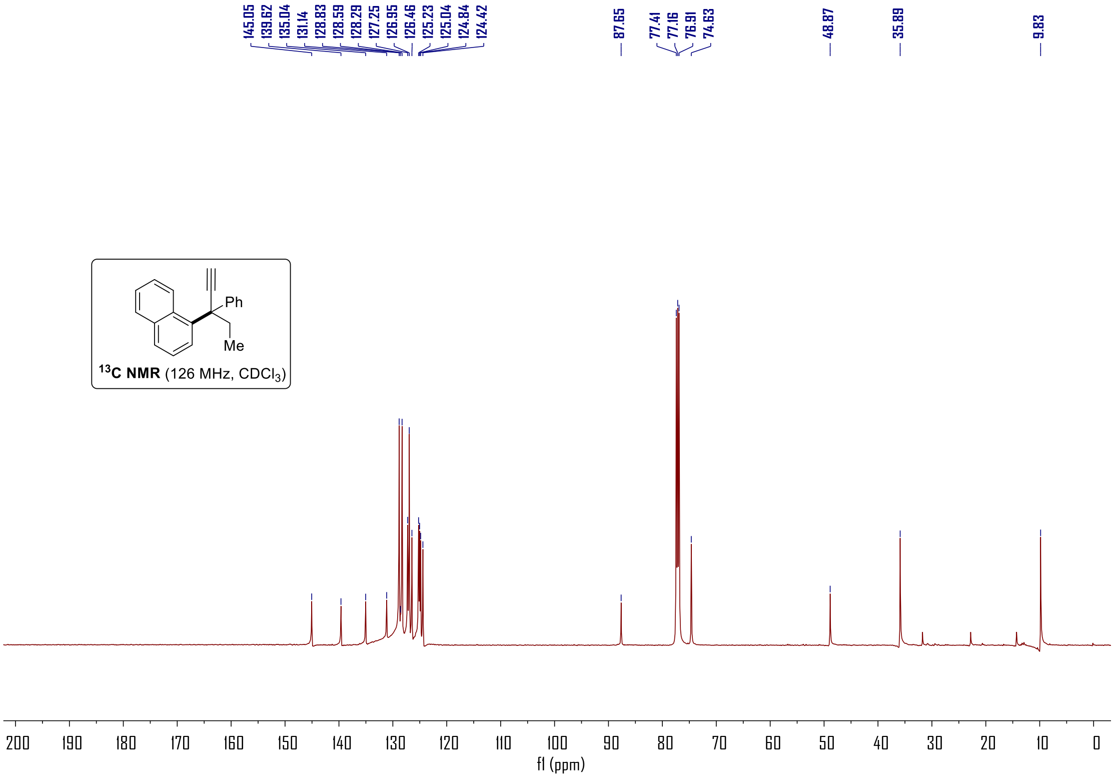

Triethyl(3-(3-phenylpent-1-yn-3-yl)phenyl)silane (4wa)

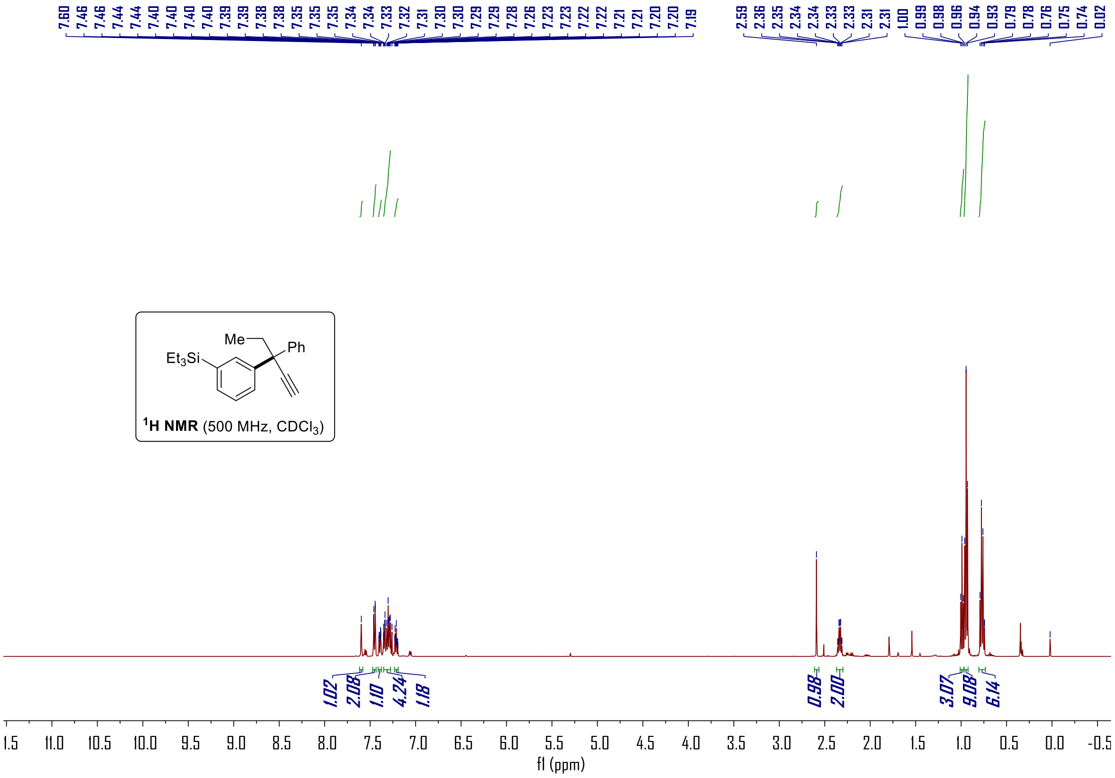

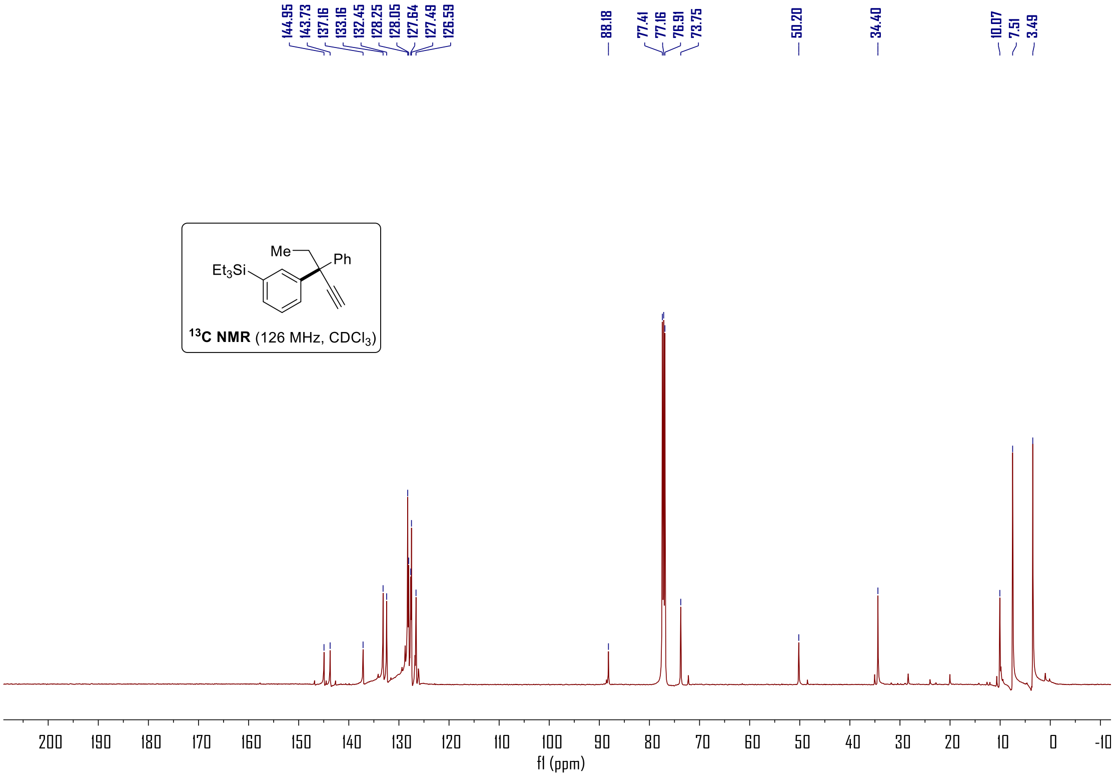

1-(3-Phenylpent-1-yn-3-yl)-4-(trifluoromethyl)benzene (4xa)

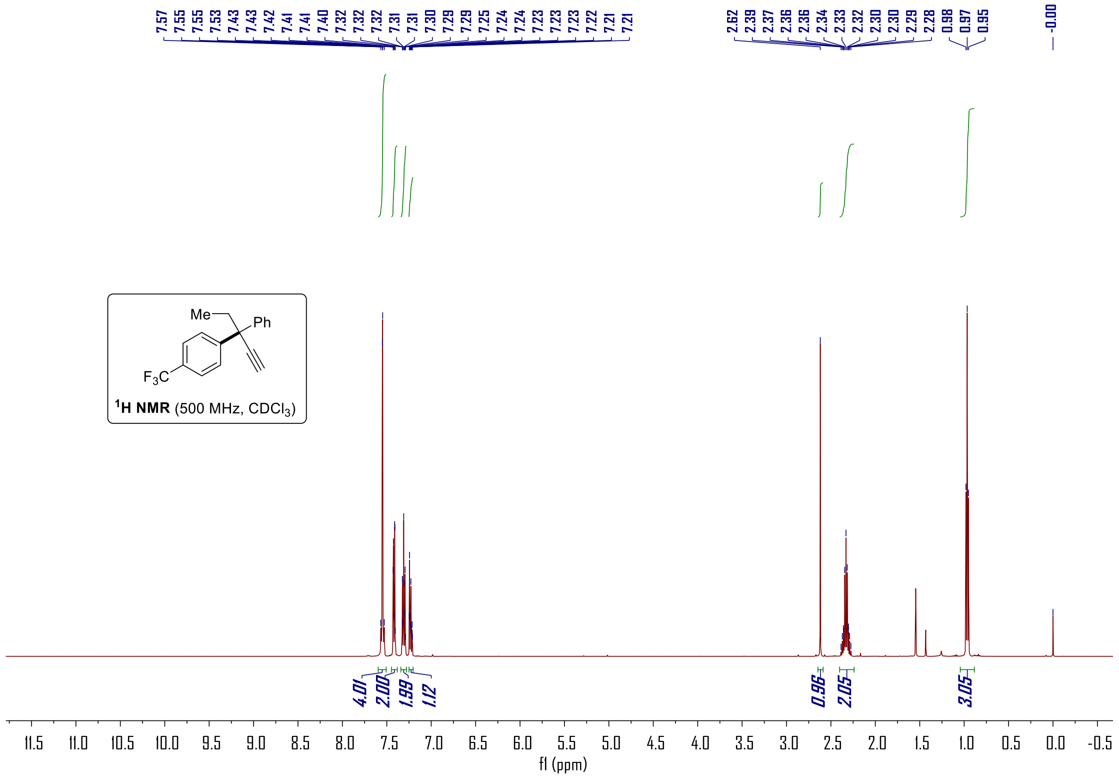

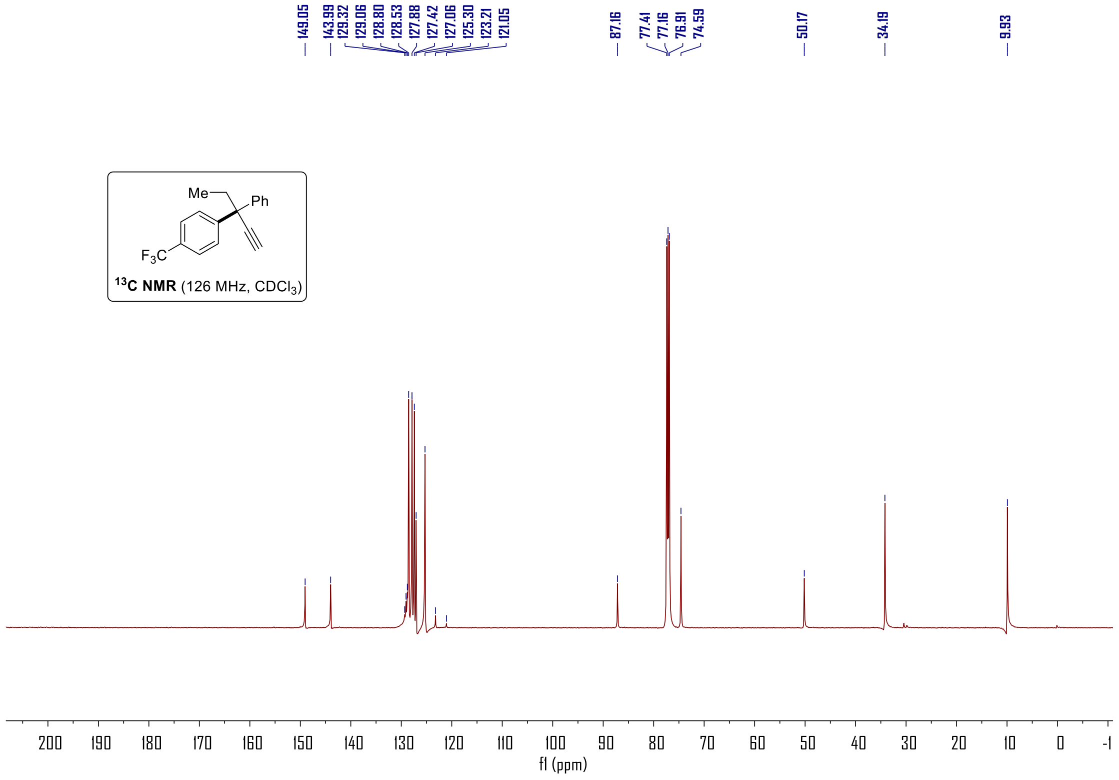

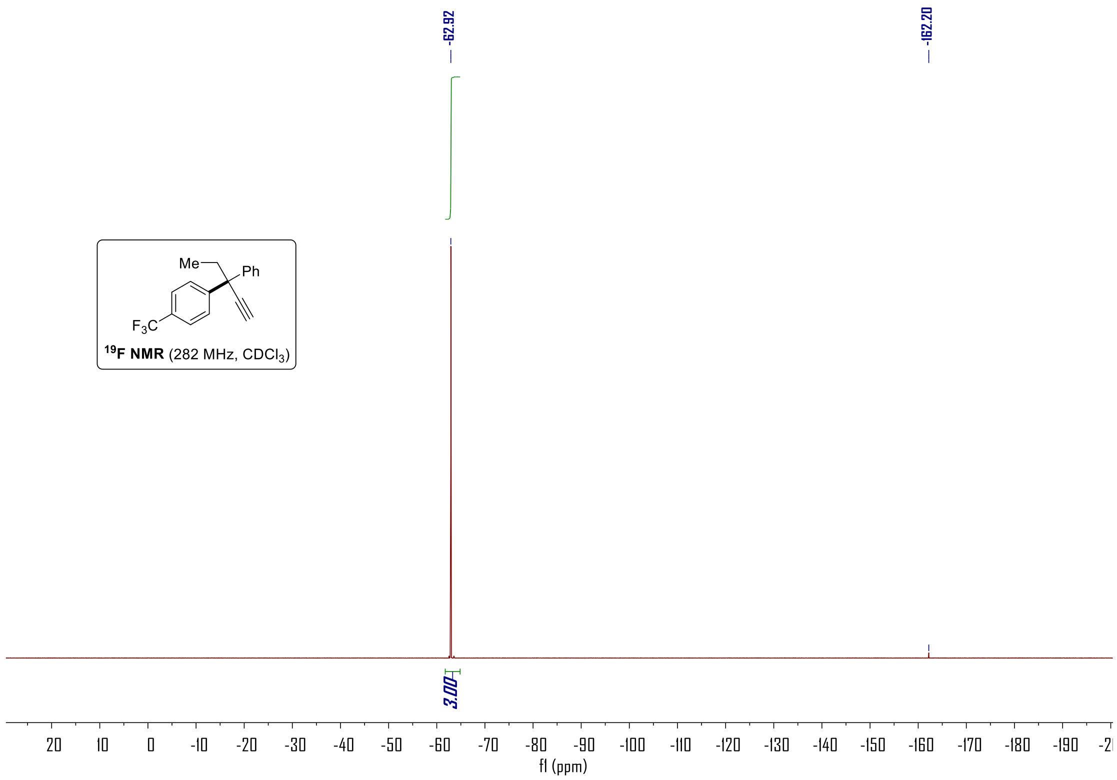

4-(2-Ethyl-2-phenylbut-3-yn-1-yl)-1,1'-biphenyl (5aa)

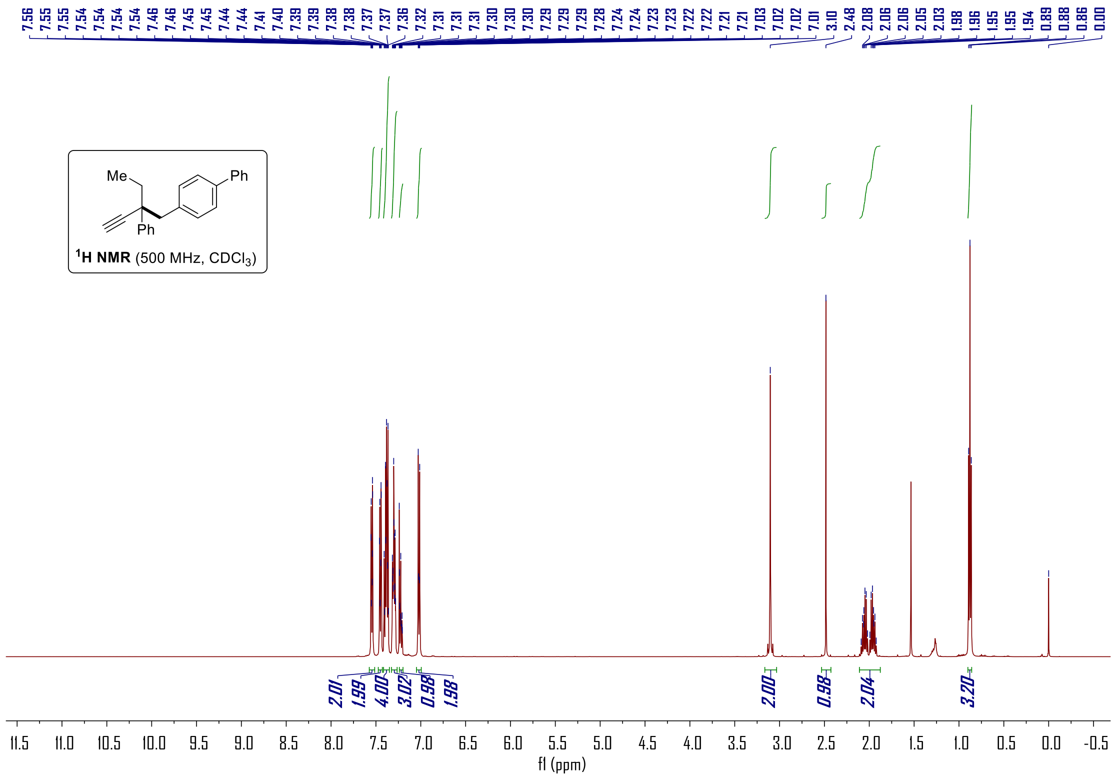

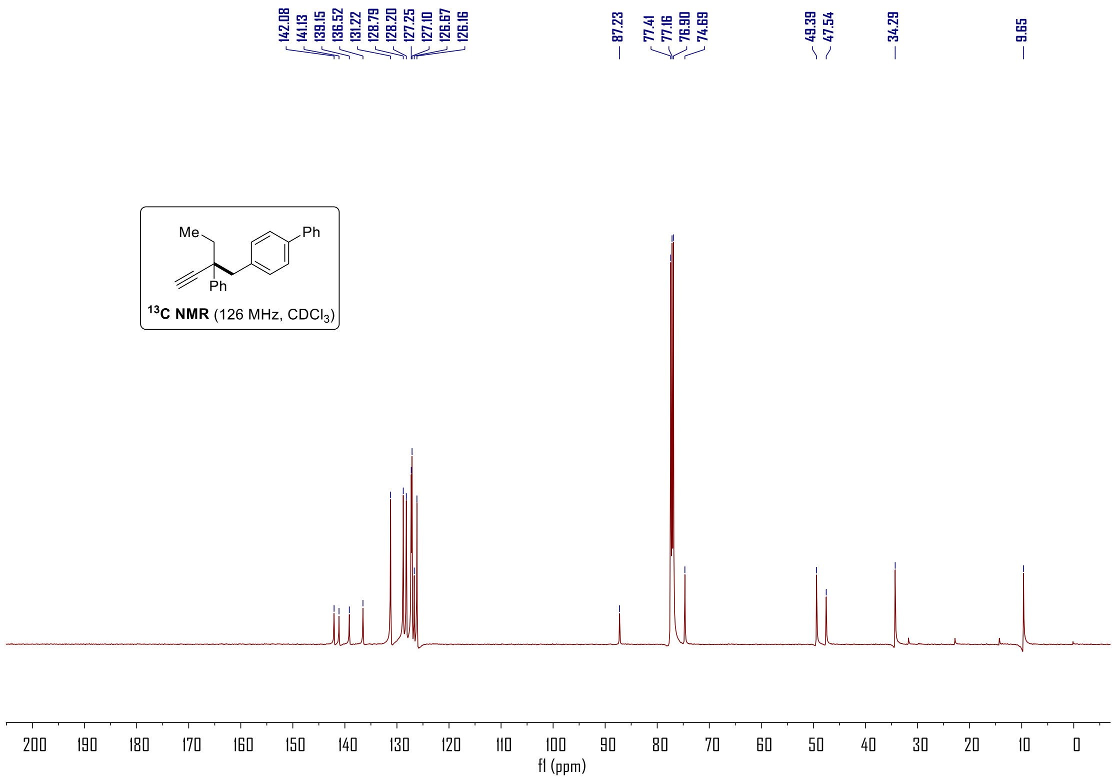

1-(2-Ethyl-2-phenylbut-3-yn-1-yl)-4-(trifluoromethoxy)benzene (5ba)

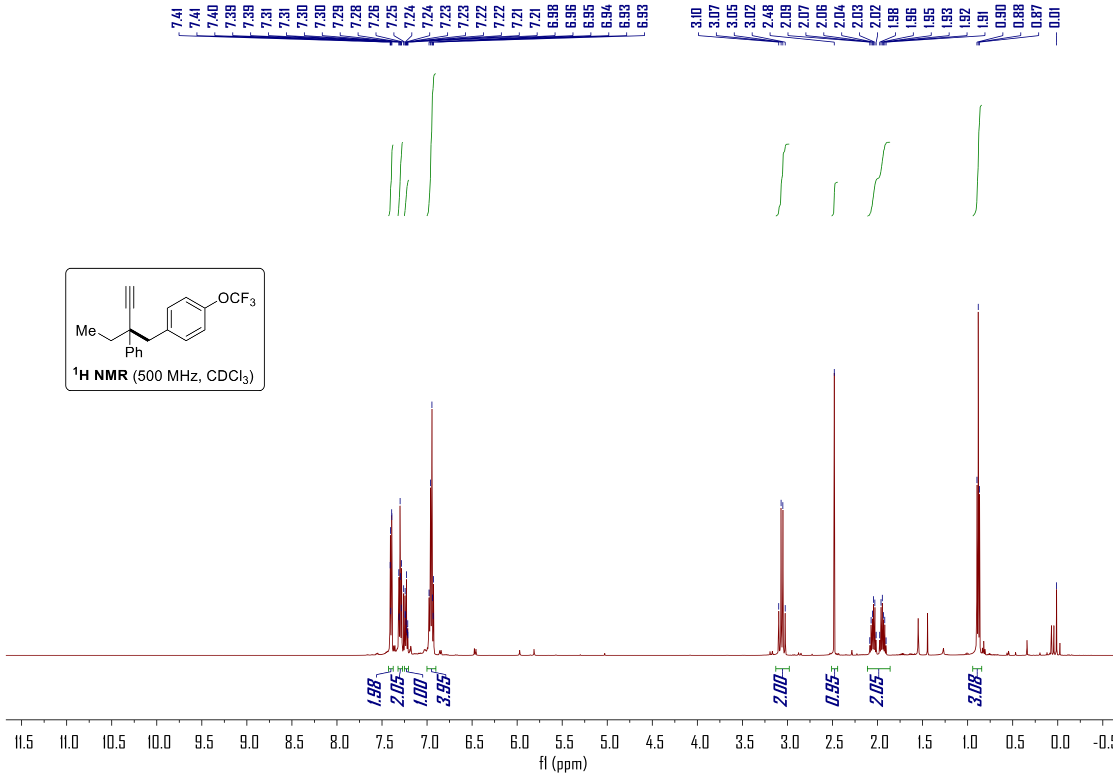

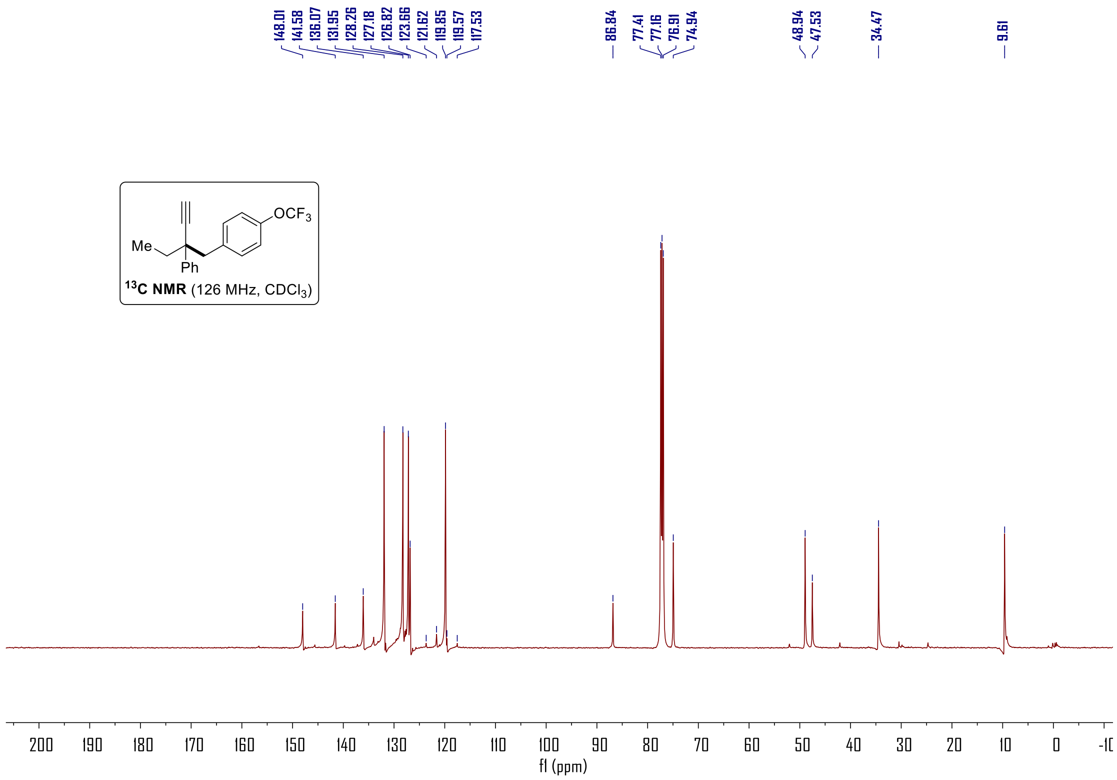

148.01
141.58
136.07
131.95
128.26
127.18
126.82
123.66
121.62
119.85
119.57
117.53
86.84
77.41
77.16
76.91
74.94
48.94
47.53
34.47
9.61
OCF3
Me
Ph
13C NMR (126 MHz, CDCl3)
fl (ppm)

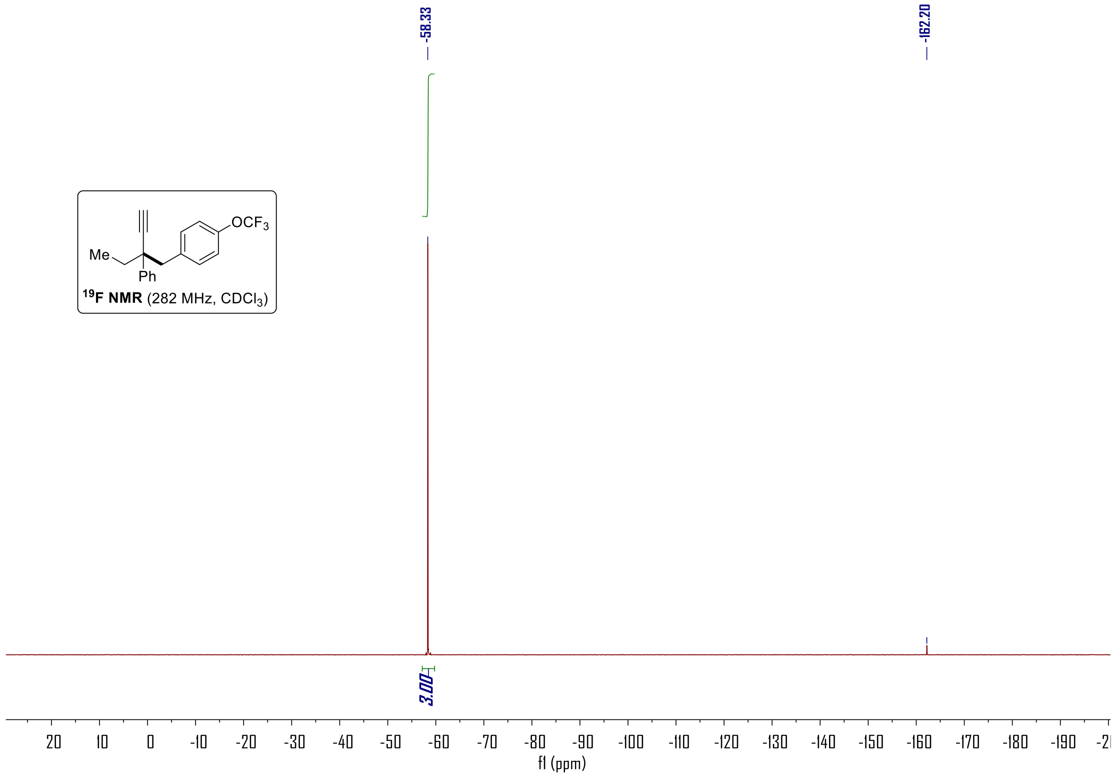

-58.33
-162.20
OCF3
Me
Ph
19F NMR (282 MHz, CDCl3)
3.00
fl (ppm)

1-Chloro-4-(2-ethyl-2-phenylbut-3-yn-1-yl)benzene (5ca)

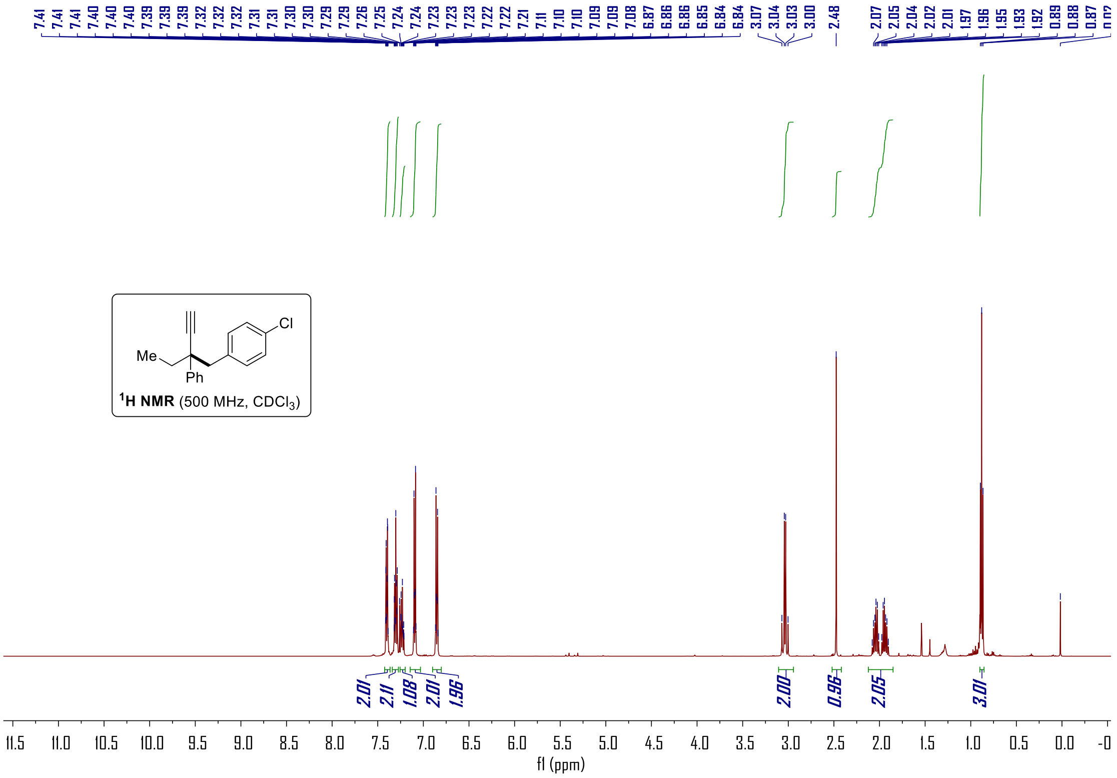

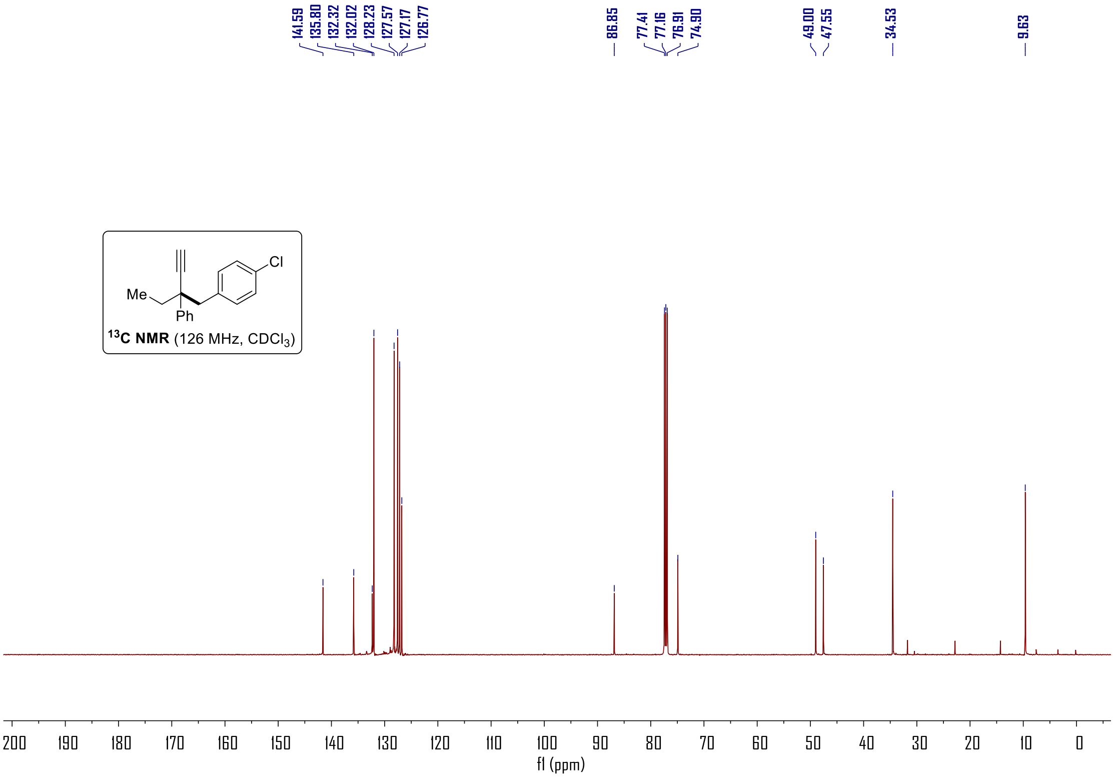

1-Bromo-4-(2-ethyl-2-phenylbut-3-yn-1-yl)benzene (5da)

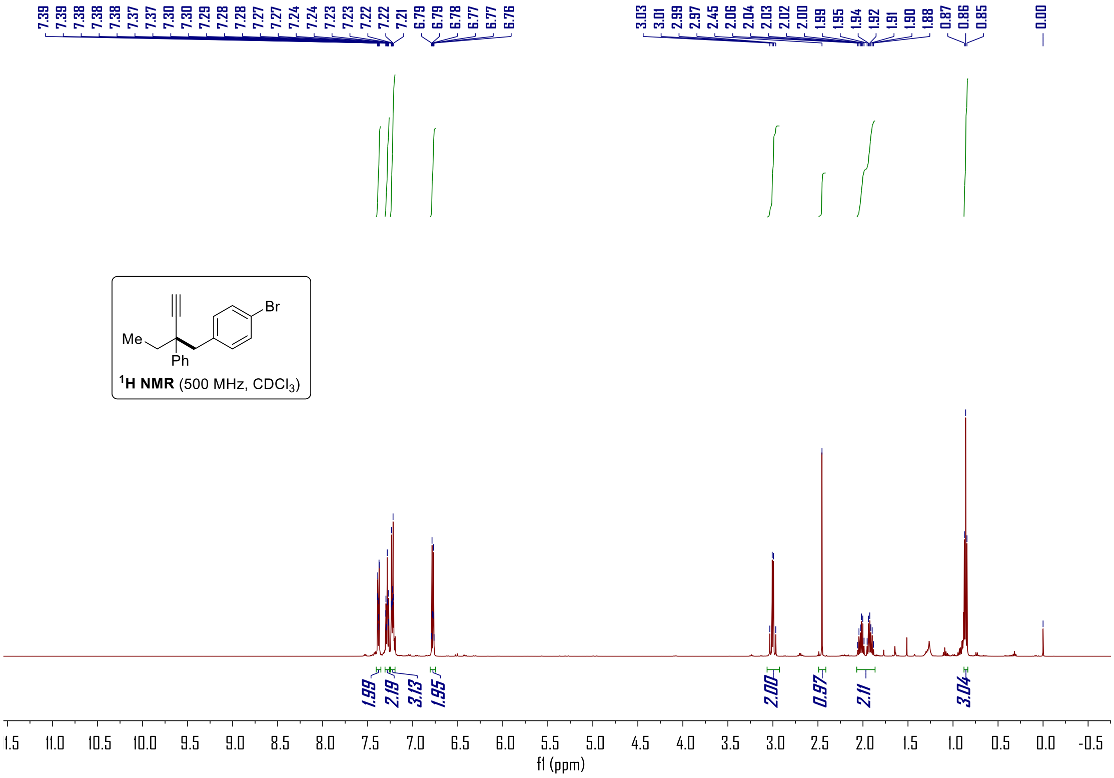

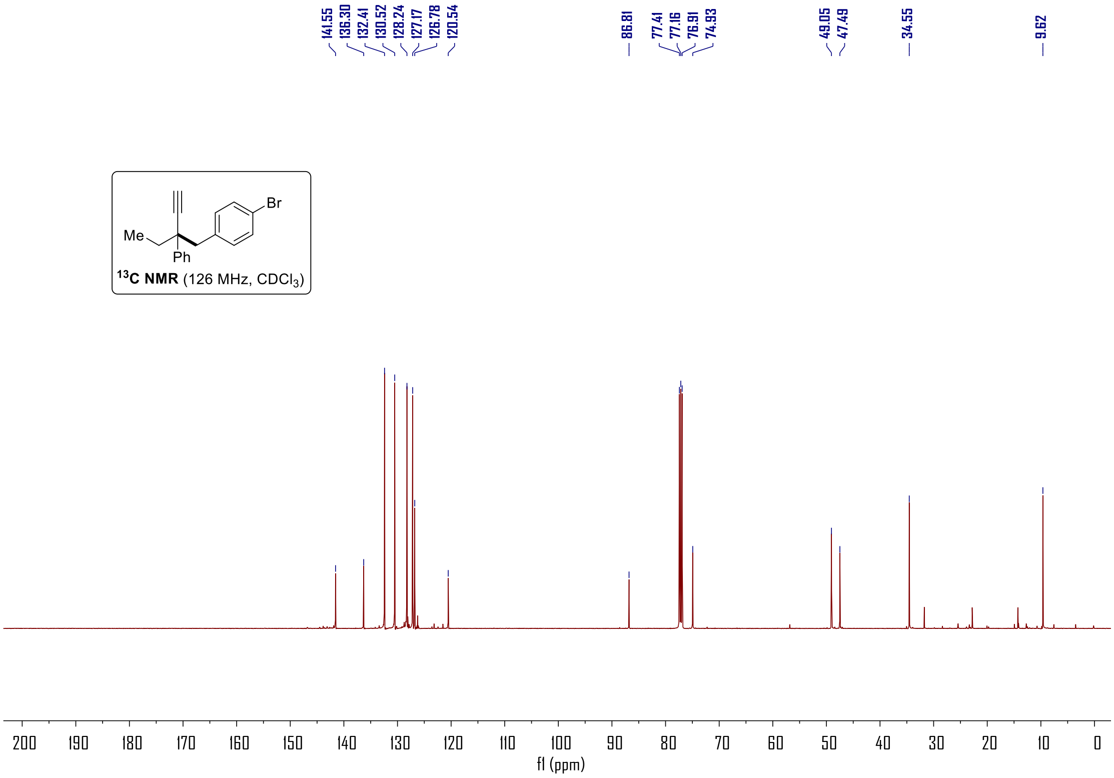

1-(*tert*-Butyl)-4-(2-ethyl-2-phenylbut-3-yn-1-yl)benzene (xx) (5ea)

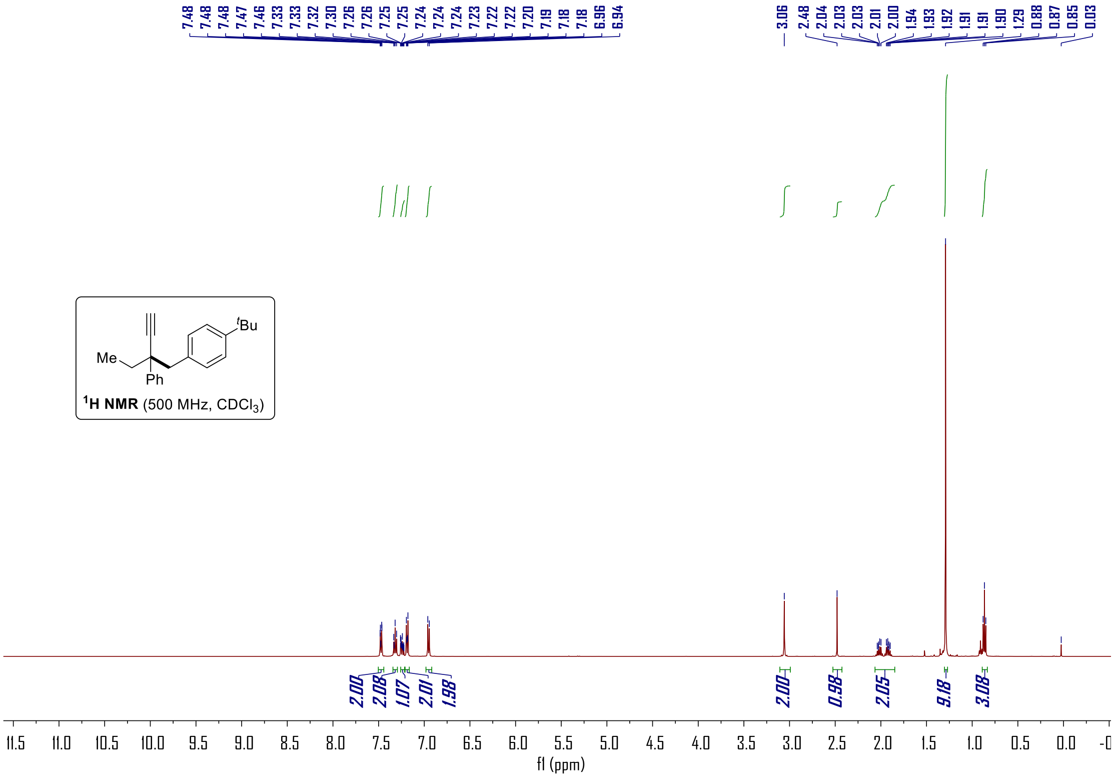

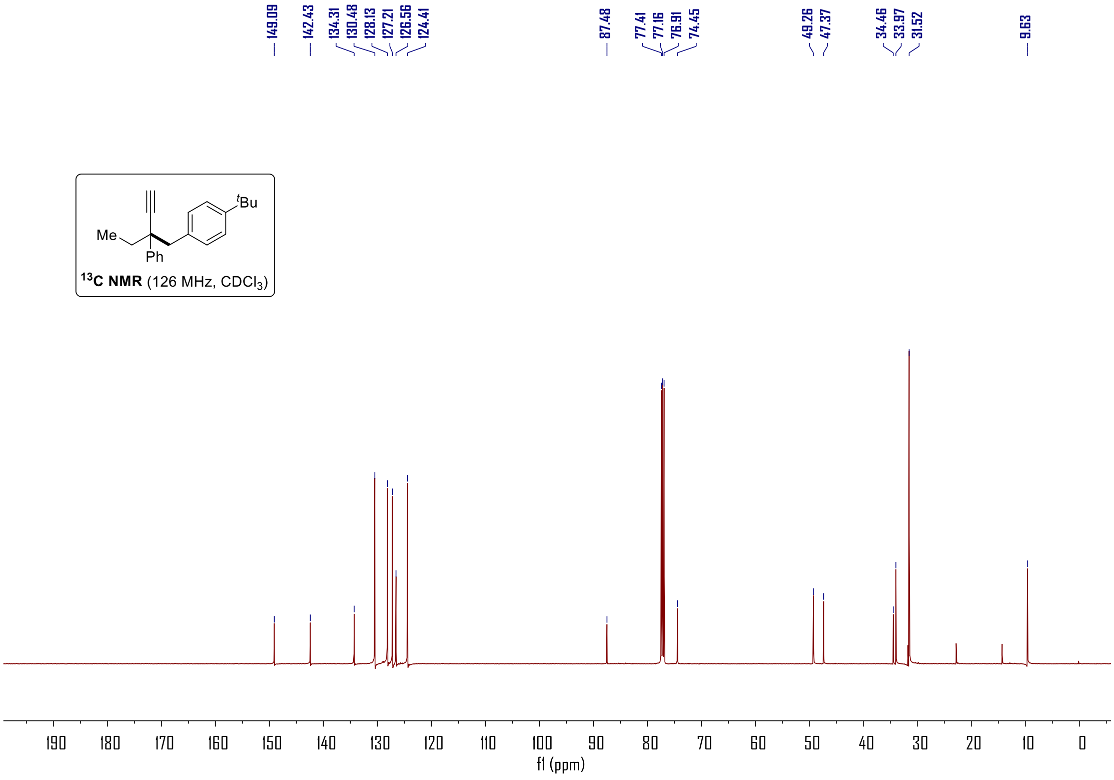

2-(2-Ethyl-2-phenylbut-3-yn-1-yl)naphthalene (5fa)

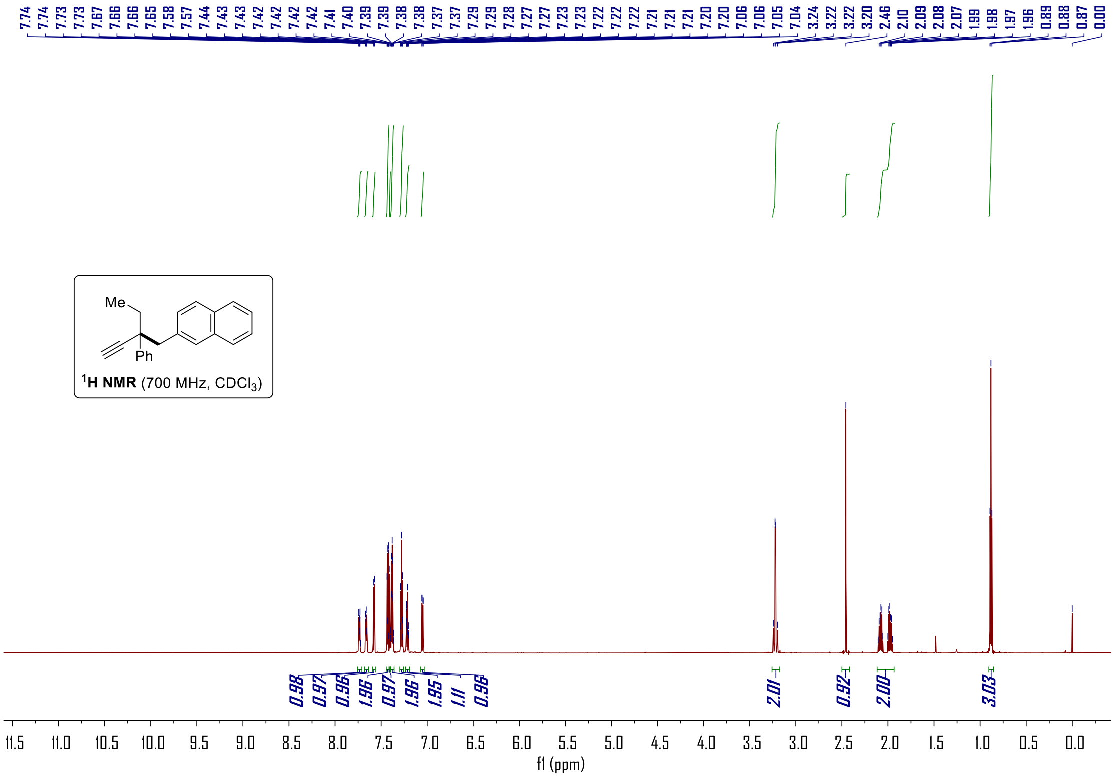

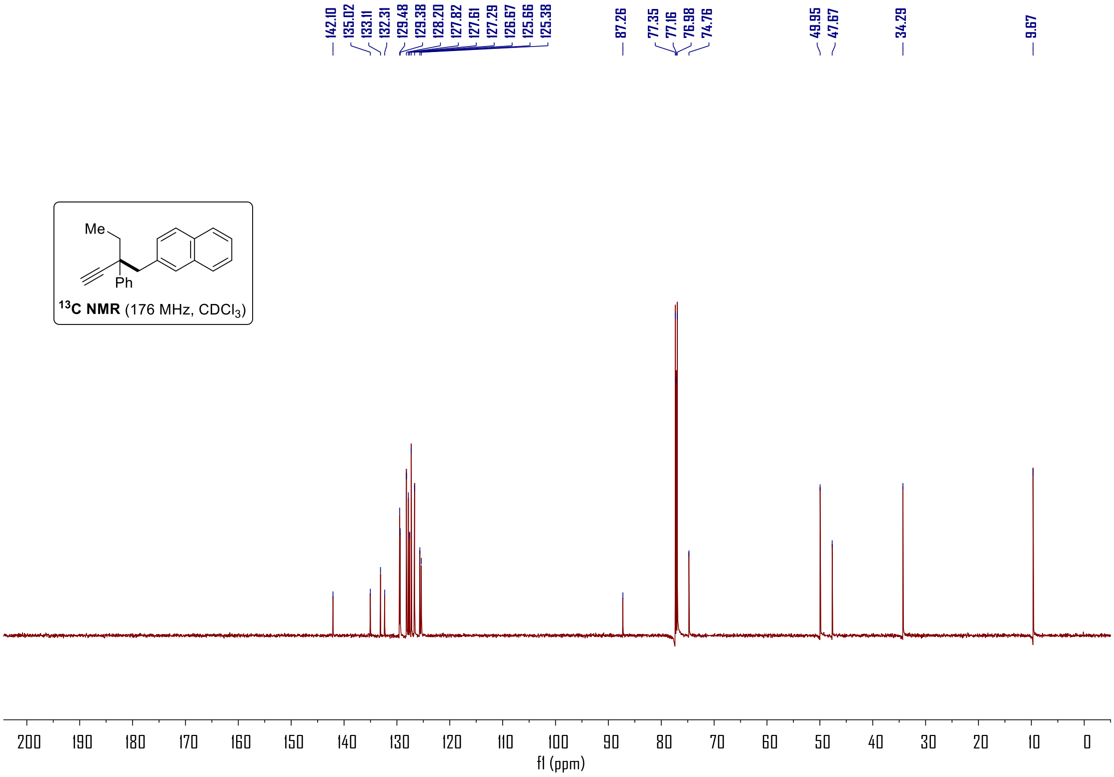

1-(2-Ethyl-2-phenylbut-3-yn-1-yl)naphthalene (5ga)

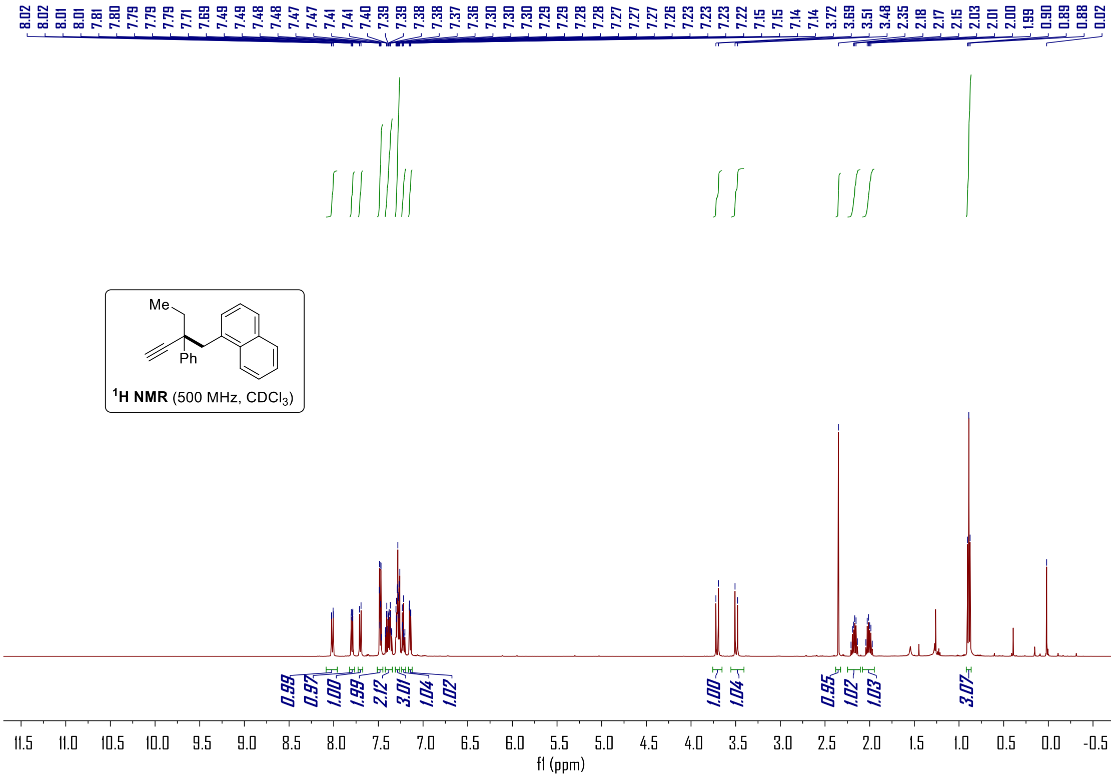

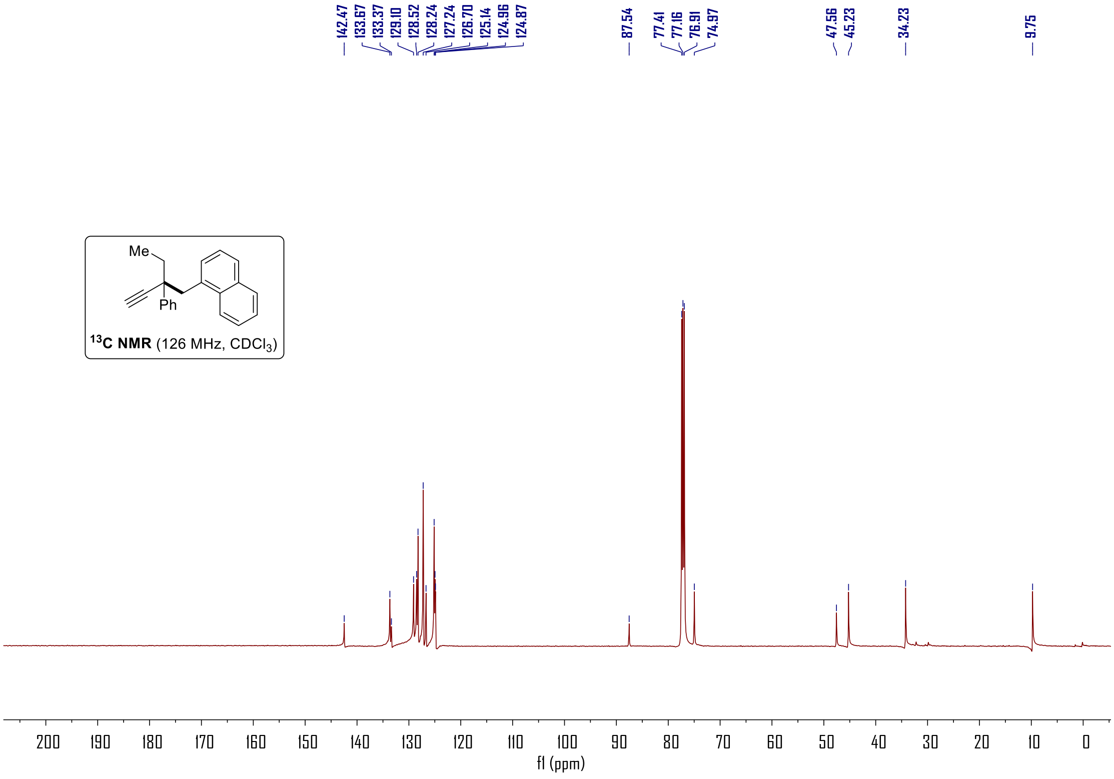

Adapalene derivative (4adc)

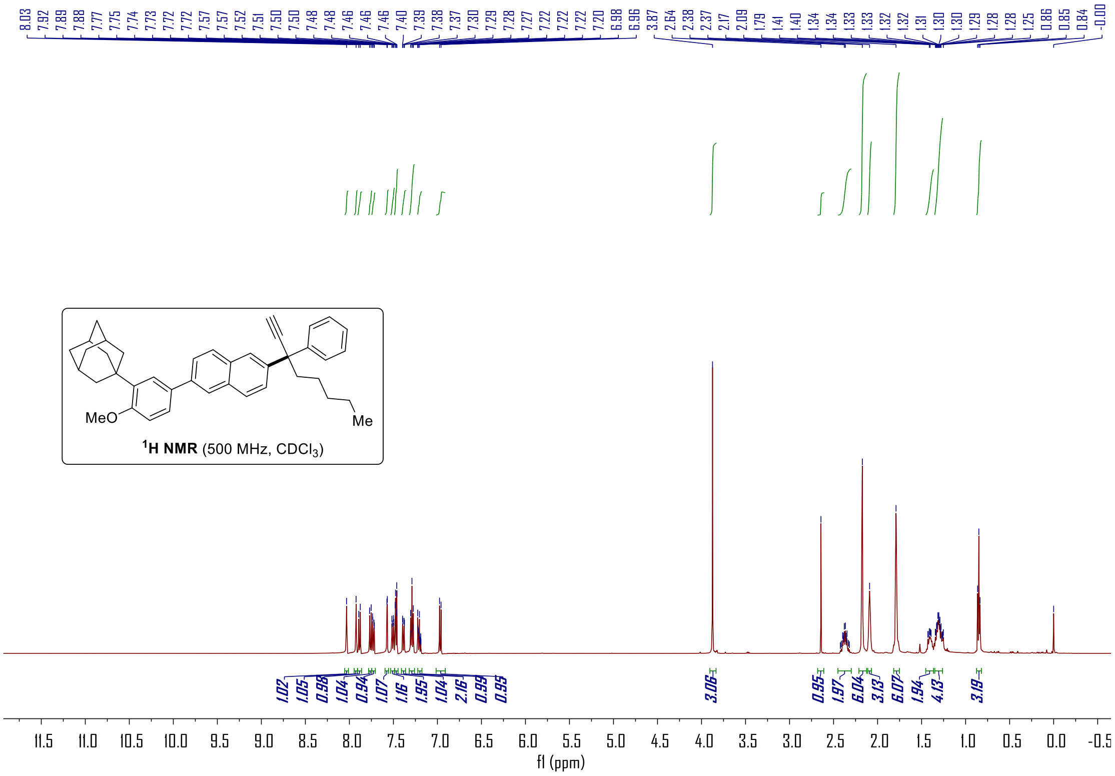

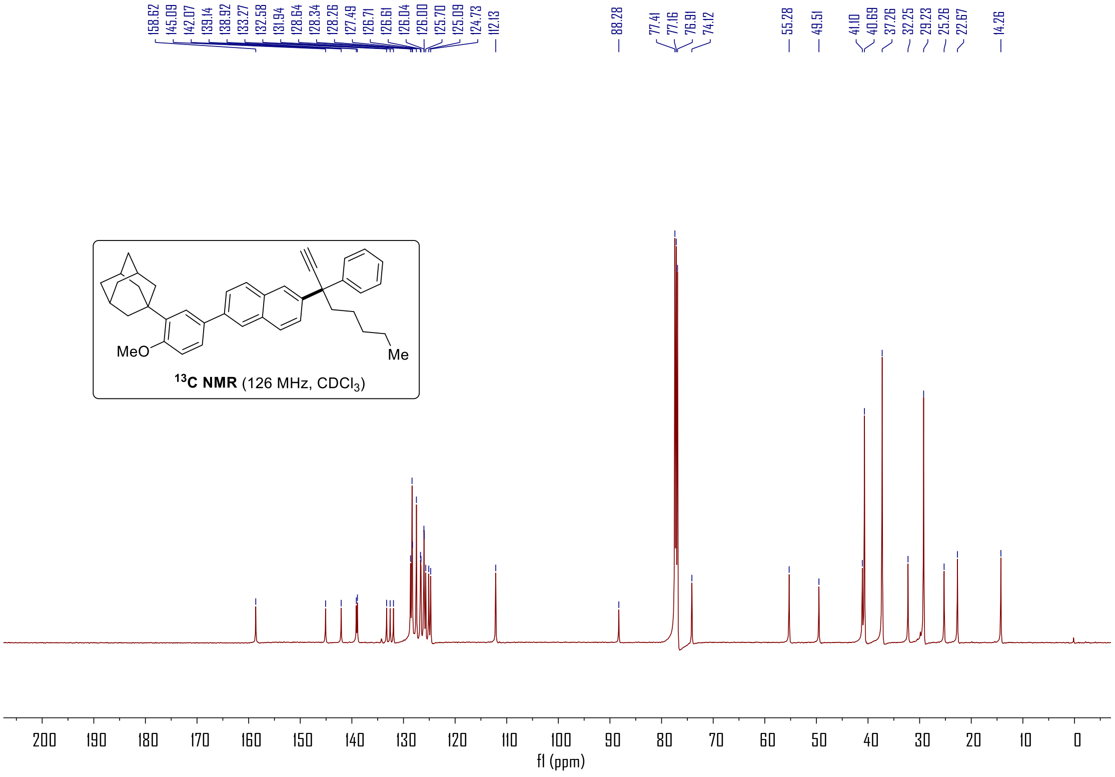

Estrone derivative (4aei)

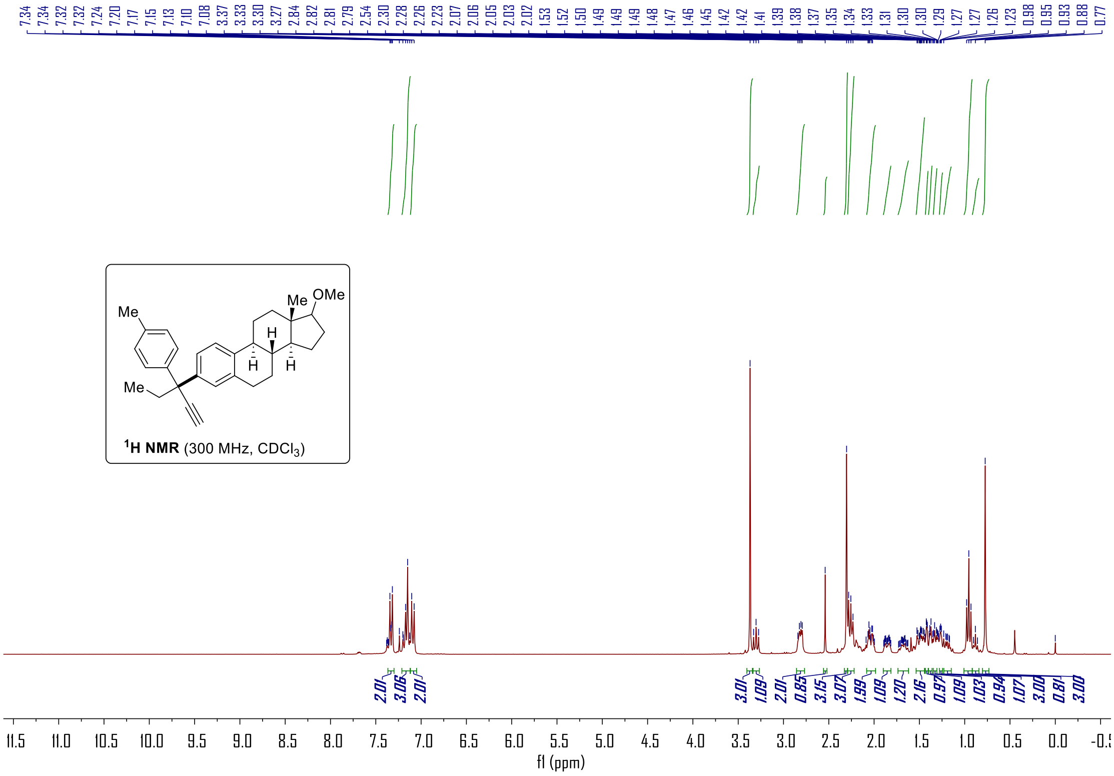

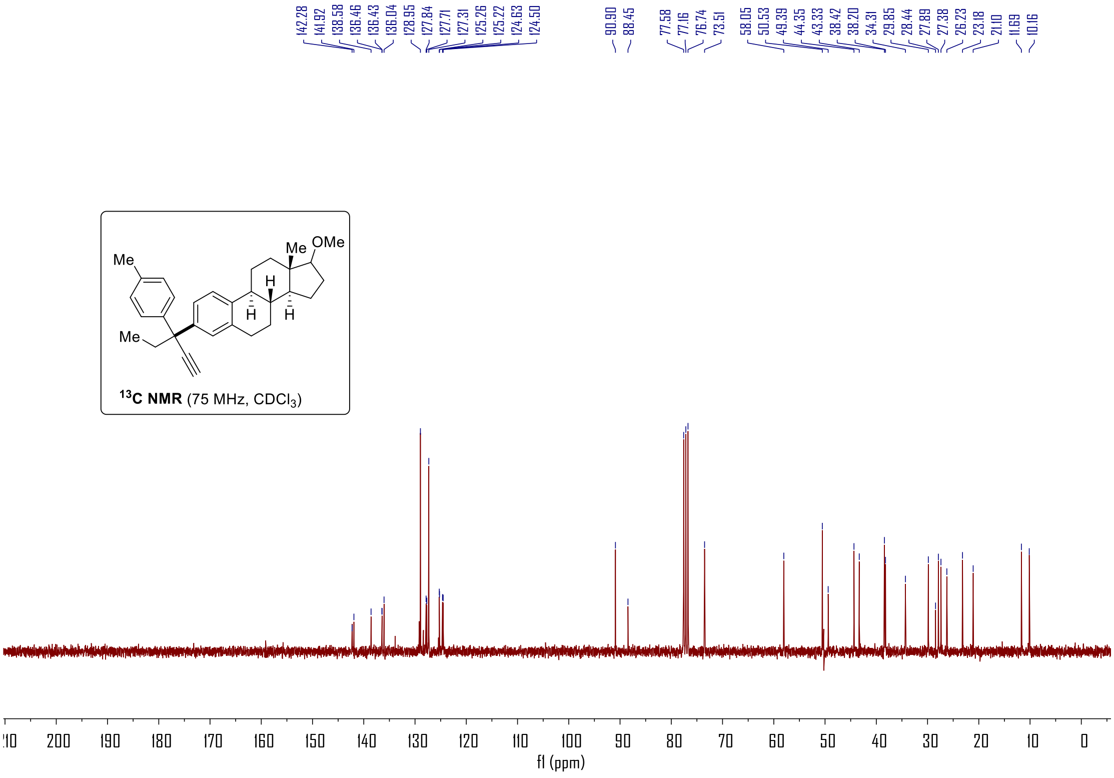

Blonanserin derivative (Antipsychotic) (4afg)

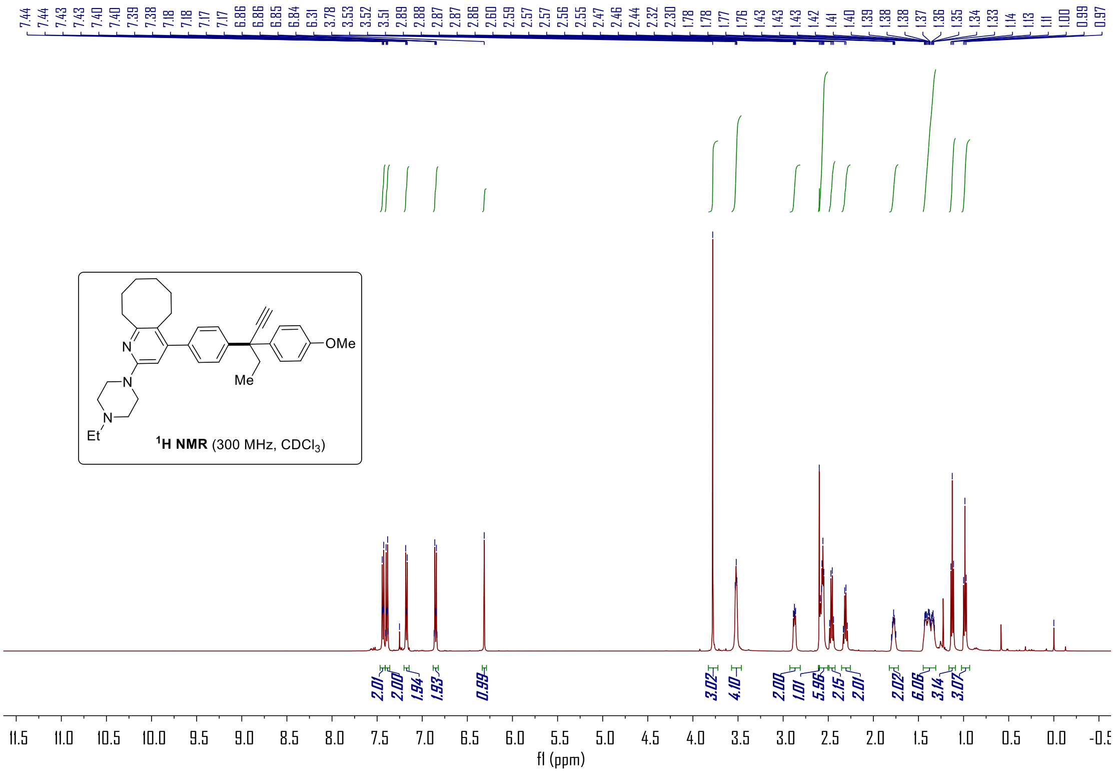

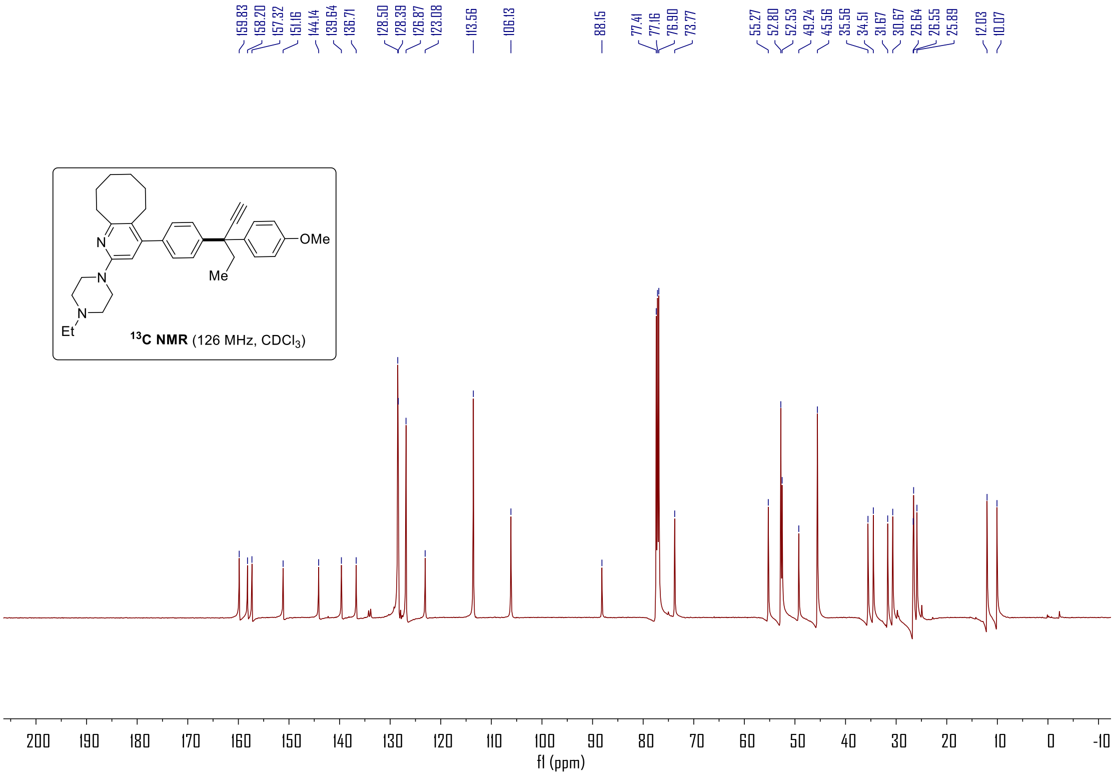

Liquid crystal material derivative (4agg)

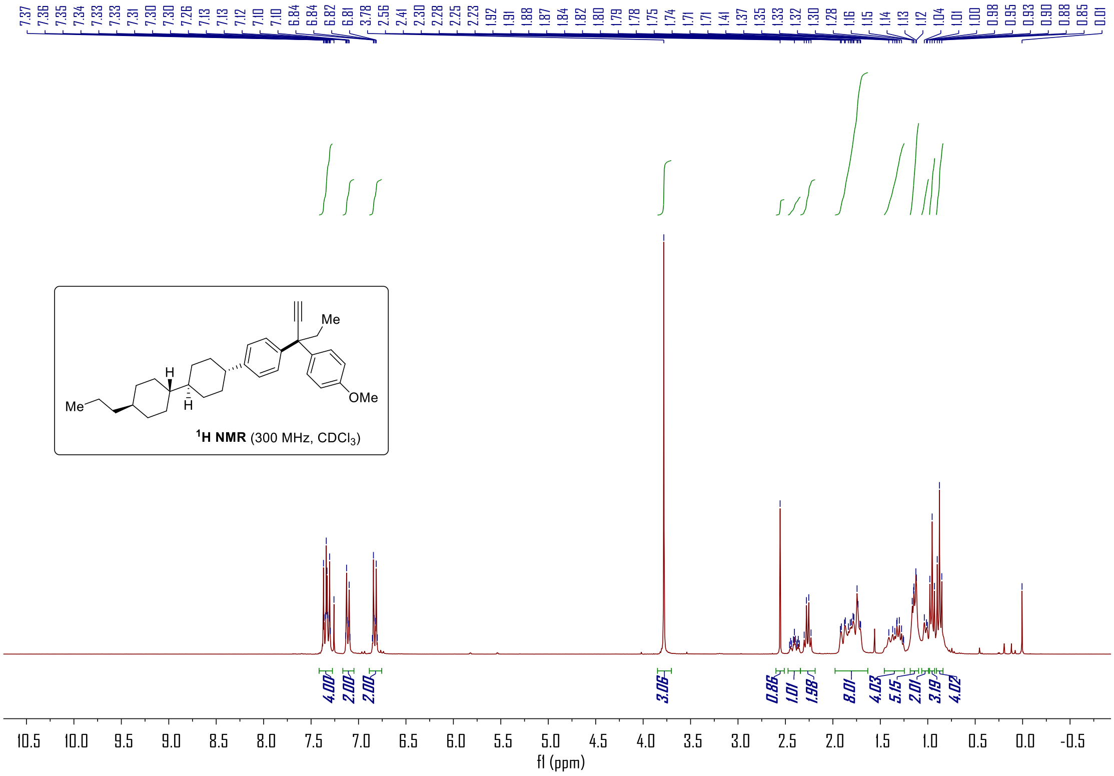

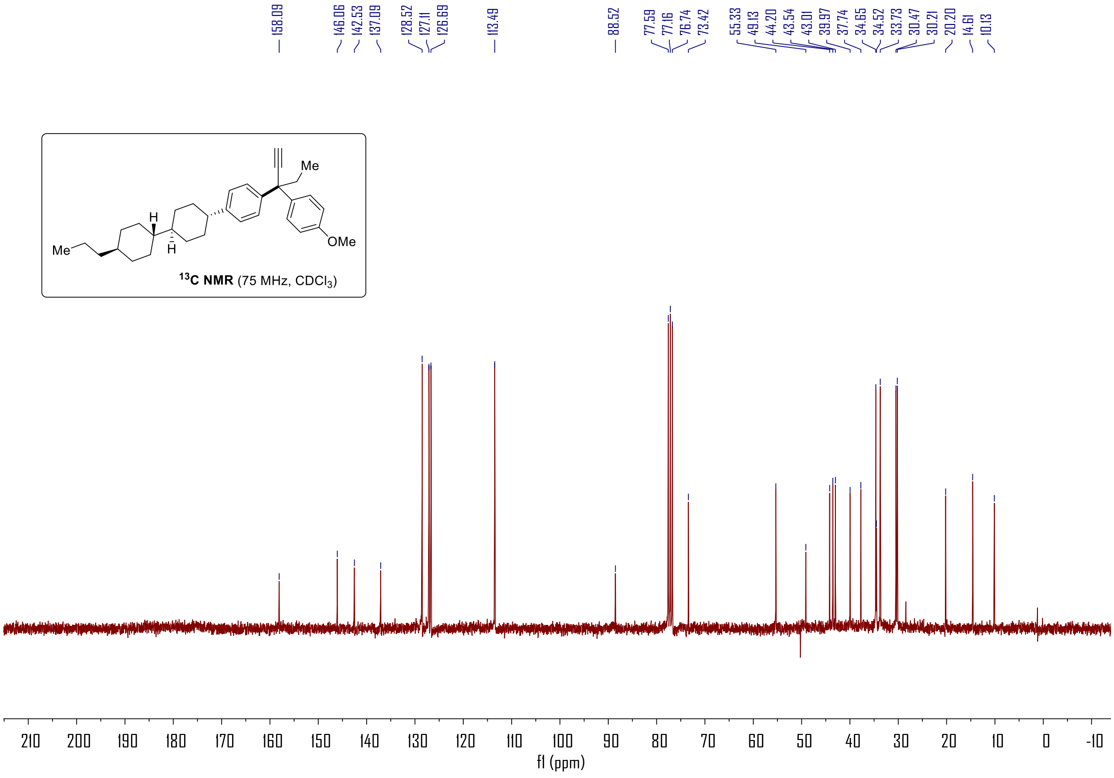

4-(biphenyl-4-yl)-1-(4-methoxyphenyl)-4-phenylhex-2-yn-1-ol (6)

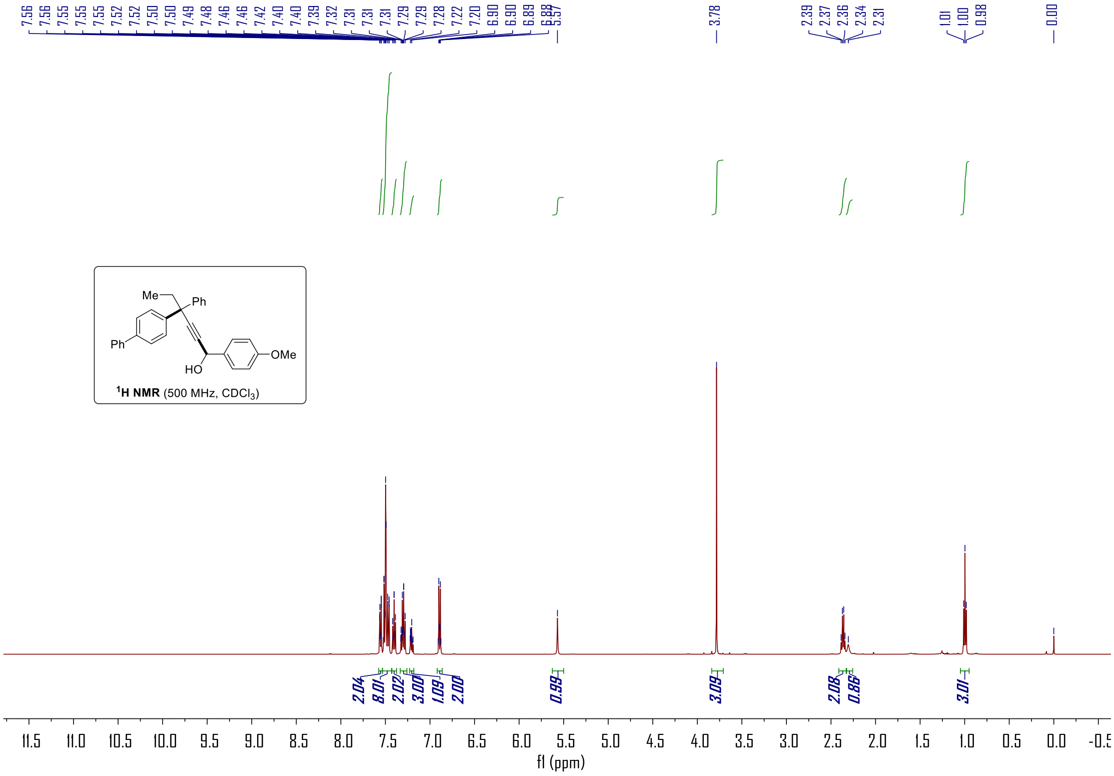

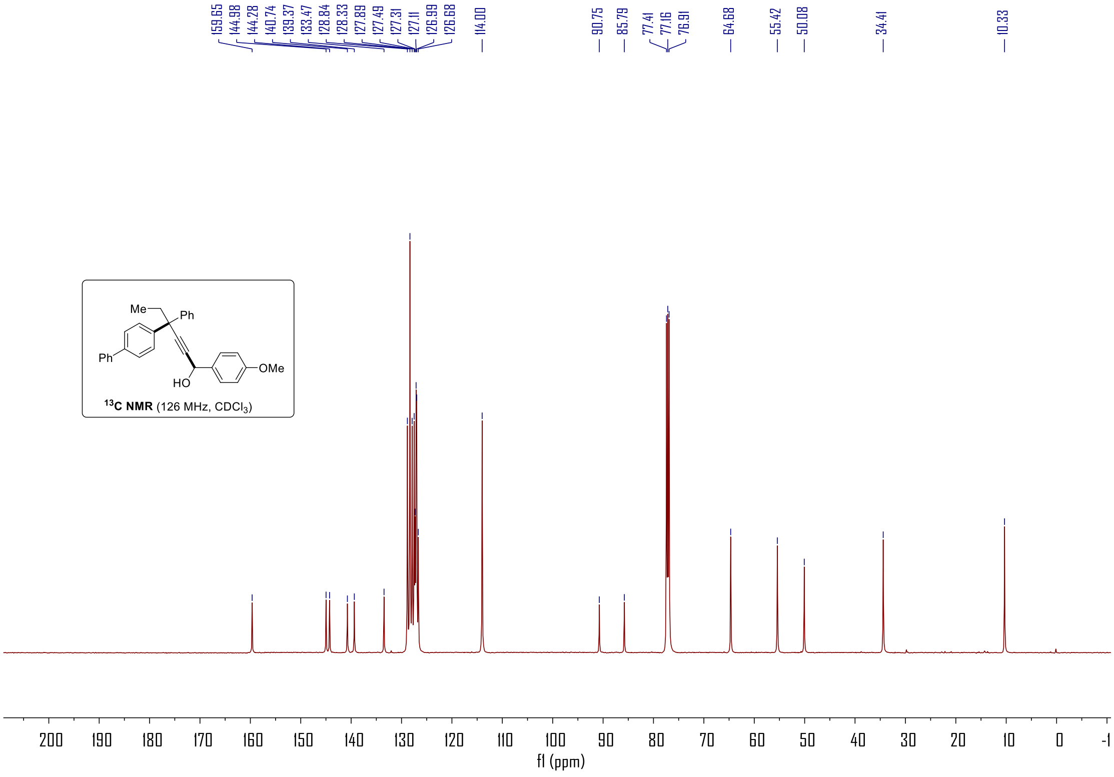

4-(3-Phenylpent-1-yn-3-yl-1-d)biphenyl (*d*-4aa)

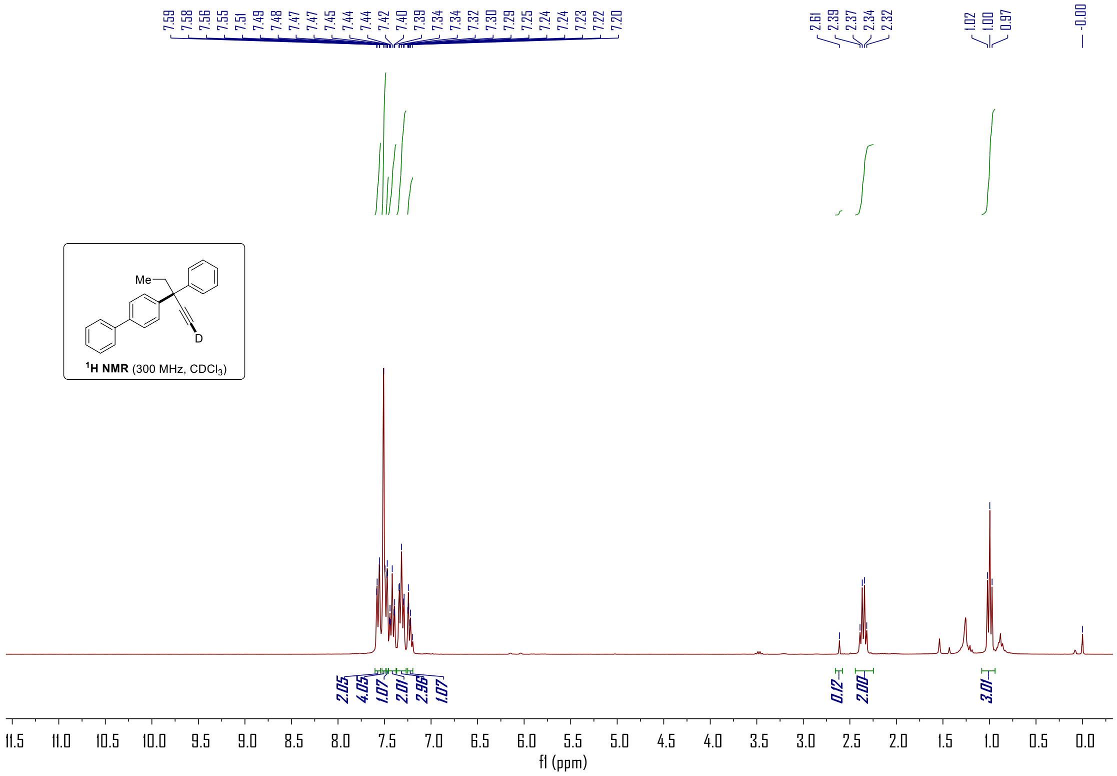

4-(1-Bromo-3-(4-methoxyphenyl)pent-1-yn-3-yl)phenyl (7)

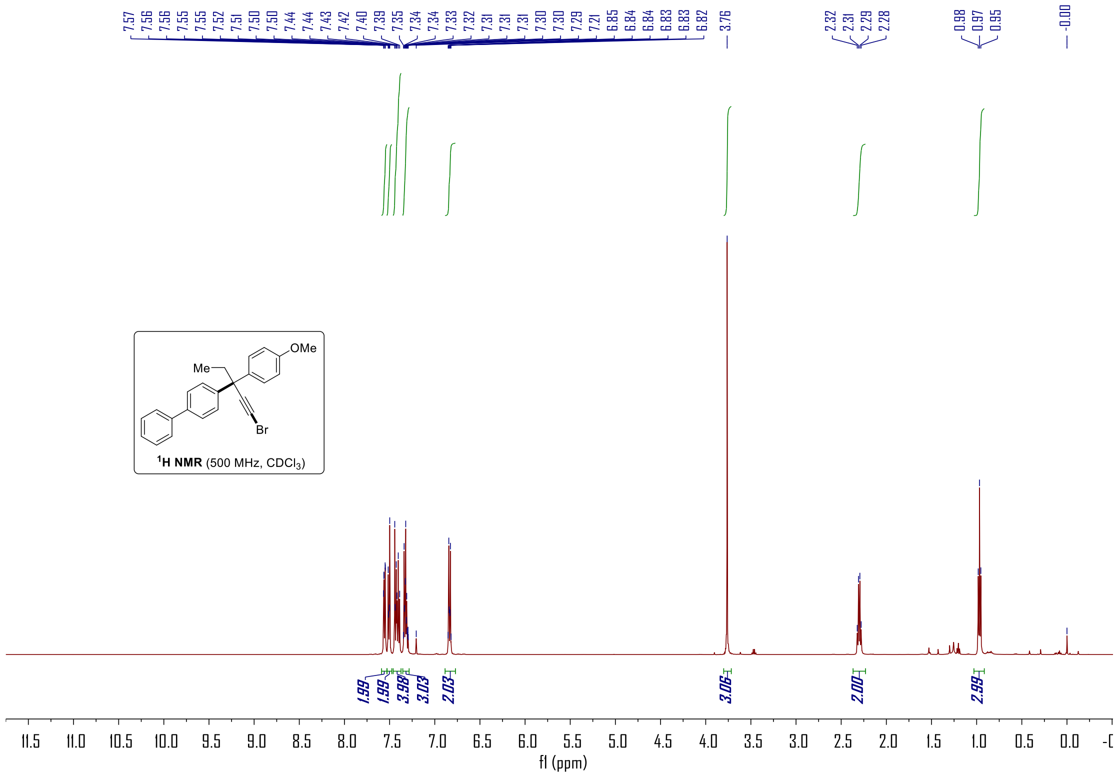

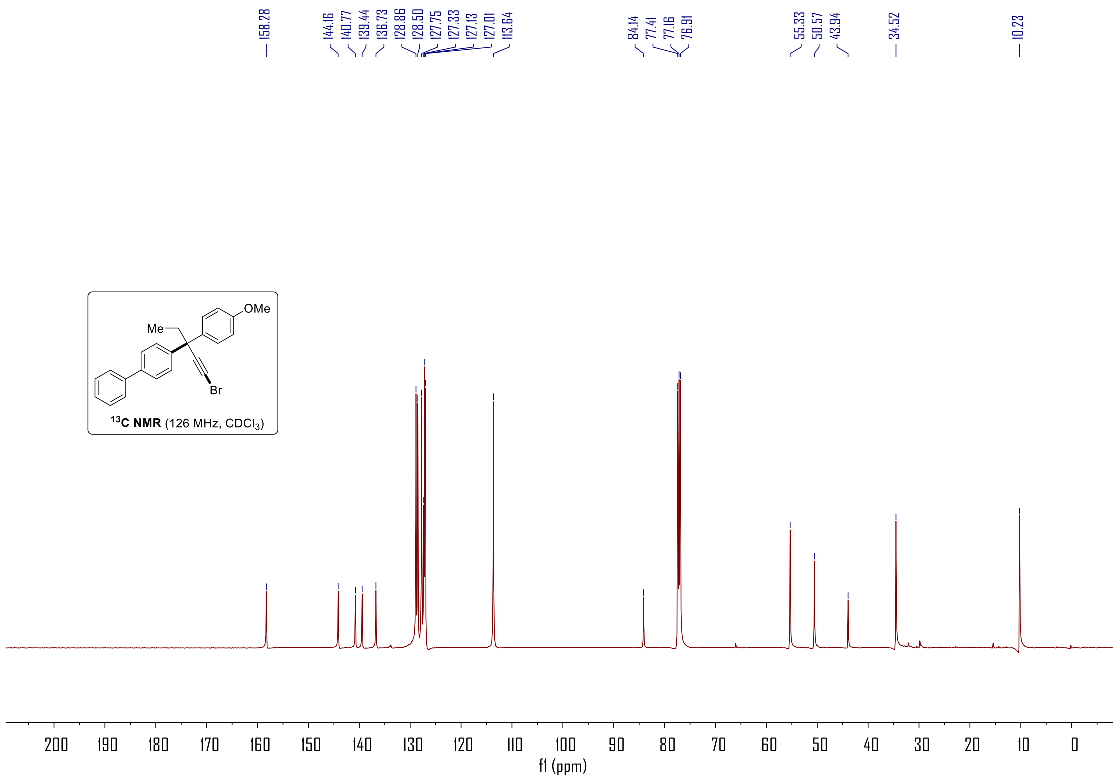

2-(3-(Biphenyl-4-yl)-3-phenylpent-1-yn-1-yl)-4-fluorobenzaldehyde (8)

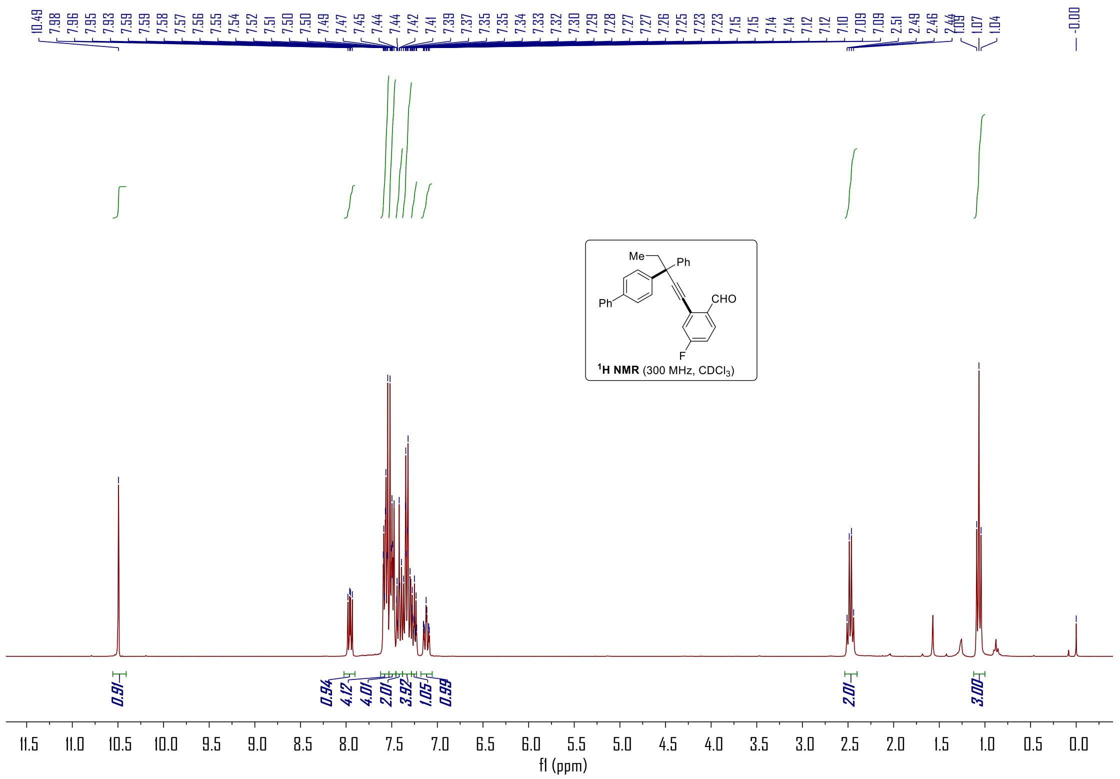

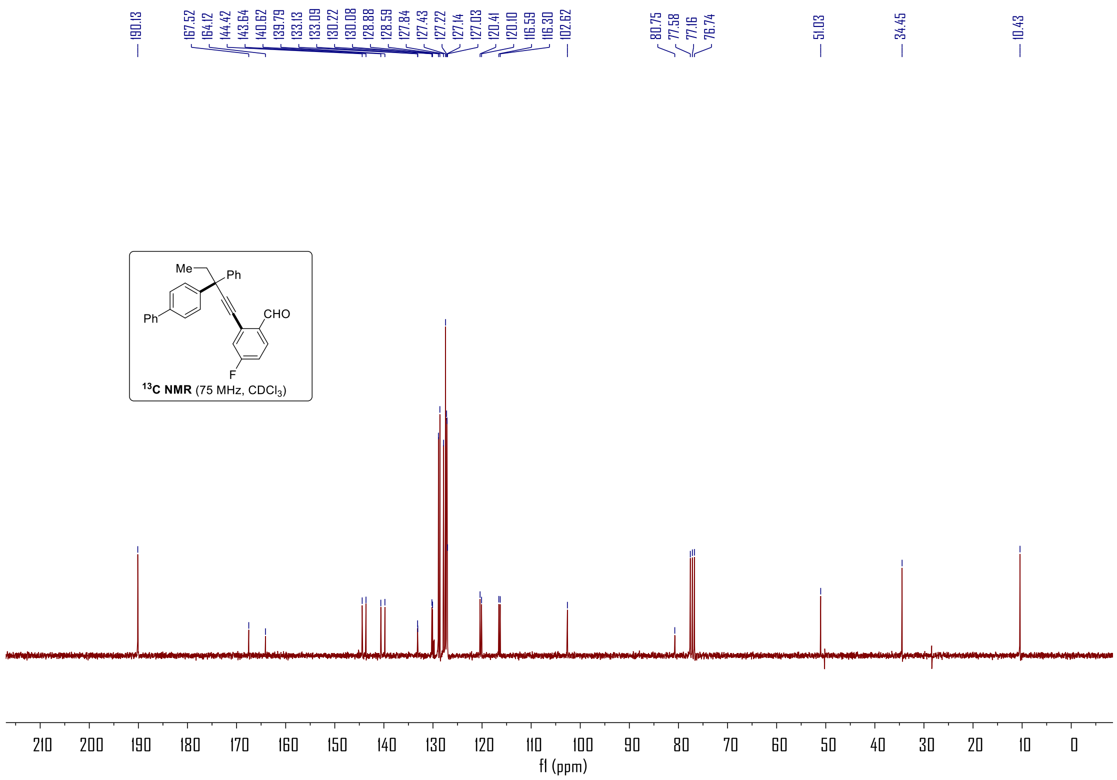

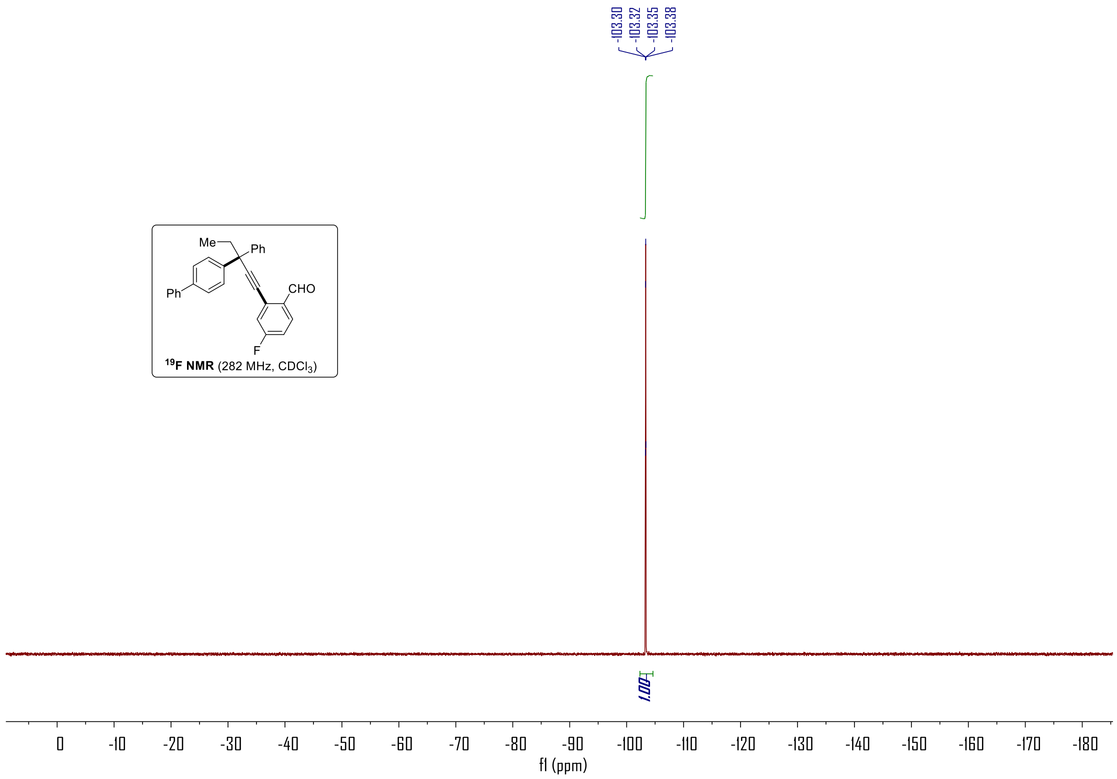

(3-(Biphenyl-4-yl)-3-phenylpent-1-yn-1-yl)trimethylsilane (9)

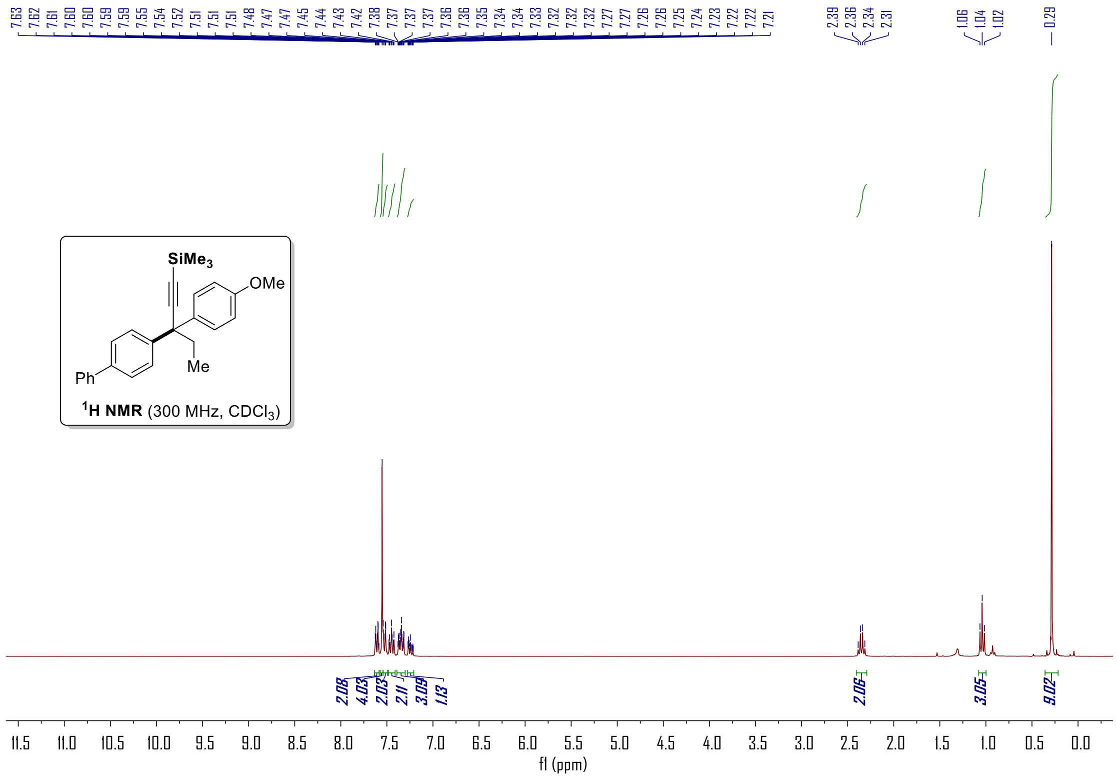

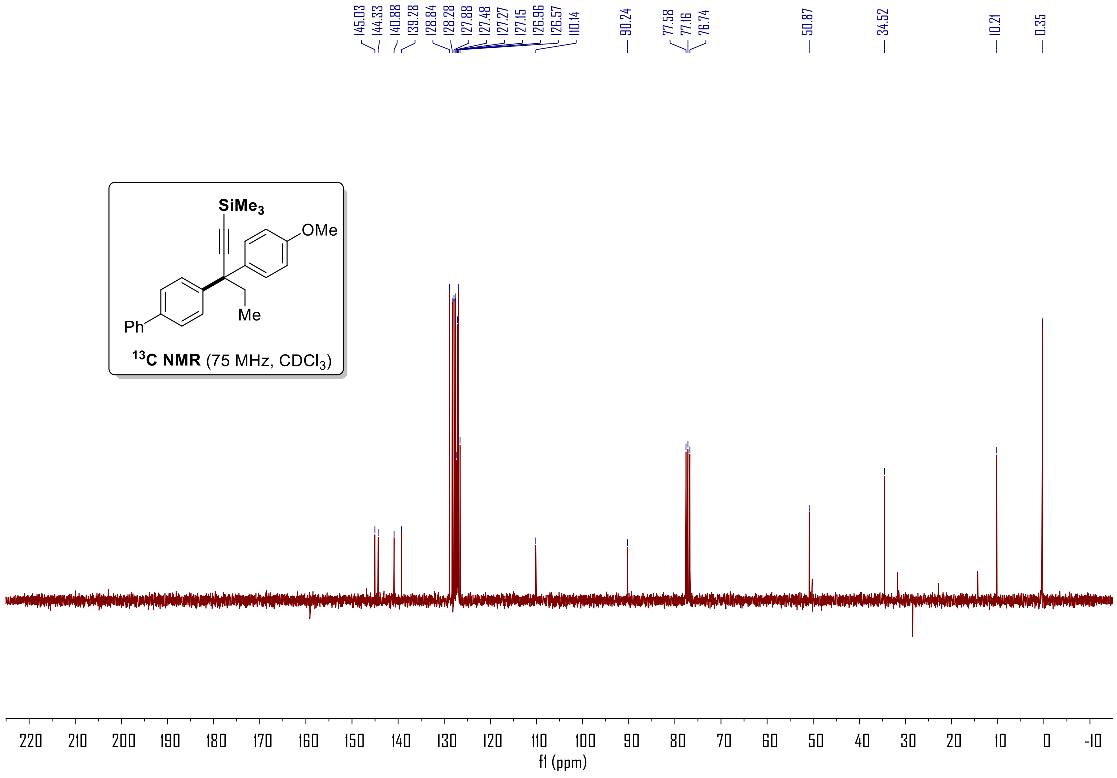

4-(3-Phenylpentan-3-yl)biphenyl (10)

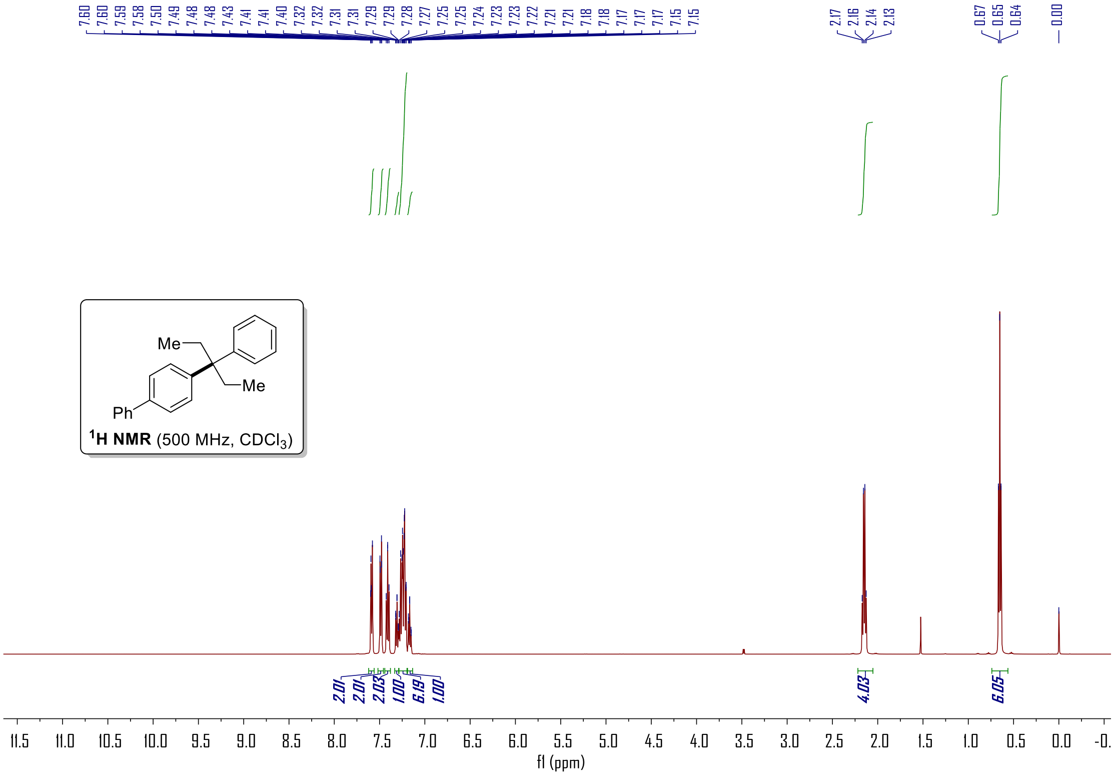

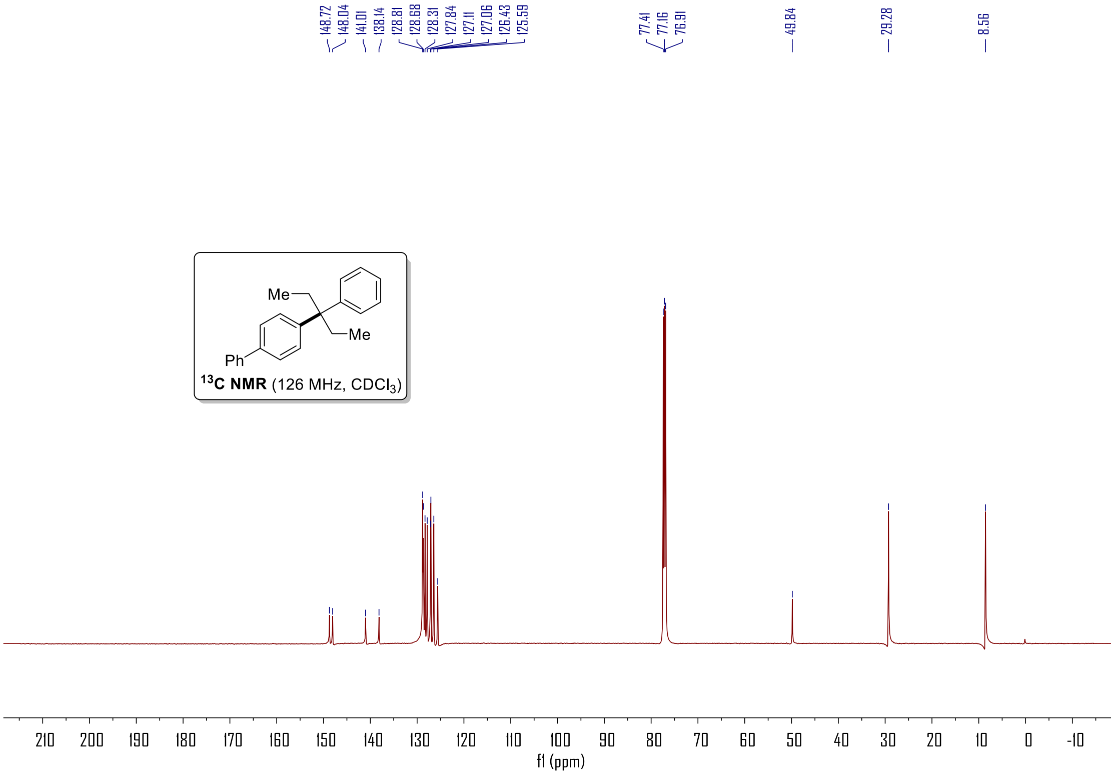

4-(1-Cyclopropyl-1-phenylprop-2-yn-1-yl)biphenyl (4ar)

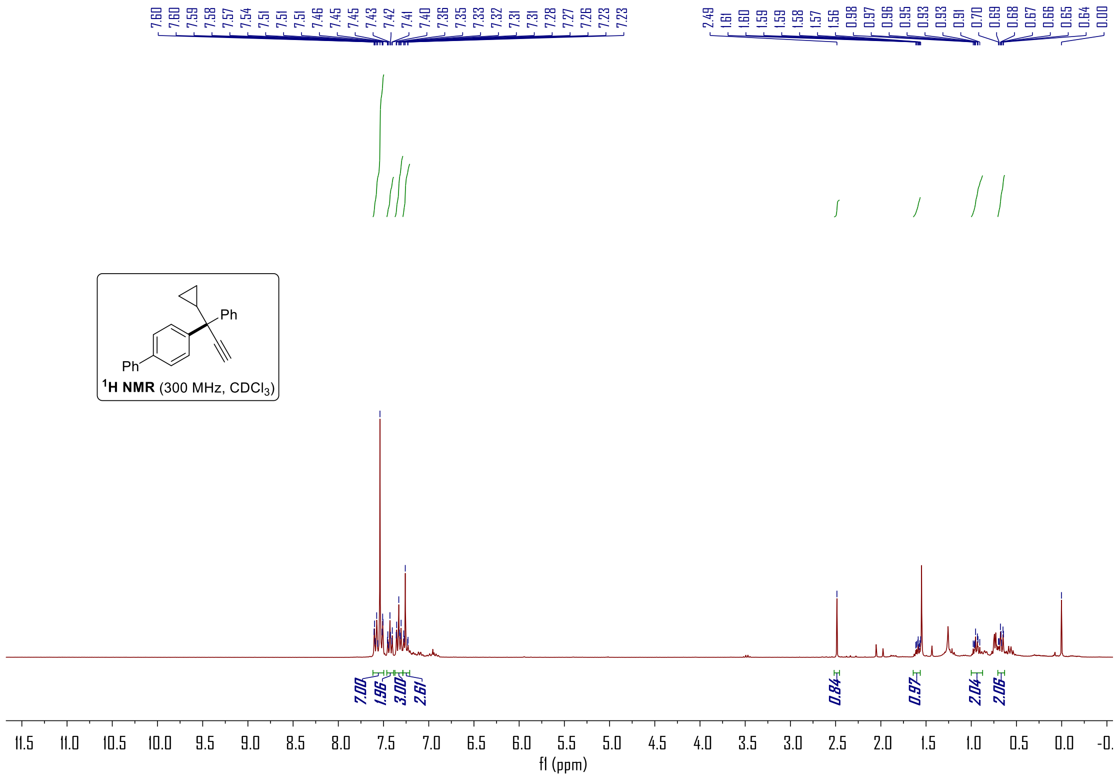

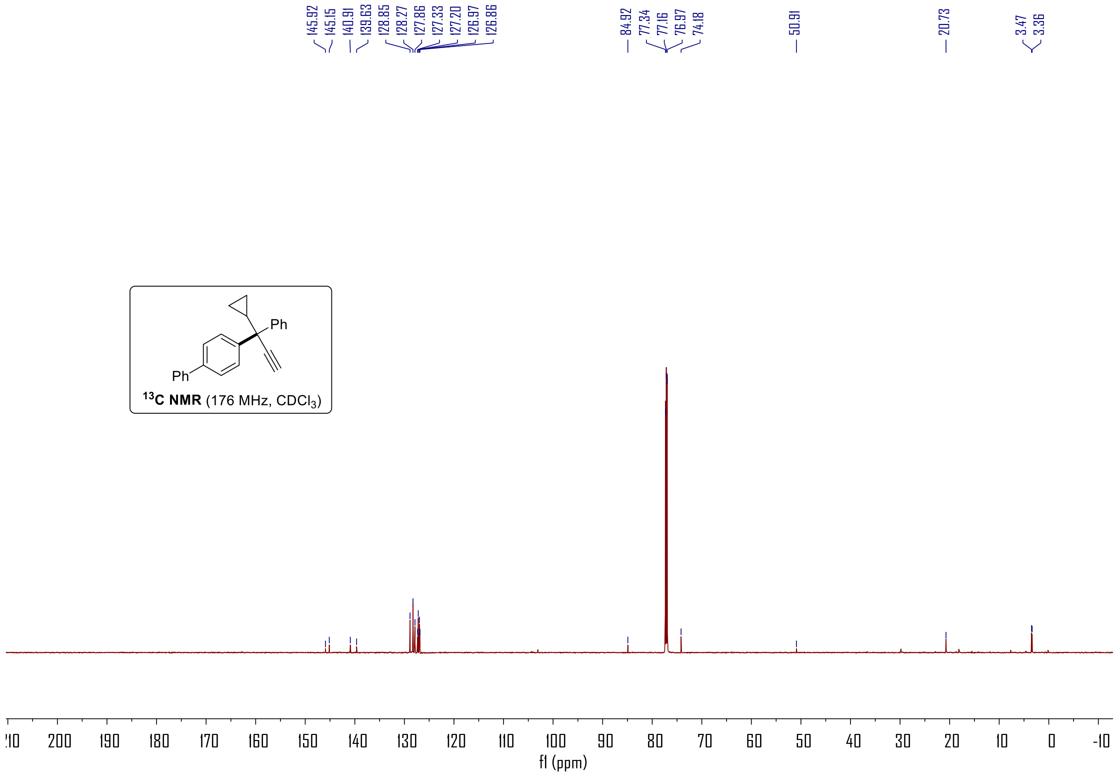

4-(3-(4-Methoxyphenyl)pent-1-yn-3-yl)biphenyl (*d*-4ag)

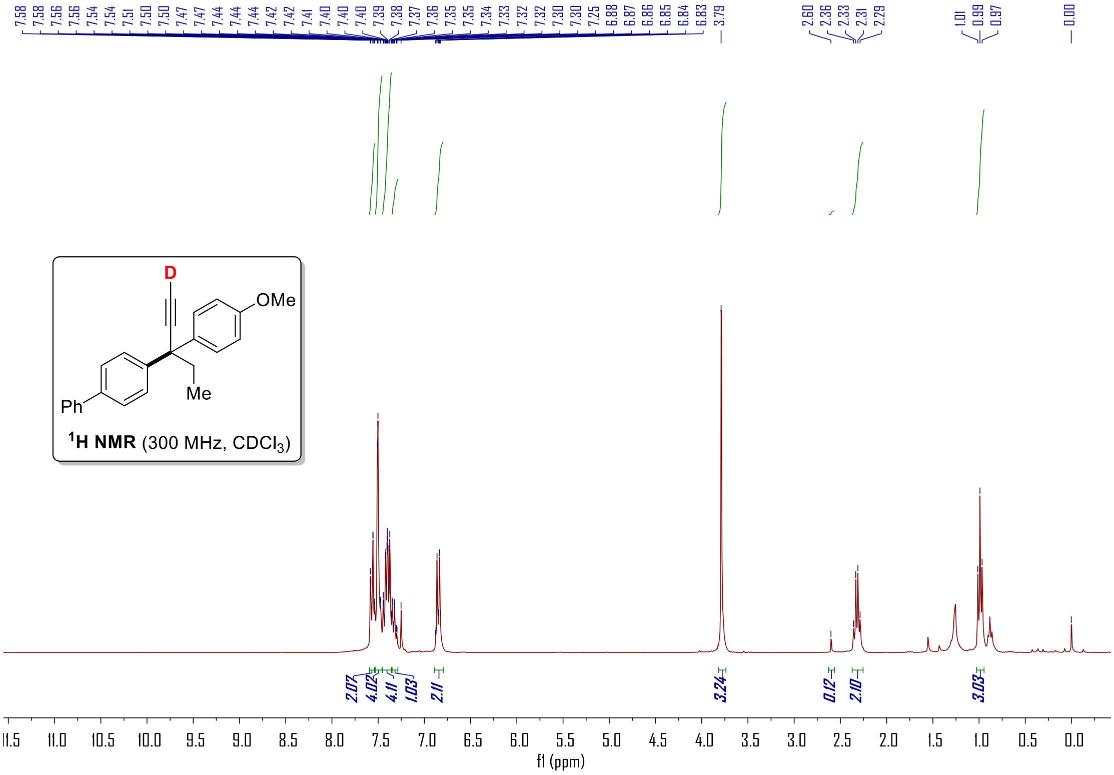

1-Methoxy-4-(pent-1-yn-3-yl)benzene (15)

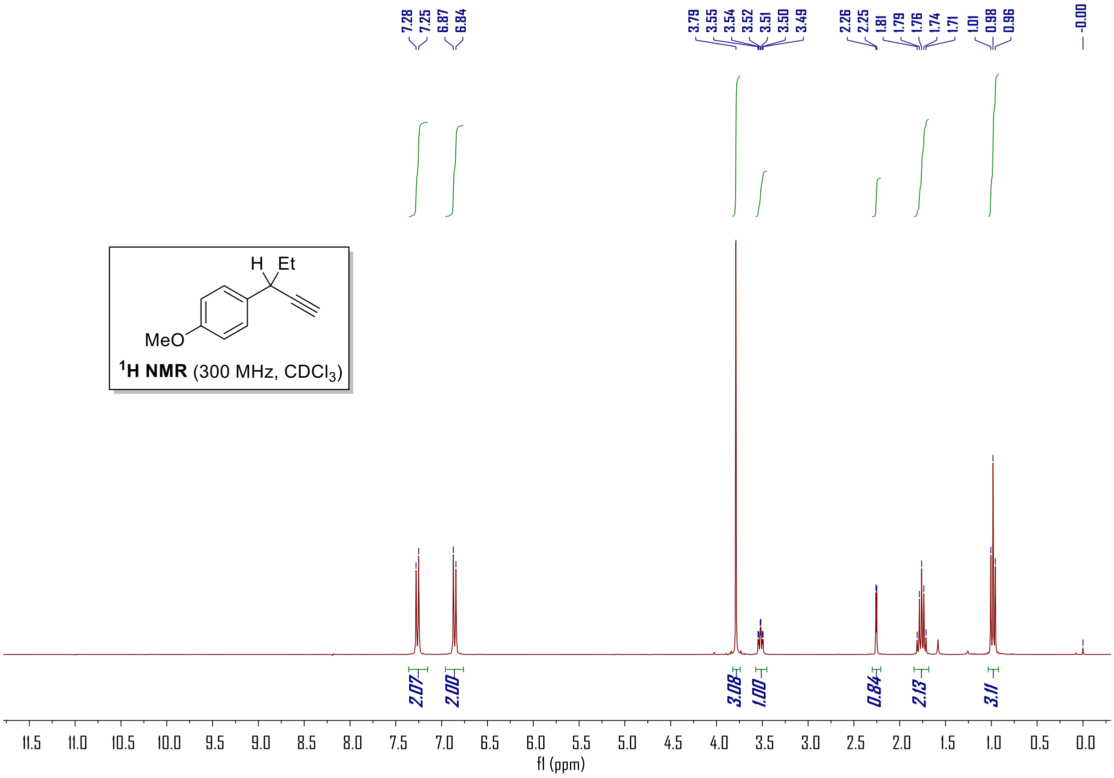

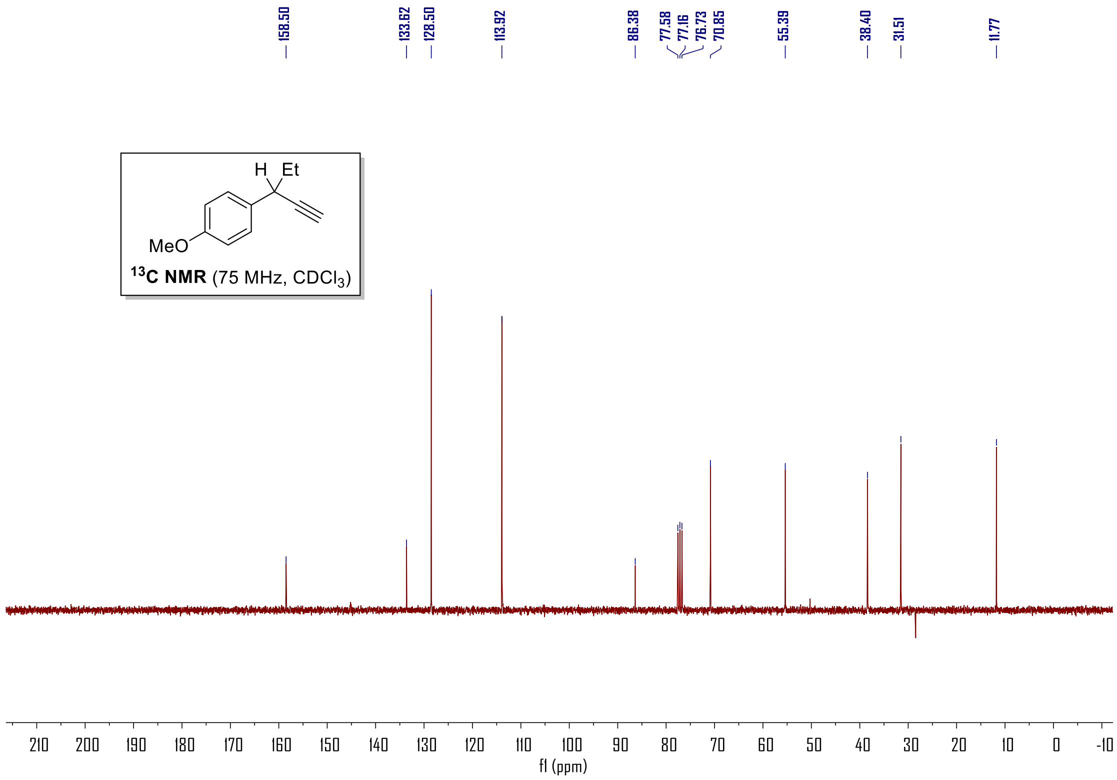
158.50
133.62
128.50
113.92
86.38
77.58
77.16
76.73
70.85
55.39
38.40
31.51
11.77
H
Et
MeO
^{13}C NMR (75 MHz, CDCl$_3$)
210
200
190
180
170
160
150
140
130
120
110
100
90
80
70
60
50
40
30
20
10
0
-10
f1 (ppm)

8. References

1. (*a*) B. Cui, S. Jia, E. Tokunaga and N. Shibata, *Nat. Commun.*, 2018, **9**, 4393–4400. (*b*) J. Zhou, B. Jiang, Y. Fujihira, Z. Zhao, T. Imai and N. Shibata, *Nat. Commun.*, 2021, **12**, 3749–3757. (*c*) J. Zhou, Z. Zhao, B. Jiang, K. Yamamoto, Y. Sumii and N. Shibata, *Chem. Sci.,* 2023, **14**, 4248–4256. (*d*) J. Zhou, Z. Zhao and N. Shibata, *Nat. Commun.,* 2023, **14**, 1847–1855.
2. A. Kumar, B. S. Bhakuni, C. D. Prasad, S. Kumar and S. Kumar, *Tetrahedron,* 2013, **69**, 5383–5392.
3. X. Yuan, J. F. Yao and Z. Y. Tang, *Org. Lett.,* 2017, **19**, 1410–1413.
4. J. Wang, H. Lu, Y. He, C. Jing and H. Wei, *J. Am. Chem. Soc.,* 2022, **144**, 22433–22439.
5. K. S. Rao, K. N. Rao, T. U. S. Sastry, P. Muralikrishna and A. Jayashree, *Asian J. Chem.,* 2014, **26**, 5928–5930.
6. I. S. Kim, G. R. Dong and Y. H. Jung, *J. Org. Chem.,* 2007, **72**, 5424–5426.
7. S. Dutta, S. Shandilya, S. Yang, M. P. Gogoi, V. Gandon and A. K. Sahoo, *Nat. Commun.,* 2022, **13**, 1360–1369.
8. Y. Yuan, X. Zhang, H. Qian and S. Ma, *Chem. Sci.,* 2020, **11**, 9115–9121.
9. Z. Li, L. Zhang, M. Nishiura, G. Luo, Y. Luo and Z. Hou, *ACS Catal.,* 2020, **10**, 11685–11692.
10. N. Zhu, F. Wang, P. Chen, J. Ye and G. Liu, *Org. Lett.,* 2015, **17**, 3580–3583.
11. Y. Ye, I. Kevlishvili, S. Feng, P. Liu and S. L. Buchwald, *J. Am. Chem. Soc.,* 2020, **142**, 10550–10556.
12. Q. H. Li, J. W. Liao, Y. L. Huang, R. T. Chiang and H. M. Gau, *Org. Biomol. Chem.,* 2014, **12**, 7634–7642.
13. X. Yang, Y. She, Y. Chong, H. Zhai, H. Zhu, B. Chen, G. Huang and R. Yan, *Adv. Synth. Catal.,* 2016, **358**, 3130–3134.
14. J. L. Moreau and M. Gaudemar, *J. Organomet. Chem.,* 1976, **108**, 159–164.
15. Q. Jiang, H. Li and X. Zhao, *Org. Lett.,* 2021, **23**, 8777–8782.
16. A. Preinfalk, A. Misale and N. Maulide, *Chem. Eur. J.,* 2016, **22**, 14471–14474.
17. A. Nagy, L. Collard, K. Indukuri, T. Leyssens and O. Riant, *Chem. Eur. J.,* 2019, **2019**, 8705–8708.
18. K. Sakaguchi, Y. Okada, T. Shinada and Y. Ohfune, *Tetrahedron Lett.,* 2008, **49**, 25–28.
19. Y. Y. Li and B. Gao, *Org. Lett.,* 2023, **25**, 2756–2760.
20. E. C. Liu and J. J. Topczewski, *J. Am. Chem. Soc.,* 2021, **143**, 5308–5313.
21. P. Ji, X. Liu, J. Xu, X. Zhang, J. Guo, W. Chen and B. Zhao, *Angew. Chem.*, *Int. Ed.*, 2022, **61**, e202206111.
22. Y. Guan, M. P. López-Alberca, Z. Lu, Y. Zhang, A. A. Desai, A. P. Patwardhan, Y. Dai, M. J. Vetticatt and W. D. Wulff, *Chem. Eur. J.,* 2014, **20**, 13894–13900.
23. G. Zhang, T. Xiong, Z. Wang, G. Xu, X. Wang and Q. Zhang, *Angew. Chem.*, *Int. Ed.*, 2015, **54**, 12649–12653.
24. M. R. Uehling, S. T. Marionni and G. Lalic, *Org. Lett.,* 2012, **14**, 362–365.
25. Y. Kobayashi, Y. Takashima, Y. Motoyama, Y. Isogawa, K. Katagiri, A. Tsuboi and N. Ogawa, *Chem. Eur. J.,* 2021, **27**, 3779–3785.
26. A. M. Caporusso, A. Zampieri, L. A. Aronica and D. Banti, *J. Org. Chem.,* 2006, **71**, 1902–1910.
27. X. Jiang, B. Han, Y. Xue, M. Duan, Z. Gui, Y. Wang and S. Zhu, *Nat. Commun.,* 2021, **12**, 3792.
28. J. Maury, S. Jammi, F. Vibert, S. R. A. Marque, D. Siri, L. Feray and M. Bertrand, *J. Org. Chem.*, 2012, **77**, 9081–9086.
